# Bone marrow mesenchymal stem cell-derived exosomal miR-21 protects C-kit^+^ cardiac stem cells from oxidative injury through the PTEN/PI3K/Akt axis

**DOI:** 10.1371/journal.pone.0191616

**Published:** 2018-02-14

**Authors:** Bei Shi, Yan Wang, Ranzhun Zhao, Xianping Long, Wenwen Deng, Zhenglong Wang

**Affiliations:** Department of Cardiology, Affiliated Hospital of Zunyi Medical College, Zunyi, China; University of Cincinnati College of Medicine, UNITED STATES

## Abstract

Stem cell (SC) therapy for ischemic cardiomyopathy is hampered by poor survival of the implanted cells. Recently, SC-derived exosomes have been shown to facilitate cell proliferation and survival by transporting various proteins and non-coding RNAs (such as microRNAs and lncRNAs). In this study, miR-21 was highly enriched in exosomes derived from bone marrow mesenchymal stem cells (MSCs). Interestingly, exosomes collected from hydrogen peroxide (H_2_O_2_)-treated MSCs (H-Exo) contained higher levels of miR-21 than exosomes released from MSCs under normal conditions (N-Exo). The pre-treatment of C-kit^+^ cardiac stem cells (CSCs) with H-Exos resulted in significantly increased levels of miR-21 and phosphor-Akt (pAkt) and decreased levels of PTEN, which is a known target of miR-21. AnnexinV-FITC/PI analysis further demonstrated that the degree of oxidative stress-induced apoptosis was markedly lower in H-Exo-treated C-kit^+^ CSCs than that in N-Exo-treated cells. These protective effects could be blocked by both a miR-21 inhibitor and the PI3K/Akt inhibitor LY294002. Therefore, exosomal miR-21 derived from H_2_O_2_-treated MSCs could be transported to C-kit^+^ cardiac stem cells to functionally inhibit PTEN expression, thereby activating PI3K/AKT signaling and leading to protection against oxidative stress-triggered cell death. Thus, exosomes derived from MSCs could be used as a new therapeutic vehicle to facilitate C-kit^+^ CSC therapies in the ischemic myocardium.

## 1. Introduction

Recently, cardiac stem cells (CSCs) residing in the adult mammalian heart have emerged as one of the most promising stem cell types for cardiac regeneration and repair[1–7]. However, the poor engraftment and viability of CSCs hamper functional improvements and optimal cardiac outcomes[8–10]. Preconditioning stem cells using various strategies could significantly enhance CSC survival after adoptive transfer in myocardial infarction patients[11–14]. Exosomes released from cells have been recently shown to mediate cell-cell communication to ensure information transfer from donor cells to recipient cells and allow cells to react to environmental changes[15]. These exosomes constitute a delicate and complex system that can be used to control tissue regeneration and cell protection and survival[16–18].

Exosomes are membrane vesicles 30–100 nm in diameter that are released from many cell types under specific physiological or pathological states. Exosomes contain many protein factors, mRNAs, miRNAs, lncRNAs and other nutritional elements. These cargoes are selectively wrapped into the microbubble structure and finally secreted into the extracellular environment via exosomes[19, 20]. However, the contents of exosomes vary across different cell types and under different pathophysiological conditions, which may generate completely different outcomes in recipient cells[21, 22]. Hence, investigating the biological functions of exosomes under specific pathological conditions is imperative. MSC-released exosomes have been shown to improve cardiac function after myocardial infarction[18, 23]. Moreover, an injection of exosomes from exogenous MSCs could recruit endogenous CSCs to the ischemic and border zones of infarcted hearts and promote their expansion[24]. Additionally, exosomes released from MSCs could stimulate the proliferation, migration, and angiogenic potency of CSCs *in vitro* and *in vivo*[16]. Considering the potential therapeutic effects of MSC-exosomes (MSC-Exo) in cardioprotection and cell therapy, we sought to determine whether C-kit^+^ CSCs preconditioned with MSC-Exos could enhance survival and function under oxidative stress conditions.

miRNAs, which are among the many exosome cargo types, have been confirmed to play a pivotal role in improving the undesirable consequences associated with acute myocardial infarction[25]. miRNAs are endogenous single-stranded non-coding RNAs consisting of 20–22 nucleotides that play critical roles in mRNA inhibition and degradation[26]. miRNAs have been shown to be involved in the regulation of CSC apoptosis[8, 27]. miRNAs released by MSC-Exos may also regulate the proliferation, differentiation and survival of CSCs[16]. However, whether MSC-Exo-derived miRNAs protect against apoptosis induced by H_2_O_2_ in C-kit^+^ CSCs and specific miRNAs that play critical roles remain unknown. According to gain-of-function studies, miR-21 reduces cardiomyocyte apoptosis induced by oxidative stress [28, 29]. One study have confirmed that MiR-21 modulates the immunoregulatory function of MSCs by controlling the PTEN[30]. Furthermore, It is also reported that miR-21 via regulating the PTEN/HIF-1α/VEGF-A signaling cascade to enhances the therapeutic effects of human multipotent cardiovascular progenitors[31]. These studies have identified a potential exosomal miRNA target gene that likely regulates C-kit^+^ CSC apoptosis under oxidative stress conditions.

Phosphatase and tensin homolog deleted on chromosome ten (PTEN) is a tumor suppressor gene that is involved in the regulation of cell proliferation, migration, differentiation and invasion in a variety of tumor cells[32, 33]. PTEN partially functions through the pro-survival pathway by inhibiting the phosphorylation of Akt to its active form (p-Akt)[33]. The inactivation of PTEN activates Akt signaling, which reduces apoptosis and increases survival[34–37]. PTEN is a well-documented target gene of miR-21[30, 38–40].Moreover, miR-21 promotes cell proliferation via PTEN-dependent PI3K/Akt activation in cancer cells[41–45]. Additionally, in our previous study, miR-21 protected C-kit^+^ CSCs from H_2_O_2_-induced apoptosis and increases cell proliferationin partially through the PTEN/PI3K/ Akt pathway[46–47]. The current study investigated the protective effects of MSC-Exos on C-kit^+^ CSCs under oxidative stress. These effects are mainly mediated through the transmission of exosomal miR-21, which inhibits PTEN and activates the PI3K/Akt pathway in C-kit^+^ CSCs. These findings provide a potential cellular therapeutic strategy for ischemic cardiomyopathy.

## 2. Materials and methods

### 2.1. Animals

Sprague-Dawley rats (males and females, approximately 3 weeks old, 45–60 g) were purchased from the Third Military Medical University (Chongqing, China) and housed at Zunyi Medical College. All experimental procedures were performed according to the “Guide for the Care and Use of Laboratory Animals” in China and approved by the local Experimental Animal Care and Use Committee.

### 2.2. Materials

Collagenase type II was obtained from Sigma (USA). Trypsin was obtained from Gibco (USA). Penicillin and streptomycin were obtained from Sorlabio (China). Ham's/F-12 medium and fetal bovine serum were both purchased from HyClone (USA). Fibroblast growth factor was obtained from PeproTech (USA). Leukocyte inhibitory factor was obtained from Gibco (USA). The rabbit anti-rat C-kit^**+**^ primary antibody was supplied by Biorbyt (UK). The M-280 beads conjugated with sheep anti-rabbit secondary antibody were obtained from Dynal Biotech (Norway). The PE-conjugated anti-CD34 and anti-CD45, APC conjugated anti-CD29, and anti-CD90 primary antibodies were obtained from BioLegend (USA). The miR-21 mimics, miR-21 inhibitors and the negative control were synthesized by RIBOBIO (China). EXO quick TC was obtained from System Biosciences. SiRNA-PTEN and the scrambled siRNA were synthesized by GeneCopoeia (MD). The lentivirus and empty vector were synthesized by HANBIO (China). Lipofectamine 2000 was obtained from Invitrogen (USA). The primers and miRNA reverse transcript and qRT-PCR kits were obtained from Sangon Biotech (China). The anti-β-actin, anti-caspase-3, anti-cleaved-caspase-3, anti-PTEN, anti-P-Akt, and anti-Akt primary antibodies and additional secondary antibodies were obtained from Boster (China). The anti-CD63, anti-CD9, and anti-Hsp70 antibodies were purchased from Abcam (USA). DiI was obtained from Invitrogen (USA). The Annexin V-FITC apoptosis detection kit was obtained from Solarbio (China). The In-situ cell death detection kit was obtained from Sigma(USA). LY294002 (PI3K inhibitor) was obtained from Beyotime Technology (China). The unlisted reagents were of analytical grade.

### 2.3. In vitro culture of C-kit^+^ cells

CSCs were isolated[48] and purified[3] using previously published methods with some modifications. The rats were deeply anesthetized with sevoflurane, and the atrial appendage was sliced and digested with 0.1% collagenase type II (Sigma, USA). After a 40-min digestion at 37°C, the cells were collected by sedimentation at 1200 rpm for 5 min. Then, the cells from the atrial appendage were incubated in a humidified chamber in Ham’s F12 medium containing 10% fetal bovine serum (FBS), 1% penicillin and streptomycin, 1% L-glutamine, 20 ng/ml human recombinant fibroblast growth factor, 20 ng/ml leukocyte inhibitory factor, and 10 ng/ml epidermal growth factor (EGF). After reaching >90% confluence, the cells were resuspended by trypsinization. Subsequently, the CSCs were incubated with a rabbit anti-C-kit antibody (1:250 in F12 medium) for 1 h and sorted with anti-rabbit secondary antibody-conjugated 2.8 μm magnetic beads (Dynal Biotech, Norway) for 30 min as instructed by the manufacturer's protocol. The purified C-kit^+^ CSCs were cultured in the previously mentioned F12 medium. Flow cytometry (FCM) was performed to confirm the surface markers on the C-kit^+^ CSCs. The cells were incubated with the fluorochrome-conjugated anti-CD34-PE, anti-CD45-PE, and anti-C-kit primary antibodies and the anti-C-kit IgG-allophycocyanin (APC) secondary antibody (all from BioLegend, USA).

### 2.4. Isolation and culture of MSCs

The culture medium was used to flush all bone marrow cells from the femurs and tibias of rats (2–4 months old) sacrificed with a sevoflurane overdose as previously described[49]. Low glucose-Dulbecco’s modified Eagle’s medium(L-DMEM) (GIBCO) complete medium containing 15% FBS, 100 U/ml penicillin, and 100 U/ml streptomycin was used to resuspend the MSCs. Then, the cells were incubated in a humidified chamber. The first medium change was performed at 48 h to remove the non-adherent cells. Trypsin (0.25%, Sigma) was used to passage the cells at a ratio of 1:2 after reaching 90% confluence. FCM was used to analyze the MSC surface markers. The cells were incubated with the fluorochrome-conjugated anti-CD90-PE and anti-CD29 allophycocyanin (APC) or anti-CD45-PE primary antibodies (all from BioLegend, USA). MSCs between P3 and P5 were used for the subsequent experiments.

### 2.5. Purification and identification of MSC exosomes

The MSC-exosomes (MSC-Exos) extraction procedures were performed as previously described[23, 50]. The MSCs were cultured in L-DMEM supplemented with 10% FBS. Prior to use, all FBS was centrifuged at 100,000–110,000 g for 8 to 10 h to eliminate preexisting bovine-derived exosomes[7]. A 50-ml conditional culture medium containing 10% Exo-free fetal bovine serum (FBS) was used to culture the MSCs for 48 h. The supernatant was harvested and centrifuged at 500 g for 5 min and then 2000 g for 30 min at 4°C to remove cell debris. ExoQuick TC (System Biosciences) was applied to precipitate the exosomes according to the manufacturer’s instructions. Briefly, 50 ml supernatant were added to 10 ml ExoQuick-TC Exosomes Precipitation Solution. This cocktail was mixed well and refrigerated overnight. Subsequently, the cocktail was centrifuged at 1500 g for 30 min, and the supernatant was removed. The sediment was then centrifuged at 1500 g for 5 min and aspirated. Then, 50 μl phosphate-buffered saline (PBS) were used to resuspend the exosomes, and the resulting solution was stored at –80°C. The amount of MSC-Exo was detected by measuring the total protein content using a BCA protein assay kit (Pierce). Then, the exosomes were observed directly under a transmission electron microscope (Hitachi H7500 TEM, Tokyo, Japan). The MSC-Exos were also identified by Western blotting using the anti-CD63, anti-CD9, and anti-Hsp70 antibodies (all purchased from Abcam) previously described as specific exosome markers[51, 52].

### 2.6. Established H_2_O_2_-induced oxidative stress model in C-kit^+^ CSCs and MSCs

The harvested CSCs and MSCs were treated with 100 μM H_2_O_2_ for 2 h as previously described[47]. FMC was used to determine early apoptosis and necrosis in C-kit^**+**^ CSCs using an Annexin V-FITC/PI staining assay as reported elsewhere[5]. The phosphatidylserine levels on the surface of the C-kit^**+**^ CSCs were estimated using the Annexin V- FITC and Propidium Iodide (PI) apoptosis detection kit (Solarbio, China) according to the manufacturer’s instructions. Apoptosis was analyzed in the C-kit^**+**^ CSCs using a flow cytometer (BD Biosciences, USA). The results are expressed as the percentage of apoptotic cells among all cells. Flow cytometry was performed twice using C-kit^+^ CSCs in three independent experiments. CCK-8 was used to determine MSC viability in three independent experiments.

### 2.7. Cell transfection

Fifty nanomoles of miR-21 mimics,inhibitors or negative control were added to 1.5 ml F12 medium in 6-well plates with 5 μl Lipofectamine 2000 transfection reagent (Invitrogen, USA) and incubated with the C-kit^**+**^ CSCs or MSCs for 48 h according to the manufacturer's instructions. The efficiency of the mimics or inhibitors was confirmed by RT-qPCR.

### 2.8. RNA interference

The synthesized siR-PTEN (siR-PTEN) and scramble (GeneCopoeia, MD) were transfected into C-kit^+^ CSCs using a lentiviral construct (HANBIO, China) according to the manufacturer’s instructions. Briefly, The lentiviral vector expressing PTEN(siR-PTEN) or PTEN negative control (siR-PTEN-NC)were constructed by inserting the siR-PTEN gene or siR-PTEN-NC into a Lv-EGFP vector using BamHI (FD0054) and EcoRI (N41890) restriction sites, all obtained from Invitrogen (Thermo Fisher Scientific). The lentiviral particles were prepared using a calcium phosphate method.The C-kit^+^ CSCs (1× 10^5^ per well) were plated into 6-well plates and then treated with siR-PTEN and siR-PTEN-NC in the presence of 2 μg/ml polybrene (Sigma-Aldrich) at a multiplicity of infection of 50 MOI for 48 h. The siR-PTEN knockdown efficiency was confirmed by Western blotting and RT-qPCR.

### 2.9. Reverse transcription and Real-Time qPCR analysis of miR-21 and PTEN

The mRNA and miRNA levels were determined using quantitative RT-PCR as previously described[53, 54]. Briefly, the RNAs from the CSCs, MSCs and exosomes were isolated using the TRIzol (Invitrogen, USA) method. RT-PCR was performed on cDNA generated from 3 μg of the total RNA using a cDNA synthesis kit (TaKaRa, Japan) according to the manufacturer's protocol. RT-qPCR was performed using the CFX Connect Real-Time system (Bio-Rad, USA) and a SYBR green PrimScript RT kit (TaKaRa, Japan) according to the manufacturer's instructions. U6 and β-actin were used as the internal controls for the miR-21 and PTEN mRNA quantification, respectively.

### 2.10. Internalization of DiI-labeled exosomes into C-kit+ CSCs

The C-kit^+^ CSCs were harvested and seeded in fibronectin-coated dishes and maintained at 37°C overnight. Briefly, the MSC-Exos were labeled with 1 g/ml DiI (Invitrogen, USA) as previously described[18]. Then, the exosomes were washed with PBS and centrifuged at 100,000 g for 2 h to remove the unbound DiI. DiI-labeled exosomes were added to the culture medium of C-kit^+^ CSCs at a concentration of 10 Ug/ml for 24 h. Then, the C-kit^+^ CSCs were washed with PBS, fixed in 4% paraformaldehyde, and stained with 1 mg/ml 40,6-diamidino-2-phenylindole (DAPI) (Invitrogen, USA) for 10 min. Finally, the fluorescence was observed under a fluorescence microscope (Olympus).

### 2.11. Flow cytometry assay of apoptosis in C-kit^+^ CSCs

The C-kit^**+**^ CSCs were pre-incubated with different treatments (2 × 10^9^ particles per ml) and then incubated with 100 μM H_2_O_2_ for 2 h. Following treatment, the apoptosis rate was analyzed by flow cytometry using the Annexin V-FITC/PI kit (Solarbio, China) according to the manufacturer’s instructions. Flow cytometry was performed twice using C-kit^**+**^ CSCs in three independent experiments.

### 2.12. Terminal deoxynucleotidyl transferase dUTP nick end labeling (TUNEL) staining for detecting the apoptosis of C-kit^+^ CSCs

The percentage of apoptotic cells was also detected by using In-situ cell death detection kit (Sigma, USA) following the manufacturer‘s protocol. In brief, cells were planted on Petrl dish. After the cells were fixed with 4% paraformaldehyde and permeabilized by incubating with 1% Triton X-100.they were incubated in 50 μl/slide TUNEL reaction mixture (viaL1:viaL2 = 1:9) at 37°C with in darkness for 60 min under a humidified atmosphere.After incubation, the cells were stained with Hoechst33258 for 5 min. Apoptotic cells were counted in random fields by fluorescence microscopy; each experiment was performed in triplicate (×40 magnification, at least 6 fields per sample).

### 2.13. Western blotting

A Western blot analysis of the total protein from the C-kit^**+**^ cell lysates was performed as previously described[55]. The protein extracts were separated by SDS-polyacrylamide gel electrophoresis (SDS-PAGE) and transferred to PVDF membranes. After blocking overnight in a nonfat milk solution, the membranes were probed with the anti-PTEN, -phospho-Akt, -Akt, -caspase-3, -cleaved caspase-3, -β-actin or -GAPDH primary antibodies. The PVDF membranes were incubated with horseradish peroxidase-conjugated secondary antibodies for 1 h and then exposed to an enhanced chemiluminescence substrate (Amersham Biosciences, USA). The immunoreactivity was visualized using a ChemiDoc MP system (Bio-Rad, USA). The protein levels were normalized to β-actin or GAPDH.

### 2.14. Statistical analysis

All data were analyzed by performing Student’s t-tests or one-way ANOVAs, followed by LSD or Dunnett’s T3 post-hoc test for multiple comparisons. A P-value less than 0.05 was considered statistically significant. The data analyses were performed using SPSS software (v.19.0, IBM, USA). The data are presented as the mean ± SD.

## 3. Results

### 3.1. Isolated MSCs and C-kit^+^ CSCs

**Fig 1:**
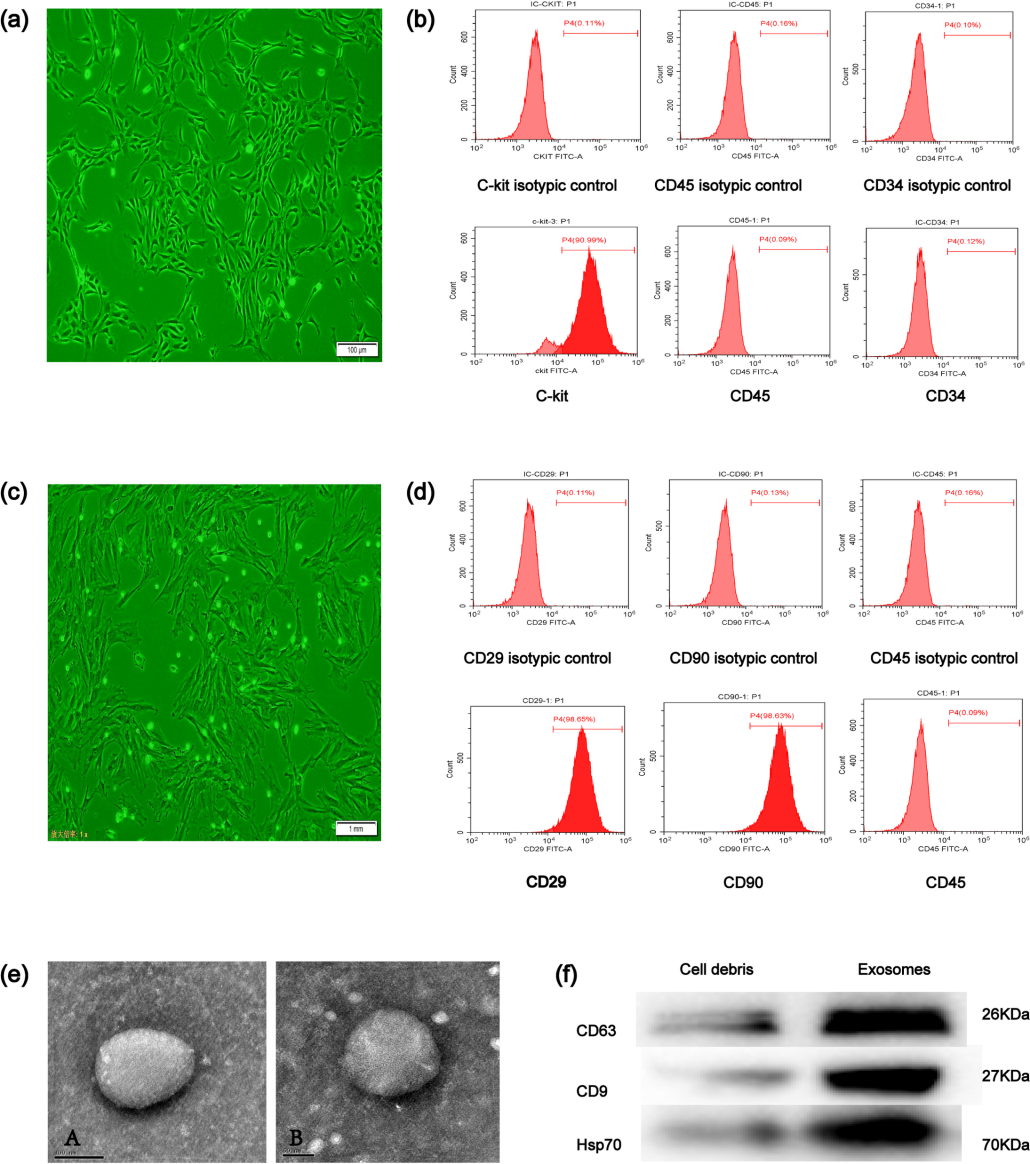
Characterization of C-kit^+^ CSCs, MSCs, and exosomes. (a) Phase morphology of C-kit^+^ CSCs (Olympus, Japan); scale bar = 100 μm. (b) Representative flow cytometric characterization of C-kit^+^ CSCs for the typical surface antigens and isotype control after magnetic bead sorting. surface expression of C-kit, and absence of surface expression of CD45, CD34. (c) MSC morphology was observed under a microscope (Olympus, Japan); scale bar = 100 μm. (d) MSCs were characterized by flow cytometric analysis for typical surface antigens or isotype control: surface expression of CD29, CD90,and absence of surface expression of CD45. (e) A transmission electron microscope was used to analyze MSC-derived exosomes. Images show a round-shaped vesicle with a diameter of approximately 100 nm. Scale bar = 100 nm/50 nm. (f) Western blotting characterization of the CD63, CD9, and Hsp70 MSC-Exos markers.

C-kit^**+**^ CSCs were purified using anti-rabbit secondary antibody-conjugated magnetic beads. The morphology of C-kit^+^ CSCs was triangular or polygonal (Fig 1(A)). According to flow cytometry analysis, 90.99% of the cells were positive for C-kit, 0.09% of the cells were positive for CD45, and 0.12% of the cells were positive for CD34 (Fig 1(B)). Primary MSCs isolated from the rats began adherent growth after 48 h of *in vitro* culture. Primary MSCs sub-cultured for 2–4 generations had a long spindle or polygonal appearance (Fig 1(C)). The following surface markers were identified on the MSCs by flow cytometry: (1) CD29 98.65%, (2) CD90 98.63%, and (3) CD45 0.09% (Fig 1(D)).

### 3.2. Exosomes secreted by MSCs were isolated and identified

MSC-Exos were obtained by precipitation. Then, the morphology of the exosomes was confirmed by performing transmission electron microscopy (TEM) and Western blotting as previously described[56] The exosomes had a round or oval-shaped appearance and were approximately 30–100 nm in size as directly observed by TEM(Fig 1(E)-A), and the size of exosome was not changed when MSCs are exposed to H_2_O_2_ (Fig 1(E)-B). The exosome surface markers CD63, CD9 and HSP70 could be detected in MSC-Exos by Western blotting (Fig 1(F)).

### 3.3. Oxidative stress induced apoptosis in the C-kit^+^ CSCs and altered the expression of miR-21 in MSCs, CSCs and exosomes

**Fig 2:**
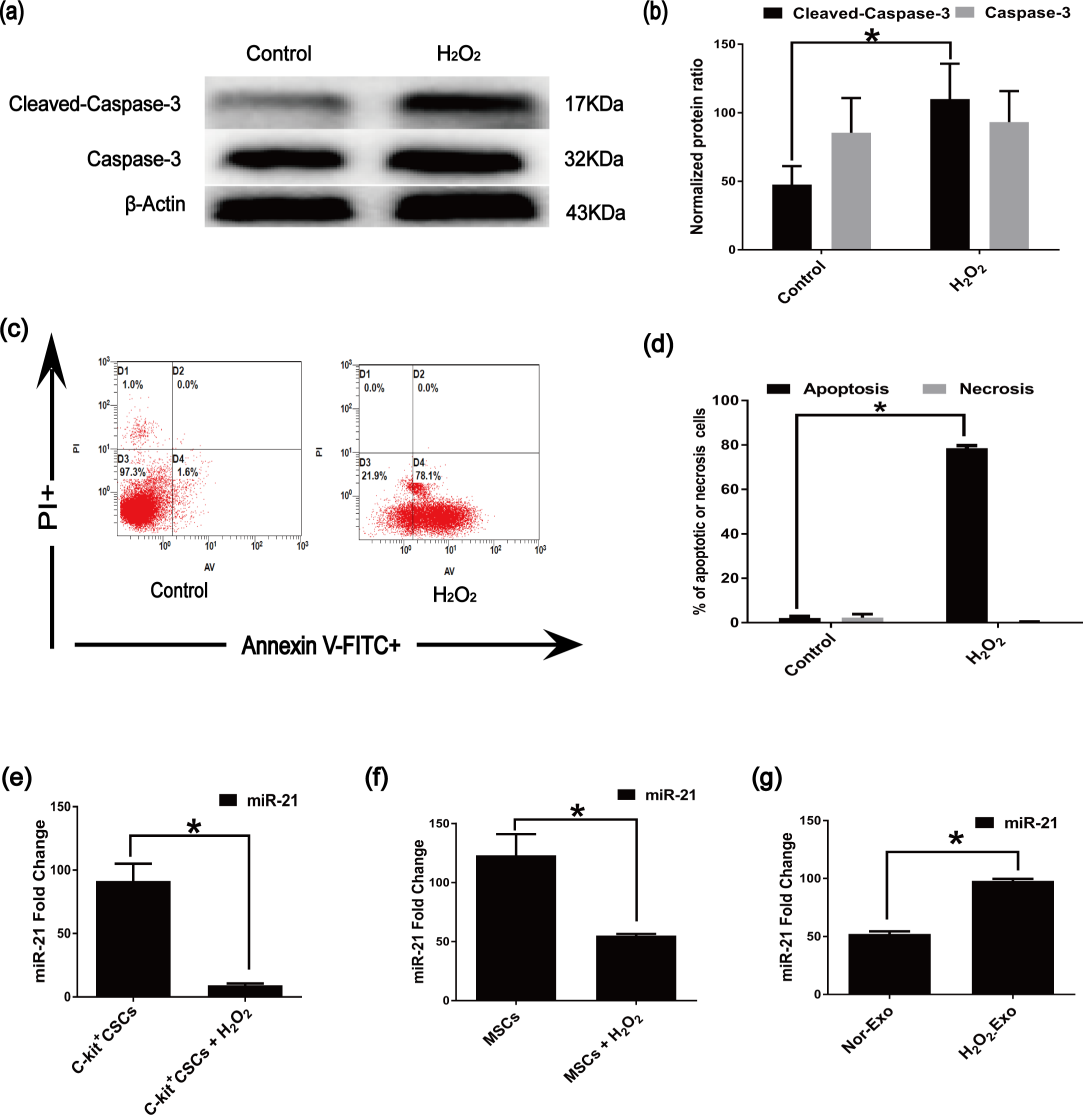
H_2_O_2_ affects C-kit^+^ CSC apoptosis and changes the expression of miR-21 in MSCs, C-kit^+^ CSCs and exosomes. (a)and(b) Western blotting analysis of caspase-3 and cleaved caspase-3 in C-kit^+^ CSCs cells after treatment with H_2_O_2_ (c) Rates of apoptosis in C-kit^+^ CSCs exposed to 100 μM H_2_O_2_ for 2 h measured by performing an Annexin V-FITC/PI staining assay.The upper left quadrant (%f Gated) shows the necrotic cells (Annexin V- /PI+), The upper right quadrant (% Gated) shows the late apoptotic cells(Annexin V+/PI+), The left lower quadrant (% Gated) shows the live cells (Annexin V- /PI-)and.The right lower quadrant (% Gated) shows the early apoptotic cells(Annexin V+/PI -), PI = propidium iodide.(d)The percentage of apoptotic cells was representing as both early and late apoptotic cells. H_2_O_2_ increased the percentage of apoptotic cells compared with Control groups. (e) Effects of H_2_O_2_ on miR-21 expression in C-kit^+^ CSCs. (f) Effects of H_2_O_2_ on miR-21 expression in MSCs (g) miR-21 expression levels in exosomes after exposure to H_2_O_2_ (n = 3, *P<0.05 versus control groups).

We established an *in vitro* model of C-kit^+^ CSC apoptosis by treating the cells with 100 μM H_2_O_2_ for 2 h. Western blotting was performed to detect the expression of the mitochondria-related pro-apoptotic protein cleaved caspase-3, which is the active form of caspase-3. Treatment with 100 μM H_2_O_2_ up-regulated the levels of cleaved caspase-3 in C-kit^+^ CSCs (Fig 2(A) and 2(B)). According to flow cytometry analysis, H_2_O_2_ challenge resulted in apoptosis increasing of C-kit^+^ CSCs in comparison to the control (Fig 2(C) and 2(D)). miR-21 levels were also examined in H_2_O_2_-treated C-kit^+^ CSC cells, and the result showed miR-21 levels were markedly reduced in C-kit^+^ CSCs following H_2_O_2_ treatment (Fig 2(E)), suggesting that miR-21 is likely negatively correlated with apoptosis in C-kit^+^ CSCs under oxidative stress conditions. In addition, compared with the controls, H_2_O_2_ treatment significantly reduced the expression of miR-21 in MSCs (Fig 2(F)). However, compared with the controls, the expression of miR-21 in MSC-Exos was up-regulated following H_2_O_2_-treatment (Fig 2(G)).

### 3.4. MSC-derived exosomes prevented H_2_O_2_-induced C-kit^+^ CSC apoptosis

**Fig 3:**
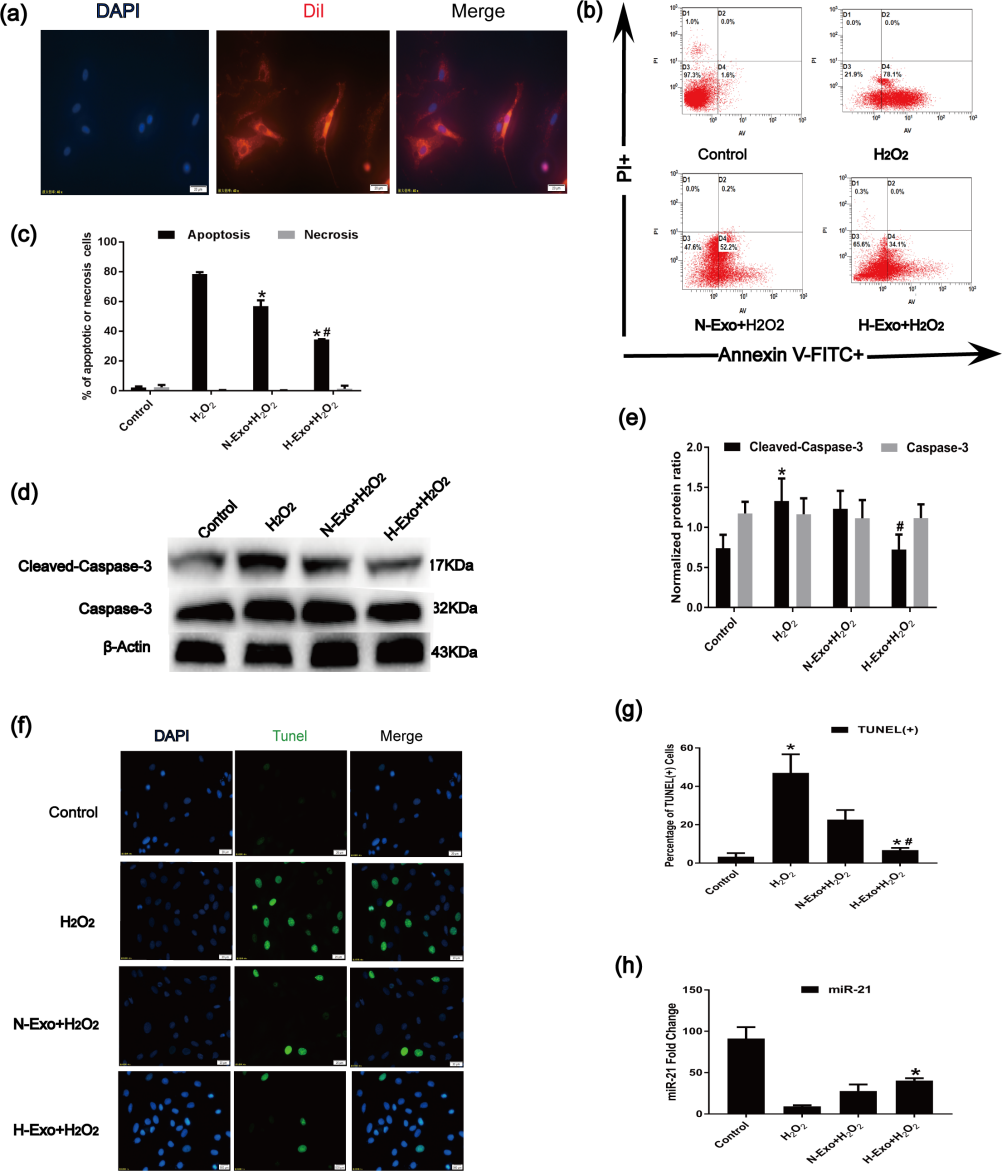
MSC-Exos inhibit H_2_O_2_-induced apoptosis in C-kit^+^ CSCs. (a) Cellular internalization of mesenchymal stem cell (MSC)-Exos into C-kit^+^ CSCs. DiI-labeled MSC-Exos (red) were internalized into DAPI-labeled CSCs (blue). Bar = 20 μm. (b) Apoptosis rates of C-kit^+^ CSCs were measured using the Annexin V-FITC/PI staining assay. The upper left quadrant (%f Gated) shows the necrotic cells (Annexin V- /PI+), The upper right quadrant (% Gated) shows the late apoptotic cells(Annexin V+/PI+), The left lower quadrant (% Gated) shows the live cells (Annexin V- /PI-)and.The right lower quadrant (% Gated) shows the early apoptotic cells(Annexin V+/PI -), PI = propidium iodide. (c)The percentage of apoptotic cells was representing as both early and late apoptotic cells. (n = 3, *P<0.05 versus the H_2_O_2_ group. ^#^P<0.05 versus the N-Exo group). (d)and(e) Immunoblotting was performed to detect caspase-3 and cleaved caspase-3 in C-kit^+^ CSCs (n = 3, *P<0.05 versus the control group. ^#^P<0.05 versus the H_2_O_2_ group). (f) Representative immunofluorescence staining of Hoechst33258 (blue), TUNEL (green) and merged images. Photos were taken randomly using fluorescence microscopy. Scale bar: 20 μm. (g) The panel shows the percentages of TUNEL positive cells. (n = 6, *P<0.05 versus the control group. ^#^P<0.05 versus the H_2_O_2_ group). (h) RT-qPCR analysis of miR-21 in C-kit^+^ CSCs treated with 100 μM H_2_O_2_ after pre-protection with N-Exos or H-Exos (n = 9, *P<0.05 versus the H_2_O_2_ group).

The unique biological function of exosomes is mainly to mediate cell-to-cell communication. The first step in the exchange of cargoes between cells is the internalization of exosomes by the target cells. To determine whether MSC-Exos can be internalized by C-kit^+^ CSCs, MSC-Exos were labeled with DiI. After incubation, labeled MSC-Exos (400 μg/ml) were combined with C-kit^+^ CSCs for 24 h and counterstained with DAPI to visualize the nuclei. Immunofluorescence staining showed strong red fluorescence in the cytoplasm and a blue nucleus in C-kit^+^ CSCs (Fig 3(A)), indicating that many MSC-Exos were internalized by C-kit^+^ CSCs. The anti-apoptotic effect of MSC-derived exosome was detected with Annexin V/PI assay. The Annexin V/PI assay showed that oxidative stress preconditioning MSC-derived exosome (H-Exo)decreased the percentage of the apoptotic cells compared with the normoxia preconditioning BMSC-derived exosome (N-exo) group and H2O2 group (Fig 3(B) and 3(C)). To examine whether BMSC-derived exosome protected against H_2_O_2_-Induced DNA fragmentation in C-kit^+^ CSCs, As shown in (Fig 3(F) and 3(G)), the percentages of TUNEL^+^ cells were significantly higher following treatment with H_2_O_2_ compared with control group, while H-Exo could significantly reduce the TUNEL^+^ cells ompared with H_2_O_2_ group or N-exo group.Expectedly, caspase-3 cleavage was suppressed (Fig 3(D) and 3(E)), and the decrease in miR-21 levels was significantly rescued (Fig 3(H)) in receptor cells under oxidative stress following pretreatment with exosomes derived from H_2_O_2_-treated MSCs. Therefore, H-Exo might exert a strong protective effect that helps C-kit^+^ CSCs resist apoptosis caused by oxidative stress. During this process, miR-21 likely plays an important role.

### 3.5. miR-21 in MSC-Exos participated in the protection of C-kit^+^ CSCs from apoptosis

**Fig 4:**
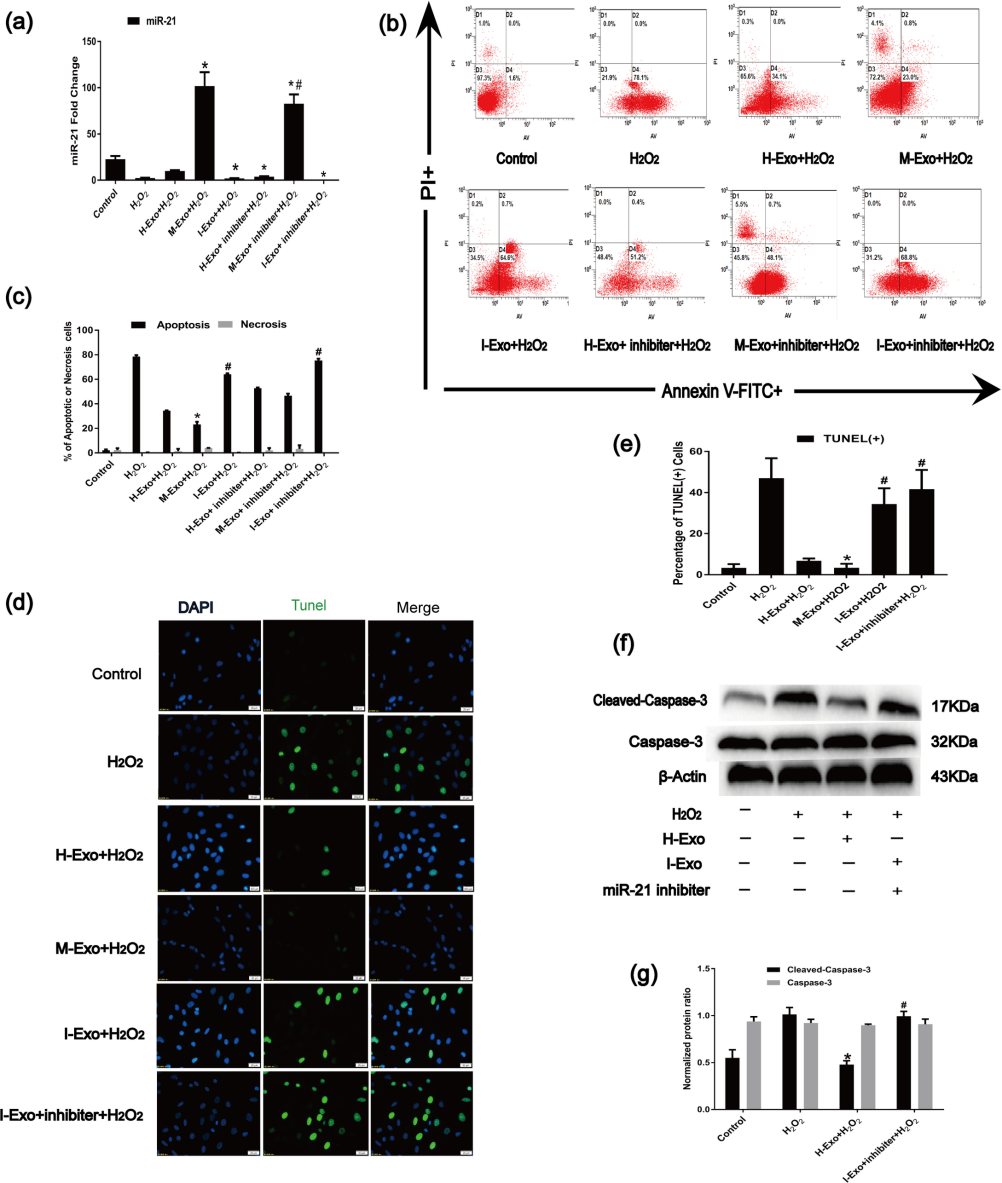
MSC-exosomal miR-21 inhibits apoptosis in C-kit^+^ CSCs. (a) RT-qPCR was performed to analyze miR-21 levels in C-kit^+^ CSCs (n = 9, *P<0.05 versus the H-Exo group. ^#^P<0.05 versus the M-Exo group). (b) Apoptosis rates of C-kit^+^ CSCs were measured using the Annexin V-FITC/PI staining assay. The upper left quadrant (%f Gated) shows the necrotic cells (Annexin V- /PI+), The upper right quadrant (% Gated) shows the late apoptotic cells(Annexin V+/PI+), The left lower quadrant (% Gated) shows the live cells (Annexin V- /PI-)and.The right lower quadrant (% Gated) shows the early apoptotic cells(Annexin V+/PI -), PI = propidium iodide.(c)The percentage of apoptotic cells was representing as both early and late apoptotic cells. (n = 3, *P<0.05 versus the H_2_O_2_ group. ^#^P<0.05 versus the H-Exo group). (f) Representative immunofluorescence staining of Hoechst33258 (blue), TUNEL (green) and merged images. Photos were taken randomly using fluorescence microscopy. Scale bar: 20 μm. (g) The panel shows the percentages of TUNEL positive cells. (n = 6, *P<0.05 versus the H_2_O_2_ group. ^#^P<0.05 versus the H-Exo group). (f)and(g) Western blot analysis of pro-apoptotic protein caspase-3 and cleaved caspase-3 in C-kit^+^ CSCs (n = 3, *P<0.05 versus the H_2_O_2_ group. ^#^P<0.05 versus the H-Exo group).

We investigated whether the effects of MSC-Exos on H_2_O_2_-induced apoptosis in C-kit+ CSCs were dependent on miR-21. MSCs were transfected with miR-21 mimics or inhibitors for 48 h. Then, MSC exosomes were harvested. The harvested exosomes were designated either Mimics-Exosome (M-Exo) or Inhibitor-Exosome (I-Exo). According to RT-qPCR analysis of miR-21, miR-21 significant increased and decreased following the pretreatment of H_2_O_2_-treated CSCs with M-Exo or I-Exo, respectively (Fig 4(A)). We also blocked miR-21 in C-kit^+^ CSCs using a miR-21 inhibitor for 48 h. Compared with that in the H-Exo or I-Exo groups, the miR-21 inhibitor treatment further decreased the expression of miR-21 after H_2_O_2_ insult (Fig 4(A)).

The anti-apoptotic effect of miR-21 in MSC-Exos was detected using the Annexin V-FITC/PI staining assay and the TUNEL measurement assays.M-Exos significantly decreased C-kit^+^ apoptosis after H_2_O_2_ insult, while I-Exos increased apoptosis in C-kit^+^ CSCs (Fig 4(B)–4(E)). When we simultaneously inhibited miR-21 in CSCs, we found that C-kit^+^ CSC apoptosis rates markedly increased under I-Exo+inhibitor conditions(Fig 4(B)–4(E)).Undoubtedly, the miR-21 inhibitor significantly increased the expression of pro-apoptotic protein-cleaved caspase-3, whereas H-Exos or M-Exo suppressed cleaved caspase-3 levels (Fig 4(F) and 4(G)) in C-kit^+^ CSCs under oxidative stress conditions. Thus, the miR-21 inhibitor could partially block the anti-apoptosis properties of exosomal miR-21, further indicating that rescuing the decreased miR-21 levels in C-kit^+^ CSCs by an H-Exo treatment might be a possible strategy to protect C-kit^+^ CSCs against oxidative stress-induced apoptosis.

### 3.6. Contribution of PTEN to the anti-apoptotic effects of miR-21 in C-kit^+^ CSCs

**Fig 5:**
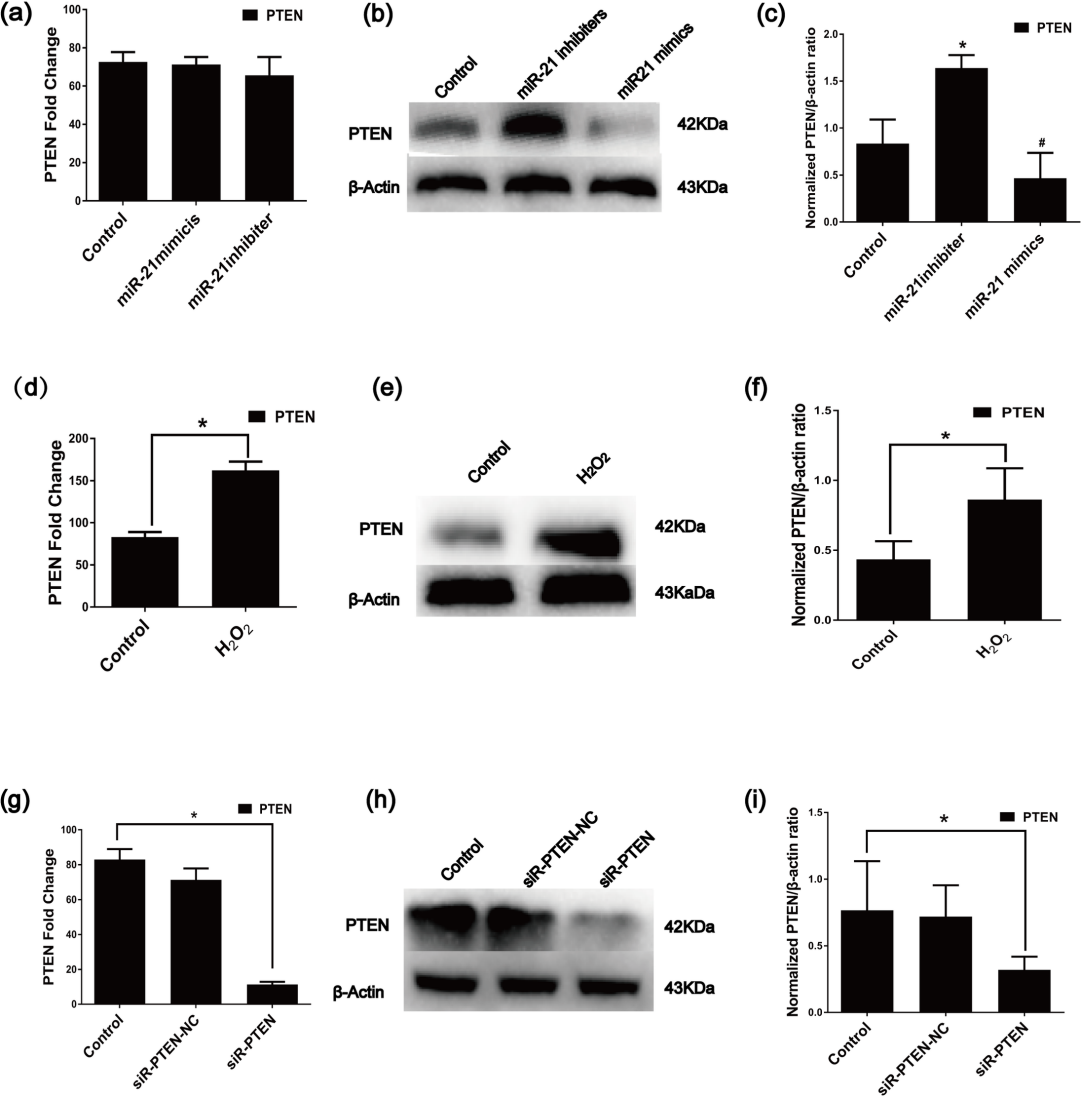
Effect of miR-21 on PTEN expression in CSCs. (a) PTEN mRNA did not significantly differ among groups by RT-qPCR. (b)and(c) the PTEN protein levels dramatically decreased after treatment with miR-21 mimics as demonstrated by Western blotting (n = 3, *P<0.05 versus control groups, ^#^P<0.05 versus inhibiter groups). (d) Difference in PTEN mRNA expression between the Control group and the H_2_O_2_ group confirmed by RT-qPCR. (n = 3, *P<0.05 versus control groups) (e)and(f) Western blot analysis of PTEN in C-kit^**+**^ CSCs treated with 100 μM H_2_O_2_ (n = 3, *P<0.05 versus the control group).(g) RT-qPCR analyzed PTEN in CSC after different conditions treated (n = 9, *P<0.05 versus control group).(h)and(i) Western blot analysis of PTEN protein levels after transfection of C-kit^+^ CSCs with siR-PTEN (n = 3, *P<0.05 versus control group).

**Fig 6:**
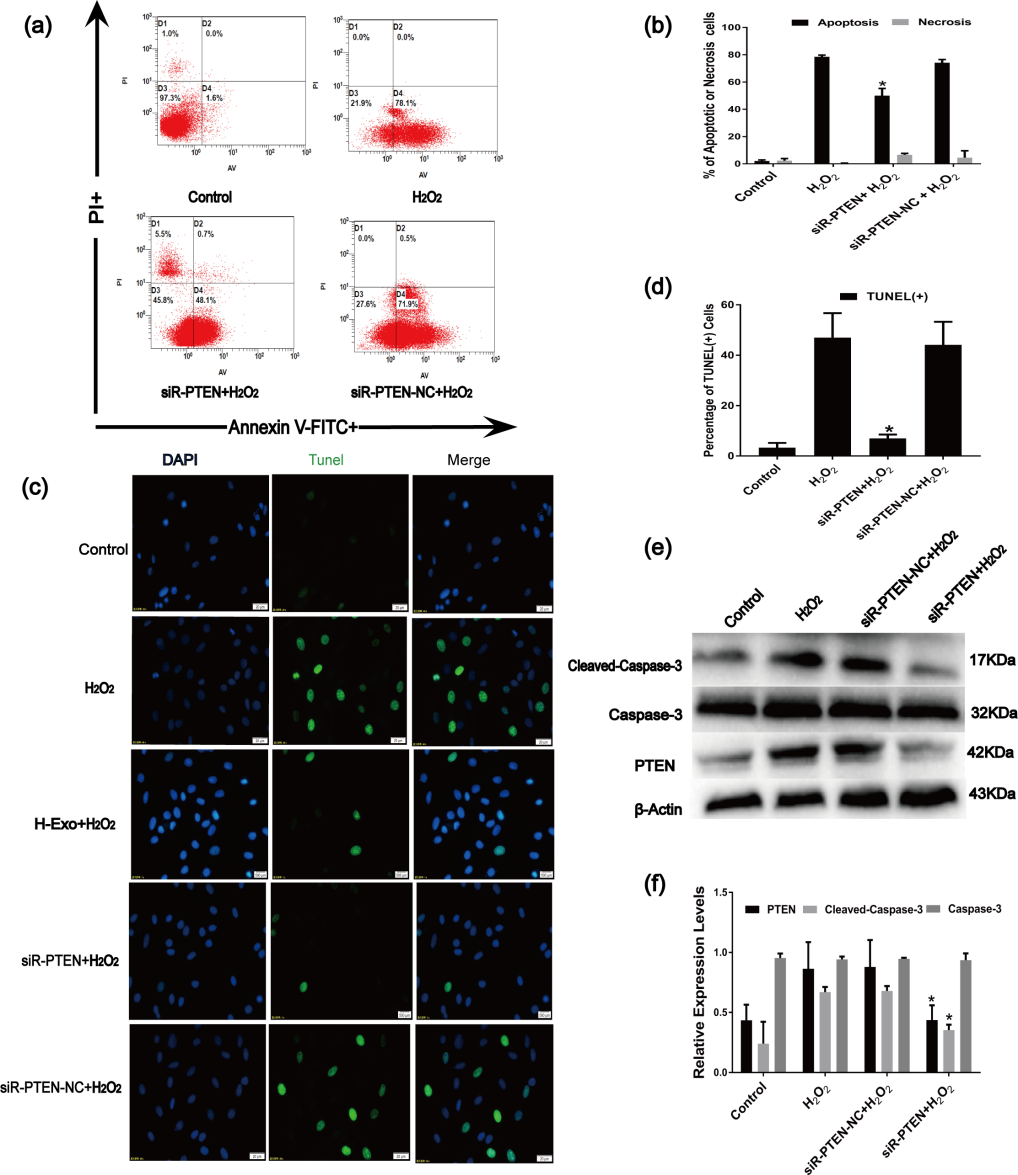
The anti-apoptotic contributions of PTEN in C-kit^+^ CSCs. (a) Apoptosis rates of C-kit^+^ CSCs were measured using the Annexin V-FITC/PI staining assay. The upper left quadrant (%f Gated) shows the necrotic cells (Annexin V- /PI+), The upper right quadrant (% Gated) shows the late apoptotic cells(Annexin V+/PI+), The left lower quadrant (% Gated) shows the live cells (Annexin V- /PI-)and.The right lower quadrant (% Gated) shows the early apoptotic cells(Annexin V+/PI -), PI = propidium iodide. (b)The percentage of apoptotic cells was representing as both early and late apoptotic cells. (n = 3, *P<0.05 versus the H_2_O_2_ group). (c) Representative immunofluorescence staining of Hoechst33258 (blue), TUNEL (green) and merged images. Photos were taken randomly using fluorescence microscopy. Scale bar: 20 μm. (d) The panel shows the percentages of TUNEL positive cells. (n = 6, *P<0.05 versus the H_2_O_2_ group).(e)and(f)Western blot analysis of caspase-3,cleaved caspase-3 and PTEN protein levels after transfection of C-kit^+^ CSCs with siR-PTEN. (n = 3, *P<0.05 versus the control group. ^#^P<0.05 versus the H_2_O_2_ groups).

Because PTEN has been shown to be a target gene of miR-21[39, 57, 58], we performed gain- and loss-of-function assays to verify the effects of miR-21 inhibitors and mimics on PTEN expression in C-kit^+^ CSCs. Compared with the control, the PTEN protein was significantly up-regulated in the inhibitor group and down-regulated in the mimic group, while the PTEN mRNA levels did not change (Fig 5(A)–5(C)) in the C-kit^+^ CSCs. Furthermore, according to the RT-qPCR and Western blot analyses, the mRNA and protein levels of PTEN were significantly up-regulated in C-kit^+^ CSCs after pretreatment with H_2_O_2_ (Fig 5(D)–5(F)). Therefore, miR-21 likely attenuates apoptosis by targeting PTEN. However, whether a relationship exists between PTEN and apoptosis in C-kit^+^ CSCs remains unknown. Thus, EGFP-labeled siRNA PTEN lentiviruses (siR-PTEN) and EGFP-labeled siRNA PTEN Negative Control vector (siR-PTEN-NC) were transfected into C-kit^+^ CSCs. The knockdown efficiency of siR-PTEN was detected by RT-qPCR and Western blotting, and the PTEN mRNA and protein levels were significantly down-regulated in siR-PTEN group(Fig 5(G)–5(I)). The percentage of apoptotic cells significantly decreased in the siR-PTEN group compared to the H_2_O_2_ group or the siR-PTEN-NC group (Fig 6(A)–6(D))as demonstrated by the Annexin V/PI assay and TUNEL measurement assays. Moreover, according to the Western blot analysis, cleaved caspase-3 levels were down-regulated in the siR-PTEN group compared with those in the H_2_O_2_ groups (Fig 6(E) and 6(F)). Altogether, the anti-apoptotic effects of miR-21 in C-kit^+^ CSCs were likely achieved via the inhibition of PTEN expression.

### 3.7. MSC-derived exosomes protected CSCs from H_2_O_2_-induced apoptosis via the PTEN/PI3K/Akt pathway

**Fig 7:**
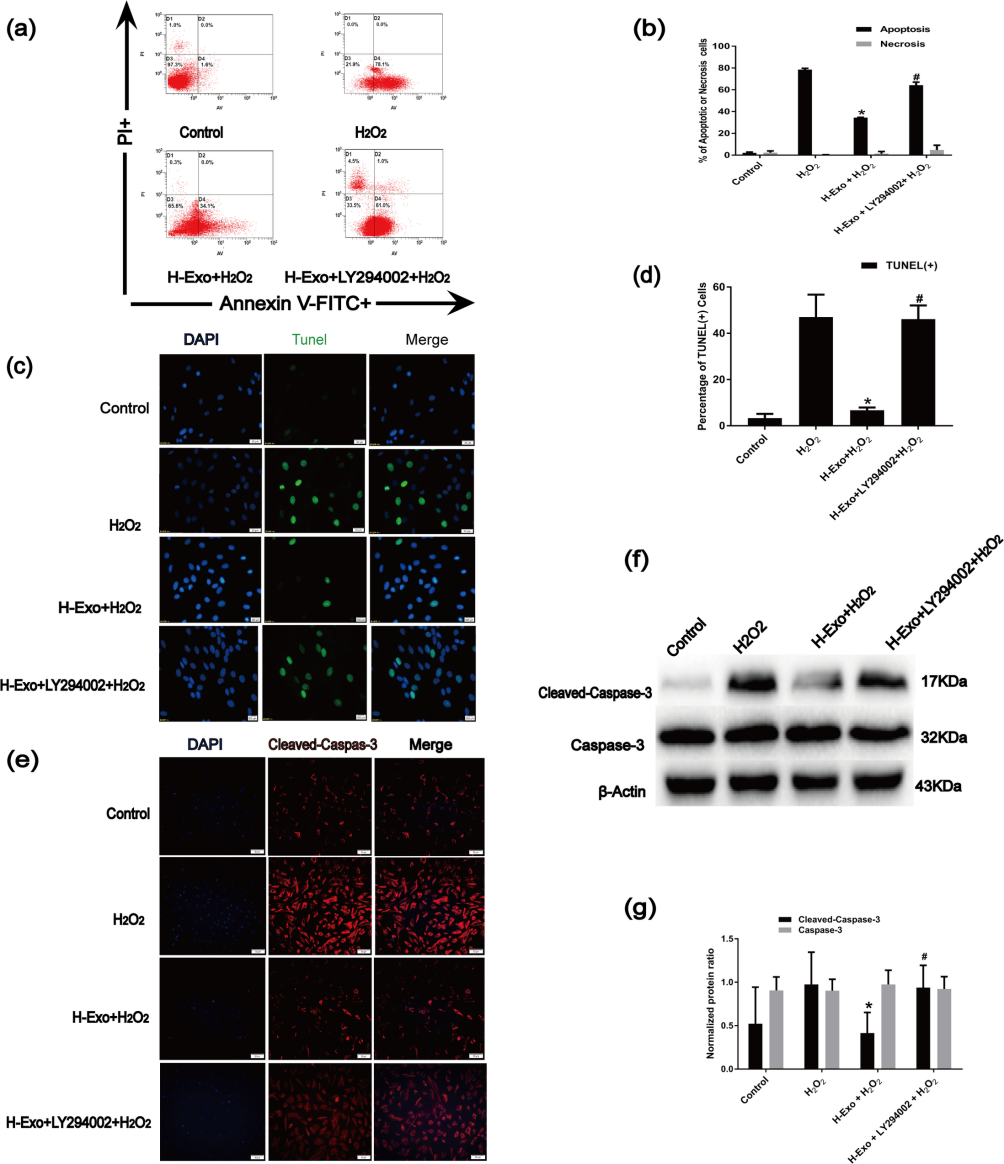
Contribution of the PTEN/PI3K/Akt axis to H_2_O_2_-induced apoptosis in C-kit^+^ CSCs. (a) Flow cytometry was performed to detect apoptosis using Annexin V-FITC/PI staining in C-kit^+^ CSCs that underwent different treatments. The first quadrant (%f Gated) shows the necrotic cells (Annexin V- /PI+), The second quadrant (% Gated) shows the late apoptotic cells(Annexin V+/PI+), The third quadrant (% Gated) shows the live cells (Annexin V- /PI-)and The fourth quadrant (% Gated) shows the early apoptotic cells(Annexin V+/PI -), PI = propidium iodide.(b)The percentage of apoptotic cells was representing as both early and late apoptotic cells. (n = 3, *P<0.05 versus the H_2_O_2_ group. ^#^ P<0.05 versus the H-Exo group). (c) Representative immunofluorescence staining of Hoechst33258 (blue), TUNEL (green) and merged images. Photos were taken randomly using fluorescence microscopy. Scale bar: 20 μm. (d) The panel shows the percentages of TUNEL positive cells. (n = 6, *P<0.05 versus the H_2_O_2_ group. ^#^ P<0.05 versus the H-Exo group). (e) Representative immunofluorescence staining of DAPI(blue), Cleaved-Caspase-3 (red) and merged images. Photos were taken randomly using fluorescence microscopy. Scale bar: 20 μm. (f-g)Apoptotic was further confirmed by immunoblotting for cleaved-caspase-3, (n = 3, *P<0.05 versus the H_2_O_2_ group. ^#^ P<0.05 versus the H-Exo group).

**Fig 8:**
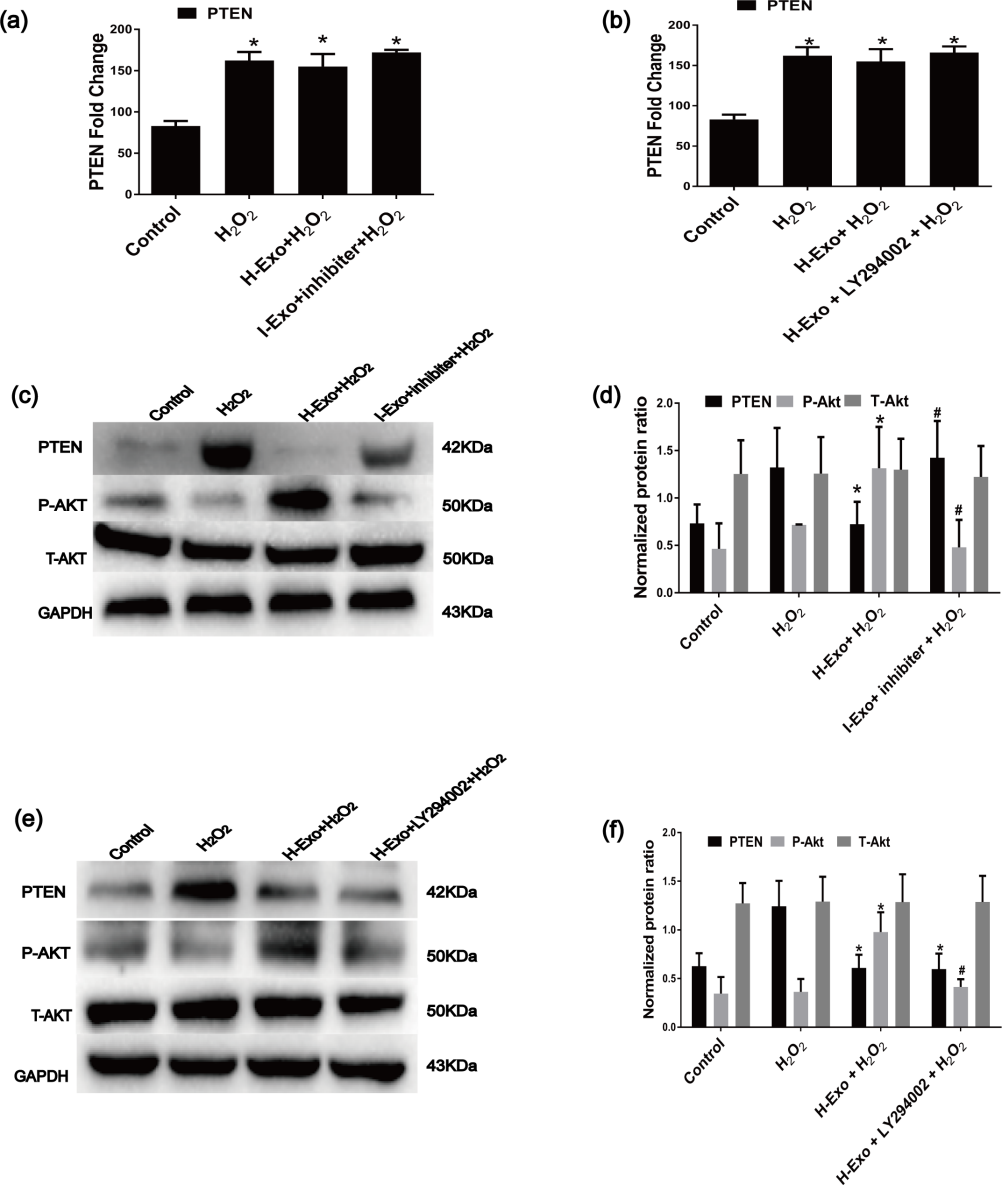
Contributions of the PTEN/PI3K/Akt pathway to the anti-apoptotic effects of MSC-derived exosomal miR-21. (a) RT-qPCR was performed to detect PTEN expression at the mRNA level in cells that underwent different treatments(n = 3, * P<0.05 versus the control group). (b) RT-qPCR was performed to detect PTEN expression at the mRNA level in cells that underwent different treatments(n = 3, * P<0.05 versus the control group)(c)and(d) Western blotting was performed to detect the effects of H-Exo on PTEN, P-Akt protein expression(n = 3, *P<0.05 versus the H_2_O_2_ group. ^#^P<0.05 versus the H-Exo group. p-Akt = phosphor-Akt. T-Akt = Total–Akt). (e)and(f) Western blotting was performed to detect the effects of H-exosomes on PTEN, P-Akt protein expression(n = 3, * P<0.05 versus the H_2_O_2_ group. ^#^P<0.05 versus the H-Exo group. p-Akt = phosphor-Akt. T-Akt = Total–Akt).

To identify the mechanisms responsible for the MSC-derived exosomal miR-21-mediated anti-apoptotic effects in C-kit^+^ CSCs, we blocked PI3K with the specific inhibitor LY294002. The Annexin V-FITC/PI staining assay and the TUNEL measurement assays were used to detecte the apoptotic cells. LY294002 partially reversed the anti-apoptotic effects of H-Exos (Fig 7(A)–7(D)).Molecularly, LY294002 reversed the H-Exo-induced effects on cleaved caspase-3 expression (Fig 7(E)), which was demonstrated by an increase in cleaved caspase-3 levels(Fig 7(F) and 7(G)). Furthermore, the miR-21 inhibitor could also reverse the anti-apoptotic effects of H-Exo. In this experiment, RT-qPCR showed that compared with the H_2_O_2_ group, H-Exo, miR-21 inhibiters or PI3K inhibitor LY294002 did not influnced PTEN expression levels (Fig 8(A) and 8(B)), PTEN protein was significantly down-regulated in the H-Exo group. Additionally, the exosomes incubation increased p-Akt levels(Fig 8(C)–8(F)),while the miR-21 inhibitor and PI3K inhibitor LY294002 dramatically decreased p-Akt levels (Fig 8(C)–8(F)).

## 4. Discussion

C-kit^+^ cardiac stem cells (C-kit^+^ CSCs)have emerged as some of the most promising CSCs for the prevention or treatment of myocardial remodeling and cardiac dysfunction after myocardial infarction[59]. However, after adoptive transfer, CSCs will encounter with various undesirable factors including oxidative stress, inflammation reactions and so on. all of which could decrease the cell viability and thereby compromise their therapeutic activities. [10]. Exosomes are intracellular messengers whose contents have been confirmed to be crucial signaling components for downstream reactions[20, 60]. Exosomes derived from MSCs could stimulate the proliferation, migration, and angiogenic potency of CSCs *in vitro* and *in vivo* and improve cardiac function[16]. However, very few studies have focused on the anti-apoptotic effects of MSC-Exos in C-kit^+^ CSCs under oxidative stress conditions. Additionally, the underlying molecular mechanisms by which exosomes protect C-kit^+^ CSCs must be elucidated. In this study, we obtained exosome vesicles (round, 30–100 nm) from conditioned MSC medium (Fig 1(E)) and confirmed the identity of these vesicles by detecting the expression of specific surface markers (Fig 1(F)).

Indeed, transplanted C-kit^+^ CSCs and pretreatment conditions must remain in an oxidative stress environment. Thus, establishing a similar pathological state in which to study the effects of exosomes in transplanted C-kit+ CSCs embedded in infarct zone or infarct border zones is imperative. miRNAs, which are shuttled by exosomes, are among the most important factors controlling gene expression. Additionally, exosomal miR-21 is up-regulated in many cell types under oxidative stress conditions[52, 61]. The exosome contents, however, greatly vary across different cell types and under different pathological conditions[22]. We also found that miR-21 reduces hydrogen peroxide-induced apoptosis and increases cell proliferationin c-kit cardiac stem cells in vitro through PTEN/PI3K/Akt signaling before[47, 62]. H_2_O_2_ has been widely used as an inducer of oxidative stress to mimic the pathophysiology of cardiovascular disease and cause cell apoptosis[28]. Therefore, we evaluated miR-21 expression in MSC-derived exosomes treated with the same concentration of H_2_O_2_. The 2h H_2_O_2_ treatment significantly induced C-kit^+^ CSC apoptosis (up to 78.1%). Consistently, H_2_O_2_ treatment induced the up-regulation of the pro-apoptosis protein cleaved caspase-3, which was associated with significantly reduced miR-21 expression levels (Fig 2(A)–2(E)). The negative correlation between apoptosis and miR-21 expression suggests that miR-21 plays an important role in the regulation of C-kit^+^ CSC apoptosis under oxidative stress conditions. Moreover, compared with the control group, H_2_O_2_ significantly reduced miR-21 expression in MSCs (Fig 2(F)). Interestingly, compared with that in the untreated group, the expression of miR-21 in the MSC-exosomes was up-regulated after H_2_O_2_-treatment (Fig 2(G)).

The harsh ischemic microenvironment in acute MI, which kills most injected cells, is a primary barrier limiting the effectiveness of stem cell transplantation. Preconditioning C-kit^+^ CSCs with MSC-Exos may serve as a promising therapeutic approach because the useful cellular components encapsulated in MSC-Exos may greatly improve the survival rate of C-kit^+^ CSCs in ischemic environments. In this study, exosomes released from MSCs were internalized by C-kit^+^ CSCs (Fig 3(A)), and the levels of miR-21 were significantly increased in C-kit^+^ CSCs pre-treated with H-Exos and N-Exos (Fig 3(H)) prior to oxidative stress exposure. Moreover, the exosomes derived from H_2_O_2_-treated MSCs were more effective at increasing miR-21 levels in receptor cells and decreasing C-kit^**+**^ CSC apoptosis and cleaved caspase-3 (Fig 3(B)–3(G)). To further confirm the anti-apoptotic effects of miR-21 from MSC-Exos after H_2_O_2_ treatment, we blocked miR-21 in C-kit^+^ CSCs and/or MSCs using a miR-21 inhibitor. Consistently, miR-21 inhibition significantly down-regulated miR-21 levels and partially reversed the anti-apoptotic effects of H-Exos (Fig 4(A)–4(G)). In conclusion, our datas indicated an intricate exosome-mediated crosstalk interface between the MSCs and the CSCs that regulates the oxidative damage program, at least partly, via miR-21.

miRNA transfer between cells can activate the recipient cells to produce a series of biological effects by inhibiting miRNA target genes. PDCD4, PTEN, RECK and Bcl-2 can be regulated by miR-21 in many cell types. These genes are critical for promoting cell proliferation, differentiation and migration[63–65]. PTEN has been reported to be a target gene of miR-21 in many cell types[3, 39]. To further confirm that PTEN is a target of miR-21 in C-kit^+^ CSCs, gain- and loss-of-function studies were performed, and miR-21 inhibitors increased while miR-21 mimics decreased PTEN protein levels in C-kit^+^ CSCs; however, PTEN mRNA levels did not change (Fig 5(A)–5(C)). We further tested whether PTEN is involved in the regulation of H_2_O_2_-induced apoptosis in C-kit^+^ CSCs and showed that the PTEN expression is significantly up-regulated in C-kit^+^ CSCs following the H_2_O_2_ treatment (Fig 5(D)–5(F)). Furthermore, using siRNA-mediated gene silencing, the siR-EPTEN vector efficiently infected the C-kit^+^ CSCs, and the PTEN mRNA/protein levels were efficiently inhibited(Fig 5(G)–5(I)).The inactivation of PTEN significantly decreased the rate of cell apoptosis (Fig 6(A)–6(F)). Altogether, miR-21 mediates cell protection by regulating PTEN.

miR-21 affects the PI3K/Akt pathway by targeting the PTEN gene[39]. The activation of Akt can protect cells from apoptosis induced by H_2_O_2_[66, 67]. To study whether the PTEN/PI3K/Akt signaling is responsible for exosomal miR-21 mediated anti-apoptotic effect, we blocked PI3K with LY294002 and assessed Akt phosphorylation. LY294002 significantly reversed the anti-apoptotic effects of H-Exos (Fig 7(A)–7(D)), which were associated with increased levels of cleaved caspase-3 (Fig 7(E)–7(G)). Additionally, the miR-21 inhibitor could also block the anti-apoptotic effects of MSC-Exos. H-Exos decreased PTEN levels and increased p-Akt levels, while miR-21 inhibitors dramatically decreased p-Akt levels(Fig 8(A)–8(F)). This finding not only suggests that Akt is downstream of PI3K and PTEN but also indicates that the cellular protection provided by H-Exos likely occurs via miR-21 and its regulation of the PTEN /PI3K/Akt signaling pathway.

## Conclusion

Exosomes carrying miR-21 can be effectively internalized into C-kit^+^ CSCs to protect these cells against apoptosis under stress conditions. This cargo successfully reduced PTEN expression, increased p-Akt levels, and exerted anti-apoptotic effects in C-kit^+^ CSCs. This effect can be compromised by miR-21 inhibitors and LY294002. Therefore, MSC-exosomes, particularly H-Exos, can rescue C-kit^+^ CSC apoptosis by regulating the miR-21/PTEN/PI3K/AKT axis under oxidative stress conditions. Although our data revealed that exosomal miR-21 derived from H_2_O_2_-induced MSCs plays a critical role in apoptosis regulation in recipient cells through the PTEN/PI3K/Akt pathway, we did not explore the function of other exosomal cargo. *In vivo* studies are warranted to further confirm that MSC-exosomes and changes in the PTEN/PI3K/Akt pathway have similar effects on the survival of C-kit^**+**^ CSCs.

## Supporting information

S1 File Apoptotic rates of CSCs detected by Annexin V-FITC PI dual staining assay. (ZIP)

S2 File Identification of MSC, exosomes and CSCs. (ZIP)

S3 File Internalization of DiI-labeled exosomes into CSCs. (ZIP)

S4 File qRT-PCR analyzed miR-21 and PTEN mRNA. (ZIP)

S5 File Apoptotic related genes detected by western blotting. (ZIP)

S6 File TUNEL staining detected the apoptosis of CSCs. (ZIP)

S7 File TUNEL staining detected the apoptosis of CSCs. (ZIP)

## References

[1] Beltrami AP, Barlucchi L, Torella D, Baker M, Limana F, Chimenti S, et al Adult Cardiac Stem Cells Are Multipotent and Support Myocardial Regeneration. cell. 2003:763–769. 14505575 10.1016/s0092-8674(03)00687-1

[2] Hong KU, Bolli R. Cardiac Stem Cell Therapy for Cardiac Repair. Current Treatment Options in Cardiovascular Medicine.2014;16(7): 24–336. doi: 10.1007/s11936-014-0324-3 . 24903489 10.1007/s11936-014-0324-3 PMC4055849

[3] Bolli R, Tang XL, Sanganalmath SK, Rimoldi O, Mosna F, Abdel-Latif A, et al Intracoronary delivery of autologous cardiac stem cells improves cardiac function in a porcine model of chronic ischemiccardiomyopathy.Circulation.2013;128(2):122–131. doi: 10.1161/CIRCULATIONAHA.112.001075 . 23757309 10.1161/CIRCULATIONAHA.112.001075 PMC3807652

[4] Mayfield AE, Tilokee EL, Davis DR. Resident cardiac stem cells and their role in stem cell therapies for myocardial repair. Can J Cardiol. 2014; 30 (11): 1288–1298. doi: 10.1016/j.cjca.2014.03.018 . 25092406 10.1016/j.cjca.2014.03.018

[5] Tang XL, Li Q, Rokosh G, Sanganalmath SK, Chen N, Ou Q, et al Long-Term Outcome of Administration of c-kit(POS) Cardiac Progenitor Cells After Acute Myocardial Infarction: Transplanted Cells Do not Become Cardiomyocytes, but Structural and Functional Improvement and Proliferation of Endogenous Cells Persist for at Least One Year. Circulation research. 2016;118(7):1091–1105. doi: 10.1161/CIRCRESAHA.115.307647 . 26838790 10.1161/CIRCRESAHA.115.307647 PMC4818175

[6] Taghavi S, Sharp TE, Duran JM, Makarewich CA, Berretta RM, Starosta T, et al Autologous c-Kit+ Mesenchymal Stem Cell Injections Provide Superior Therapeutic Benefit as Compared to c-Kit+ Cardiac-Derived Stem Cells in a Feline Model of Isoproterenol-Induced Cardiomyopathy. Clinical and translational science. 2015;8(5):425–431. doi: 10.1111/cts.12251 . 25684108 10.1111/cts.12251 PMC5351102

[7] Cho JA, Lee YS, Kim SH, Ko JK, Kim CW. MHC independent anti-tumor immune responsesinduced by Hsp70-enriched exosomes generate tumor regression in murine models. Cancer Lett. 2009;75(2):256–265. doi: 10.1016/j.canlet.2008.10.021 19036499 10.1016/j.canlet.2008.10.021

[8] Hu S, Huang M, Nguyen PK, Gong Y, Li Z, Jia F, et.al,Novel microRNA prosurvival cocktail for improving engraftment and function of cardiac progenitor cell transplantation. Circulation. 2011;124(11):27–34. https://doi.org/10.1161/circulationaha.111.017954/-/dc1. 10.1161/CIRCULATIONAHA.111.017954 PMC3181082 21911815

[9] Van Kranenburg M, Magro M, Thiele H, de Waha S, Eitel I, Cochet A, et al Prognostic value of microvascular obstruction and infarct size, as measured by CMR in STEMI patients. JACC Cardiovascular imaging. 2014;7(9):930–939. doi: 10.1016/j.jcmg.2014.05.010 . 25212799 10.1016/j.jcmg.2014.05.010

[10] Hong KU, Li QH, Guo Y, Patton NS, Moktar A, Bhatnagar A, et al A highly sensitive and accurate method to quantify absolute numbers of c-kit+ cardiac stem cells following transplantation in mice. Basic research in cardiology. 2013;108(3):346 doi: 10.1007/s00395-013-0346-0 . 23549981 10.1007/s00395-013-0346-0 PMC3684056

[11] Laflamme MA, Chen KY, Naumova AV, Muskheli V, Fugate JA, Dupras SK, et al Cardiomyocytes derived from human embryonic stem cells in pro-survival factors enhance function of infarcted rat hearts. Nature biotechnology. 2007;25(9):1015–1024. doi: 10.1038/nbt1327 . 17721512 10.1038/nbt1327

[12] Cai C, Guo Y, Teng L, Nong Y, Tan M, Book MJ, et al Preconditioning Human Cardiac Stem Cells with an HO-1 Inducer Exerts Beneficial Effects After Cell Transplantation in the Infarcted Murine Heart. Stem cells. 2015;33(12):3596–3607. doi: 10.1002/stem.2198 . 26299779 10.1002/stem.2198 PMC4766973

[13] Mohsin S, Khan M, Toko H, Bailey B, Cottage CT, Wallach K, et al Human cardiac progenitor cells engineered with Pim-I kinase enhance myocardial repair. Journal of the American College of Cardiology. 2012;60(14):1278–1287. doi: 10.1016/j.jacc.2012.04.047 . 22841153 10.1016/j.jacc.2012.04.047 PMC3461098

[14] Teng L, Bennett E, Cai C. Preconditioning c-Kit-positive Human Cardiac Stem Cells with a Nitric Oxide Donor Enhances Cell Survival through Activation of Survival Signaling Pathways. The Journal of biological chemistry. 2016;291(18):9733–9747. doi: 10.1074/jbc.M115.687806 . 26940876 10.1074/jbc.M115.687806 PMC4850310

[15] Rustom A, Saffrich R, Markovic I, Walther P, Gerdes HH. Nanotubular highways for intercellular organelle transport. Science. 2004;303(5660):1007–10. doi: 10.1126/science.1093133 . 14963329 10.1126/science.1093133

[16] Zhang Z, Yang J, Yan W, Li Y, Shen Z, Asahara T. Pretreatment of Cardiac Stem Cells With Exosomes Derived From Mesenchymal Stem Cells Enhances Myocardial Repair. Journal of the American Heart Association. 2016;5(1):e002856 doi: 10.1161/JAHA.115.002856 26811168 10.1161/JAHA.115.002856 PMC4859399

[17] Lee C, Mitsialis SA, Aslam M, Vitali SH, Vergadi E, Konstantinou G, et al Exosomes mediate the cytoprotective action of mesenchymal stromal cells on hypoxia-induced pulmonary hypertension. Circulation.2012;126(22):2601–11. doi: 10.1161/CIRCULATIONAHA.112.114173 . 23114789 10.1161/CIRCULATIONAHA.112.114173 PMC3979353

[18] Bian S, Zhang L, Duan L, Wang X, Min Y, Yu H. Extracellular vesicles derived from human bone marrow mesenchymal stem cells promote angiogenesis in a rat myocardial infarction model. J Mol Med (Berl). 2014;92(4):387–397. doi: 10.1007/s00109-013-1110-5 . 24337504 10.1007/s00109-013-1110-5

[19] S ELA, Mager I, Breakefield XO, Wood MJ. Extracellular vesicles: biology and emerging therapeutic opportunities.Nature reviews Drug discovery. 2013, 12 (5):347–357. doi: 10.1038/nrd3978. . 23584393 10.1038/nrd3978

[20] Cervio E, Barile L, Moccetti T, Vassalli G. Exosomes for Intramyocardial Intercellular Communication. Stem cells international. 2015;2015:482171 doi: 10.1155/2015/482171 . 26089917 10.1155/2015/482171 PMC4454760

[21] Hulsmans M, Holvoet P. MicroRNA-containing microvesicles regulating inflammation in association with atherosclerotic disease. Cardiovascular research. 2013;100(1):7–18. doi: 10.1093/cvr/cvt161 . 23774505 10.1093/cvr/cvt161

[22] Sluijter JP, Verhage V, Deddens JC, van den Akker F, Doevendans PA. Microvesicles and exosomes for intracardiac communication. Cardiovascular research. 2014;102(2):302–311. doi: 10.1093/cvr/cvu022 . 24488559 10.1093/cvr/cvu022

[23] Lai RC, Arslan F, Lee MM, Sze NS, Choo A, Chen TS, et al Exosome secreted by MSC reduces myocardial ischemia/reperfusion injury. Stem cell research. 2010;4(3):214–222. doi: 10.1016/j.scr.2009.12.003 . 20138817 10.1016/j.scr.2009.12.003

[24] Hatzistergos KE, Quevedo H, Oskouei BN, Hu Q, Feigenbaum GS, Margitich IS, et al Bone marrow mesenchymal stem cells stimulate cardiac stem cell proliferation and differentiation. Circulation research. 2010;107(7):913–922. doi: 10.1161/CIRCRESAHA.110.222703 . 20671238 10.1161/CIRCRESAHA.110.222703 PMC3408082

[25] Boon RA, Dimmeler S. MicroRNAs in myocardial infarction. Nature reviews Cardiology. 2015;12(3):135–142. doi: 10.1038/nrcardio.2014.207 . 25511085 10.1038/nrcardio.2014.207

[26] Small EM, Frost RJ, Olson EN. MicroRNAs add a new dimension to cardiovascular disease.Circulation.2010;121(8):1022–1032. doi: 10.1161/CIRCULATIONAHA.109.889048 . 20194875 10.1161/CIRCULATIONAHA.109.889048 PMC2847432

[27] Lv G, Shao S, Dong H, Bian X, Yang X, Dong S. MicroRNA-214 protects cardiac myocytes against H2O2-induced injury. Journal of cellular biochemistry. 2014;115(1):93–101. doi: 10.1002/jcb.24636 . 23904244 10.1002/jcb.24636

[28] Lv C, Hao Y, Han Y, Zhang W, Cong L, Shi Y, et al Role and mechanism of microRNA-21 in H2O2-induced apoptosis in bone marrow mesenchymal stem cells. Journal of clinical neuroscience: official journal of the Neurosurgical Society of Australasia. 2016;27:154–160. doi: 10.1016/j.jocn.2015.07.029 . 26810470 10.1016/j.jocn.2015.07.029

[29] Wei C, Li L, Kim IK, Sun P, Gupta S. NF-kappaB mediated miR-21 regulation in cardiomyocytes apoptosis under oxidative stress. Free radical research. 2014;48(3):282–291. doi: 10.3109/10715762.2013.865839 . 24237305 10.3109/10715762.2013.865839

[30] Wu T, Liu Y, Fan Z, Xu J, Jin L, Gao Z, et al miR-21 Modulates the Immunoregulatory Function of Bone Marrow Mesenchymal Stem Cells Through the PTEN/Akt/TGF-β.Stem Cells. 2015 11;33(11):3281–3890. doi: 10.1002/stem.2081 26086742 10.1002/stem.2081

[31] Richart A, Loyer X, Neri T, Howangyin K, Guerin CL, Ngkelo A, et al MicroRNA-21 coordinates human multipotent cardiovascular progenitors therapeutic potential. Stem Cells.2014;32(11):2908–22. doi: 10.1002/stem.1789 . 25069679 10.1002/stem.1789

[32] Ciuffreda L, Falcone I, Incani UC, Del Curatolo A, Conciatori F, Matteoni S, et al PTEN expression and function in adult cancer stem cells and prospects for therapeutic targeting. Advances in biological regulation. 2014; 56:66–80. doi: 10.1016/j.jbior.2014.07.002 . 25088603 10.1016/j.jbior.2014.07.002

[33] Panigrahi A, Pinder S, Chan S, Paish E, Robertson J, Ellis I. The role of PTEN and its signalling pathways, including AKT, in breast cancer; an assessment of relationships with other prognostic factors and with outcome. The Journal of pathology.2004;204(1)93–100. doi: 10.1002/path.1611 . 15307142 10.1002/path.1611

[34] Mocanu M, Yellon D. PTEN, the Achilles' heel of myocardial ischaemia/reperfusion injury? British journal of pharmacology. 2007;150(7):833–838 doi: 10.1038/sj.bjp.0707155 17293884 10.1038/sj.bjp.0707155 PMC2013879

[35] Schmid AC, Byrne RD, Vilar R, Woscholski R. Bisperoxovanadium compounds are potent PTEN inhibitors.FEBS letters. 2004; 566(1–3):35–38. doi: 10.1016/j.febslet.2004.03.102 15147864 10.1016/j.febslet.2004.03.102

[36] Schwartzbauer G, Robbins J. The tumor suppressor gene PTEN can regulate cardiac hypertrophy and survival. Journal of Biological Chemistry.2001;276(38):35786–35793. doi: 10.1074/jbc.M102479200 11448956 10.1074/jbc.M102479200

[37] Wu D-N, Pei D-S, Wang Q, Zhang G-Y. Down-regulation of PTEN by sodium orthovanadate inhibits ASK1 activation via PI3-K/Akt during cerebral ischemia in rat hippocampus. Neuroscienceletters.2006;404(1):98–102 doi: 10.1016/j.neulet.2006.05.018 16762504 10.1016/j.neulet.2006.05.018

[38] Stambolic V, Suzuki A, De La Pompa JL, Brothers GM, Mirtsos C, Sasaki T, et al Negative regulation of PKB/Akt-dependent cell survival by the tumor suppressor PTEN. Cell. 1998;95(1):29–39. 9778245 10.1016/s0092-8674(00)81780-8

[39] Qi W, Li H, Cai XH, Gu JQ, Meng J, Xie HQ, et al Lipoxin A4 activates alveolar epithelial sodium channel gamma via the microRNA-21/PTEN/AKT pathway in lipopolysaccharide-induced inflammatory lung injury. Laboratory investigation; 2015;95(11):1258–68. doi: 10.1038/labinvest.2015.109 . 26302186 10.1038/labinvest.2015.109

[40] Li J, Yen C, Liaw D, Podsypanina K, Bose S, Wang SI, et al PTEN, a putative protein tyrosine phosphatase gene mutated in human brain, breast, and prostate cancer. Science. 1997;275(5308):1943–7. 9072974 10.1126/science.275.5308.1943

[41] Meng F, Henson R, Lang M, Wehbe H, Maheshwari S, Mendell JT, et al Involvement of human micro-RNA in growth and response to chemotherapy in human cholangiocarcinoma cell lines. Gastroenterology.2006;130(7):2113–21. doi: 10.1053/j.gastro.2006.02.057 16762633 10.1053/j.gastro.2006.02.057

[42] Di Cristofano A, Pandolfi PP. The multiple roles of PTEN in tumor suppression. Cell. 2000;100(4):387–90. 10693755 10.1016/s0092-8674(00)80674-1

[43] Bai H, Xu R, Cao Z, Wei D, Wang C. Involvement of miR-21 in resistance to daunorubicin by regulating PTEN expression in the leukaemia K562 cell line. FEBS letters. 2011;585(2):402–8. doi: 10.1016/j.febslet.2010.12.027 21187093 10.1016/j.febslet.2010.12.027

[44] Yan-nan B, Zhao-yan Y, Li-xi L, Qing-jie X, Yong Z. MicroRNA-21 accelerates hepatocyte proliferation in vitro via PI3K/Akt signaling by targeting PTEN. Biochemical and biophysical research communications.2014;443(3):802–7. doi: 10.1016/j.bbrc.2013.12.047 24342610 10.1016/j.bbrc.2013.12.047

[45] Hesheng Ou YL, Min Kang. Activation of miR-21 by STAT3 Induces Proliferation and Suppresses Apoptosis in Nasopharyngeal Carcinoma by Targeting PTEN Gene. PLOS ONE. 2014;9(11):e109929 doi: 10.1371/journal.pone.0109929 25365510 10.1371/journal.pone.0109929 PMC4217720

[46] Deng W, Wang Y, Long X, Zhao R, Wang Z, Liu Z, et al miR-21 Reduces Hydrogen Peroxide-Induced Apoptosis in c-kit+Cardiac Stem Cells In Vitro through PTEN/PI3K/Akt Signaling. Oxidative Medicine and Cellular Longevity. 2016;2016:1–14. doi: 10.1155/2016/5389181 27803763 10.1155/2016/5389181 PMC5075640

[47] Shi B, Deng W, Long X, Zhao R, Wang Y, Chen W, et al miR-21 increases c-kit+ cardiac stem cell proliferation in vitro through PTEN/PI3K/Akt signaling. PeerJ. 2017;5:e2859 doi: 10.7717/peerj.2859 . 28168101 10.7717/peerj.2859 PMC5289448

[48] Sanganalmath SK, Bolli R. Cell therapy for heart failure: a comprehensive overview of experimental and clinical studies, current challenges, and future directions. Circulation research. 2013;113(6):810–834. doi: 10.1161/CIRCRESAHA.113.300219 . 23989721 10.1161/CIRCRESAHA.113.300219 PMC3892665

[49] Shi B, Long X, Zhao R, Liu Z, Wang D, Xu G. Transplantation of mesenchymal stem cells carrying the human receptor activity-modifying protein 1 gene improves cardiac function and inhibits neointimal proliferation in the carotid angioplasty and myocardial infarction rabbit model. Experimental biology and medicine. 2014;239(3):356–365. doi: 10.1177/1535370213517619 . 24477823 10.1177/1535370213517619

[50] Hu Guo-wen, Li Qing, Niu Xin, Hu Bin, Juan Liu1, Shu-min Zhou,et al Exosomes secreted by human-induced pluripotent stem cell-derived mesenchymal stem cells attenuate limb ischemia by promoting angiogenesis in mice. stem Cell Research and therapy. 2015;6(11):1–15. doi: 10.1186/scrt546 26268554 10.1186/scrt546 PMC4533800

[51] Safinur Ataya CG-T, Mehmet Kesimerc, Douglas D. Taylora b.Morphologic and proteomic characterization of exosomes released by cultured extravillous trophoblast cells. EXPERIMENTAL CELL RESEARCH 2011;317(2011):1192–1202. doi: 10.1016/j.yexcr.2011.01.014 21276792 10.1016/j.yexcr.2011.01.014

[52] Xiao J, Pan Y, Li XH, Yang XY, Feng YL, Tan HH, et al Cardiac progenitor cell-derived exosomes prevent cardiomyocytes apoptosis through exosomal miR-21 by targeting PDCD4. Cell death & disease. 2016;7(6):e2277 doi: 10.1038/cddis.2016.181 . 27336721 10.1038/cddis.2016.181 PMC5143405

[53] Bobis-Wozowicz S, Kmiotek K, Sekula M, Kedracka-Krok S, Kamycka E, Adamiak M, et al Human Induced Pluripotent Stem Cell-Derived Microvesicles Transmit RNAs and Proteins to Recipient Mature Heart Cells Modulating Cell Fate and Behavior. Stem Cells. 2015;33(9):2748–2761. doi: 10.1002/stem.2078 . 26031404 10.1002/stem.2078

[54] Fisher SA, Doree C, Mathur A, Martin-Rendon E. Meta-analysis of cell therapy trials for patients with heart failure. Circulation research. 2015;116(8):1361–1377. doi: 10.1161/CIRCRESAHA.116.304386 . 25632038 10.1161/CIRCRESAHA.116.304386

[55] Writing Group M, Mozaffarian D, Benjamin EJ, Go AS, Arnett DK, Blaha MJ, et al Heart Disease and Stroke Statistics-2016 Update: A Report From the American Heart Association. Circulation. 2016;133(4):e38–360. doi: 10.1161/CIR.0000000000000350 . 26673558 10.1161/CIR.0000000000000350

[56] Yu B, Kim HW, Gong M, Wang J, Millard RW, Wang Y, et al Exosomes secreted from GATA-4 overexpressing mesenchymal stem cells serve as a reservoir of anti-apoptotic microRNAs for cardioprotection. Int J Cardiol.2015;182:349–360. doi: 10.1016/j.ijcard.2014.12.043 . 25590961 10.1016/j.ijcard.2014.12.043 PMC4382384

[57] Zhou X, Ren Y, Liu A, Han L, Zhang K, Li S, et al STAT3 inhibitor WP1066 attenuates miRNA-21 to suppress human oral squamous cell carcinoma growth in vitro and in vivo. Oncology reports. 2014;31(5):2173–2180. doi: 10.3892/or.2014.3114 . 24676554 10.3892/or.2014.3114

[58] Ou H, Li Y, Kang M. Activation of miR21 by STAT3 induces proliferation and suppresses apoptosis in nasopharyngeal carcinoma by targeting PTEN gene. PloS one. 2014;9(11):e109929 doi: 10.1371/journal.pone.0109929 25365510 10.1371/journal.pone.0109929 PMC4217720

[59] Ellison GM, Vicinanza C, Smith AJ, Aquila I, Leone A, Waring CD, et al Adult c-kit(pos) cardiac stem cells are necessary and sufficient for functional cardiac regeneration and repair. Cell. 2013;154(4):827–842. doi: 10.1016/j.cell.2013.07.039 . 23953114 10.1016/j.cell.2013.07.039

[60] El Andaloussi S, Mäger I, Breakefield XO, Wood MJA. Extracellular vesicles: biology and emerging therapeutic opportunities. Nature Reviews Drug Discovery. 2013;12(5):347–357. doi: 10.1038/nrd3978 . 23584393 10.1038/nrd3978

[61] Fang S, Xu C, Zhang Y, Xue C, Yang C, Bi H, et al Umbilical Cord-Derived Mesenchymal Stem Cell-Derived Exosomal MicroRNAs Suppress Myofibroblast Differentiation by Inhibiting the Transforming Growth Factor-beta/SMAD2 Pathway During Wound Healing. Stem cells translational medicine. 2016 5(10):1425–1439. doi: 10.5966/sctm.2015-0367 . 27388239 10.5966/sctm.2015-0367 PMC5031180

[62] Deng W, Wang Y, Long X, Zhao R, Wang Z, Liu Z, et al miR-21 Reduces Hydrogen Peroxide-Induced Apoptosis in c-kit+ Cardiac Stem Cells In Vitro through PTEN/PI3K/Akt Signaling. Oxid Med Cell Longev. 2016;12(24):1–14. doi: 10.1155/2016/5389181 . 27803763 10.1155/2016/5389181 PMC5075640

[63] Liwak-Muir U DC, Naing T, Wylie Q, Chehade L, Baird SD, Chakraborty PK, Holcik M. ERK8 is a novel HuR kinase that regulates tumour suppressor PDCD4 through a miR-21 dependent mechanism.Oncotarget.2015; 32(9):1439–1450. doi: 10.18632/oncotarget.6363 26595526 10.18632/oncotarget.6363 PMC4811471

[64] Xu LF, Wu ZP, Chen Y, Zhu QS, Hamidi S, Navab R. MicroRNA-21 (miR-21) regulates cellular proliferation, invasion, migration, and apoptosis by targeting PTEN, RECK and Bcl-2 in lung squamous carcinoma,. PloS one. 2014;9(8):e103698 doi: 10.1371/journal.pone.0103698 . 25084400 10.1371/journal.pone.0103698 PMC4118890

[65] Chan JK, Blansit K, Kiet T, Sherman A, Wong G, Earle C, et al The inhibition of miR-21 promotes apoptosis and chemosensitivity in ovarian cancer. Gynecol Oncol. 2014;132(3):739–744. doi: 10.1016/j.ygyno.2014.01.034 . 24472409 10.1016/j.ygyno.2014.01.034

[66] Yang P, Peairs JJ, Tano R, Jaffe GJ. Oxidant-mediated Akt activation in human RPE cells. Investigative Ophthalmology & Visual Science. 2006;47(10):4598–4606. doi: 10.1167/iovs.06-0140 17003457 10.1167/iovs.06-0140

[67] Suk Ho B, Sung Chul L, Soo Hyun C, Hyung-Keun L, Lee JH, Young Kwang C, et al Vascular endothelial growth factor as an autocrine survival factor for retinal pigment epithelial cells under oxidative stress via the VEGF-R2/PI3K/Akt. Investigative Ophthalmology & Visual Science. 2010;51(2):1190–1197. doi: 10.1167/iovs.09-4144 19834034 10.1167/iovs.09-4144

