# Supplementary material for: Bone marrow mesenchymal stem cell-derived exosomal miR-21 protects C-kit+ cardiac stem cells from oxidative injury through the PTEN/PI3K/Akt axis
Source: PLoS One. 2018 Feb 14;13(2):e0191616. doi: 10.1371/journal.pone.0191616 (PMC5812567; doi:10.1371/journal.pone.0191616)

2016-6-13-WY-CSC-DW 1-8 00006837 772.LMD : Gallios

Institution:
Protocol :2015-1-28-WY-CSC-DW A+P 00013198 2015-01-28 968.PRO
Listmode Replay: New Protocol
Analysis Date: 13-Apr-2017, 14:36:12
Settings File: 2015-1-28-WANGYAN-CSC-DW.PRO, 13-Jun-2016, 14:53:36
Listmode File: 2016-6-13-WY-CSC-DW 1-8 00006837 772.LMD

Run Date: 13-Jun-16, 15:16:03
Sample ID: 2016-6-13-WY-CSC-DW
User ID: user
Acquisition Time/Events: 208.6s / 52346 (MANUAL)

Instrument SN: AS34195 Software Version: Gallios 1.2

[Ungated] SS INT LIN/FS INT LIN

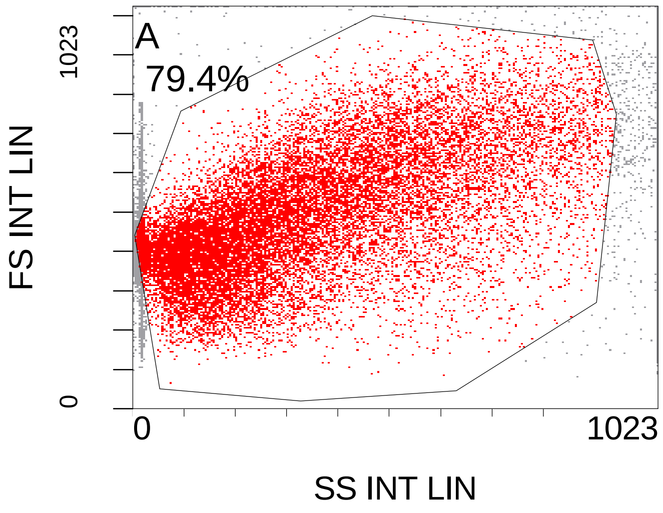


[A] FL1 INT LOG

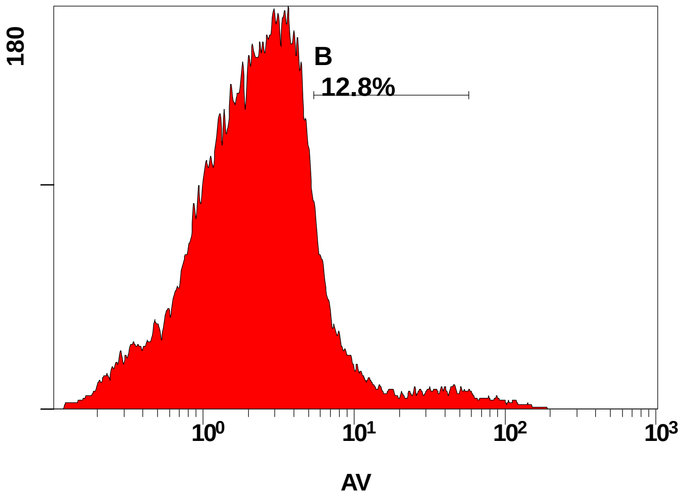


[A] FL3 INT LOG

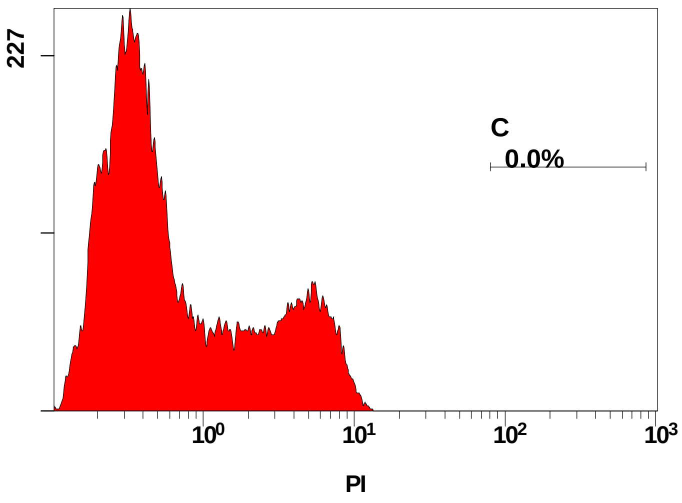


[A] FL1 INT LOG/FL3 INT LOG

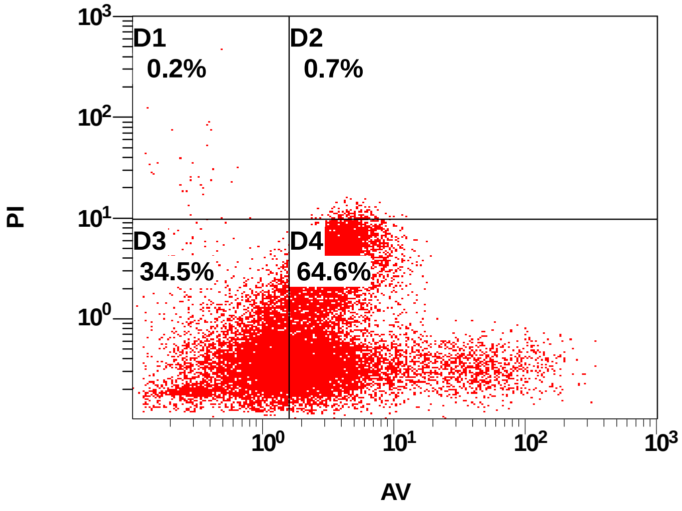

Supplement: S1 File — (ZIP) [file pone.0191616.s001.zip › Original data underlying the findings described in manuscript-Apoptosis rates of CSCs were detected by Annexin V-FITC PI staining assay/I-Exo group/3 Annexin V-FITC PI staining assay.PDF]
