# Supplementary material for: Bone marrow mesenchymal stem cell-derived exosomal miR-21 protects C-kit+ cardiac stem cells from oxidative injury through the PTEN/PI3K/Akt axis
Source: PLoS One. 2018 Feb 14;13(2):e0191616. doi: 10.1371/journal.pone.0191616 (PMC5812567; doi:10.1371/journal.pone.0191616)

2015-6-4-WY-CSC-DW 4-2 00000484 485.LMD : Gallios

Institution:
Protocol :2015-1-28-WY-CSC-DW A+P 00013198 2015-01-28 968.PRO
Listmode Replay: New Protocol
Analysis Date: 13-Apr-2017, 14:35:39
Settings File: 2015-1-28-WANGYAN-CSC-DW.PRO, 01-Jun-2015, 15:03:05
Listmode File: 2015-6-4-WY-CSC-DW 4-2 00000484 485.LMD

Run Date: 04-Jun-15, 14:33:03
Sample ID: 2015-6-4-WY-CSC-DW
User ID: user
Acquisition Time/Events: 95.2s / 42176 (MANUAL)

Instrument SN: AS34195 Software Version: Gallios 1.2

[Ungated] SS INT LIN/FS INT LIN

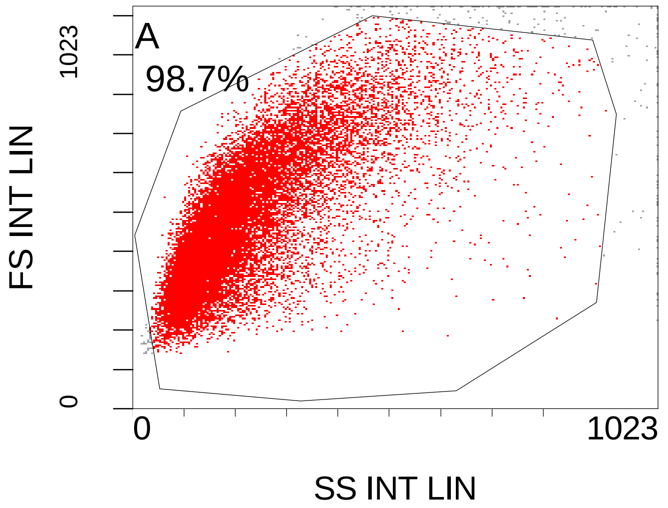

[A] FL1 INT LOG

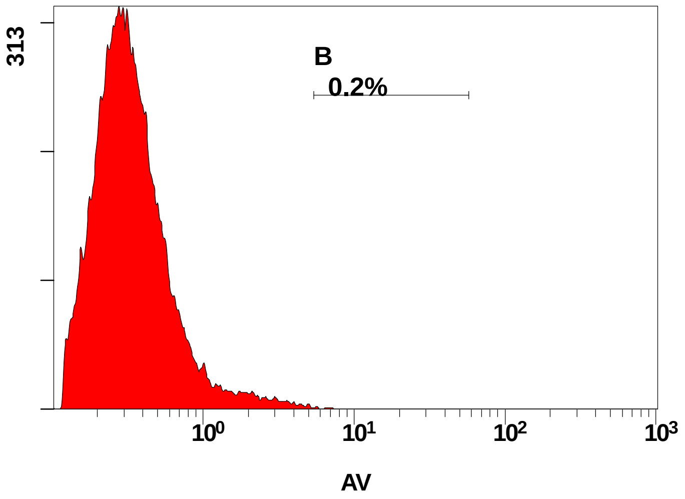

[A] FL3 INT LOG

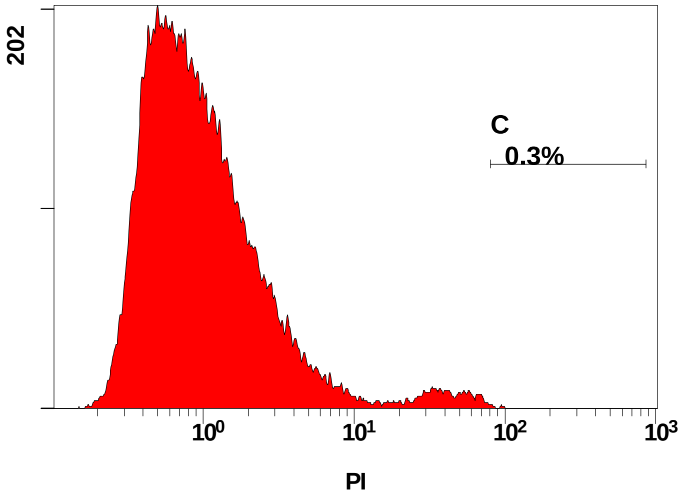

[A] FL1 INT LOG/FL3 INT LOG

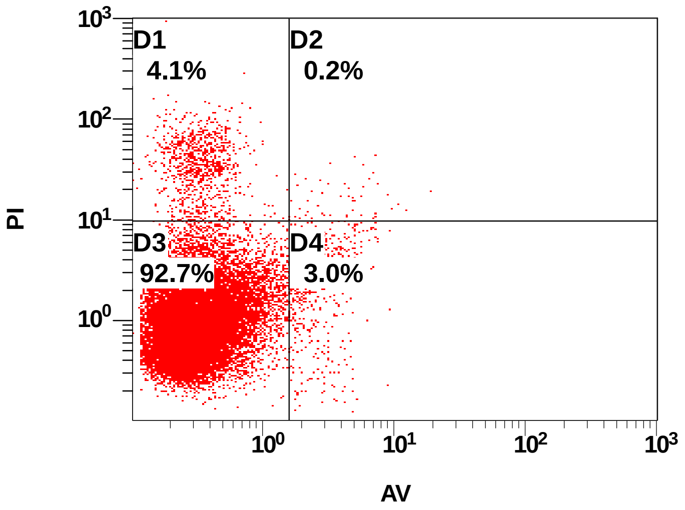

Supplement: S1 File — (ZIP) [file pone.0191616.s001.zip › Original data underlying the findings described in manuscript-Apoptosis rates of CSCs were detected by Annexin V-FITC PI staining assay/ZC group/1 Annexin V-FITC PI staining assay.PDF]
