# Supplementary figures and images for: Bone marrow mesenchymal stem cell-derived exosomal miR-21 protects C-kit+ cardiac stem cells from oxidative injury through the PTEN/PI3K/Akt axis (part 1 of 4)
Source: PLoS One. 2018 Feb 14;13(2):e0191616. doi: 10.1371/journal.pone.0191616 (PMC5812567; doi:10.1371/journal.pone.0191616)

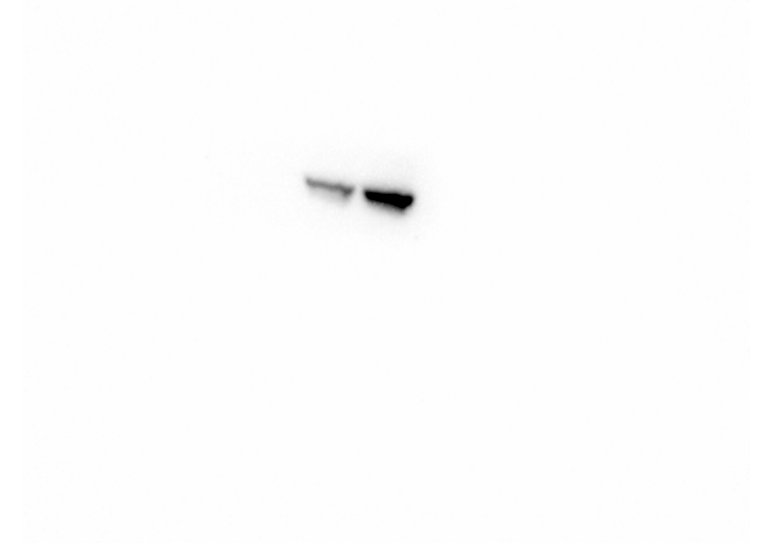

Supplement: S2 File — (ZIP) [file pone.0191616.s002.zip › Original data underlying the findings described in manuscript-Identification of MSC ,Exosomes and CSCs/ALIX.tif]

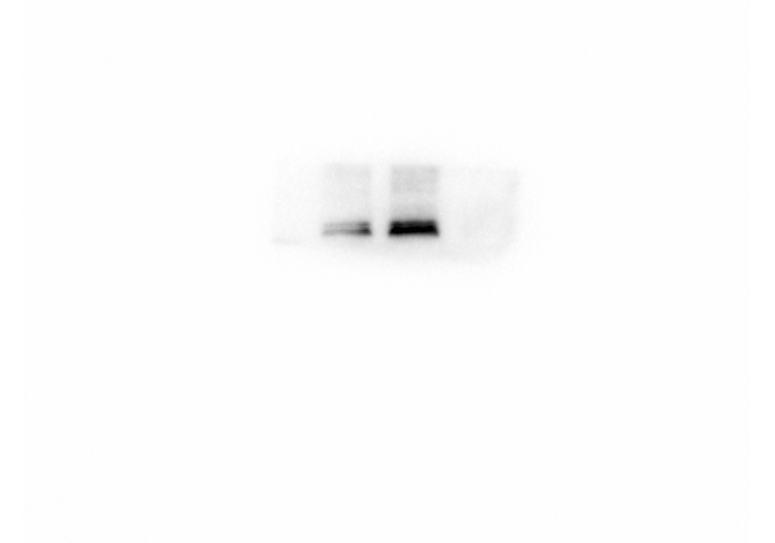

Supplement: S2 File — (ZIP) [file pone.0191616.s002.zip › Original data underlying the findings described in manuscript-Identification of MSC ,Exosomes and CSCs/CD63.tif]

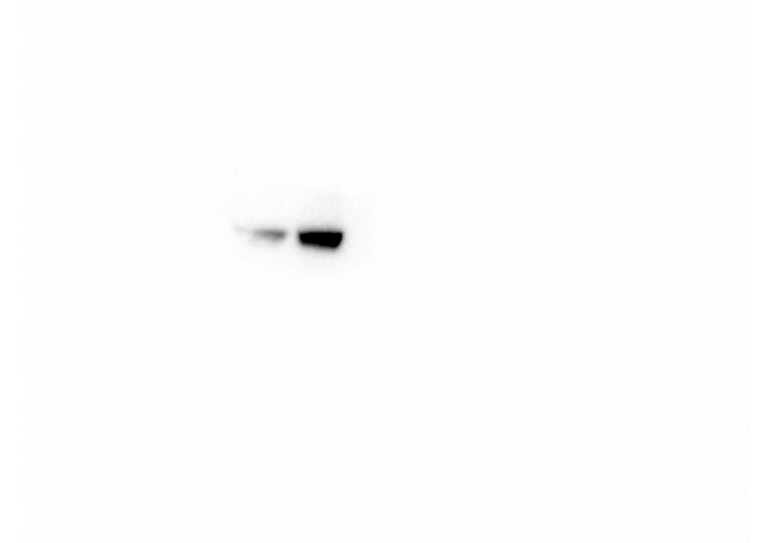

Supplement: S2 File — (ZIP) [file pone.0191616.s002.zip › Original data underlying the findings described in manuscript-Identification of MSC ,Exosomes and CSCs/CD9.tif]

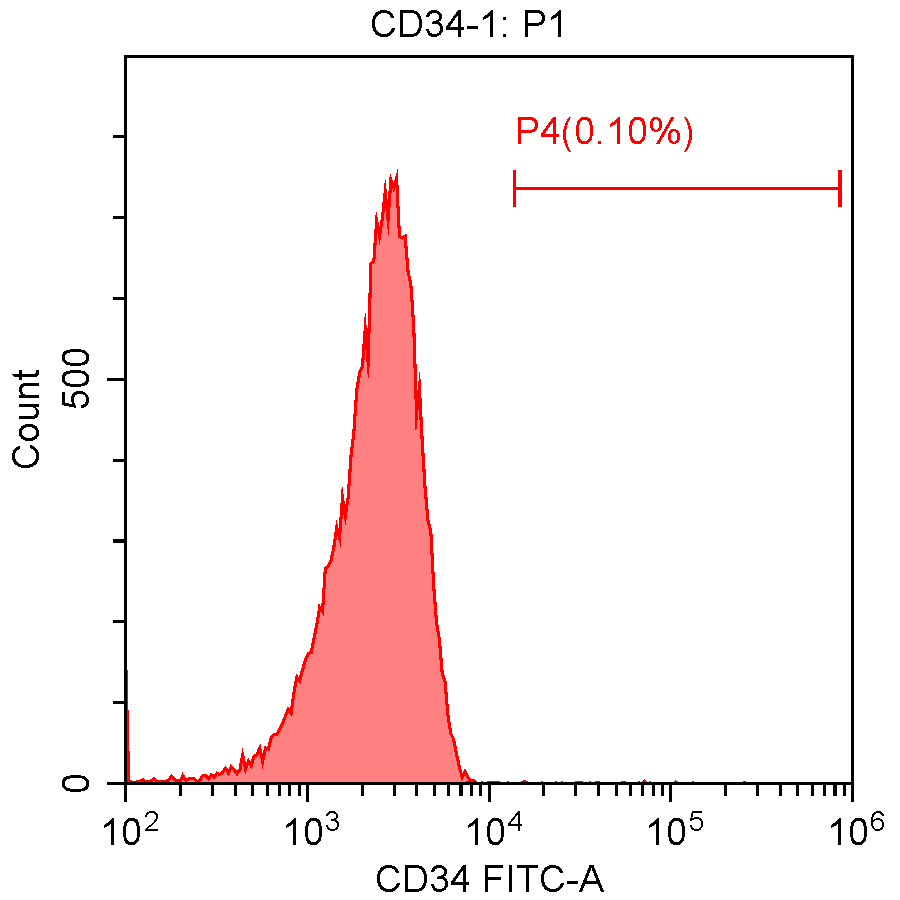

Supplement: S2 File — (ZIP) [file pone.0191616.s002.zip › Original data underlying the findings described in manuscript-Identification of MSC ,Exosomes and CSCs/CSCs identification/CD34-1_Plot1.bmp]

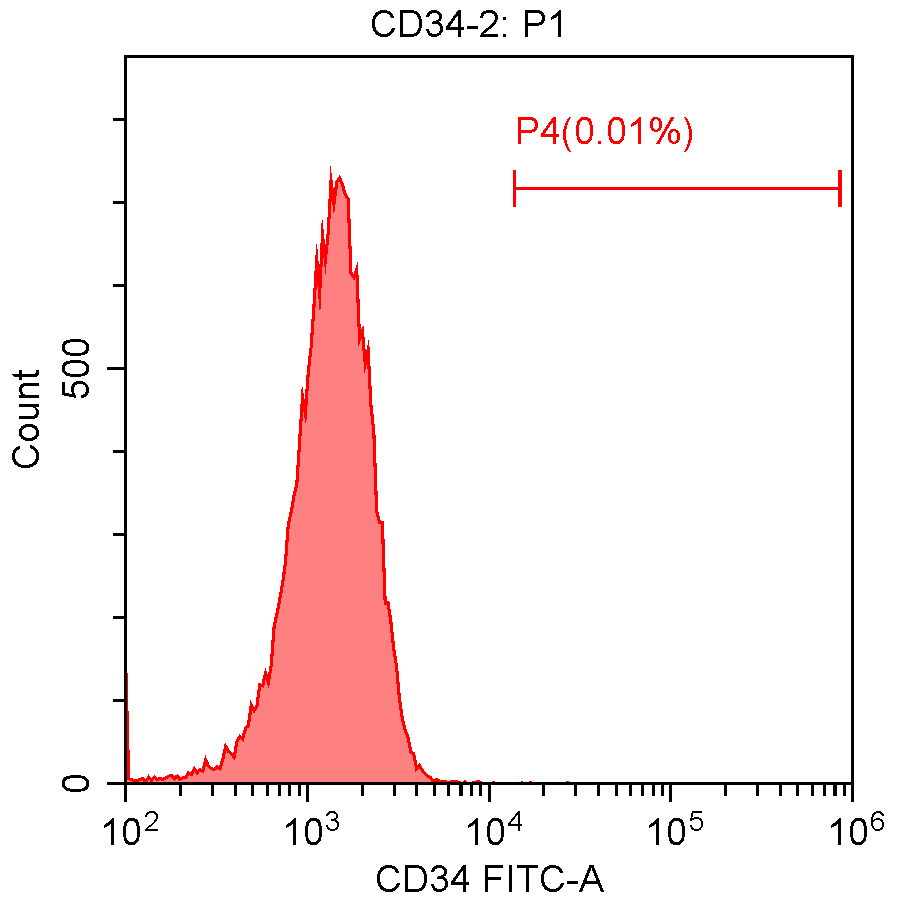

Supplement: S2 File — (ZIP) [file pone.0191616.s002.zip › Original data underlying the findings described in manuscript-Identification of MSC ,Exosomes and CSCs/CSCs identification/CD34-2_Plot1.bmp]

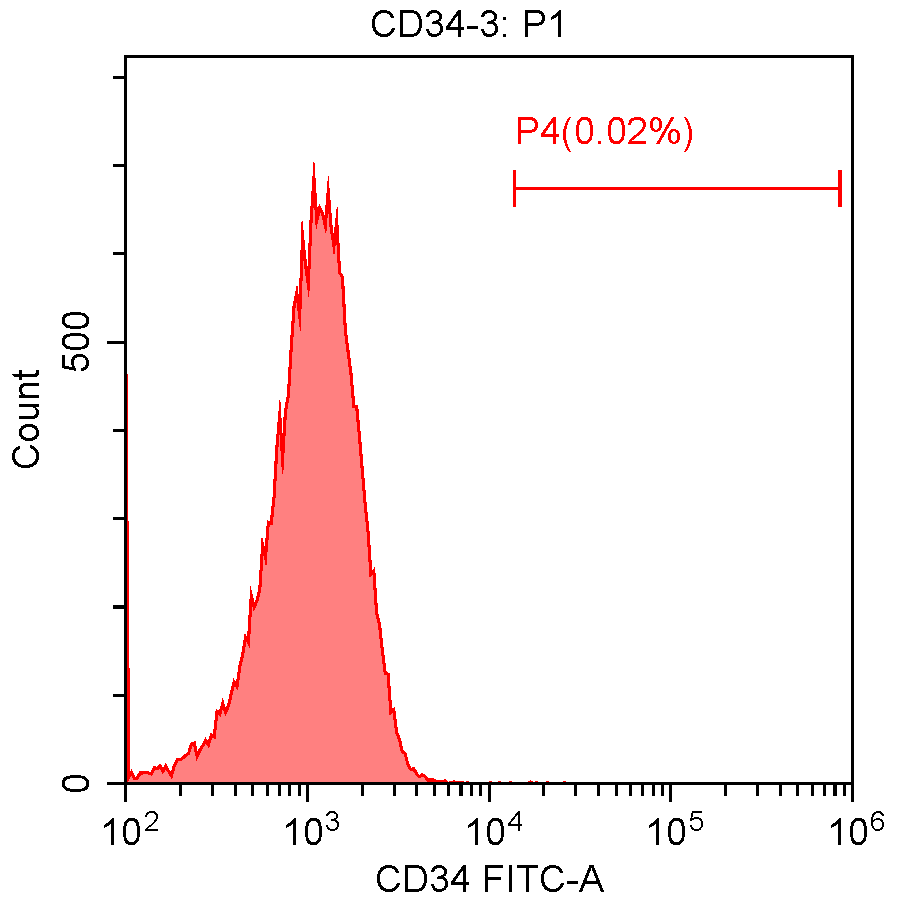

Supplement: S2 File — (ZIP) [file pone.0191616.s002.zip › Original data underlying the findings described in manuscript-Identification of MSC ,Exosomes and CSCs/CSCs identification/CD34-3_Plot1.bmp]

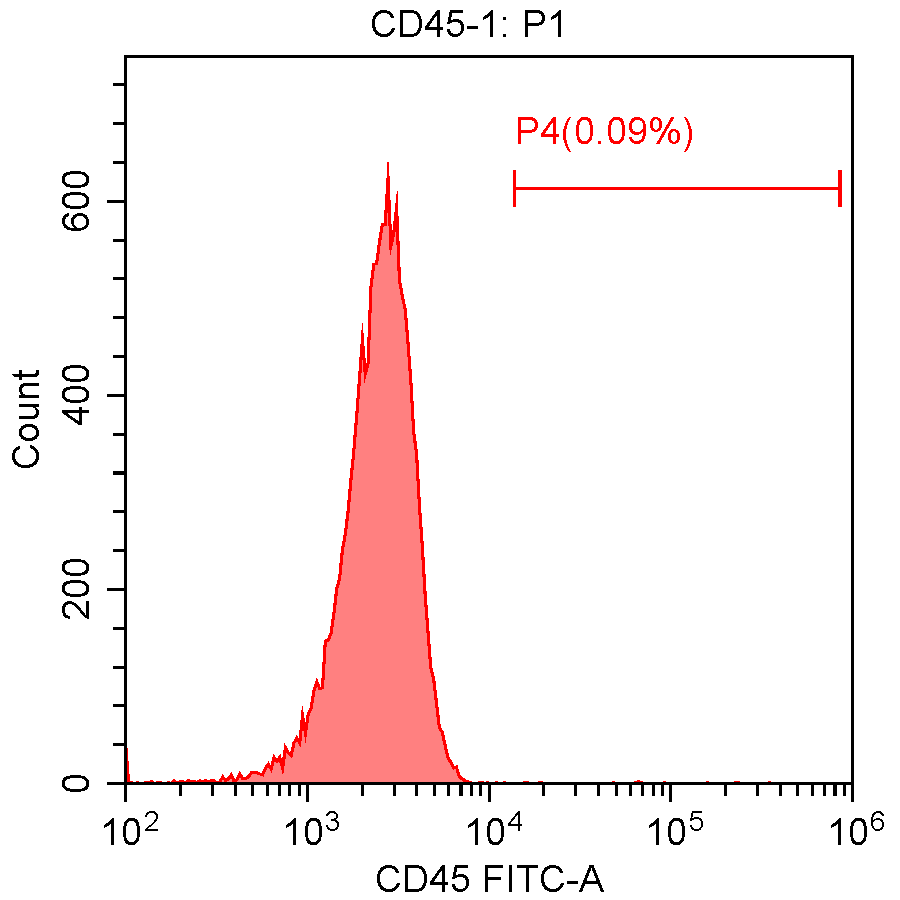

Supplement: S2 File — (ZIP) [file pone.0191616.s002.zip › Original data underlying the findings described in manuscript-Identification of MSC ,Exosomes and CSCs/CSCs identification/CD45-1_Plot1.bmp]

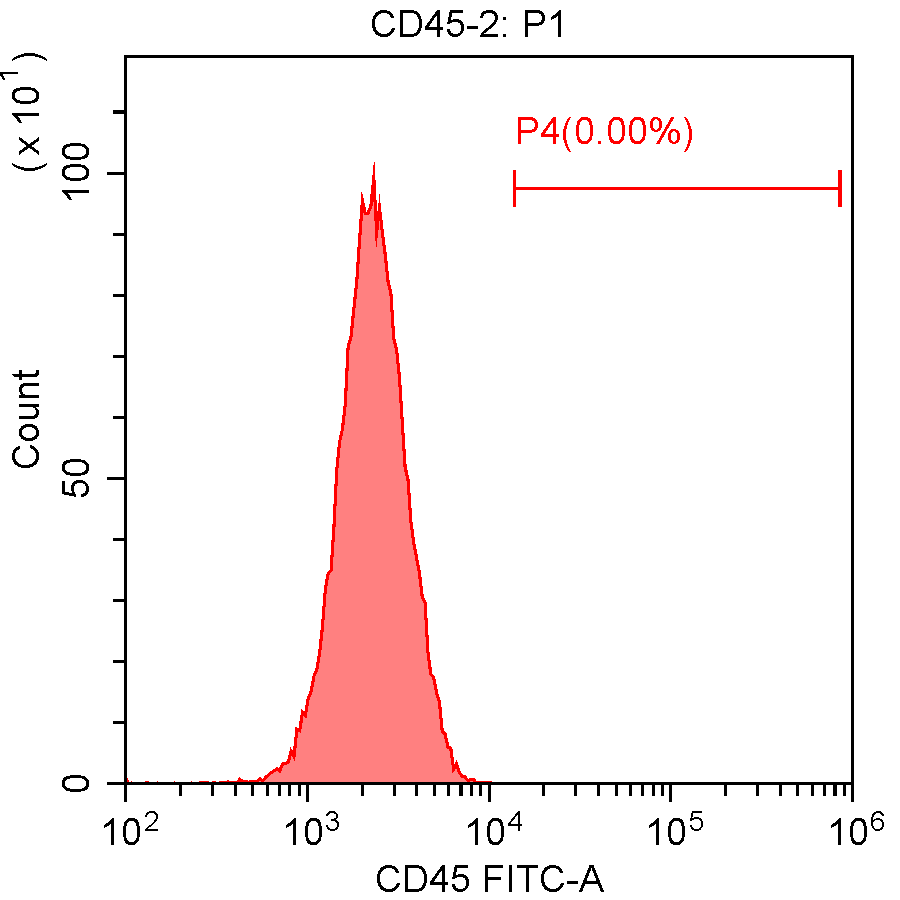

Supplement: S2 File — (ZIP) [file pone.0191616.s002.zip › Original data underlying the findings described in manuscript-Identification of MSC ,Exosomes and CSCs/CSCs identification/CD45-2_Plot1.bmp]

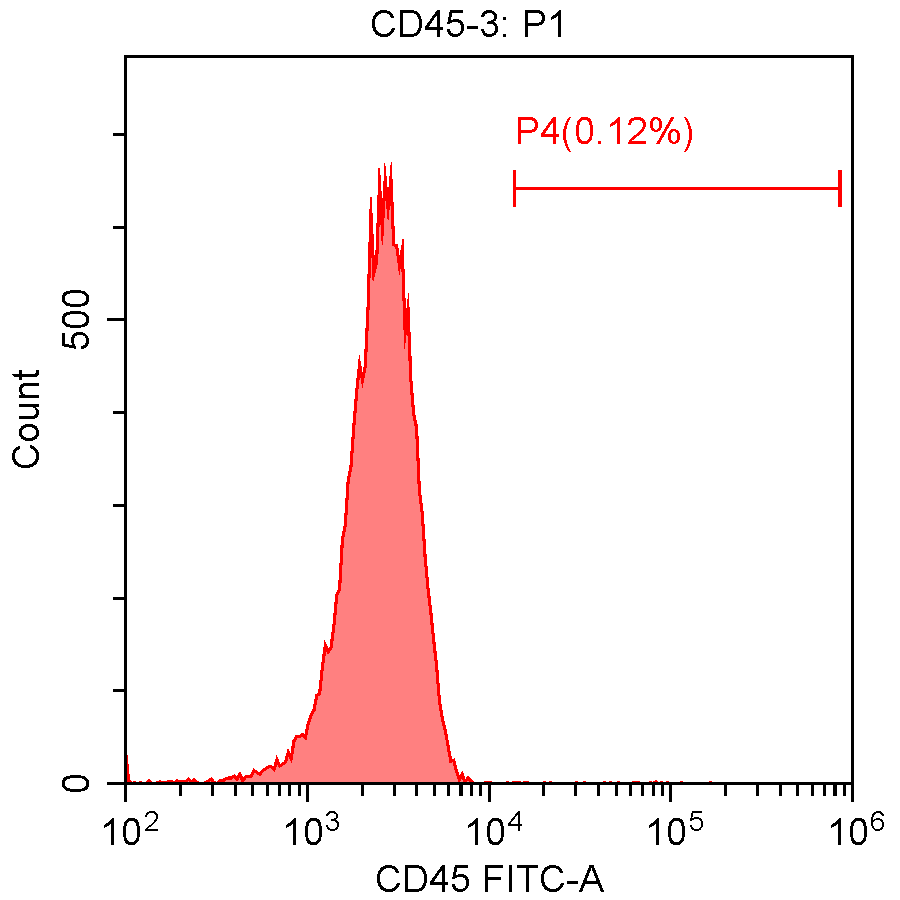

Supplement: S2 File — (ZIP) [file pone.0191616.s002.zip › Original data underlying the findings described in manuscript-Identification of MSC ,Exosomes and CSCs/CSCs identification/CD45-3_Plot1.bmp]

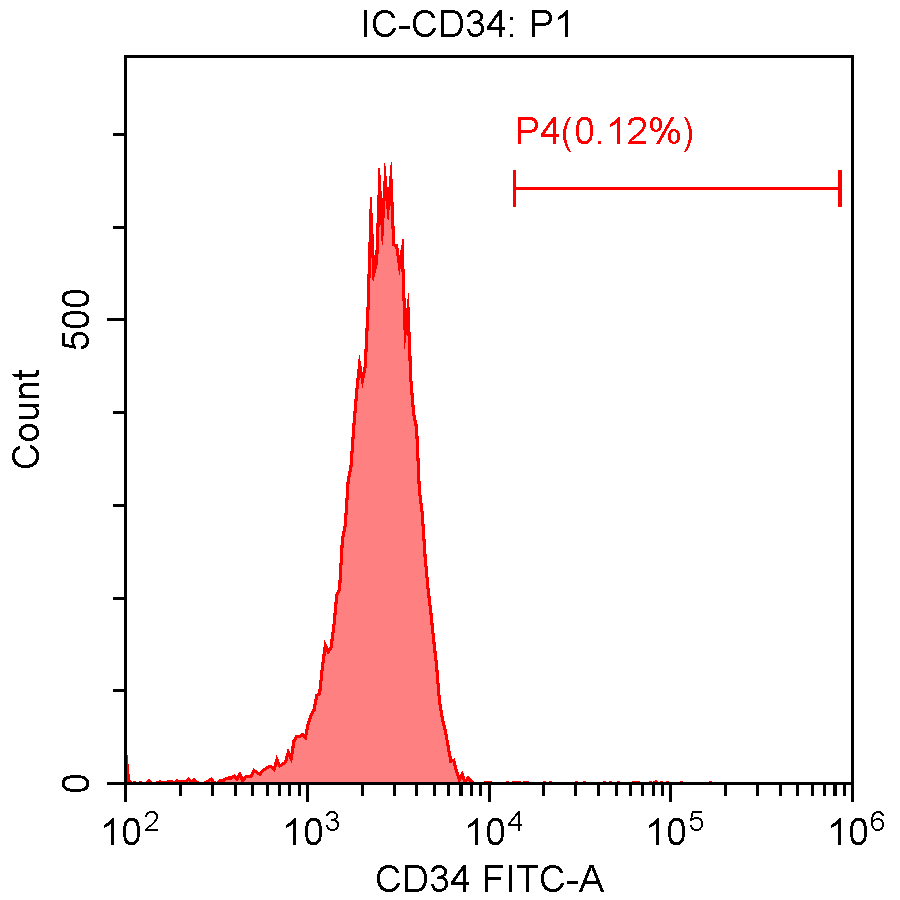

Supplement: S2 File — (ZIP) [file pone.0191616.s002.zip › Original data underlying the findings described in manuscript-Identification of MSC ,Exosomes and CSCs/CSCs identification/IC-CD34_Plot1.bmp]

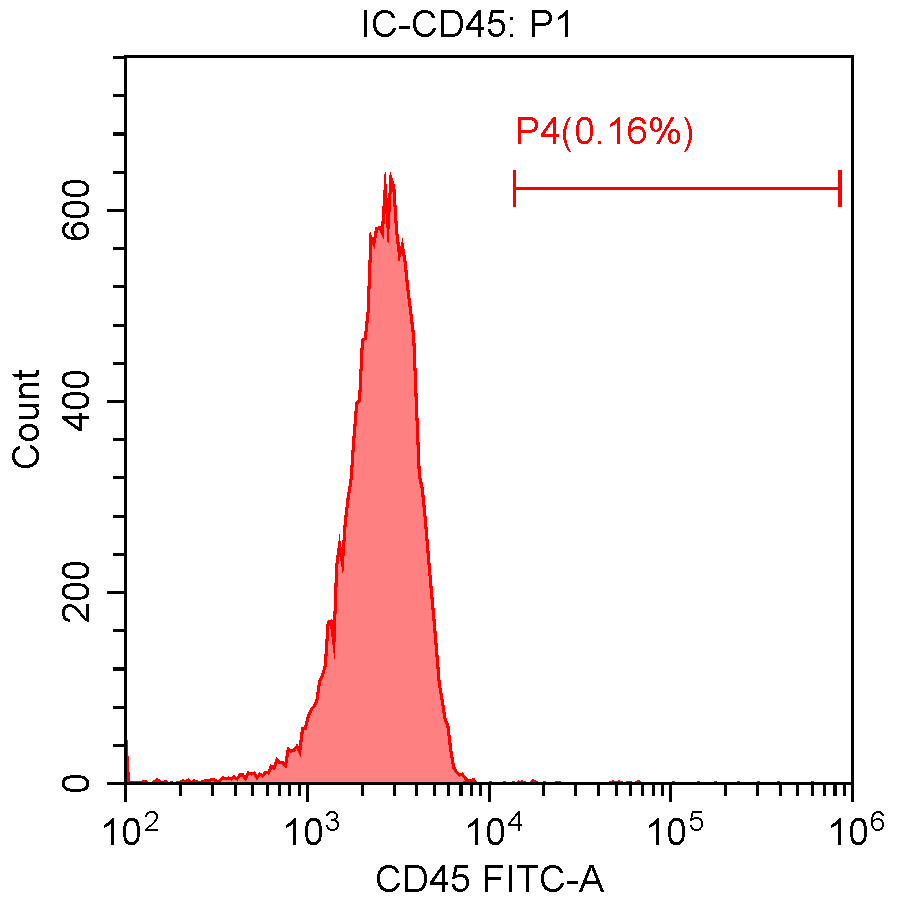

Supplement: S2 File — (ZIP) [file pone.0191616.s002.zip › Original data underlying the findings described in manuscript-Identification of MSC ,Exosomes and CSCs/CSCs identification/IC-CD45_Plot1.bmp]

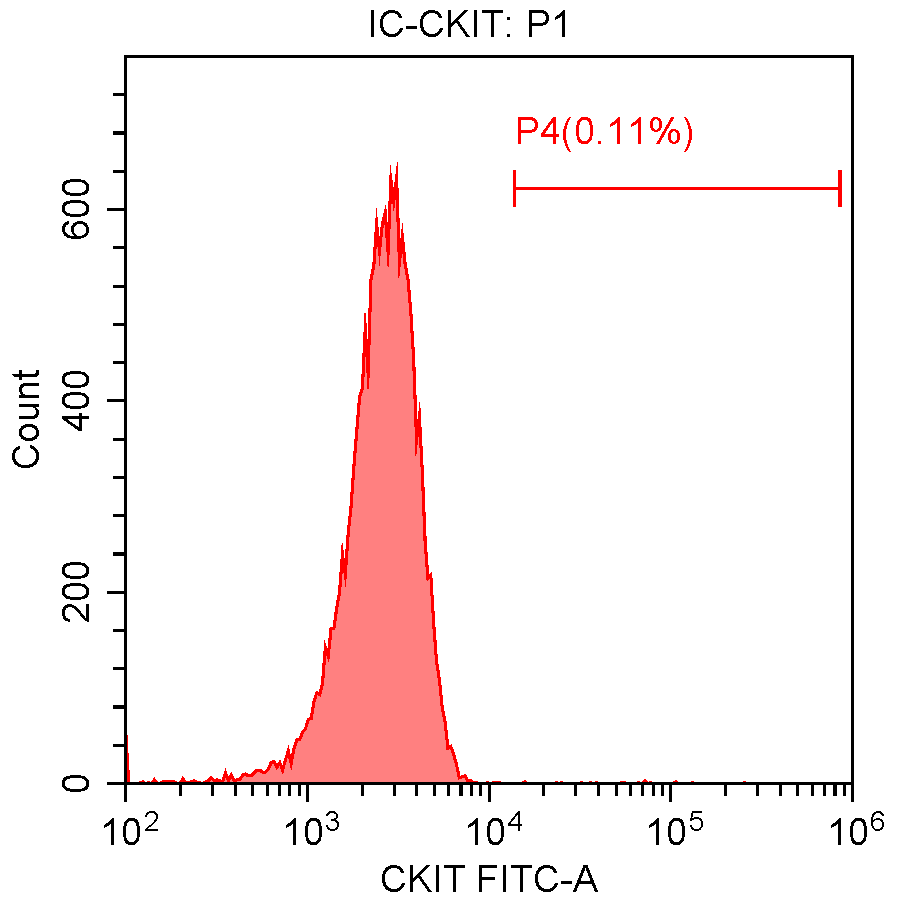

Supplement: S2 File — (ZIP) [file pone.0191616.s002.zip › Original data underlying the findings described in manuscript-Identification of MSC ,Exosomes and CSCs/CSCs identification/IC-CKIT_Plot1.bmp]

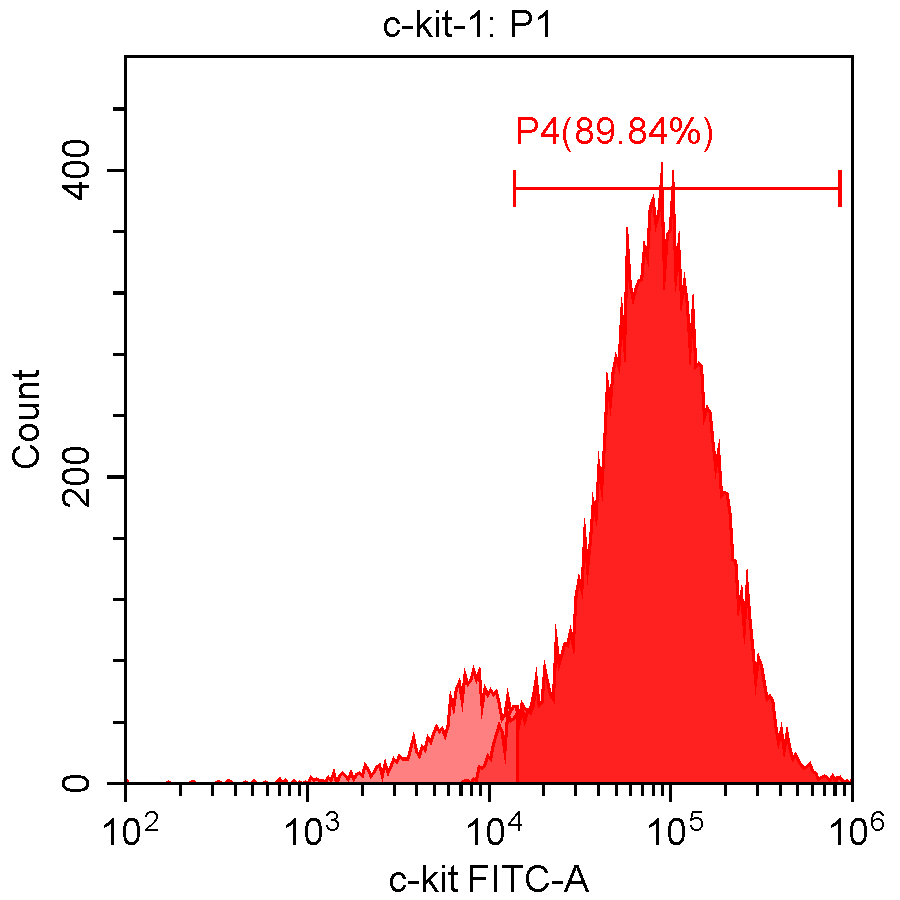

Supplement: S2 File — (ZIP) [file pone.0191616.s002.zip › Original data underlying the findings described in manuscript-Identification of MSC ,Exosomes and CSCs/CSCs identification/c-kit-1_Plot1.bmp]

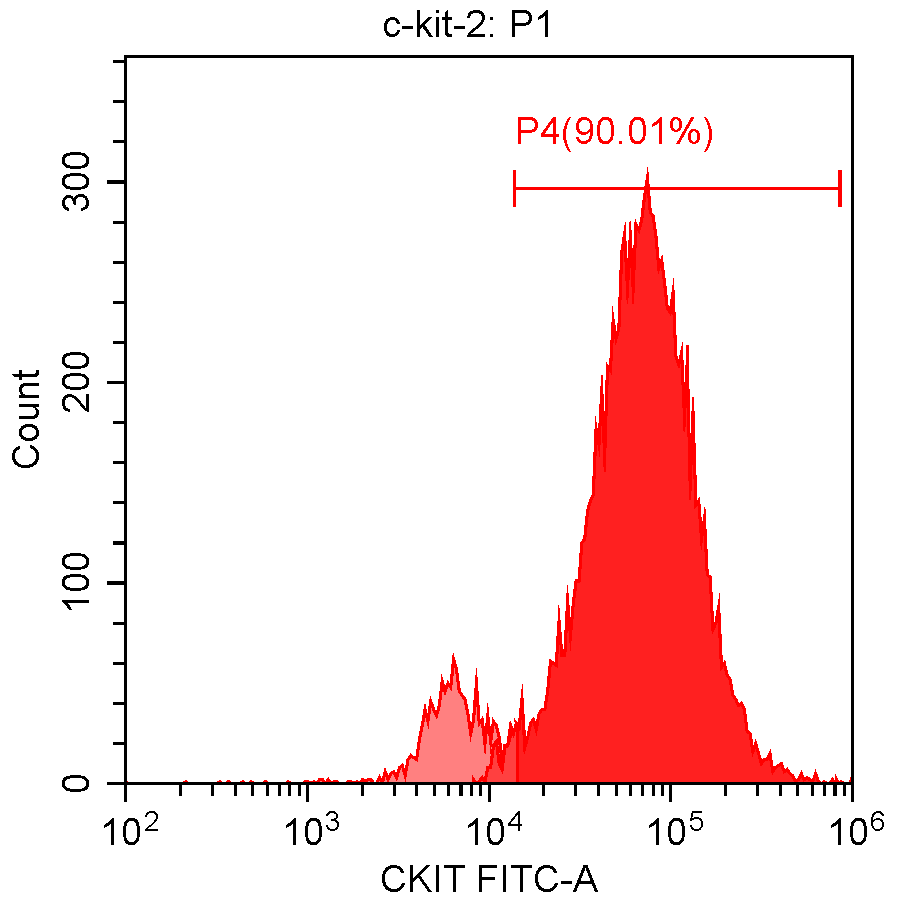

Supplement: S2 File — (ZIP) [file pone.0191616.s002.zip › Original data underlying the findings described in manuscript-Identification of MSC ,Exosomes and CSCs/CSCs identification/c-kit-2_Plot1.bmp]

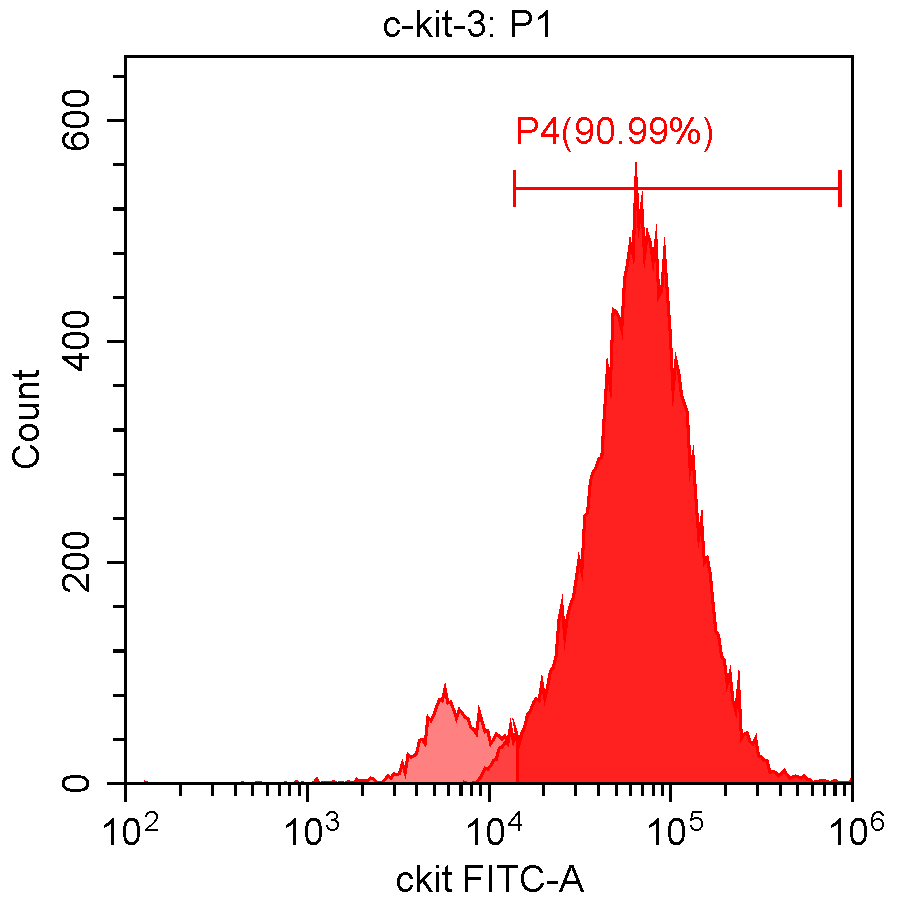

Supplement: S2 File — (ZIP) [file pone.0191616.s002.zip › Original data underlying the findings described in manuscript-Identification of MSC ,Exosomes and CSCs/CSCs identification/c-kit-3_Plot1.bmp]

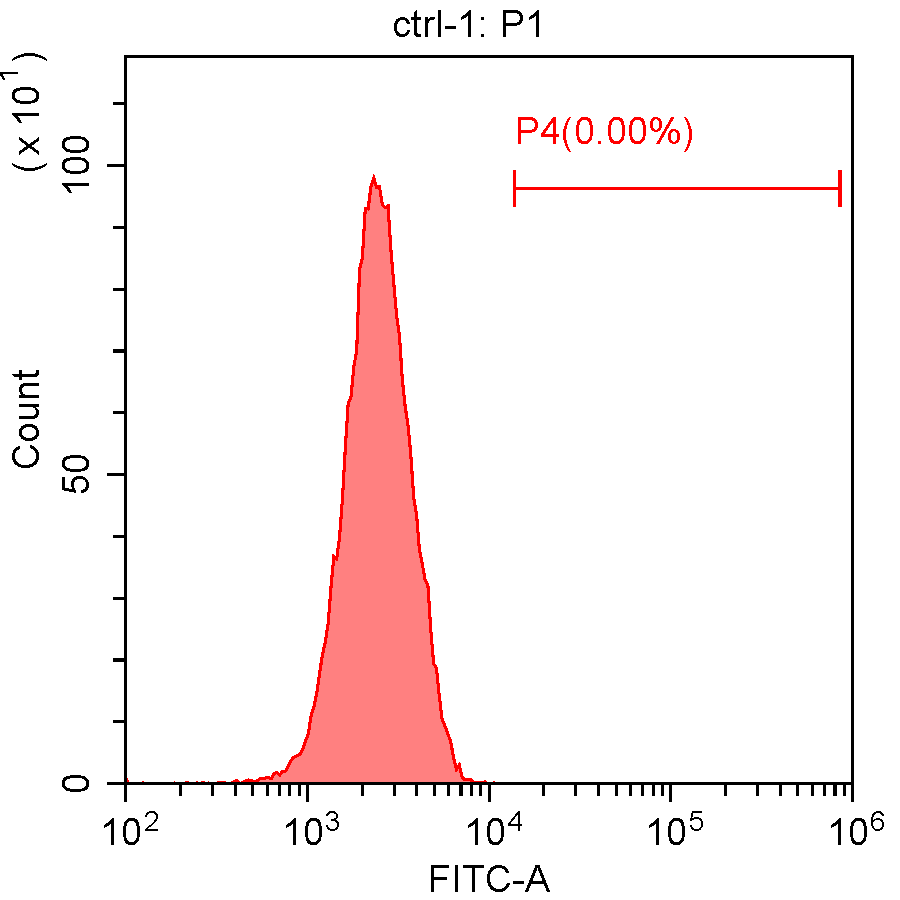

Supplement: S2 File — (ZIP) [file pone.0191616.s002.zip › Original data underlying the findings described in manuscript-Identification of MSC ,Exosomes and CSCs/CSCs identification/ctrl-1_Plot1.bmp]

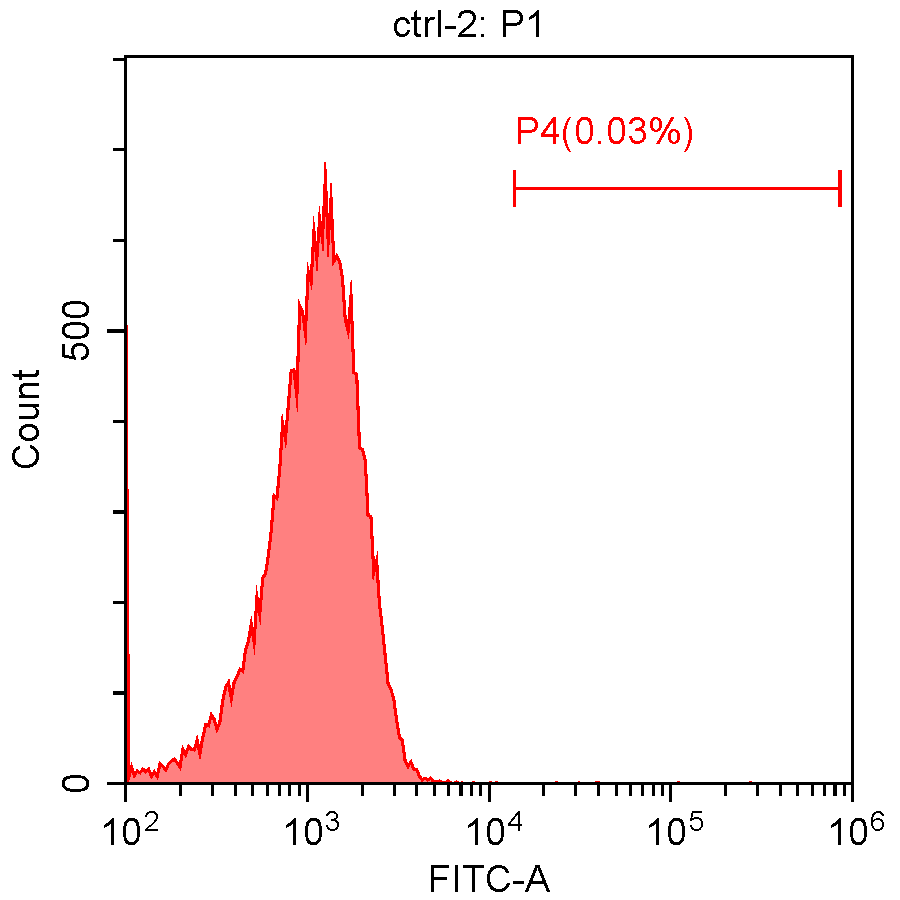

Supplement: S2 File — (ZIP) [file pone.0191616.s002.zip › Original data underlying the findings described in manuscript-Identification of MSC ,Exosomes and CSCs/CSCs identification/ctrl-2_Plot1.bmp]

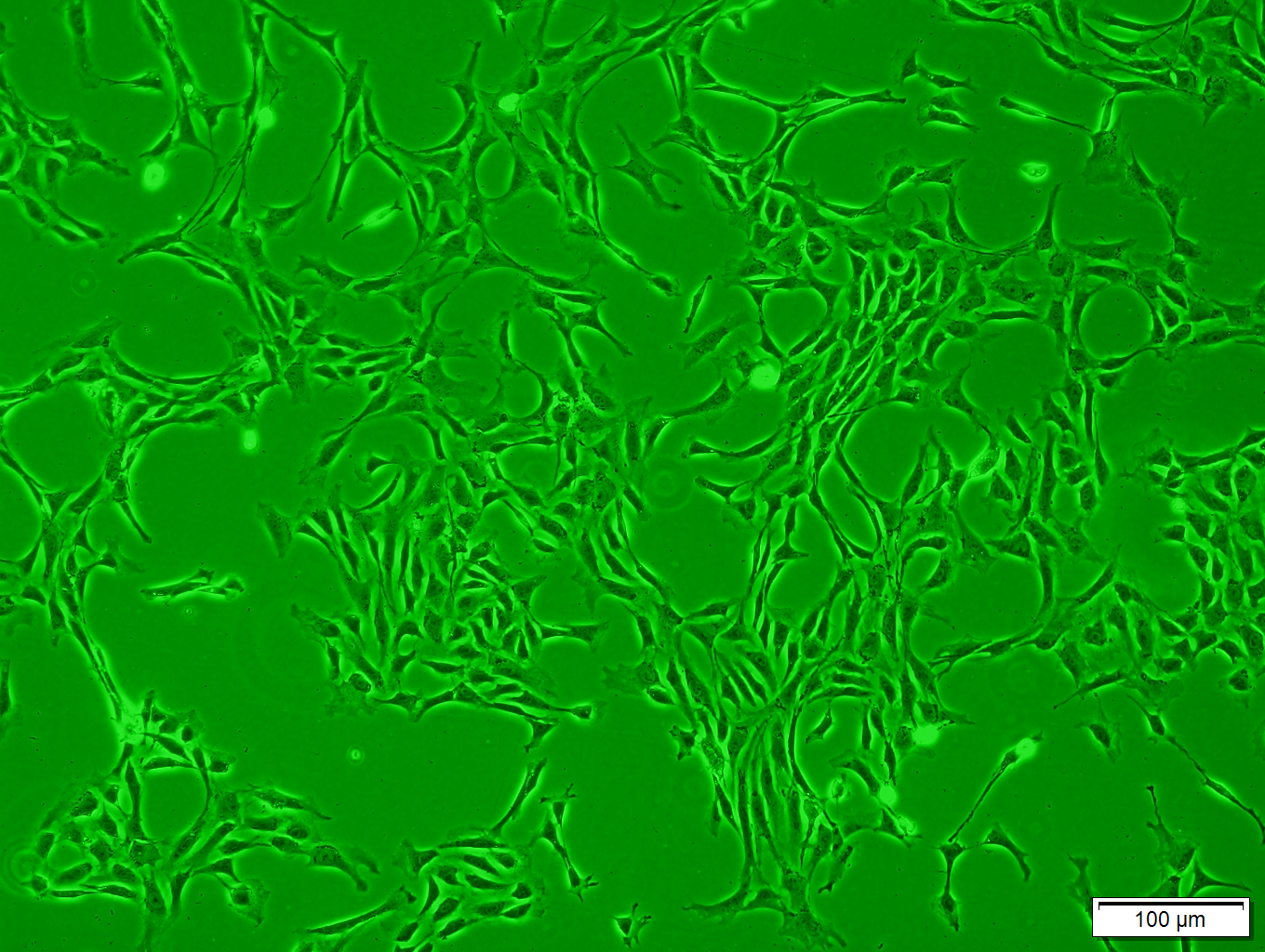

Supplement: S2 File — (ZIP) [file pone.0191616.s002.zip › Original data underlying the findings described in manuscript-Identification of MSC ,Exosomes and CSCs/CSCs.tif]

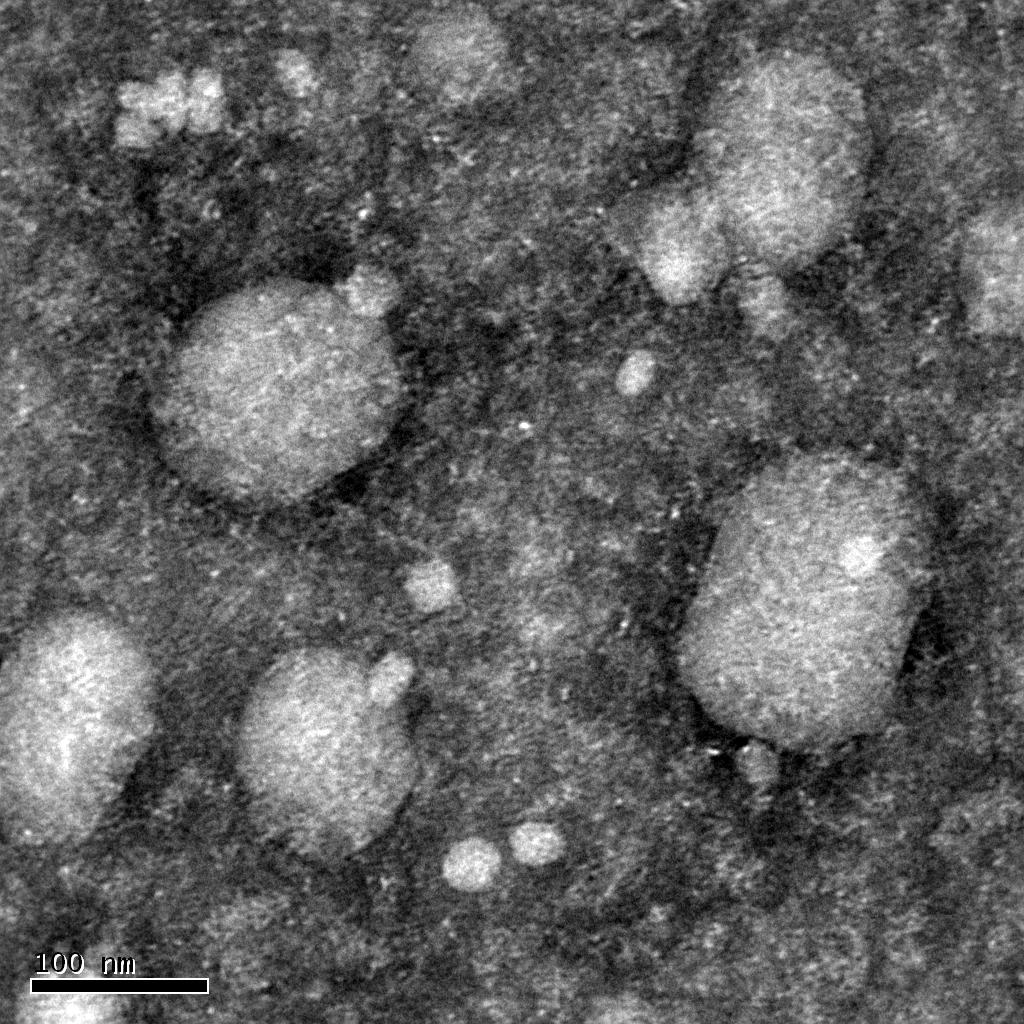

Supplement: S2 File — (ZIP) [file pone.0191616.s002.zip › Original data underlying the findings described in manuscript-Identification of MSC ,Exosomes and CSCs/H2O2-exosome/200000.0V-30000X-2157.jpg]

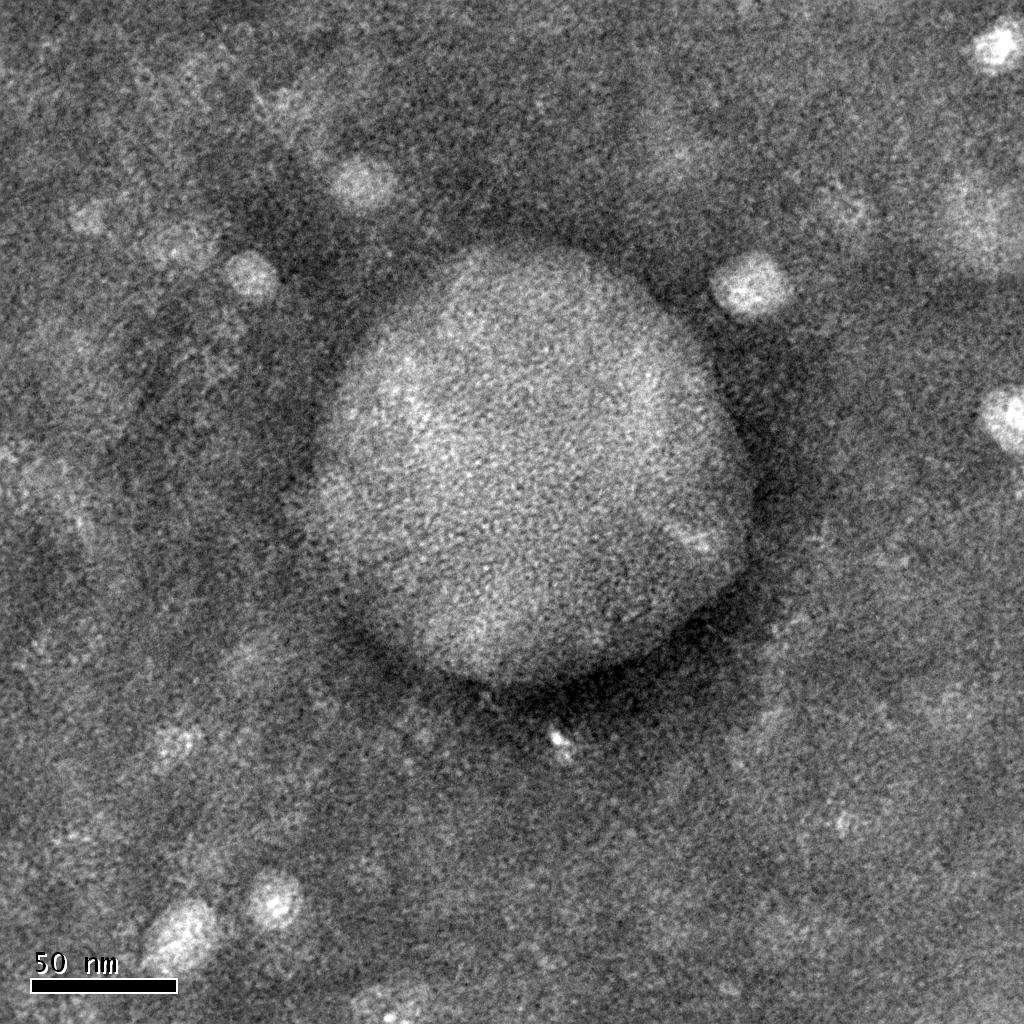

Supplement: S2 File — (ZIP) [file pone.0191616.s002.zip › Original data underlying the findings described in manuscript-Identification of MSC ,Exosomes and CSCs/H2O2-exosome/200000.0V-50000X-2119.jpg]

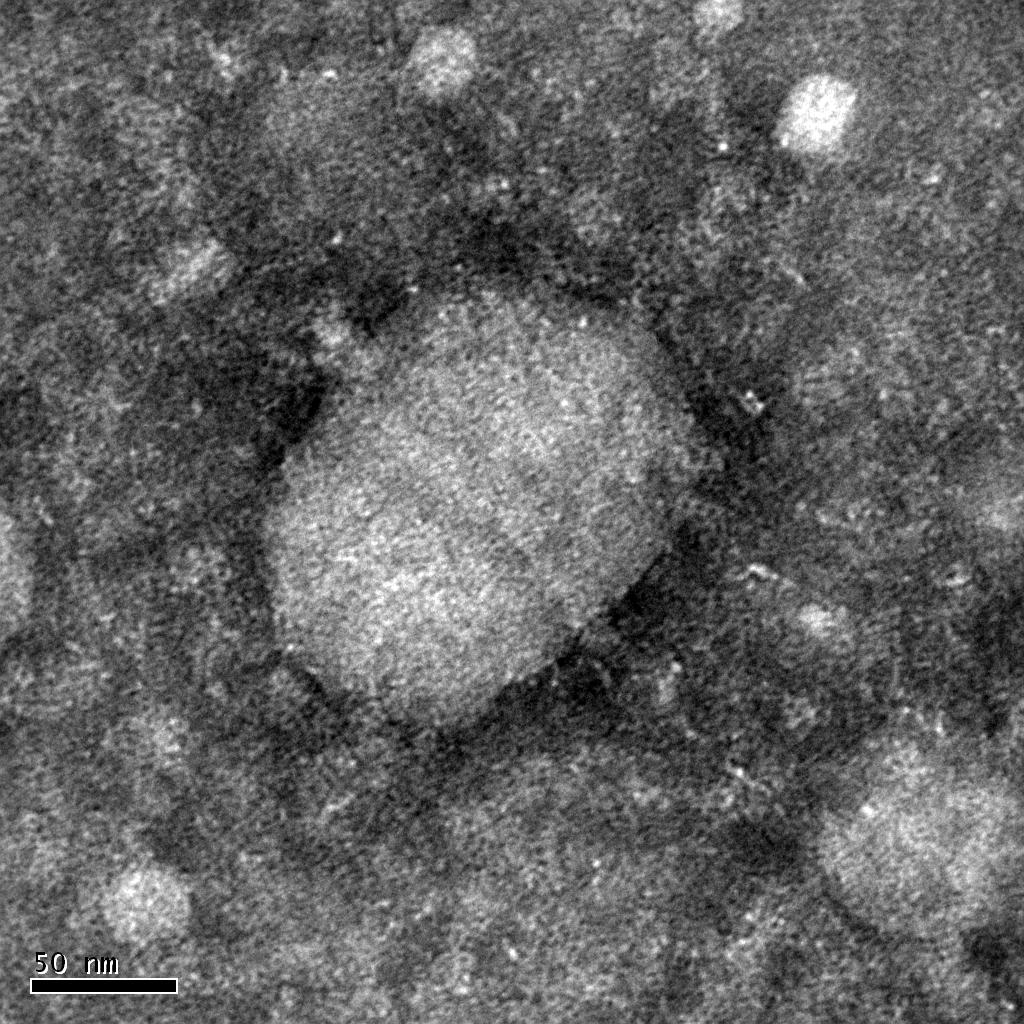

Supplement: S2 File — (ZIP) [file pone.0191616.s002.zip › Original data underlying the findings described in manuscript-Identification of MSC ,Exosomes and CSCs/H2O2-exosome/200000.0V-50000X-2160.jpg]

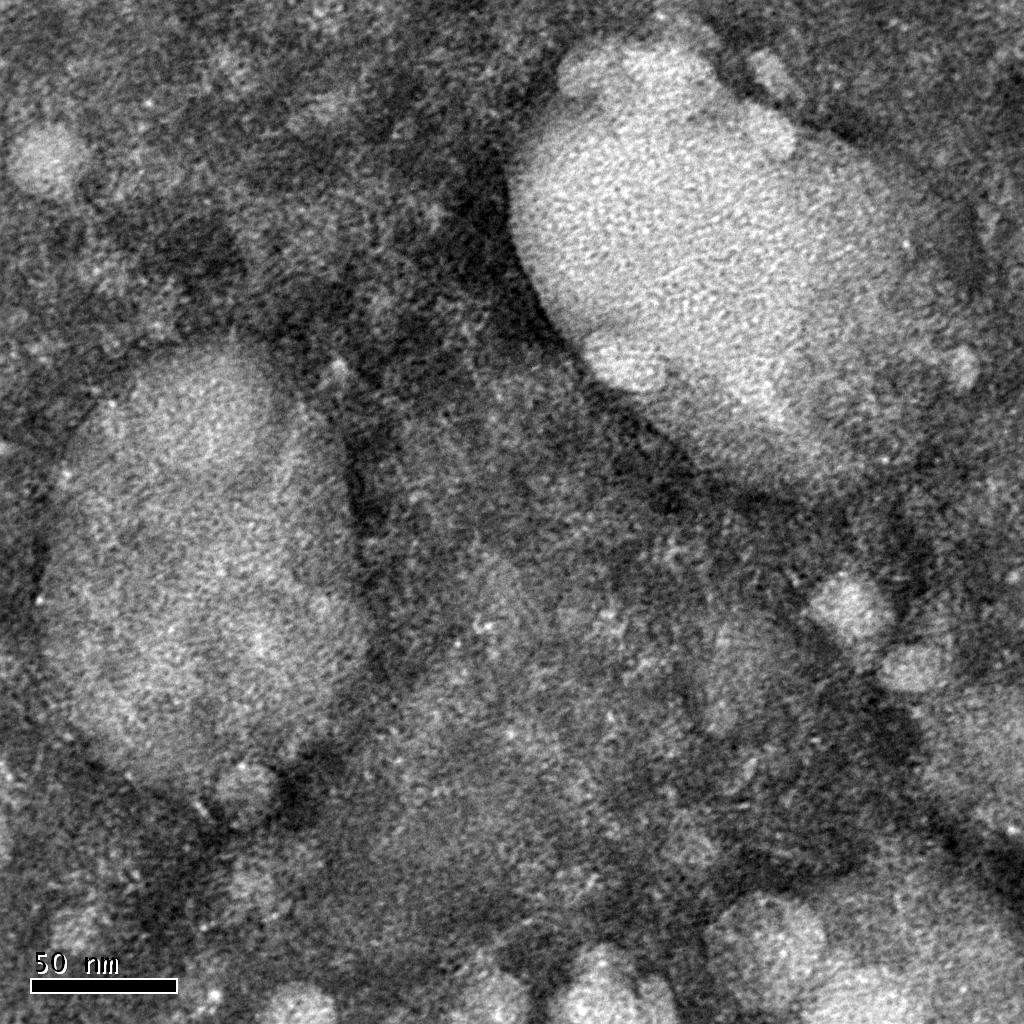

Supplement: S2 File — (ZIP) [file pone.0191616.s002.zip › Original data underlying the findings described in manuscript-Identification of MSC ,Exosomes and CSCs/H2O2-exosome/200000.0V-50000X-2162.jpg]

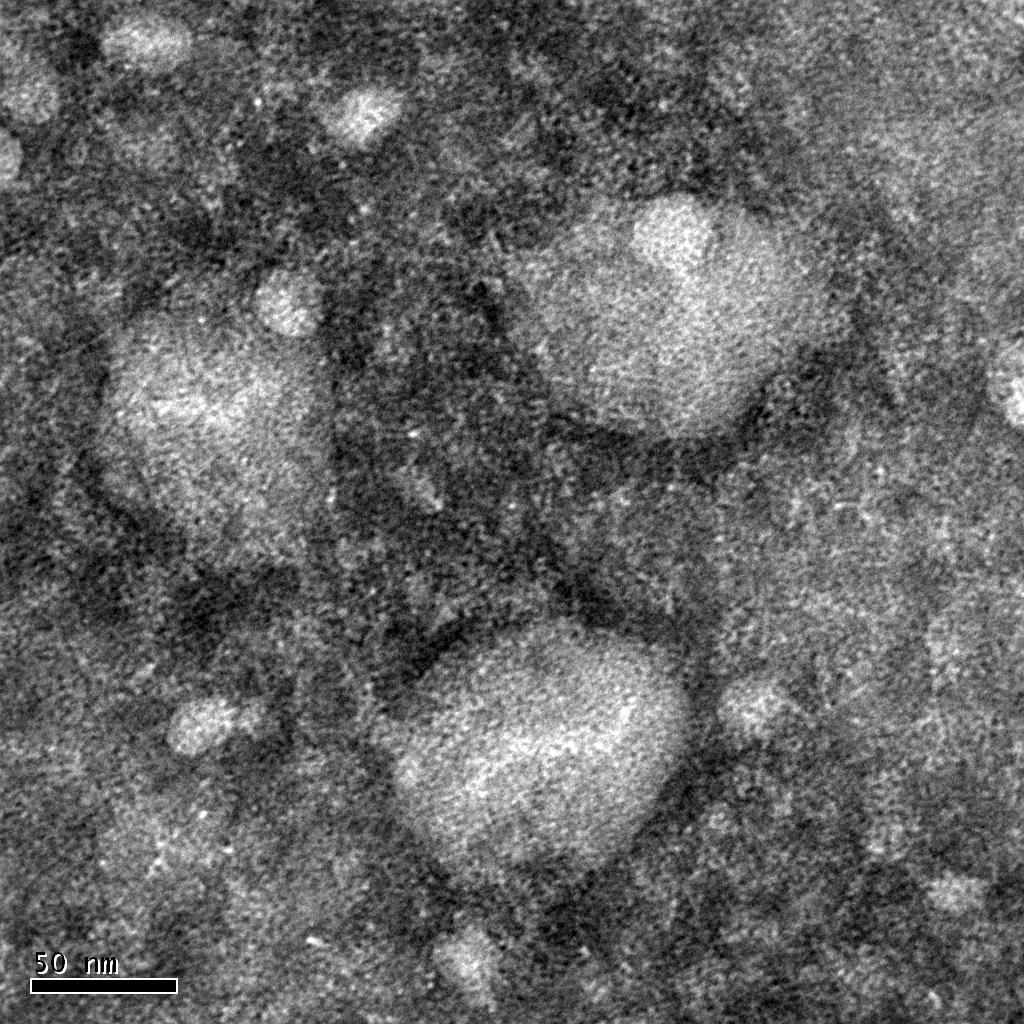

Supplement: S2 File — (ZIP) [file pone.0191616.s002.zip › Original data underlying the findings described in manuscript-Identification of MSC ,Exosomes and CSCs/H2O2-exosome/200000.0V-50000X-2164.jpg]

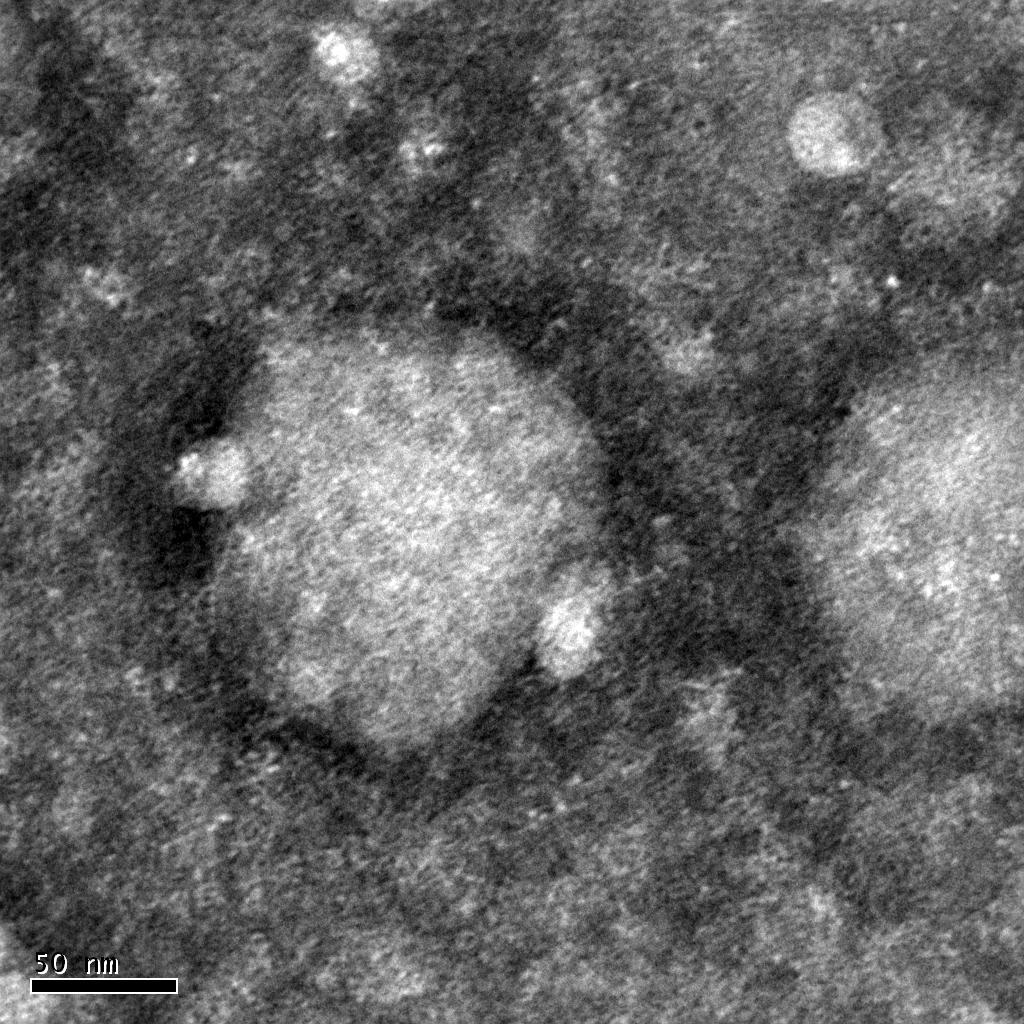

Supplement: S2 File — (ZIP) [file pone.0191616.s002.zip › Original data underlying the findings described in manuscript-Identification of MSC ,Exosomes and CSCs/H2O2-exosome/200000.0V-50000X-2165.jpg]

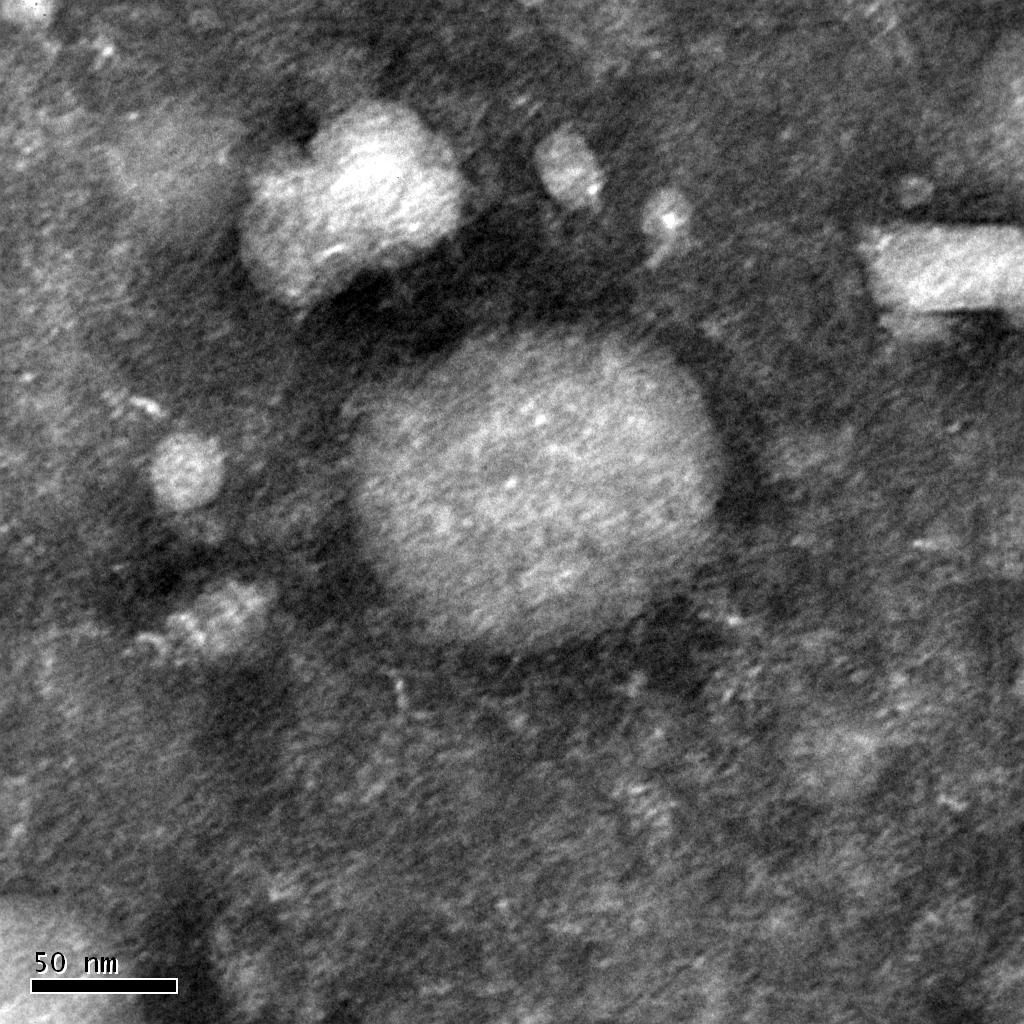

Supplement: S2 File — (ZIP) [file pone.0191616.s002.zip › Original data underlying the findings described in manuscript-Identification of MSC ,Exosomes and CSCs/H2O2-exosome/200000.0V-50000X-2167.jpg]

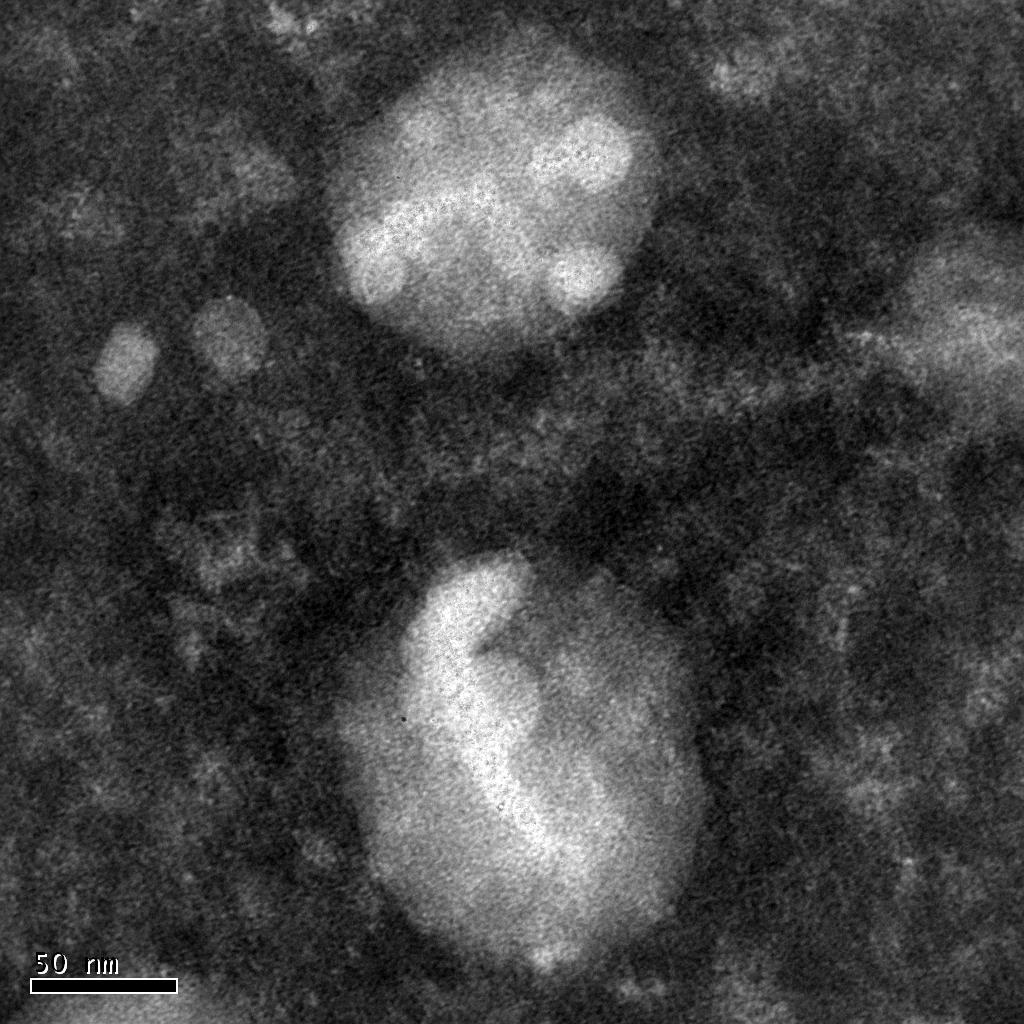

Supplement: S2 File — (ZIP) [file pone.0191616.s002.zip › Original data underlying the findings described in manuscript-Identification of MSC ,Exosomes and CSCs/H2O2-exosome/200000.0V-50000X-2171.jpg]

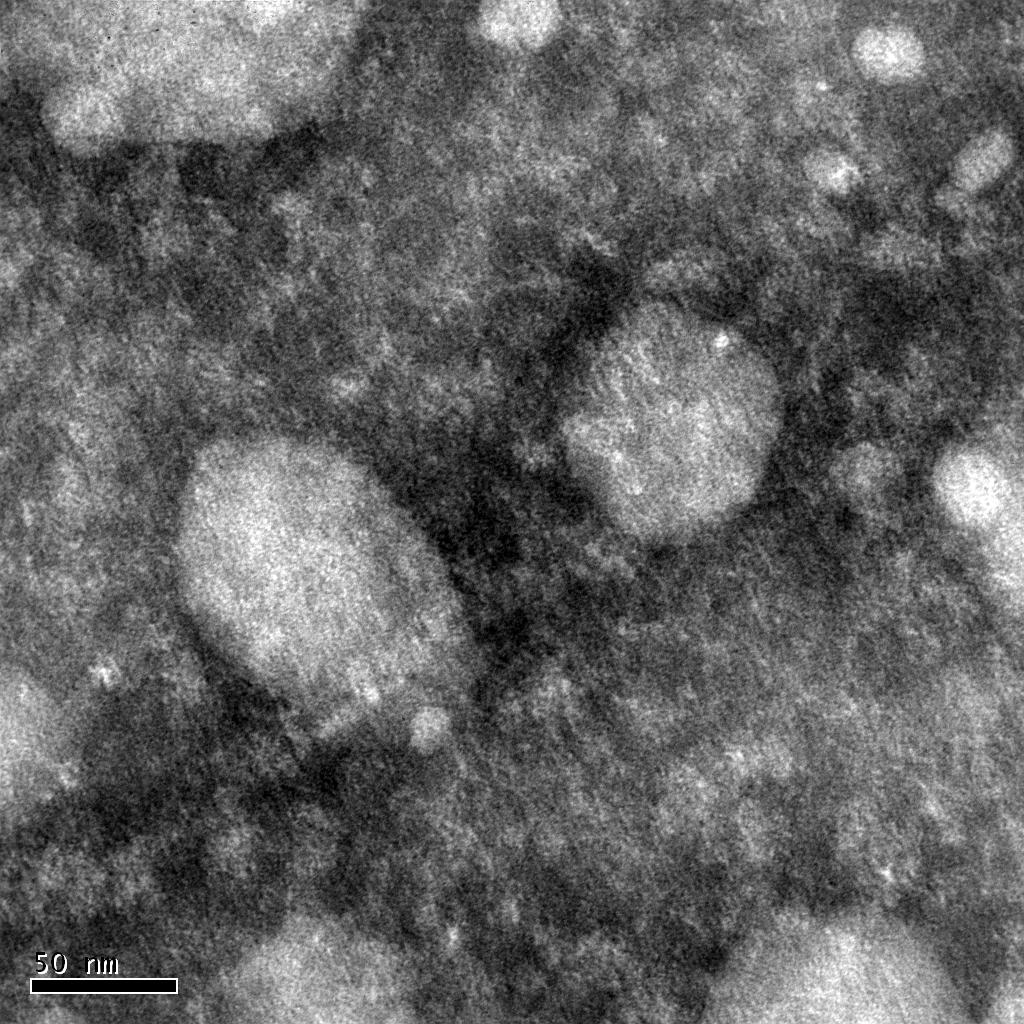

Supplement: S2 File — (ZIP) [file pone.0191616.s002.zip › Original data underlying the findings described in manuscript-Identification of MSC ,Exosomes and CSCs/H2O2-exosome/200000.0V-50000X-2173.jpg]

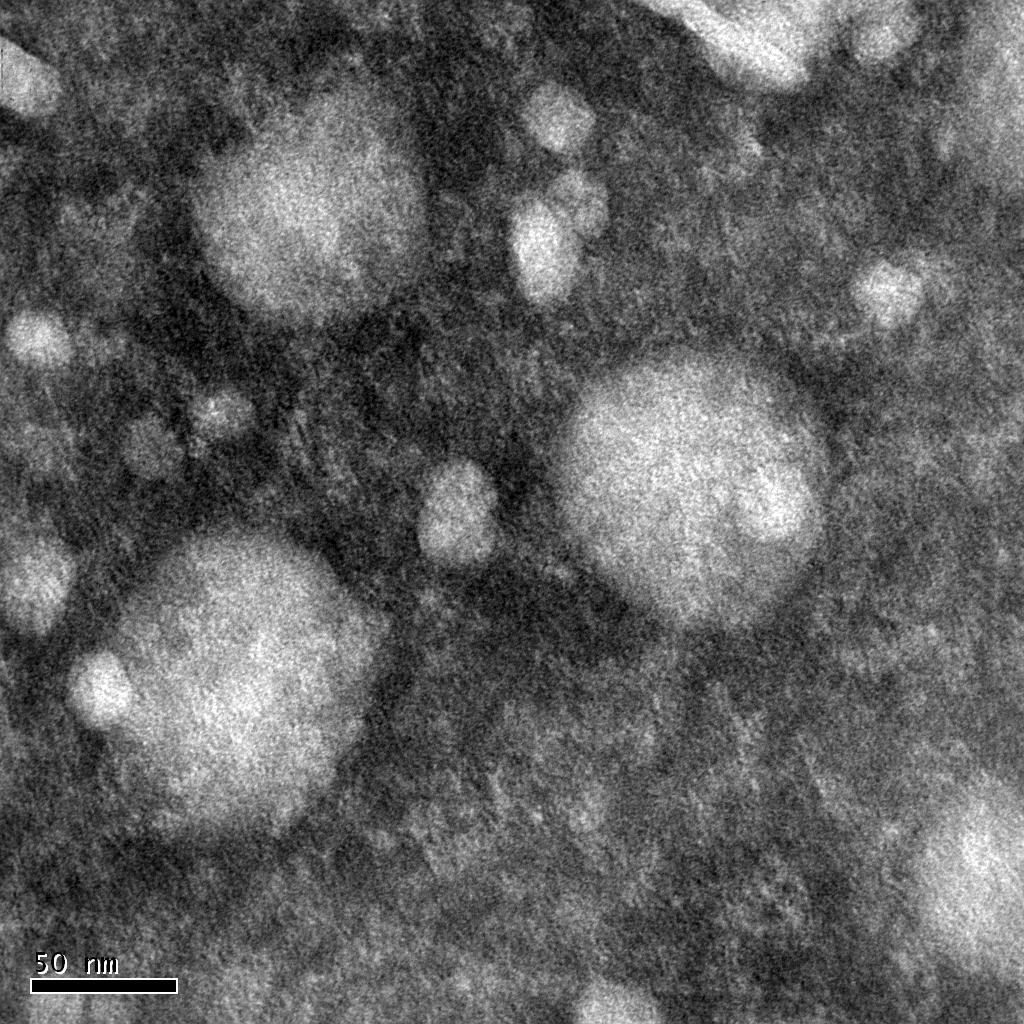

Supplement: S2 File — (ZIP) [file pone.0191616.s002.zip › Original data underlying the findings described in manuscript-Identification of MSC ,Exosomes and CSCs/H2O2-exosome/200000.0V-50000X-2174.jpg]

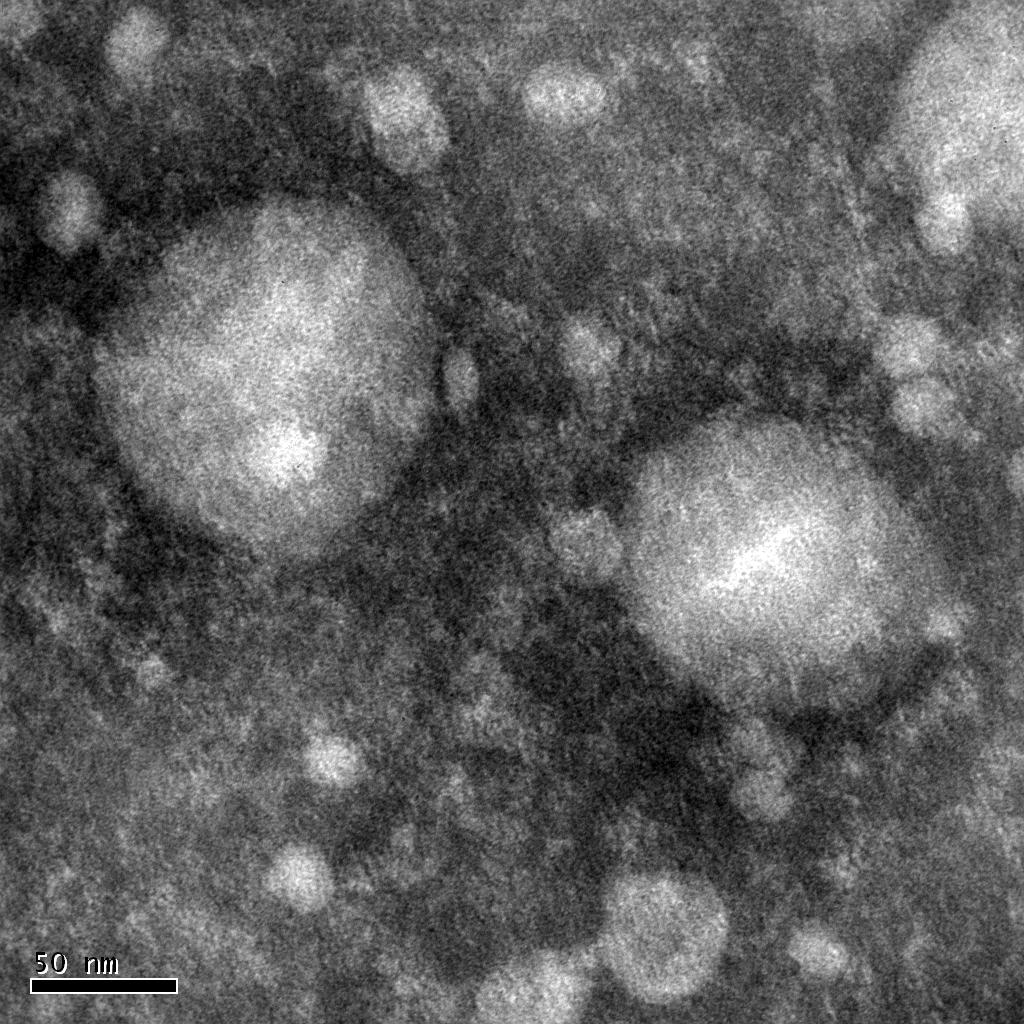

Supplement: S2 File — (ZIP) [file pone.0191616.s002.zip › Original data underlying the findings described in manuscript-Identification of MSC ,Exosomes and CSCs/H2O2-exosome/200000.0V-50000X-2175.jpg]

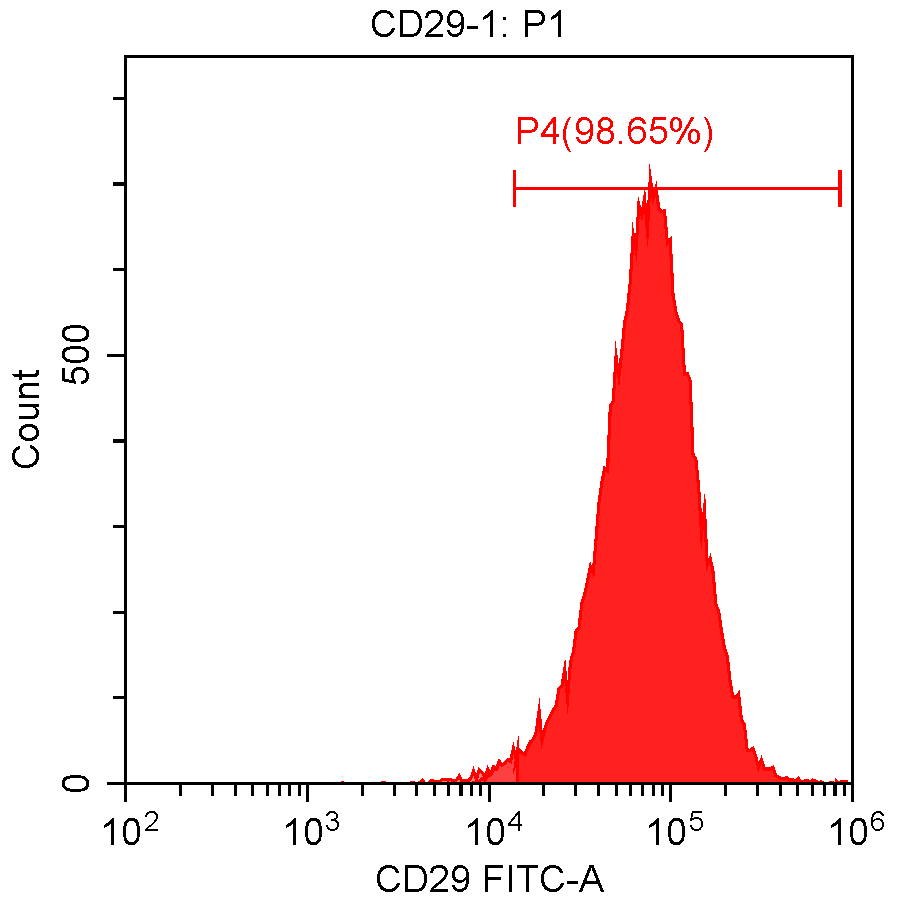

Supplement: S2 File — (ZIP) [file pone.0191616.s002.zip › Original data underlying the findings described in manuscript-Identification of MSC ,Exosomes and CSCs/MSCs identification/CD29-1_Plot1.bmp]

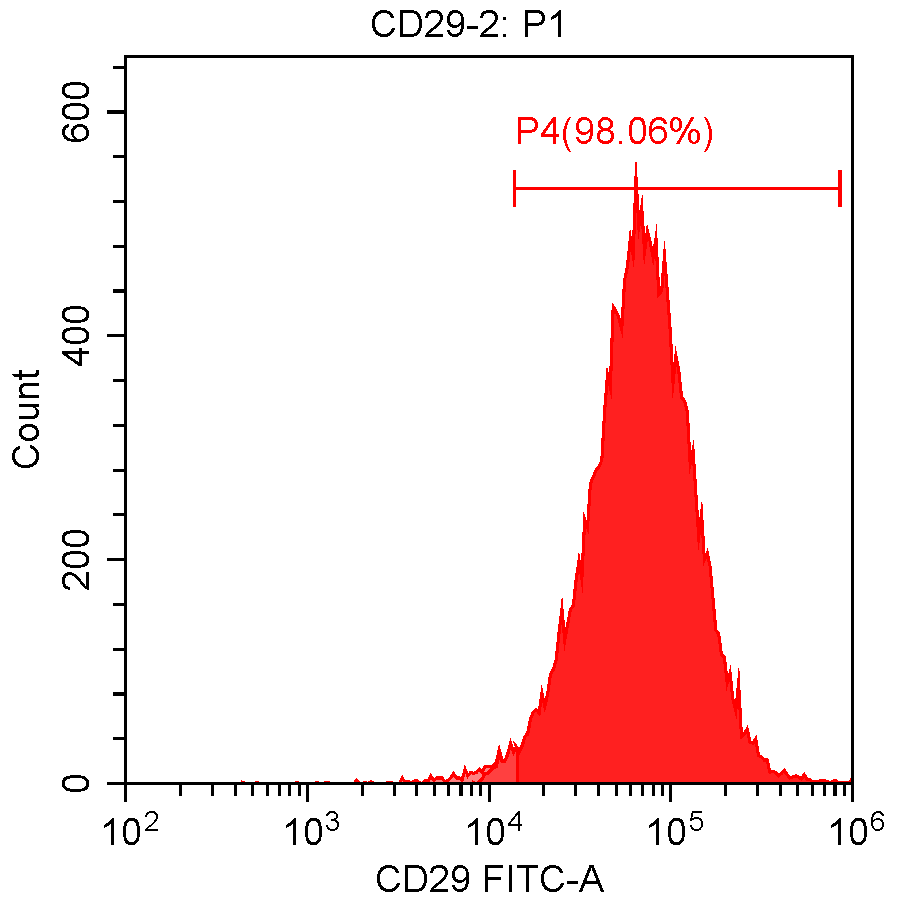

Supplement: S2 File — (ZIP) [file pone.0191616.s002.zip › Original data underlying the findings described in manuscript-Identification of MSC ,Exosomes and CSCs/MSCs identification/CD29-2_Plot1.bmp]

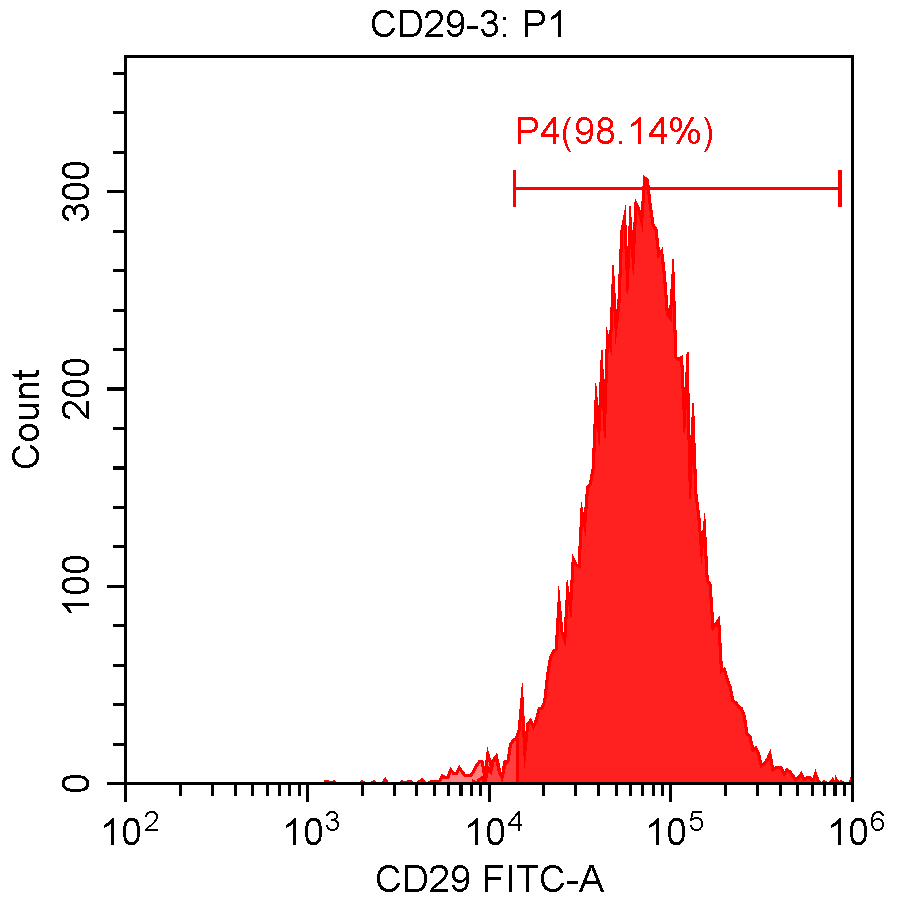

Supplement: S2 File — (ZIP) [file pone.0191616.s002.zip › Original data underlying the findings described in manuscript-Identification of MSC ,Exosomes and CSCs/MSCs identification/CD29-3_Plot1.bmp]

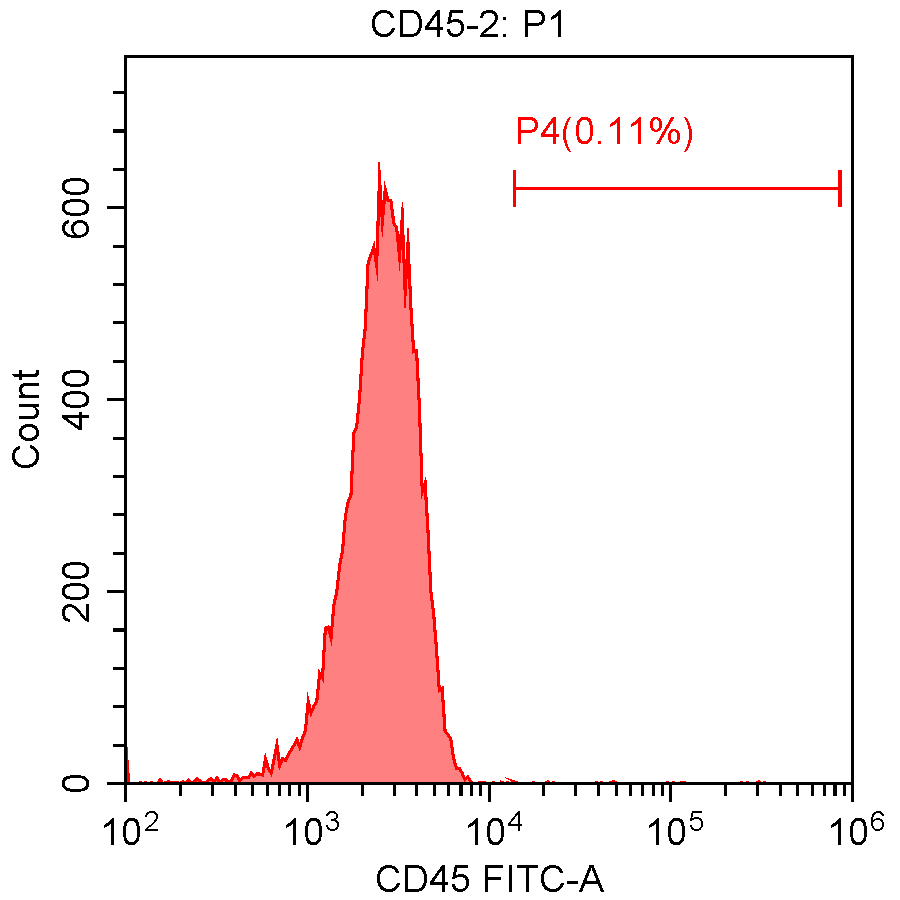

Supplement: S2 File — (ZIP) [file pone.0191616.s002.zip › Original data underlying the findings described in manuscript-Identification of MSC ,Exosomes and CSCs/MSCs identification/CD45-2_Plot1.bmp]

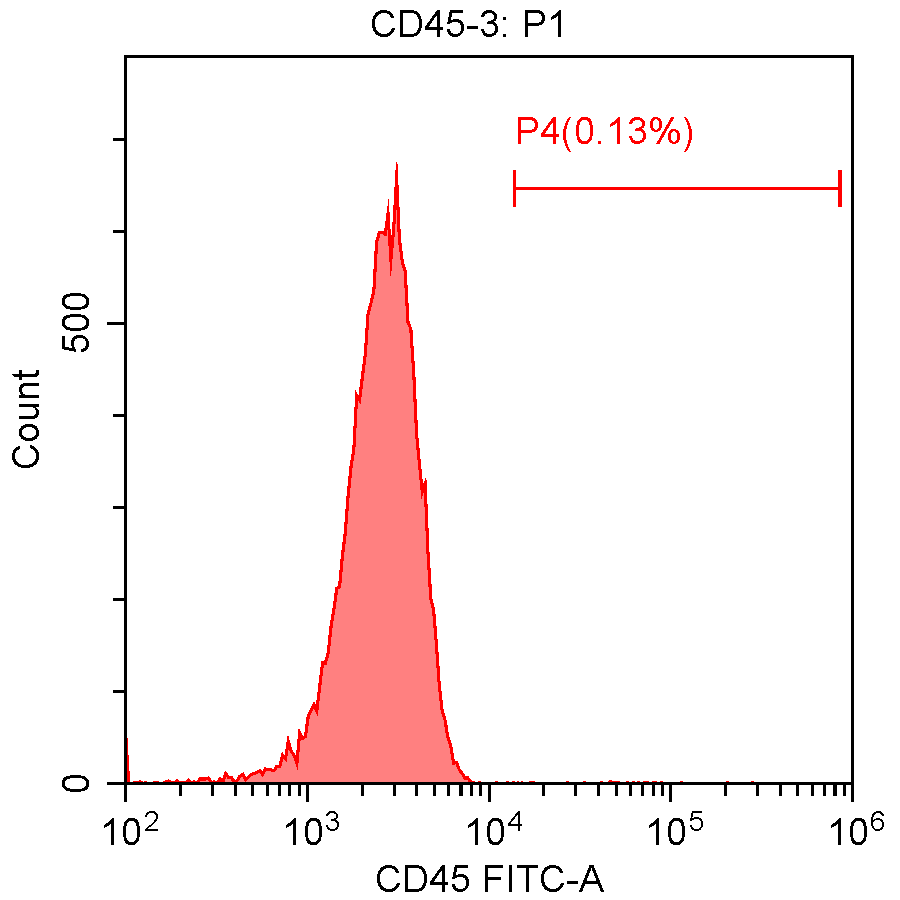

Supplement: S2 File — (ZIP) [file pone.0191616.s002.zip › Original data underlying the findings described in manuscript-Identification of MSC ,Exosomes and CSCs/MSCs identification/CD45-3_Plot1.bmp]

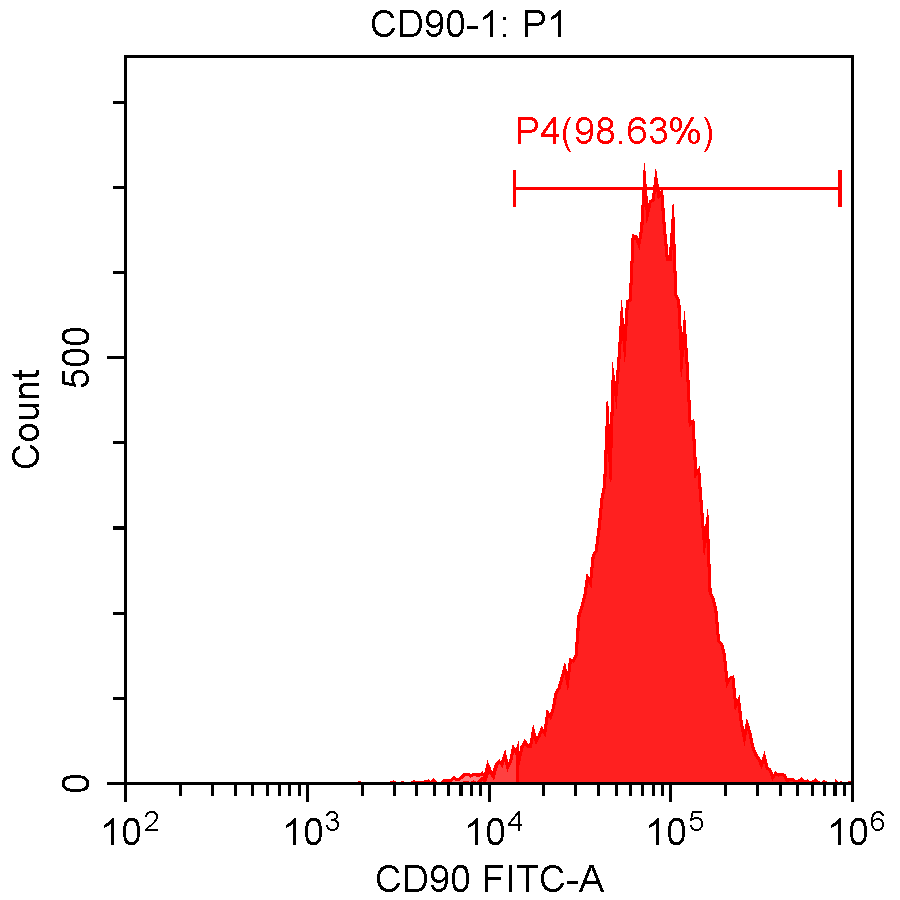

Supplement: S2 File — (ZIP) [file pone.0191616.s002.zip › Original data underlying the findings described in manuscript-Identification of MSC ,Exosomes and CSCs/MSCs identification/CD90-1_Plot1.bmp]

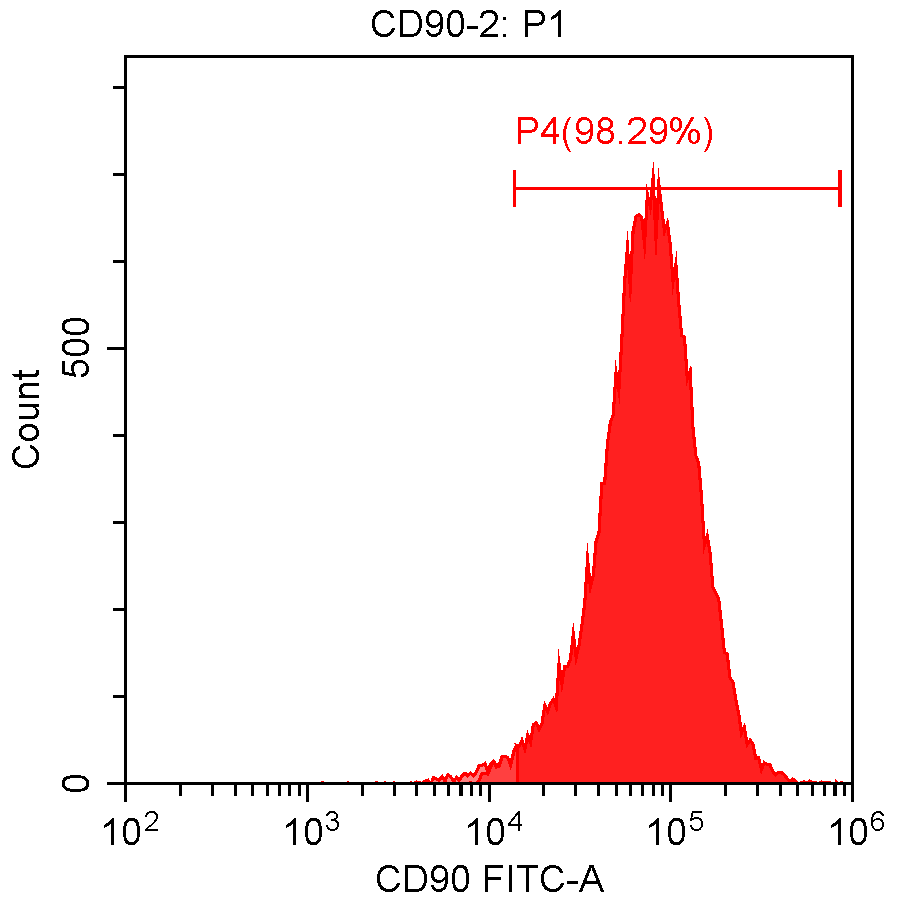

Supplement: S2 File — (ZIP) [file pone.0191616.s002.zip › Original data underlying the findings described in manuscript-Identification of MSC ,Exosomes and CSCs/MSCs identification/CD90-2_Plot1.bmp]

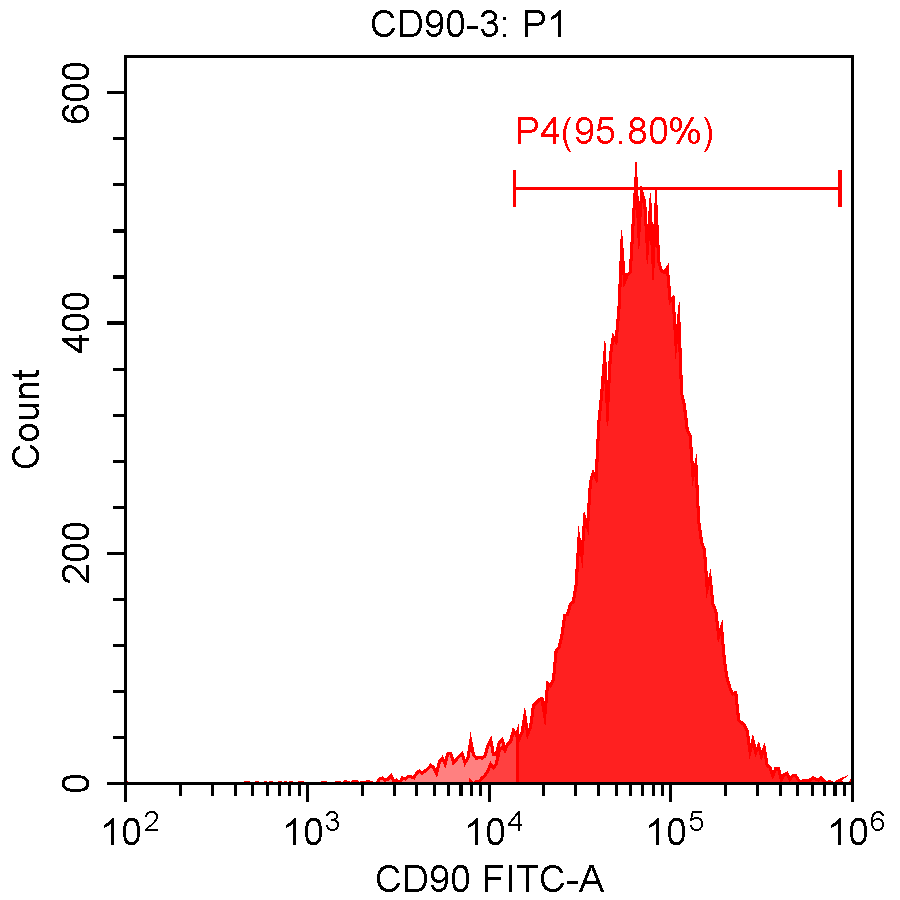

Supplement: S2 File — (ZIP) [file pone.0191616.s002.zip › Original data underlying the findings described in manuscript-Identification of MSC ,Exosomes and CSCs/MSCs identification/CD90-3_Plot1.bmp]

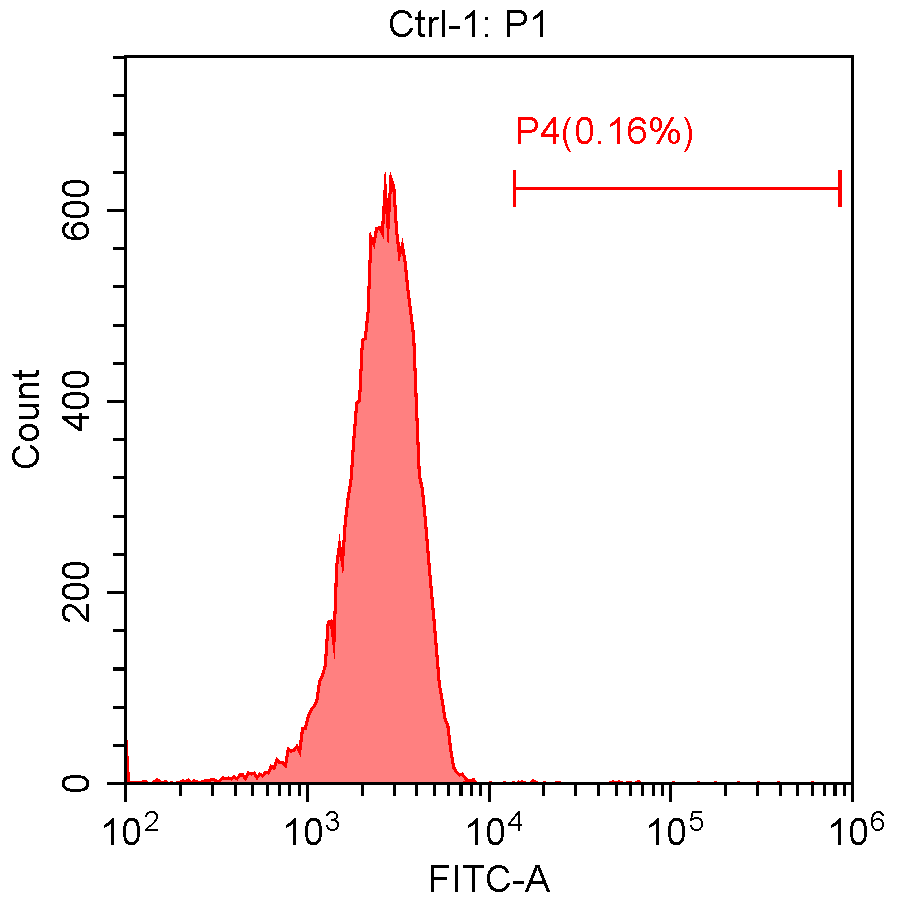

Supplement: S2 File — (ZIP) [file pone.0191616.s002.zip › Original data underlying the findings described in manuscript-Identification of MSC ,Exosomes and CSCs/MSCs identification/Ctrl-1_Plot1.bmp]

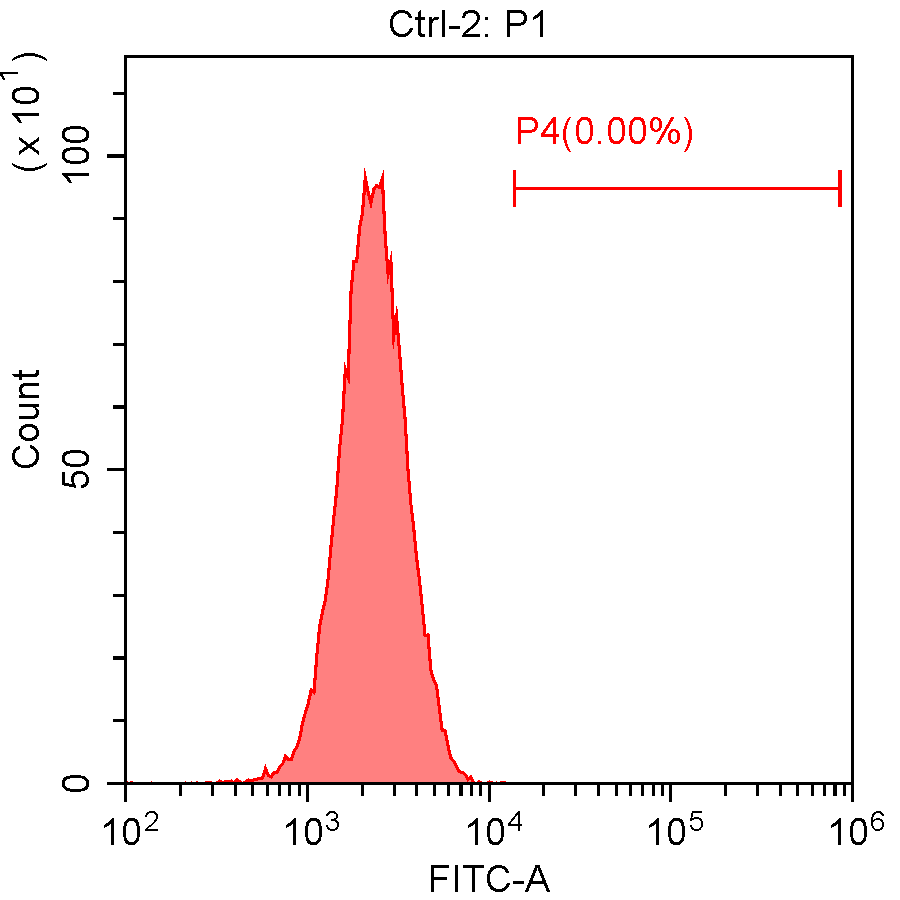

Supplement: S2 File — (ZIP) [file pone.0191616.s002.zip › Original data underlying the findings described in manuscript-Identification of MSC ,Exosomes and CSCs/MSCs identification/Ctrl-2_Plot1.bmp]

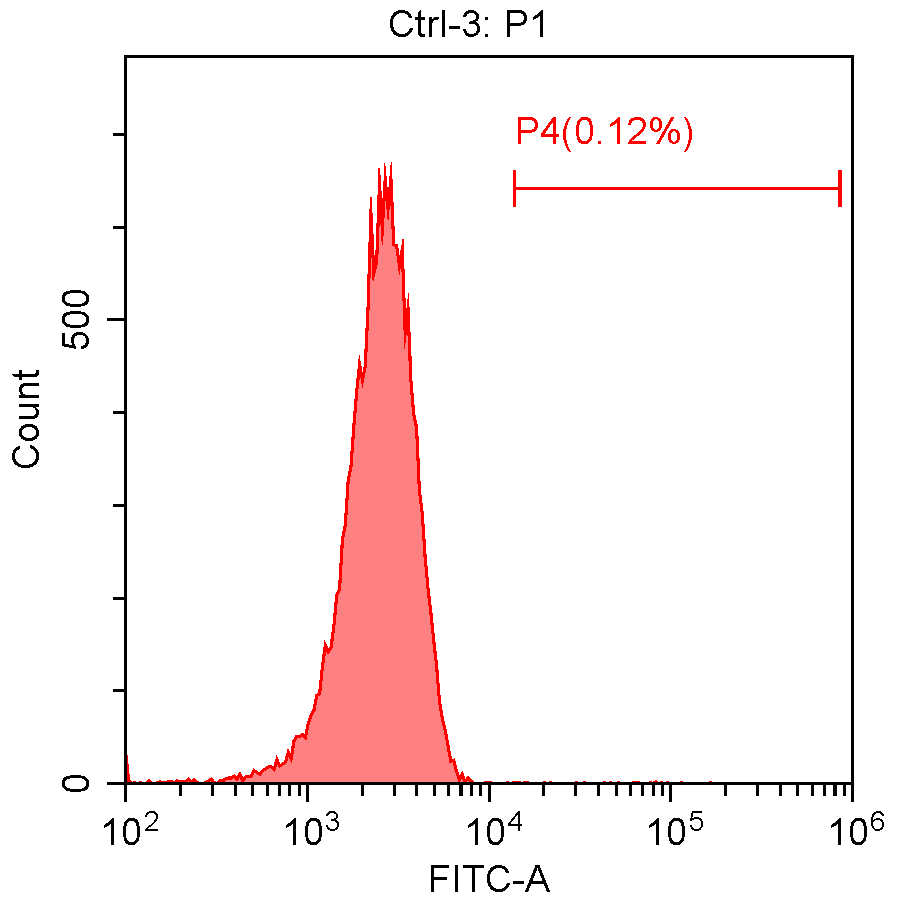

Supplement: S2 File — (ZIP) [file pone.0191616.s002.zip › Original data underlying the findings described in manuscript-Identification of MSC ,Exosomes and CSCs/MSCs identification/Ctrl-3_Plot1.bmp]

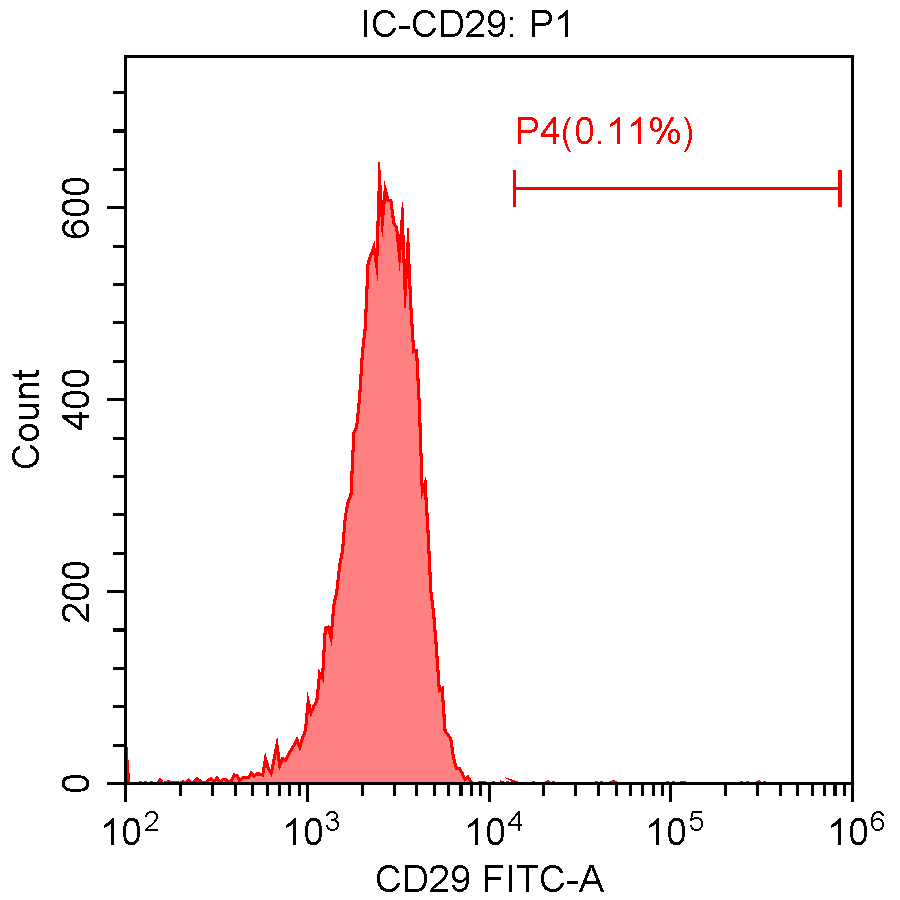

Supplement: S2 File — (ZIP) [file pone.0191616.s002.zip › Original data underlying the findings described in manuscript-Identification of MSC ,Exosomes and CSCs/MSCs identification/IC-CD29_Plot1.bmp]

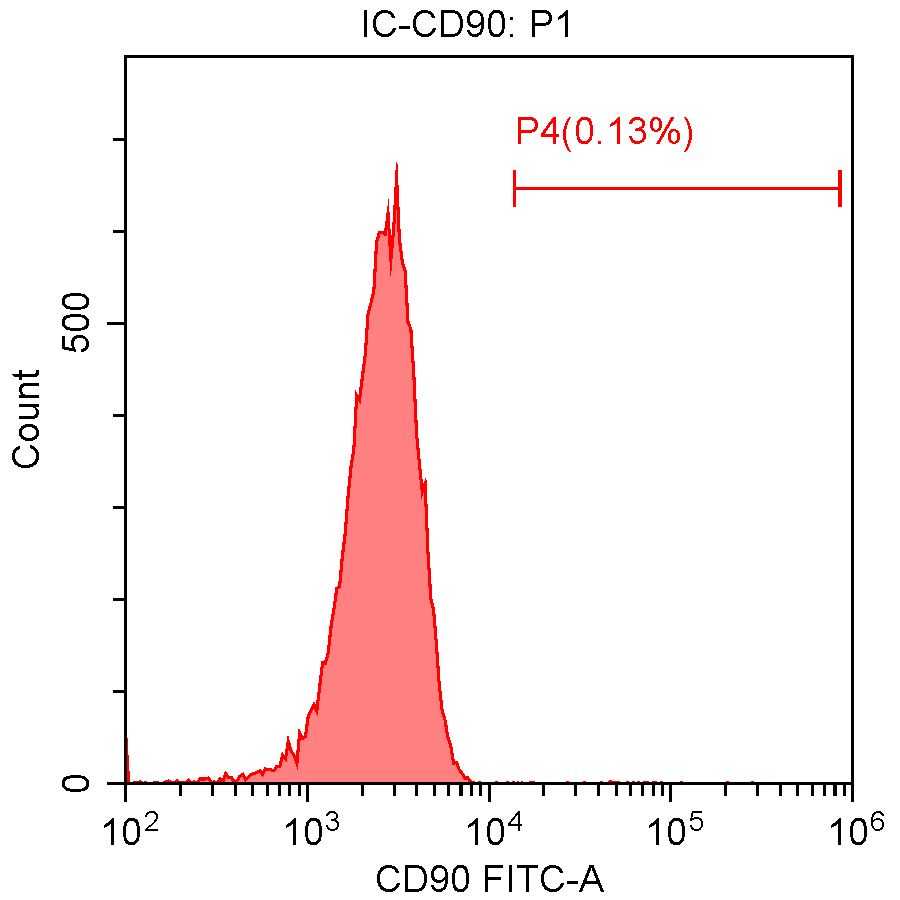

Supplement: S2 File — (ZIP) [file pone.0191616.s002.zip › Original data underlying the findings described in manuscript-Identification of MSC ,Exosomes and CSCs/MSCs identification/IC-CD90_Plot1.bmp]

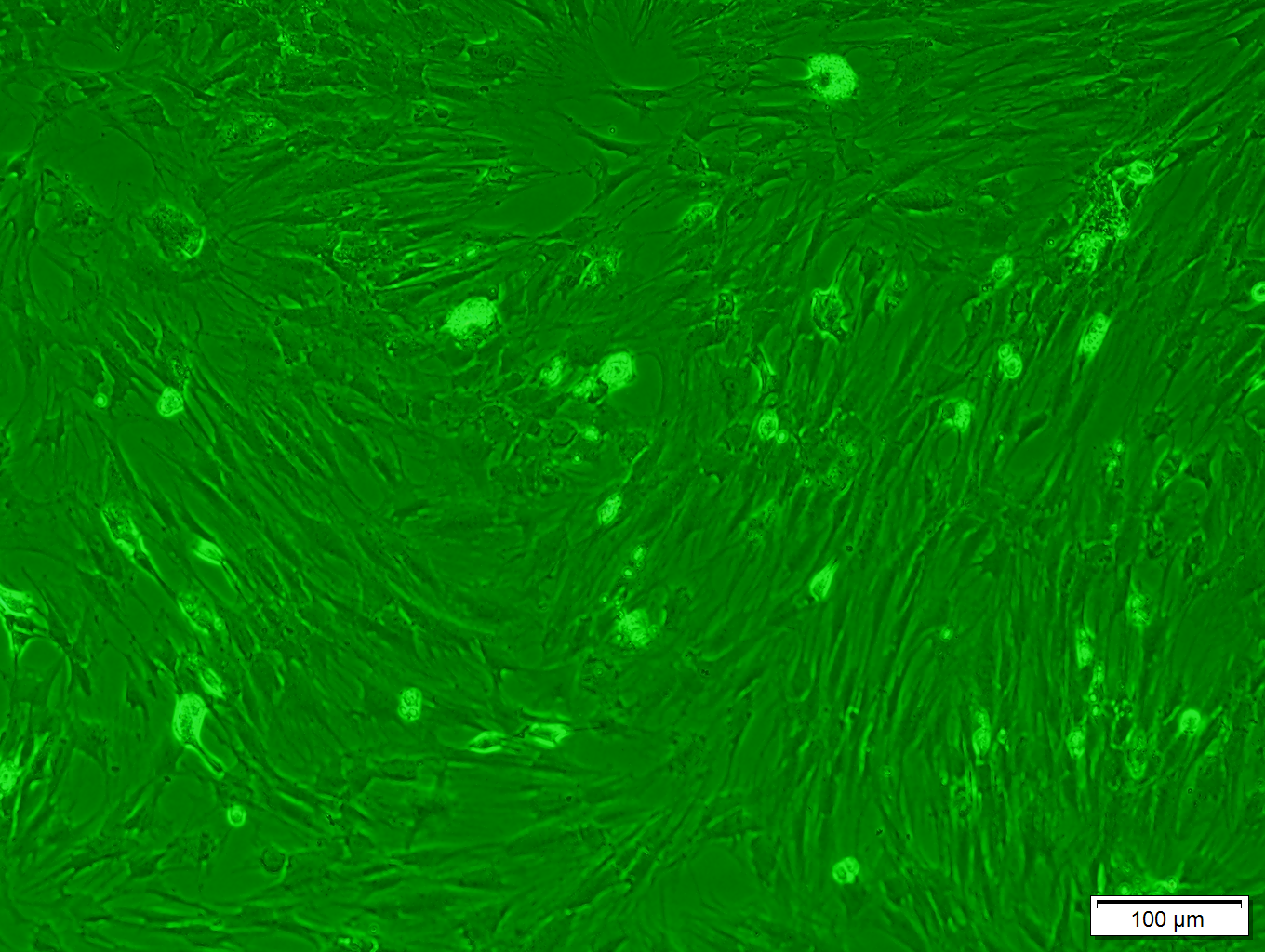

Supplement: S2 File — (ZIP) [file pone.0191616.s002.zip › Original data underlying the findings described in manuscript-Identification of MSC ,Exosomes and CSCs/MSCs.tif]

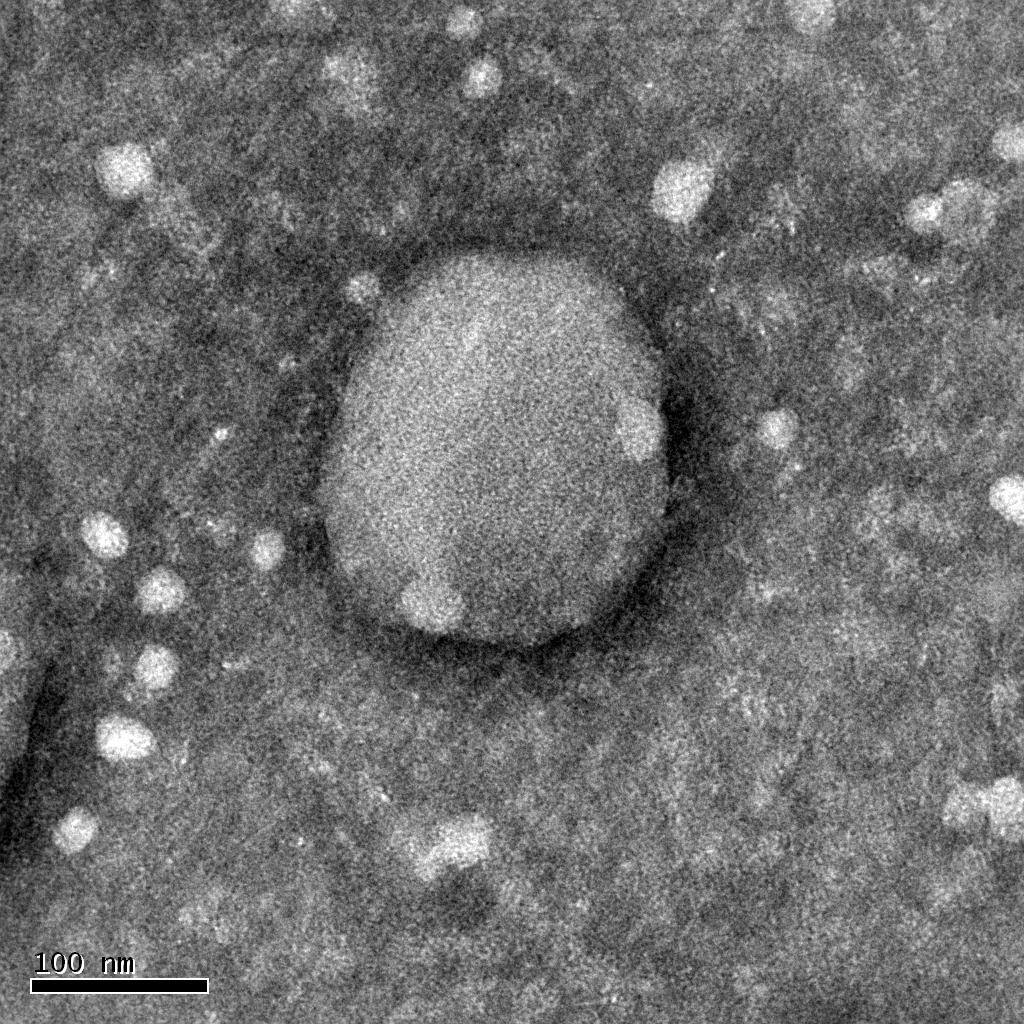

Supplement: S2 File — (ZIP) [file pone.0191616.s002.zip › Original data underlying the findings described in manuscript-Identification of MSC ,Exosomes and CSCs/Normal-exosome/200000.0V-30000X-2124.jpg]

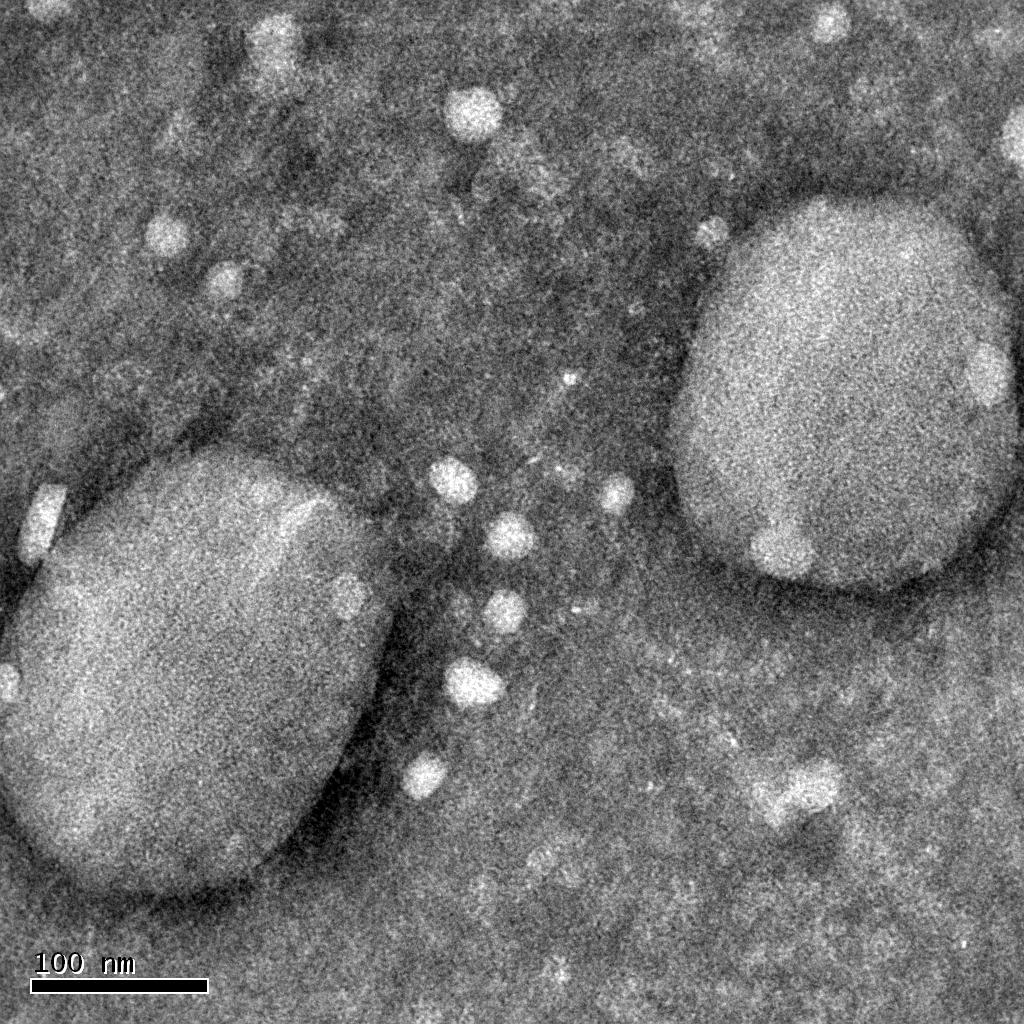

Supplement: S2 File — (ZIP) [file pone.0191616.s002.zip › Original data underlying the findings described in manuscript-Identification of MSC ,Exosomes and CSCs/Normal-exosome/200000.0V-30000X-2125.jpg]

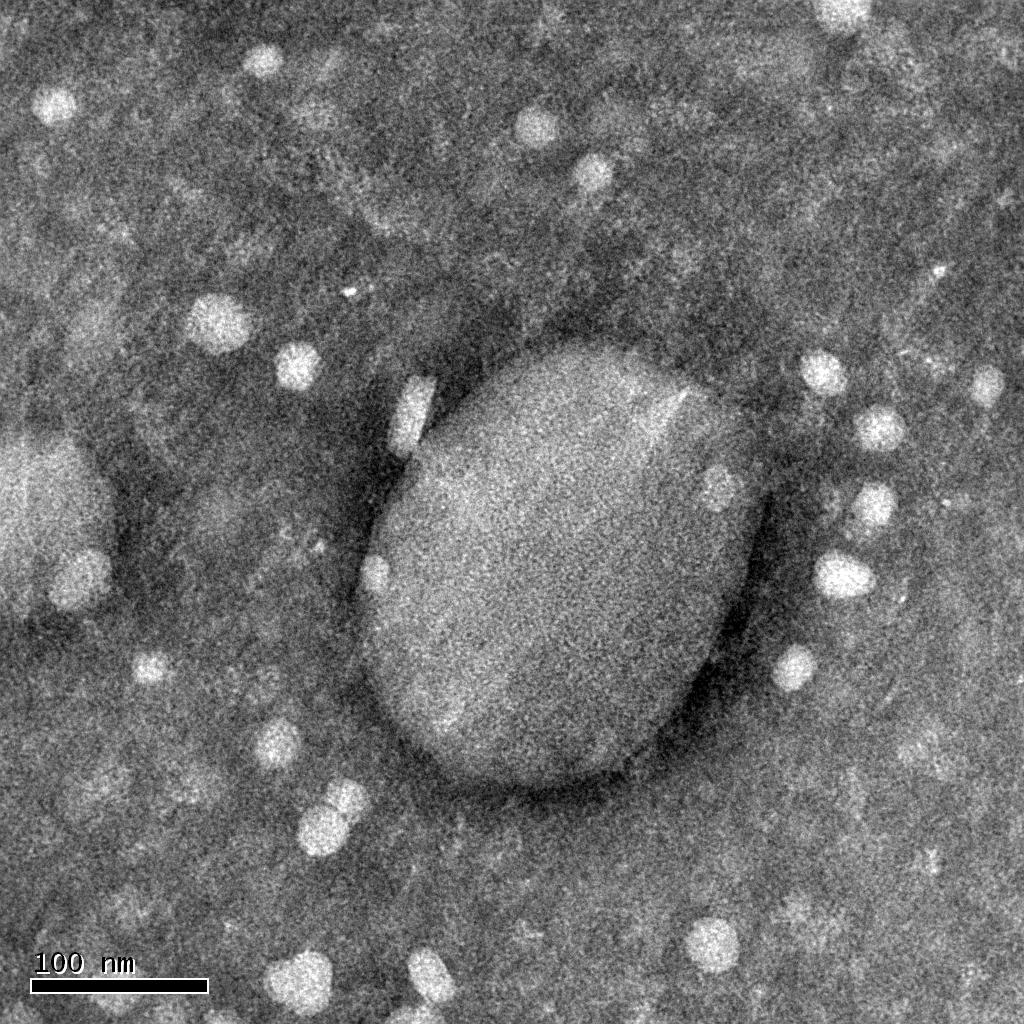

Supplement: S2 File — (ZIP) [file pone.0191616.s002.zip › Original data underlying the findings described in manuscript-Identification of MSC ,Exosomes and CSCs/Normal-exosome/200000.0V-30000X-2126.jpg]

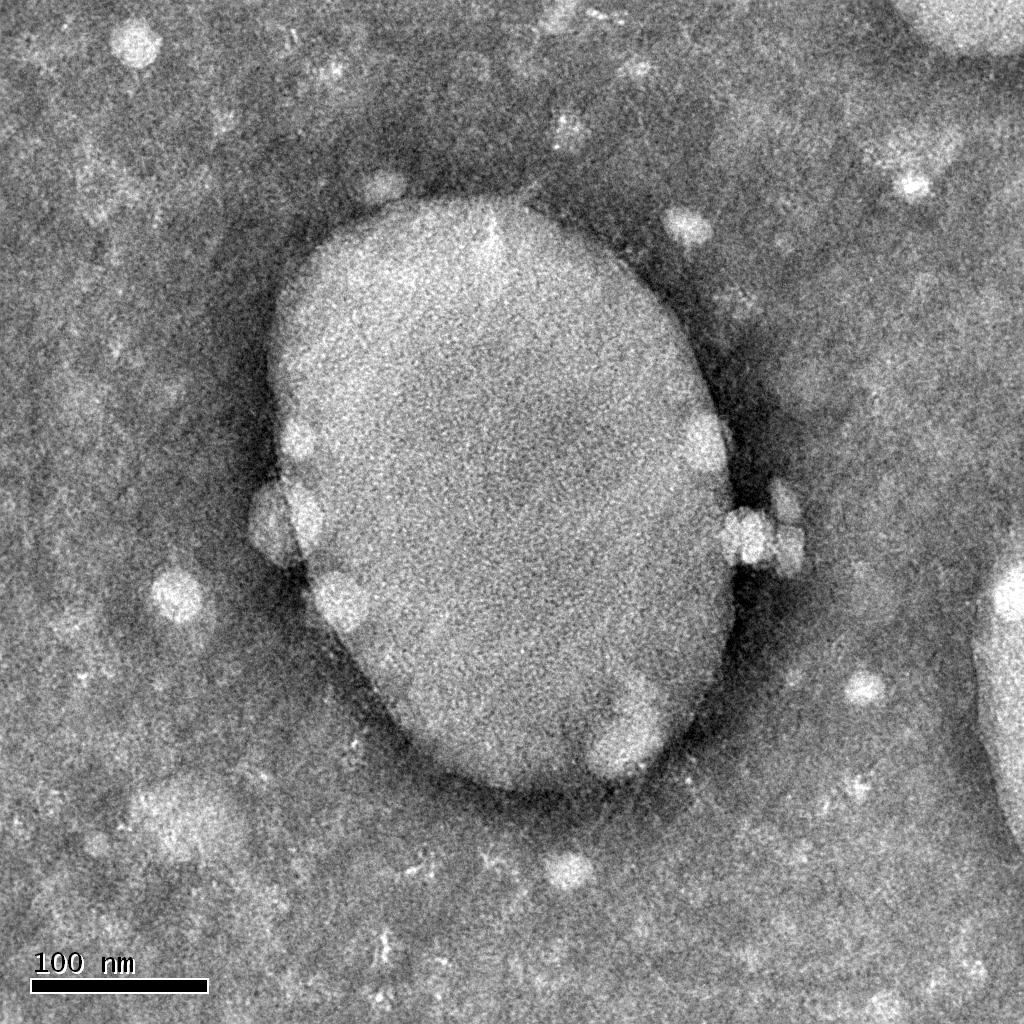

Supplement: S2 File — (ZIP) [file pone.0191616.s002.zip › Original data underlying the findings described in manuscript-Identification of MSC ,Exosomes and CSCs/Normal-exosome/200000.0V-30000X-2127.jpg]

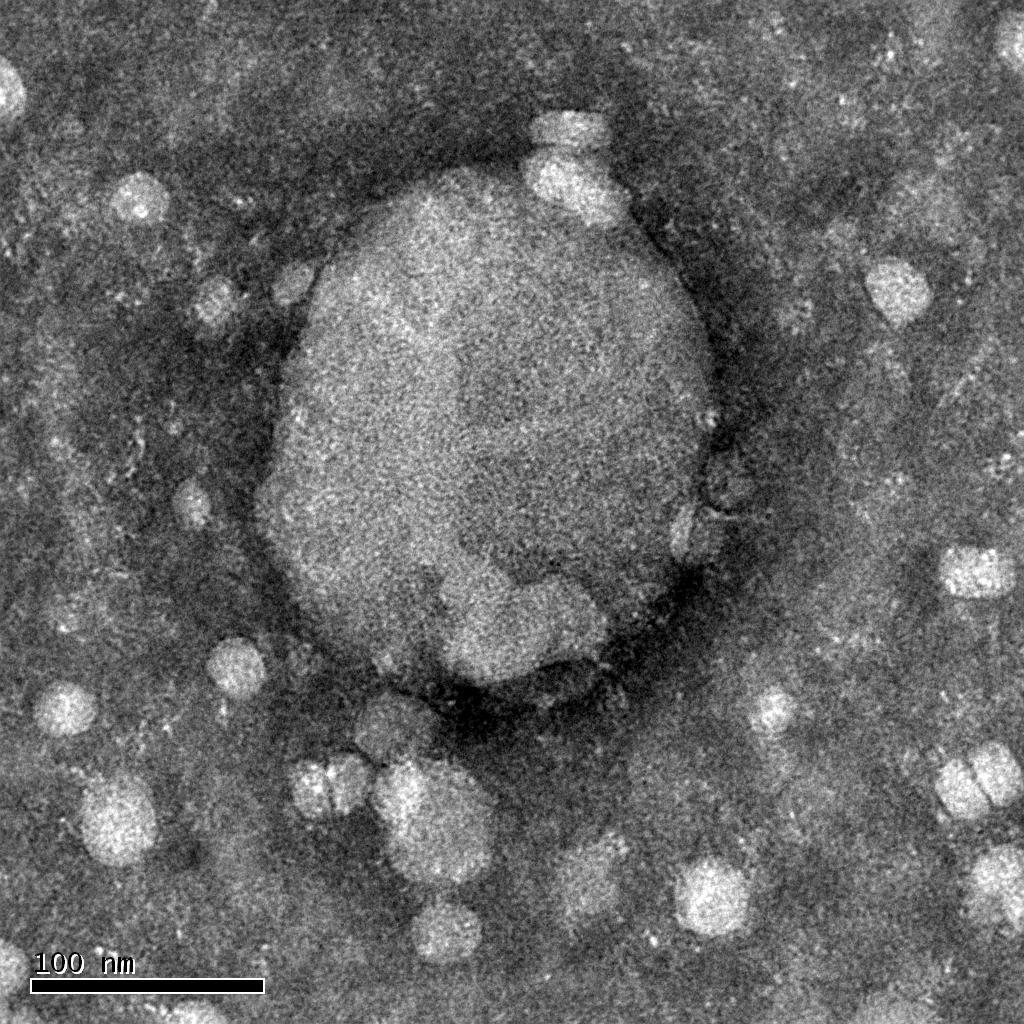

Supplement: S2 File — (ZIP) [file pone.0191616.s002.zip › Original data underlying the findings described in manuscript-Identification of MSC ,Exosomes and CSCs/Normal-exosome/200000.0V-40000X-2134.jpg]

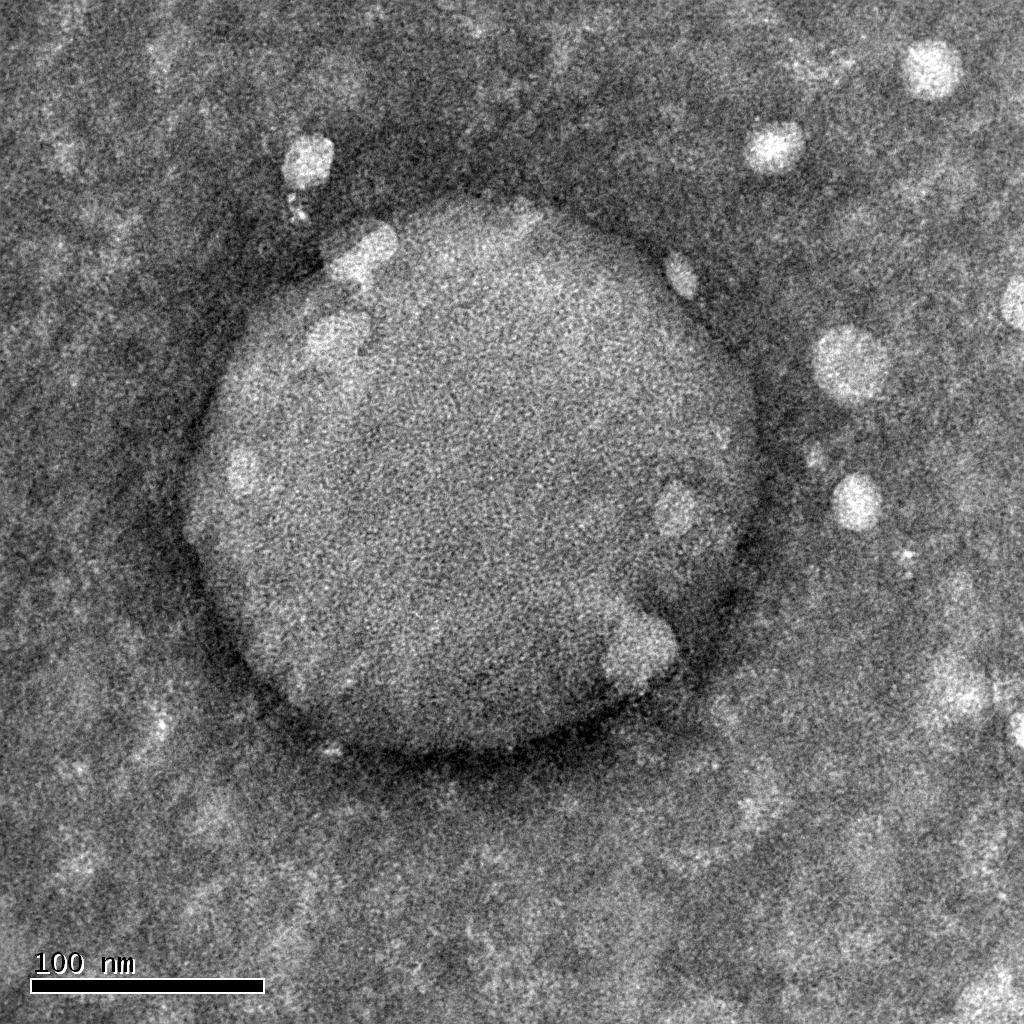

Supplement: S2 File — (ZIP) [file pone.0191616.s002.zip › Original data underlying the findings described in manuscript-Identification of MSC ,Exosomes and CSCs/Normal-exosome/200000.0V-40000X-2135.jpg]

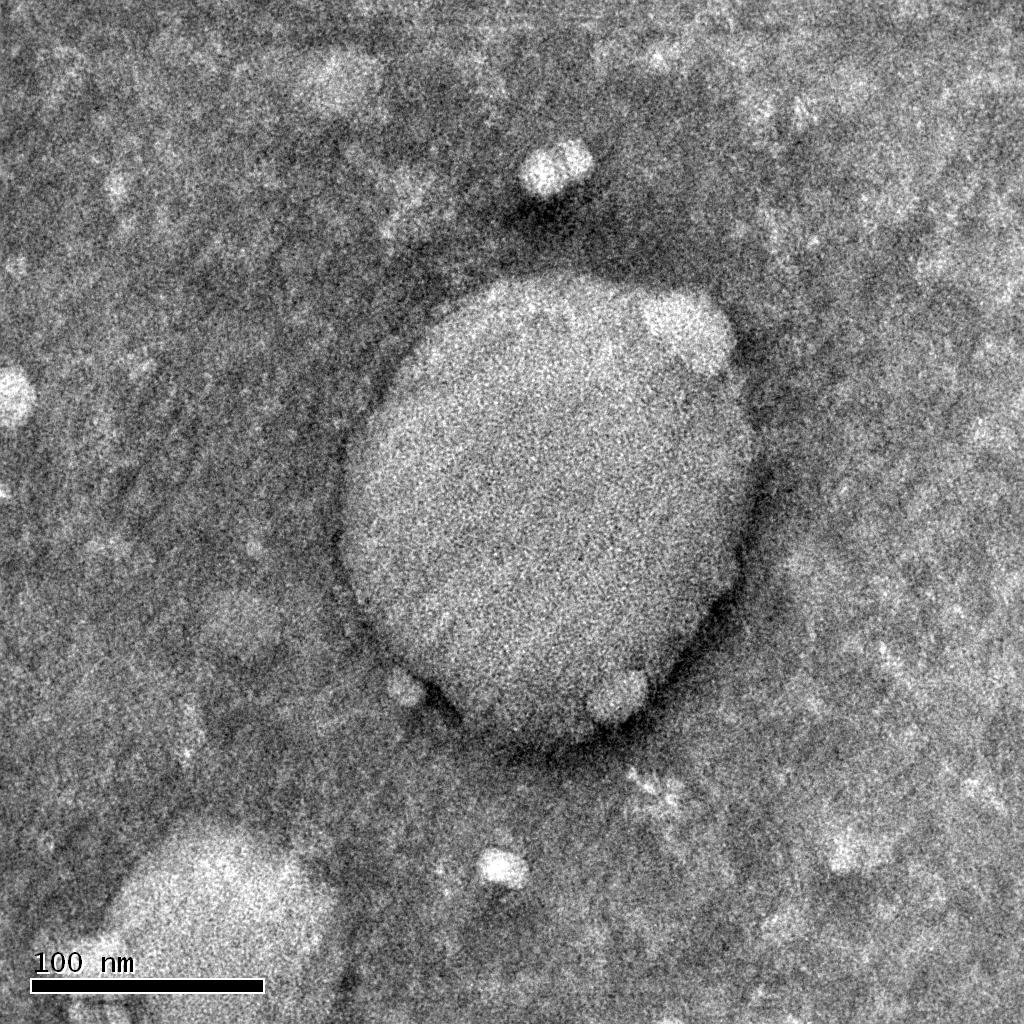

Supplement: S2 File — (ZIP) [file pone.0191616.s002.zip › Original data underlying the findings described in manuscript-Identification of MSC ,Exosomes and CSCs/Normal-exosome/200000.0V-40000X-2136.jpg]

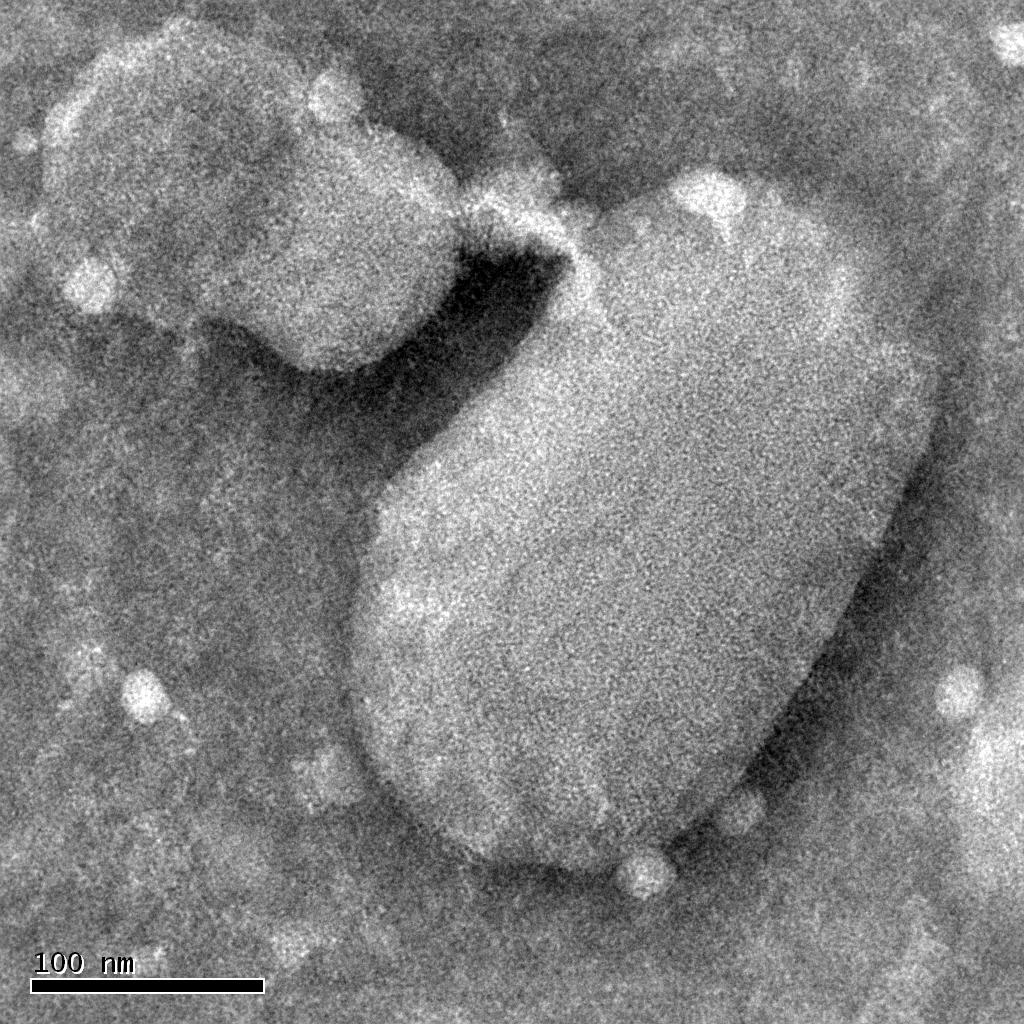

Supplement: S2 File — (ZIP) [file pone.0191616.s002.zip › Original data underlying the findings described in manuscript-Identification of MSC ,Exosomes and CSCs/Normal-exosome/200000.0V-40000X-2137.jpg]

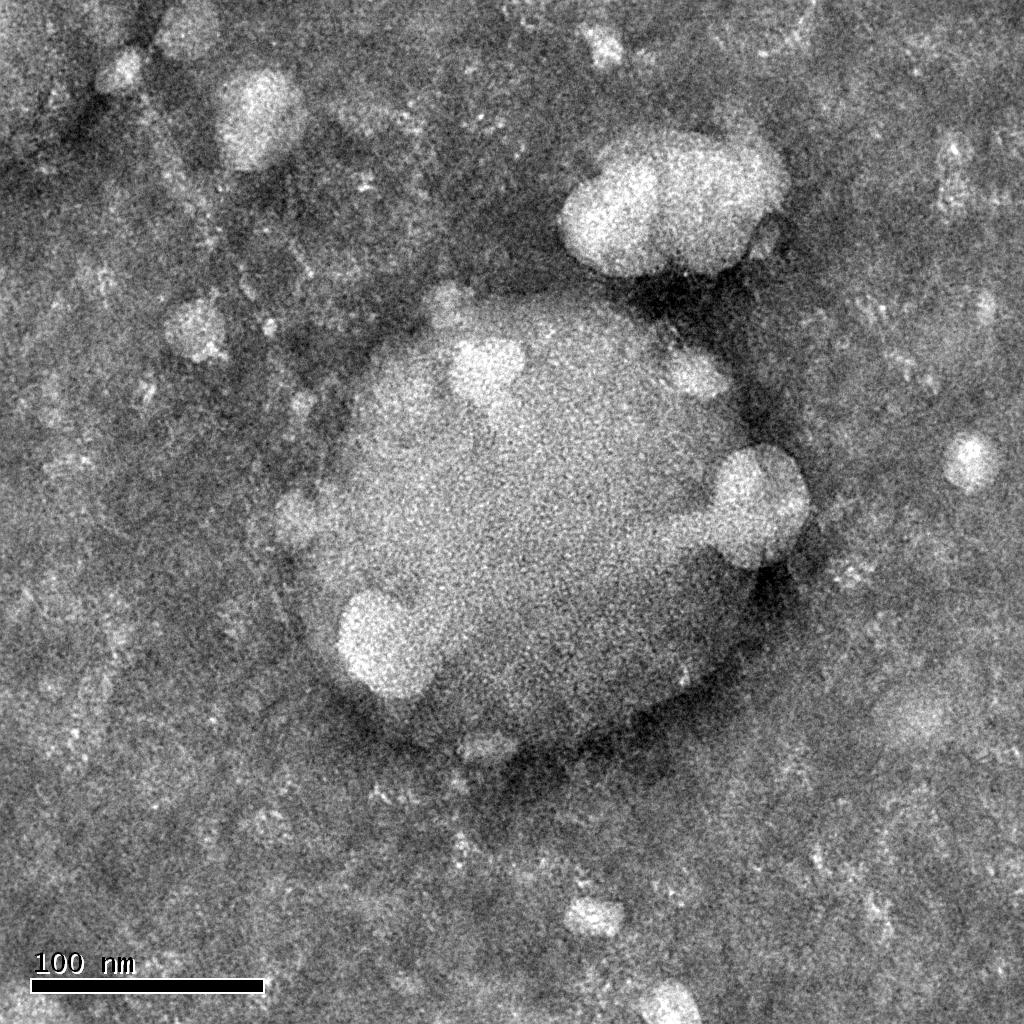

Supplement: S2 File — (ZIP) [file pone.0191616.s002.zip › Original data underlying the findings described in manuscript-Identification of MSC ,Exosomes and CSCs/Normal-exosome/200000.0V-40000X-2138.jpg]

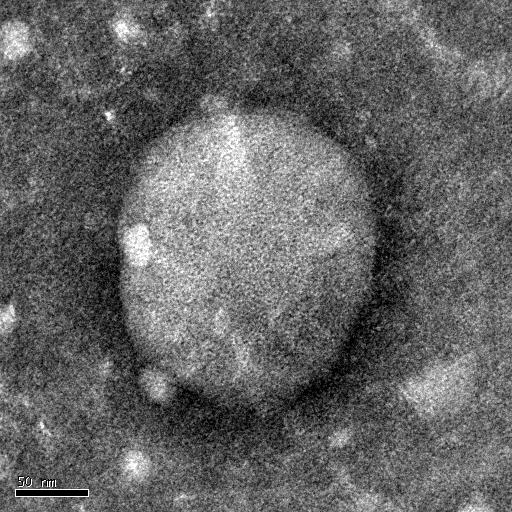

Supplement: S2 File — (ZIP) [file pone.0191616.s002.zip › Original data underlying the findings described in manuscript-Identification of MSC ,Exosomes and CSCs/Normal-exosome/200000.0V-50000X-2117.jpg]

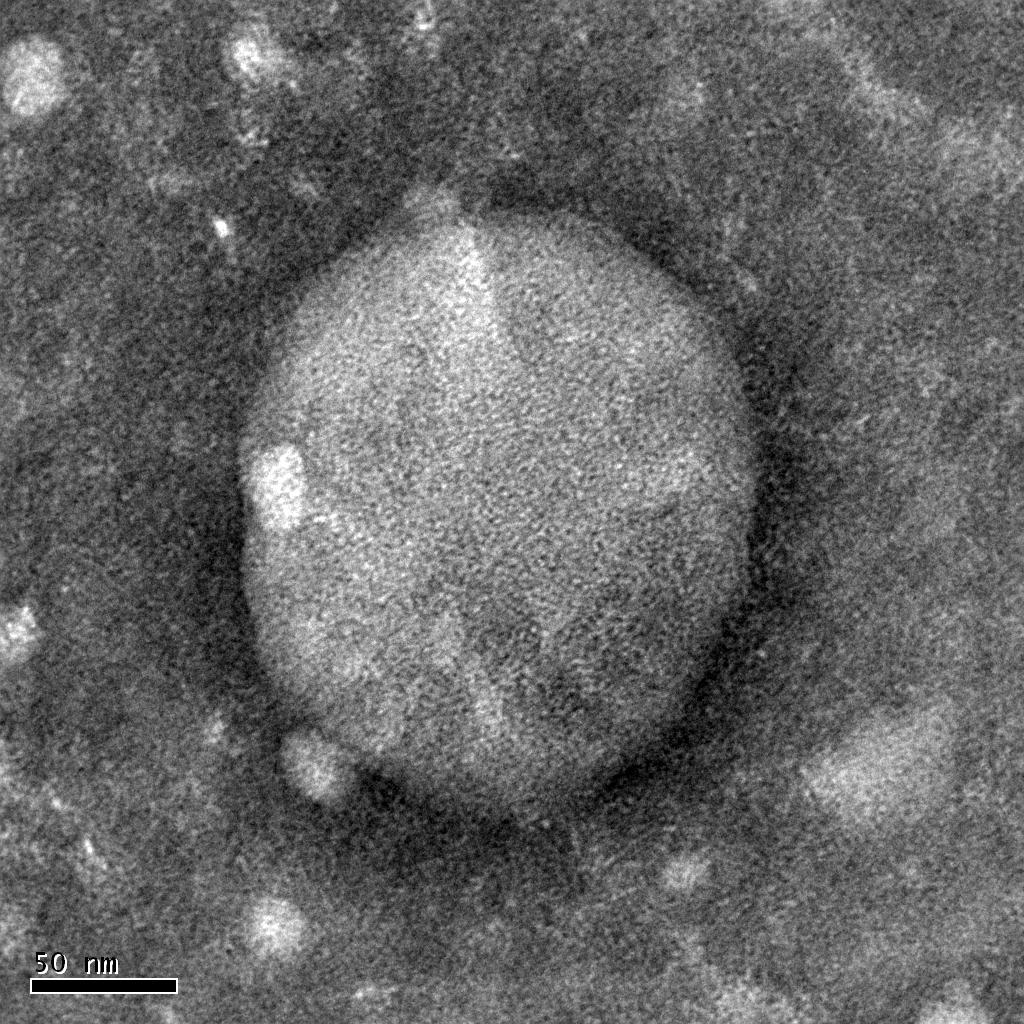

Supplement: S2 File — (ZIP) [file pone.0191616.s002.zip › Original data underlying the findings described in manuscript-Identification of MSC ,Exosomes and CSCs/Normal-exosome/200000.0V-50000X-2118.jpg]

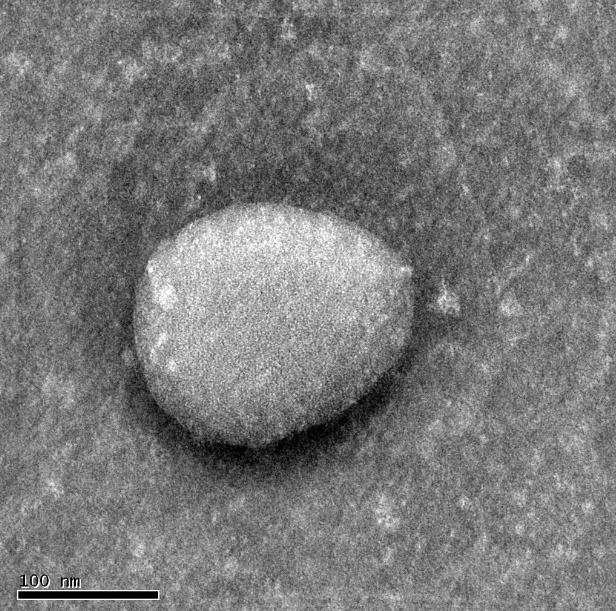

Supplement: S2 File — (ZIP) [file pone.0191616.s002.zip › Original data underlying the findings described in manuscript-Identification of MSC ,Exosomes and CSCs/exosom-1.png]

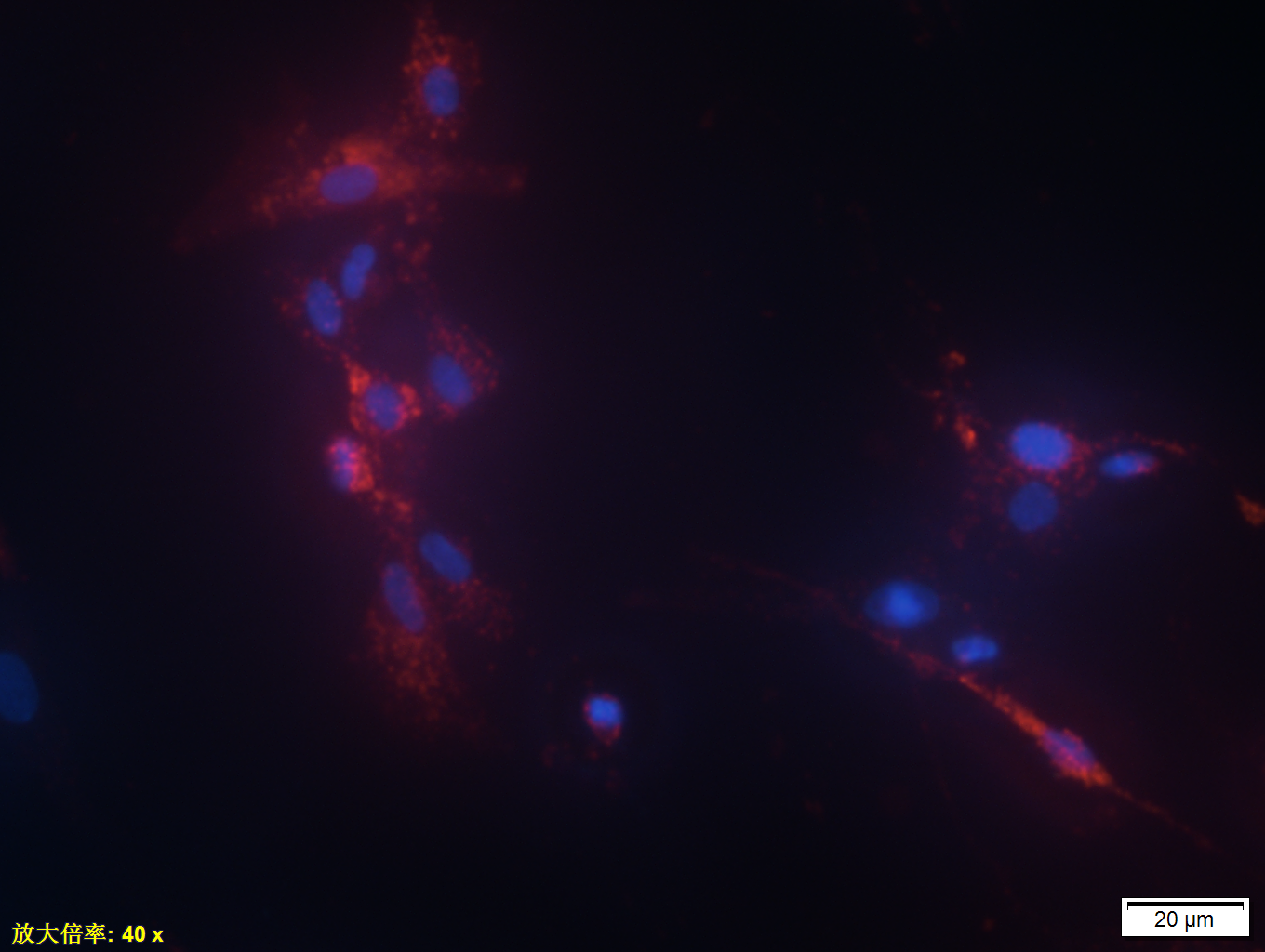

Supplement: S3 File — (ZIP) [file pone.0191616.s003.zip › Original data underlying the findings described in manuscript-Internalization of DiI-Labeled exosomes into CSCs/A40 (1).tif]

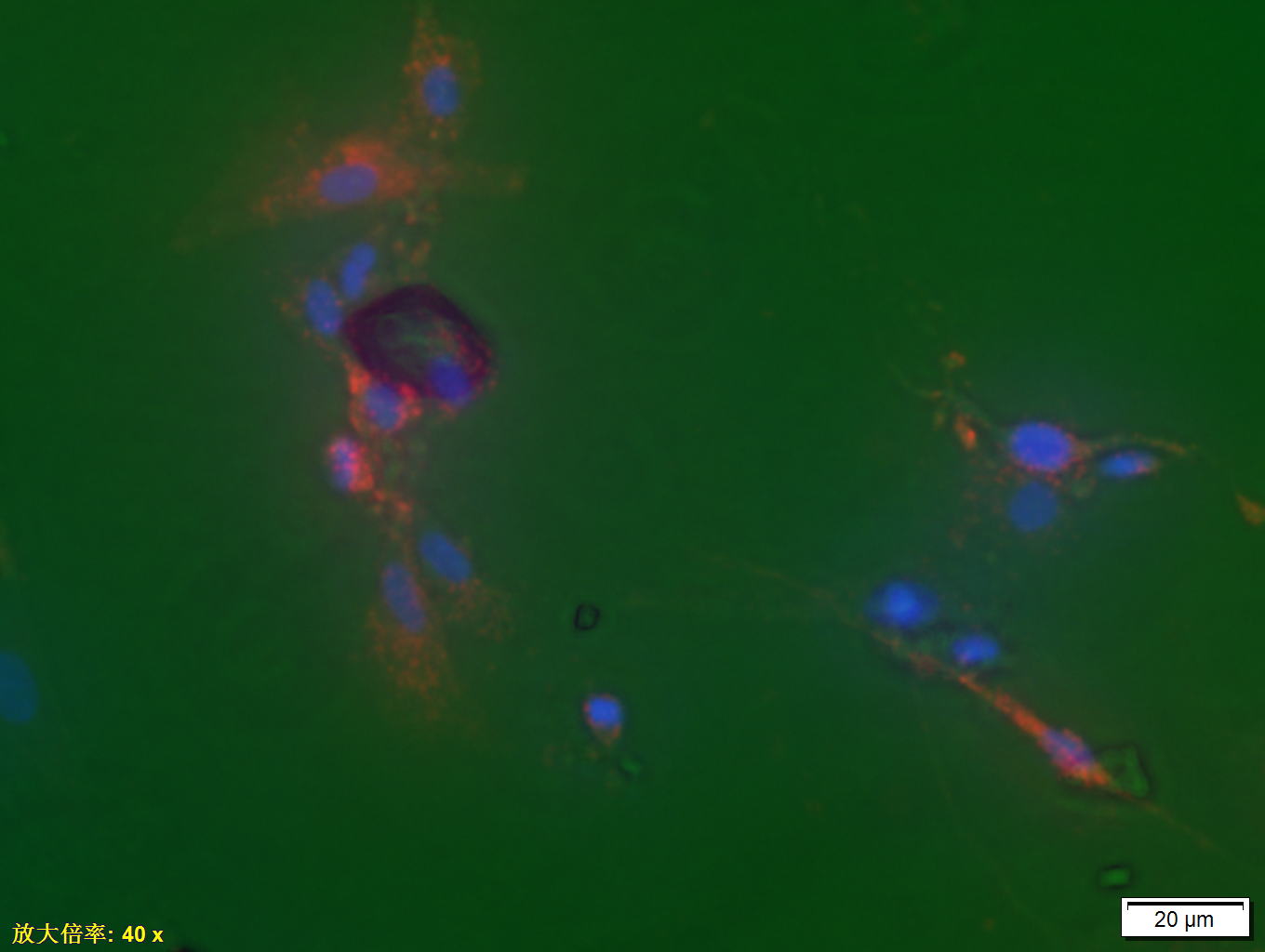

Supplement: S3 File — (ZIP) [file pone.0191616.s003.zip › Original data underlying the findings described in manuscript-Internalization of DiI-Labeled exosomes into CSCs/A40 (2).tif]

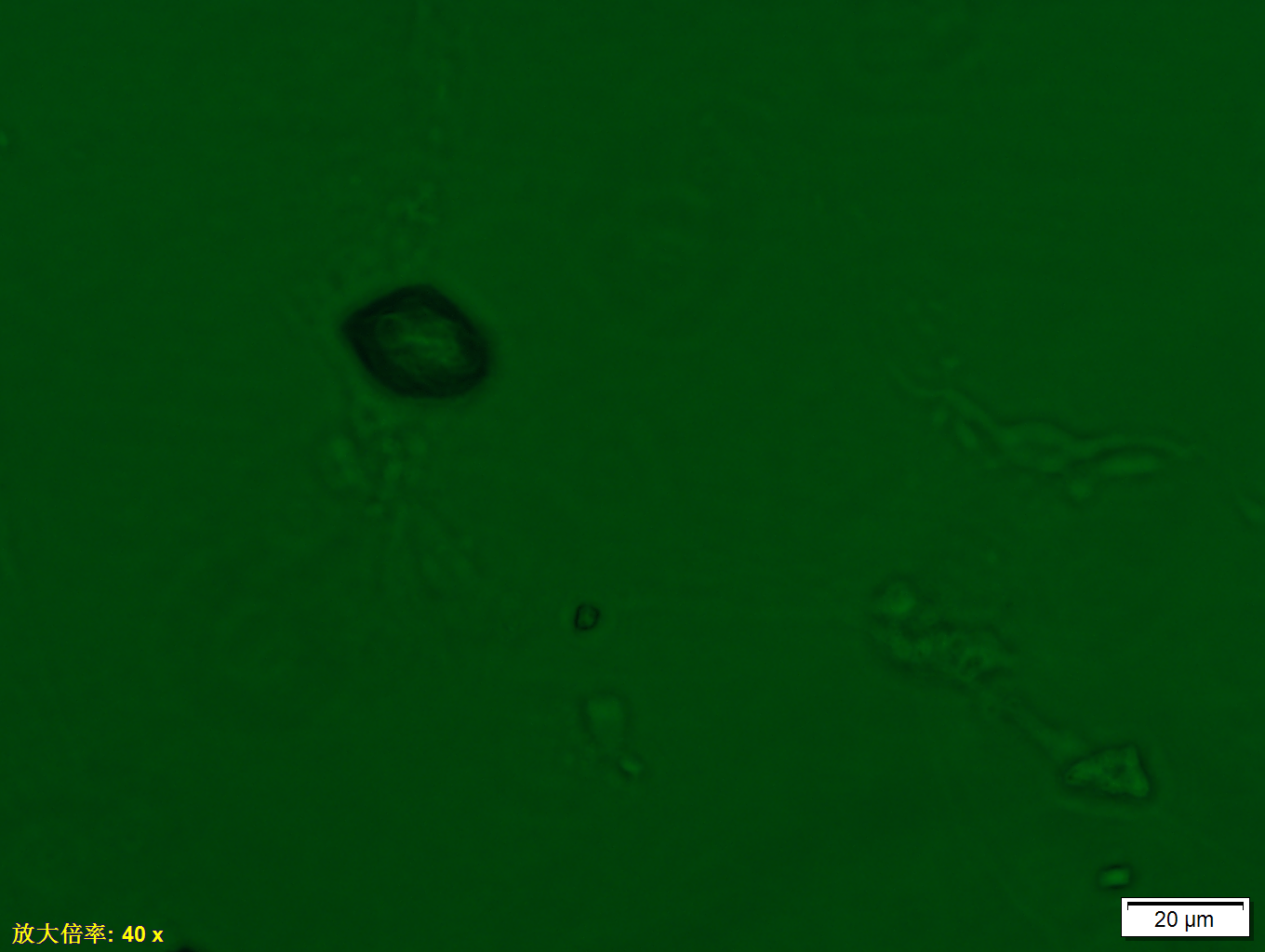

Supplement: S3 File — (ZIP) [file pone.0191616.s003.zip › Original data underlying the findings described in manuscript-Internalization of DiI-Labeled exosomes into CSCs/A40 (3).tif]

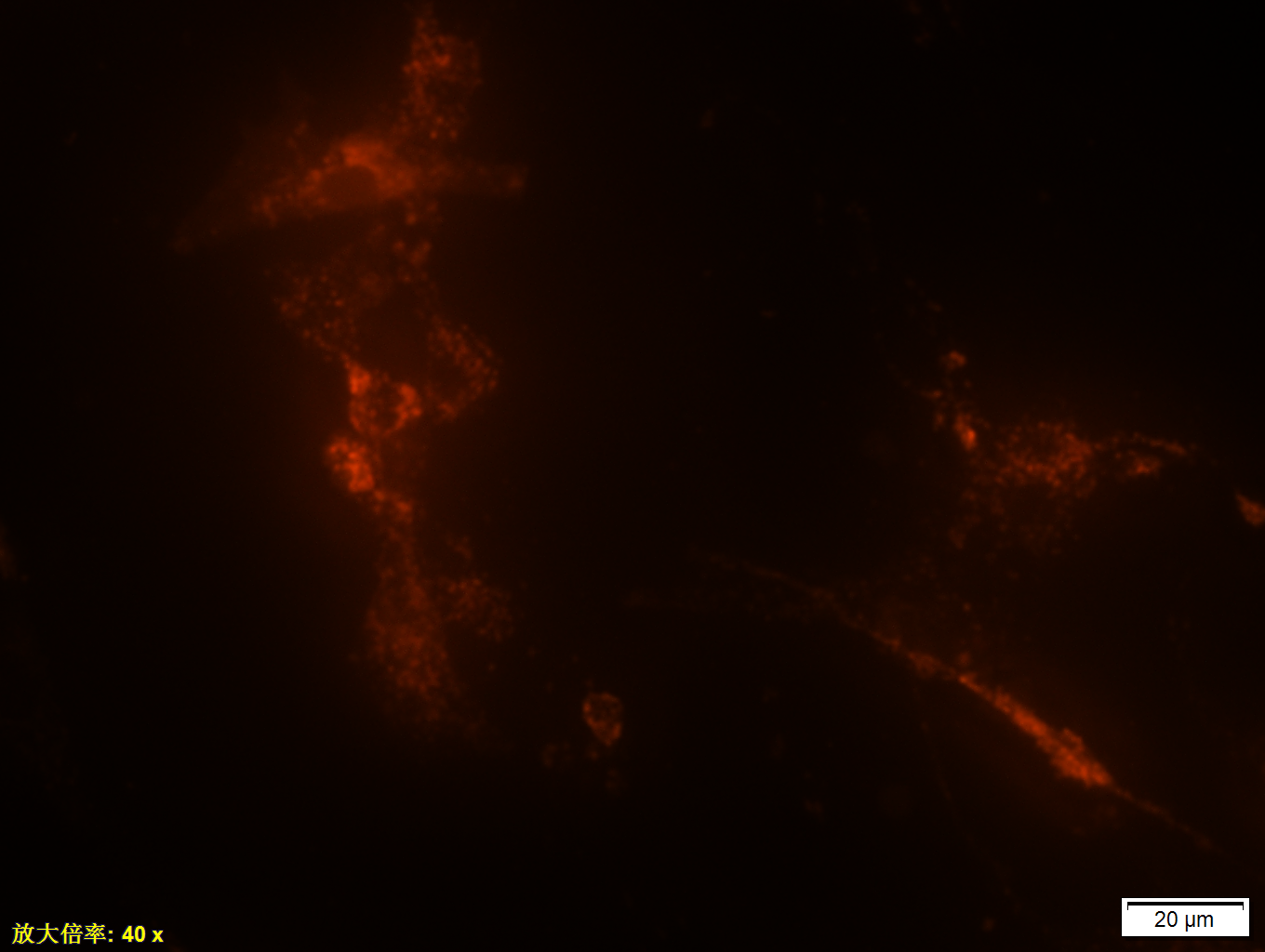

Supplement: S3 File — (ZIP) [file pone.0191616.s003.zip › Original data underlying the findings described in manuscript-Internalization of DiI-Labeled exosomes into CSCs/A40 (4).tif]

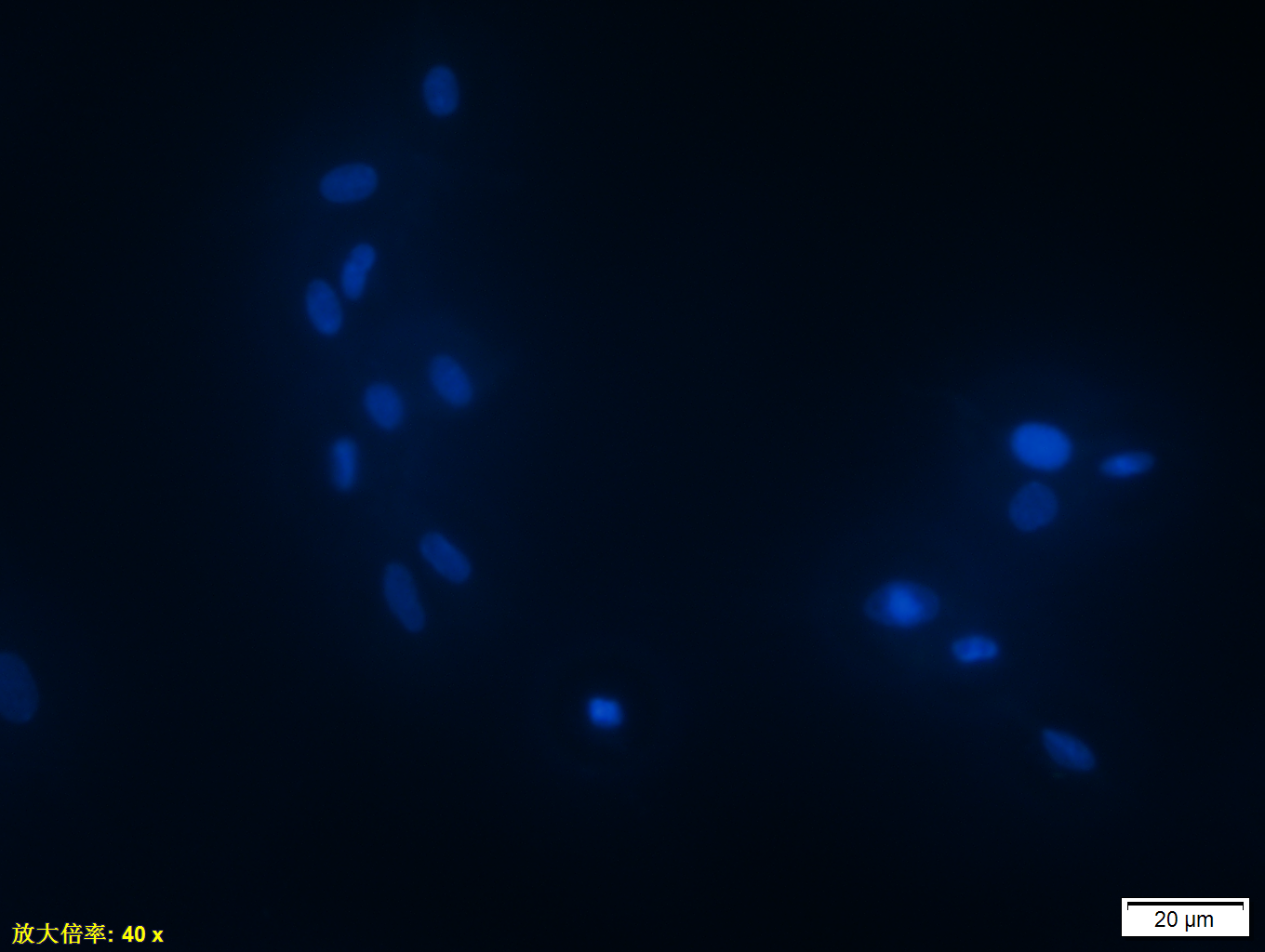

Supplement: S3 File — (ZIP) [file pone.0191616.s003.zip › Original data underlying the findings described in manuscript-Internalization of DiI-Labeled exosomes into CSCs/A40 (5).tif]

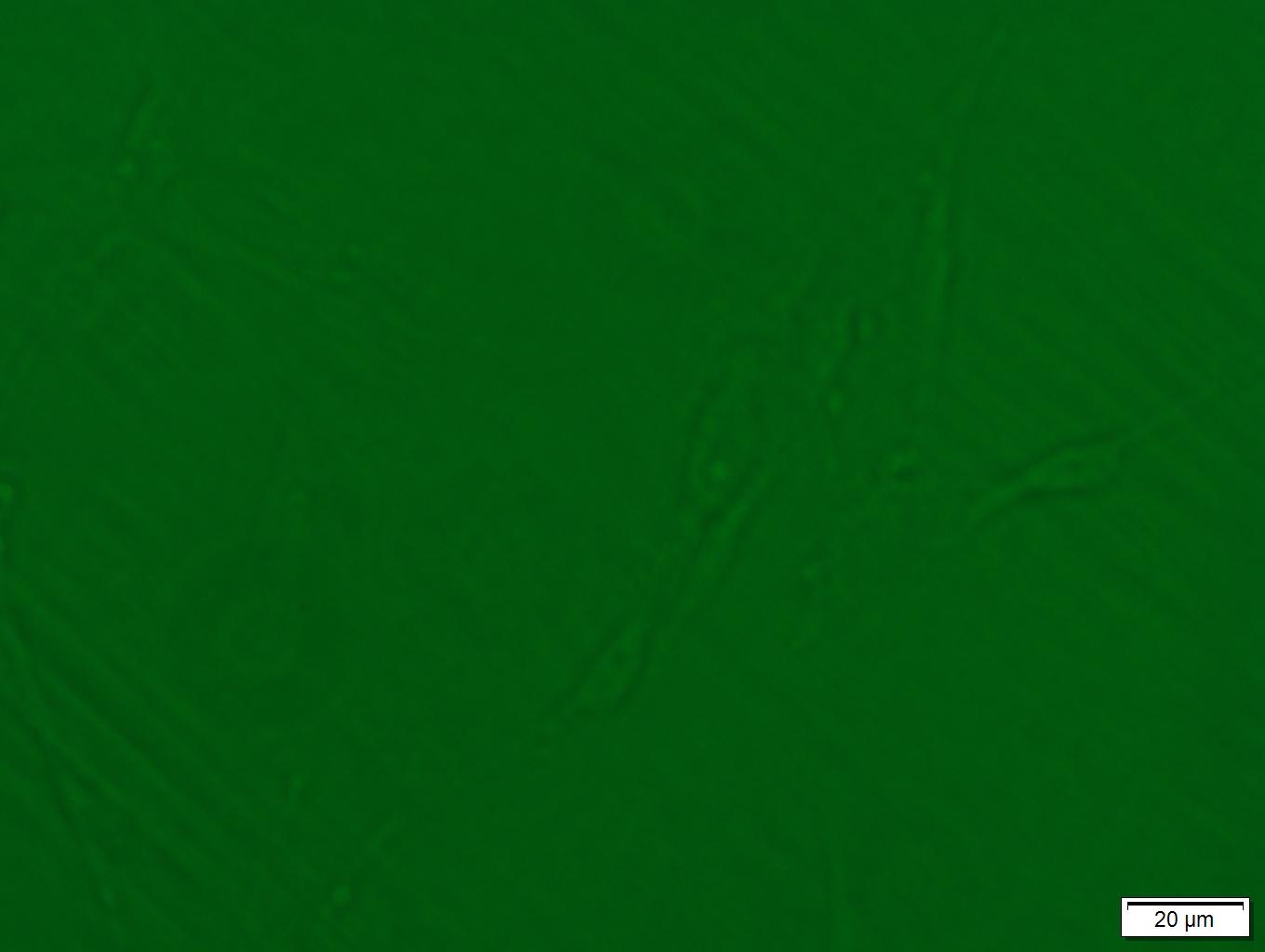

Supplement: S3 File — (ZIP) [file pone.0191616.s003.zip › Original data underlying the findings described in manuscript-Internalization of DiI-Labeled exosomes into CSCs/A41 (1).tif]

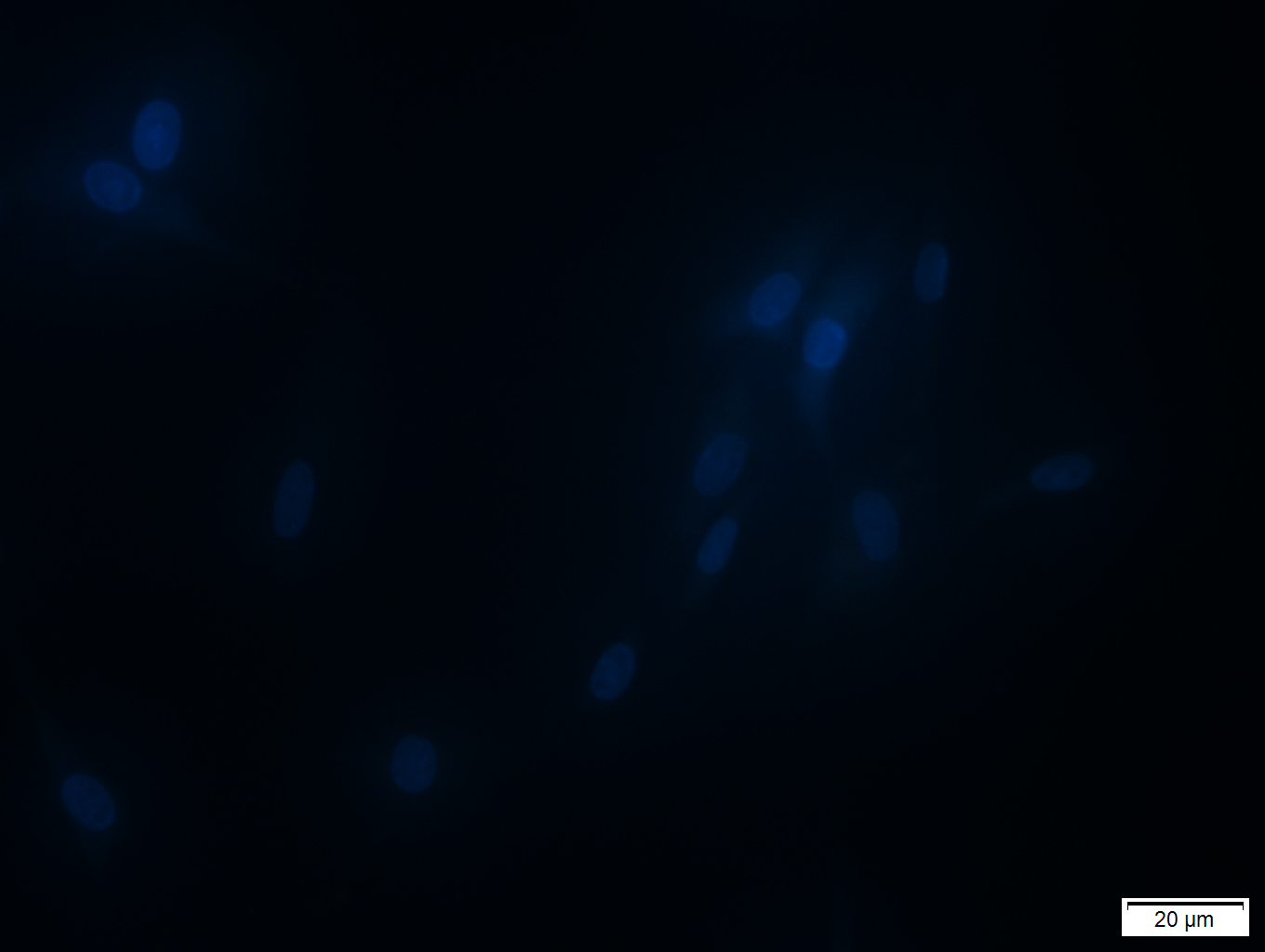

Supplement: S3 File — (ZIP) [file pone.0191616.s003.zip › Original data underlying the findings described in manuscript-Internalization of DiI-Labeled exosomes into CSCs/A41 (2).tif]

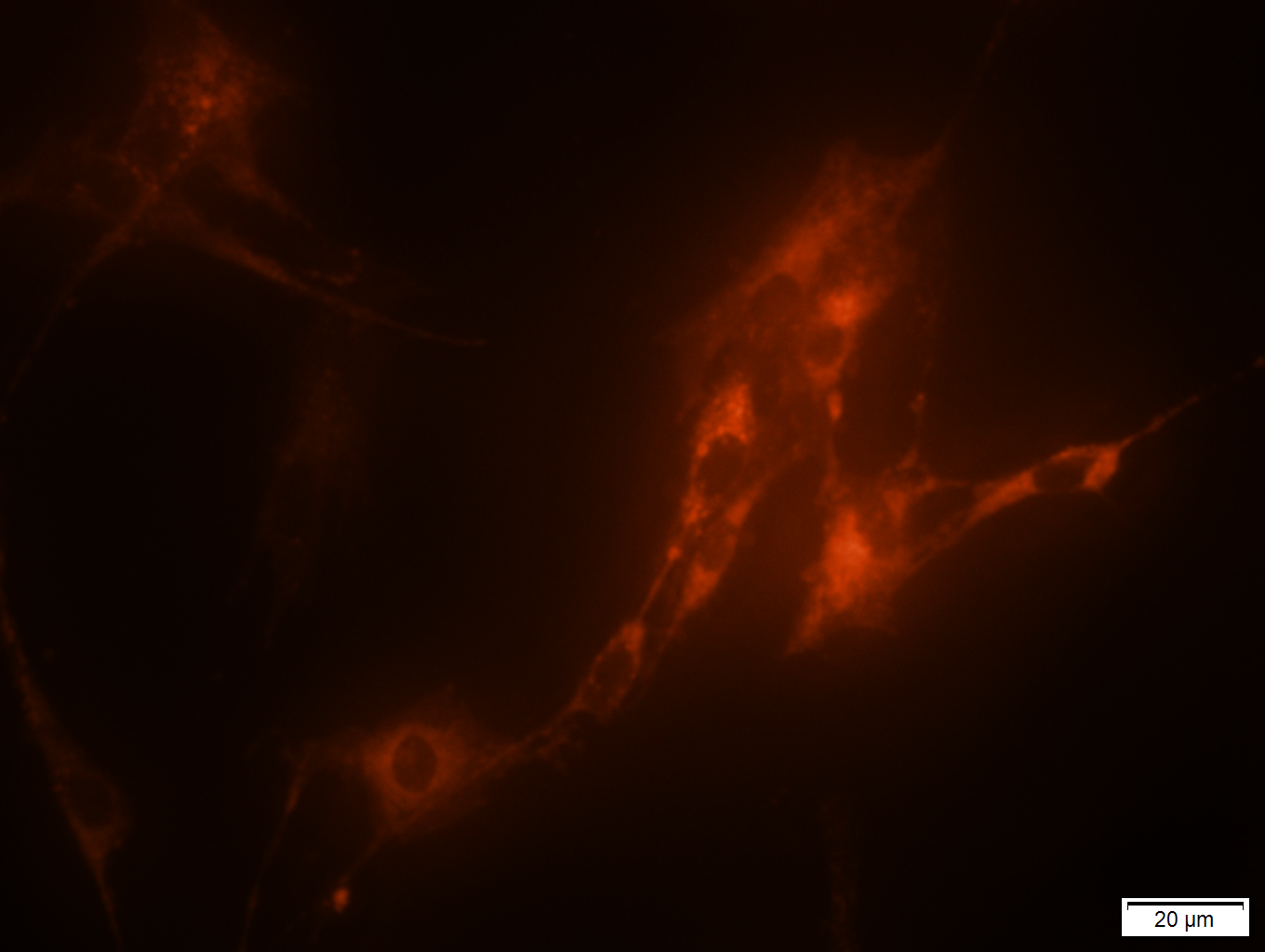

Supplement: S3 File — (ZIP) [file pone.0191616.s003.zip › Original data underlying the findings described in manuscript-Internalization of DiI-Labeled exosomes into CSCs/A41 (3).tif]

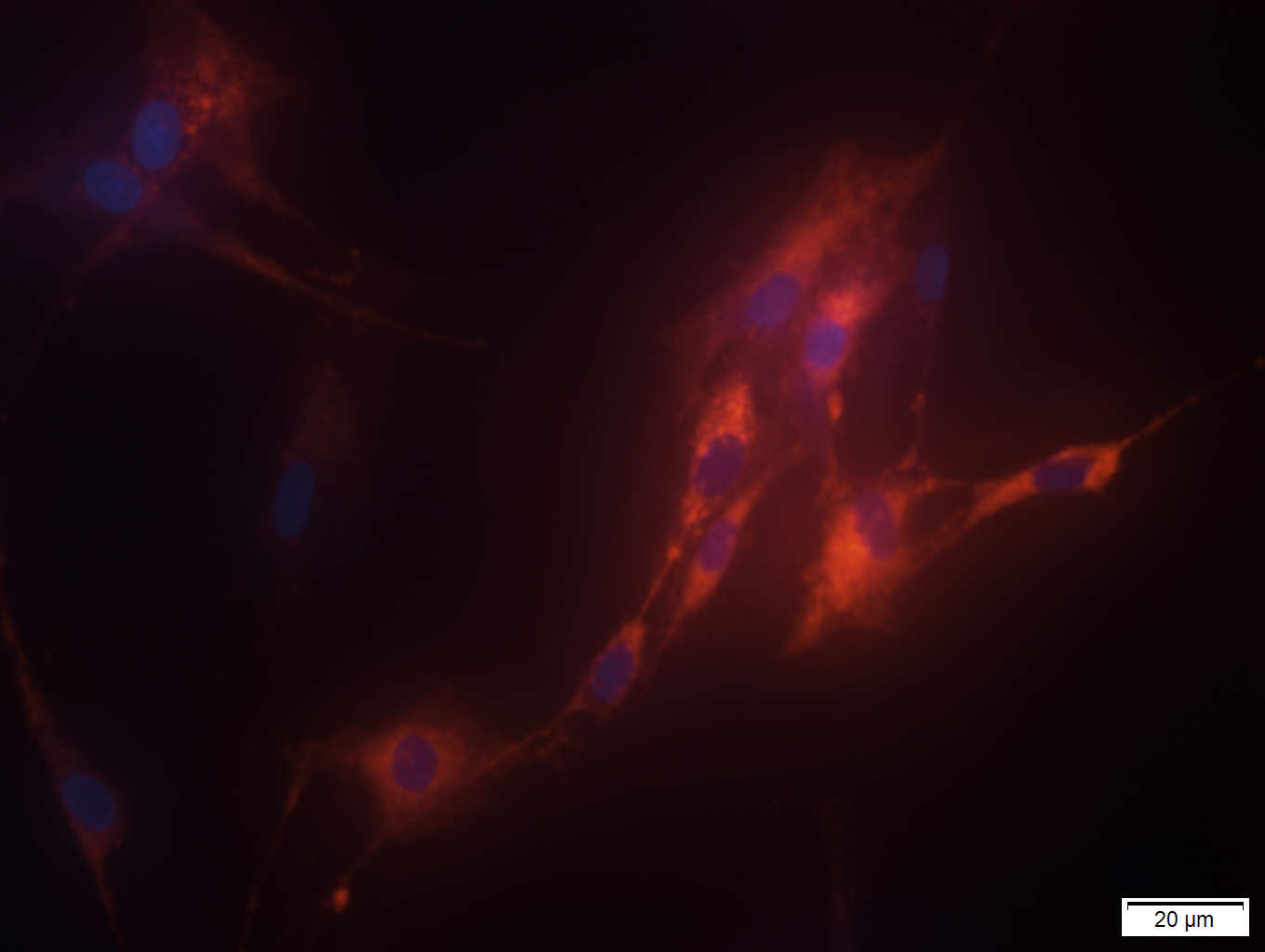

Supplement: S3 File — (ZIP) [file pone.0191616.s003.zip › Original data underlying the findings described in manuscript-Internalization of DiI-Labeled exosomes into CSCs/A41 (4).tif]

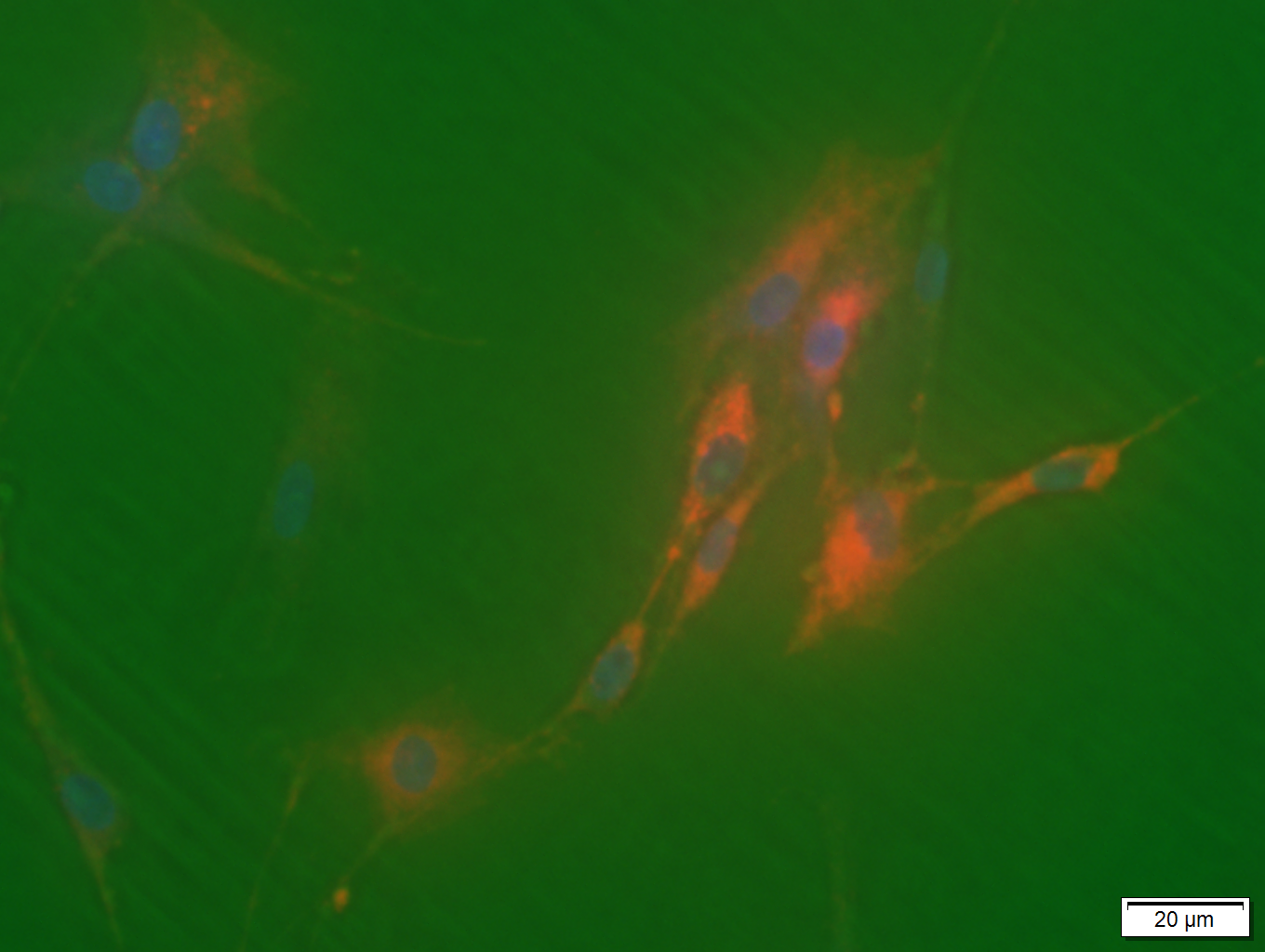

Supplement: S3 File — (ZIP) [file pone.0191616.s003.zip › Original data underlying the findings described in manuscript-Internalization of DiI-Labeled exosomes into CSCs/A41 (5).tif]

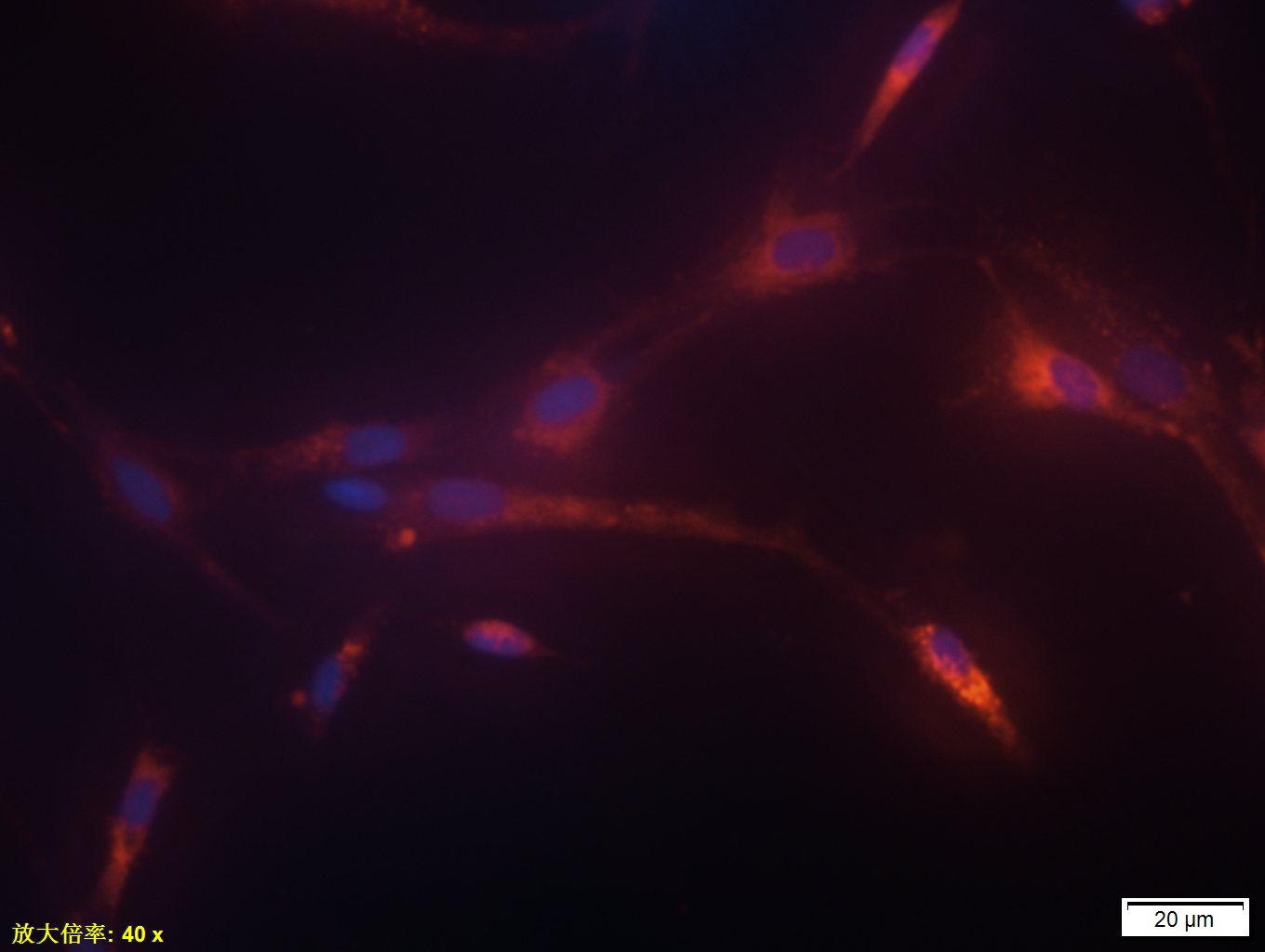

Supplement: S3 File — (ZIP) [file pone.0191616.s003.zip › Original data underlying the findings described in manuscript-Internalization of DiI-Labeled exosomes into CSCs/A42 (1).tif]

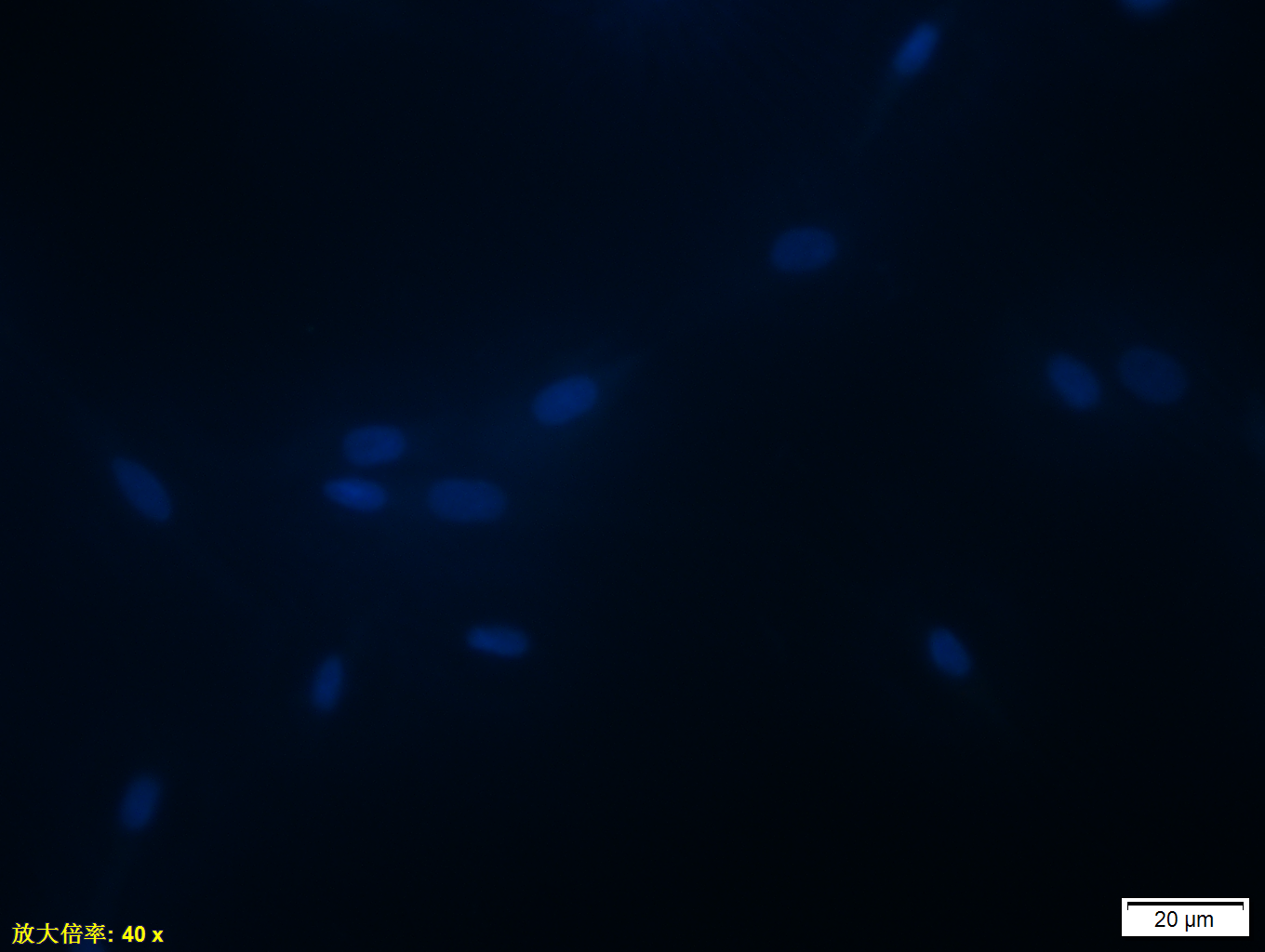

Supplement: S3 File — (ZIP) [file pone.0191616.s003.zip › Original data underlying the findings described in manuscript-Internalization of DiI-Labeled exosomes into CSCs/A42 (2).tif]

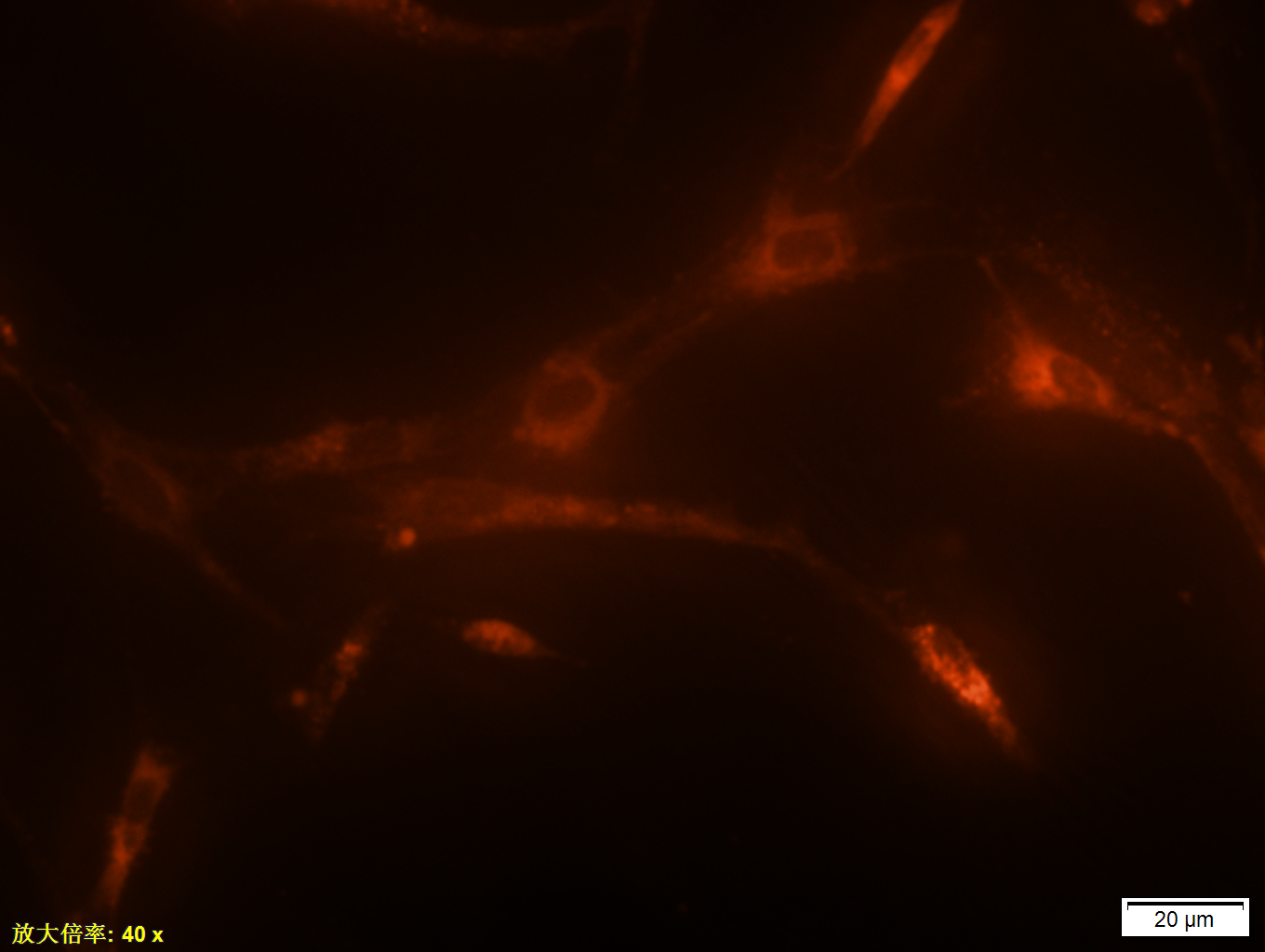

Supplement: S3 File — (ZIP) [file pone.0191616.s003.zip › Original data underlying the findings described in manuscript-Internalization of DiI-Labeled exosomes into CSCs/A42 (3).tif]

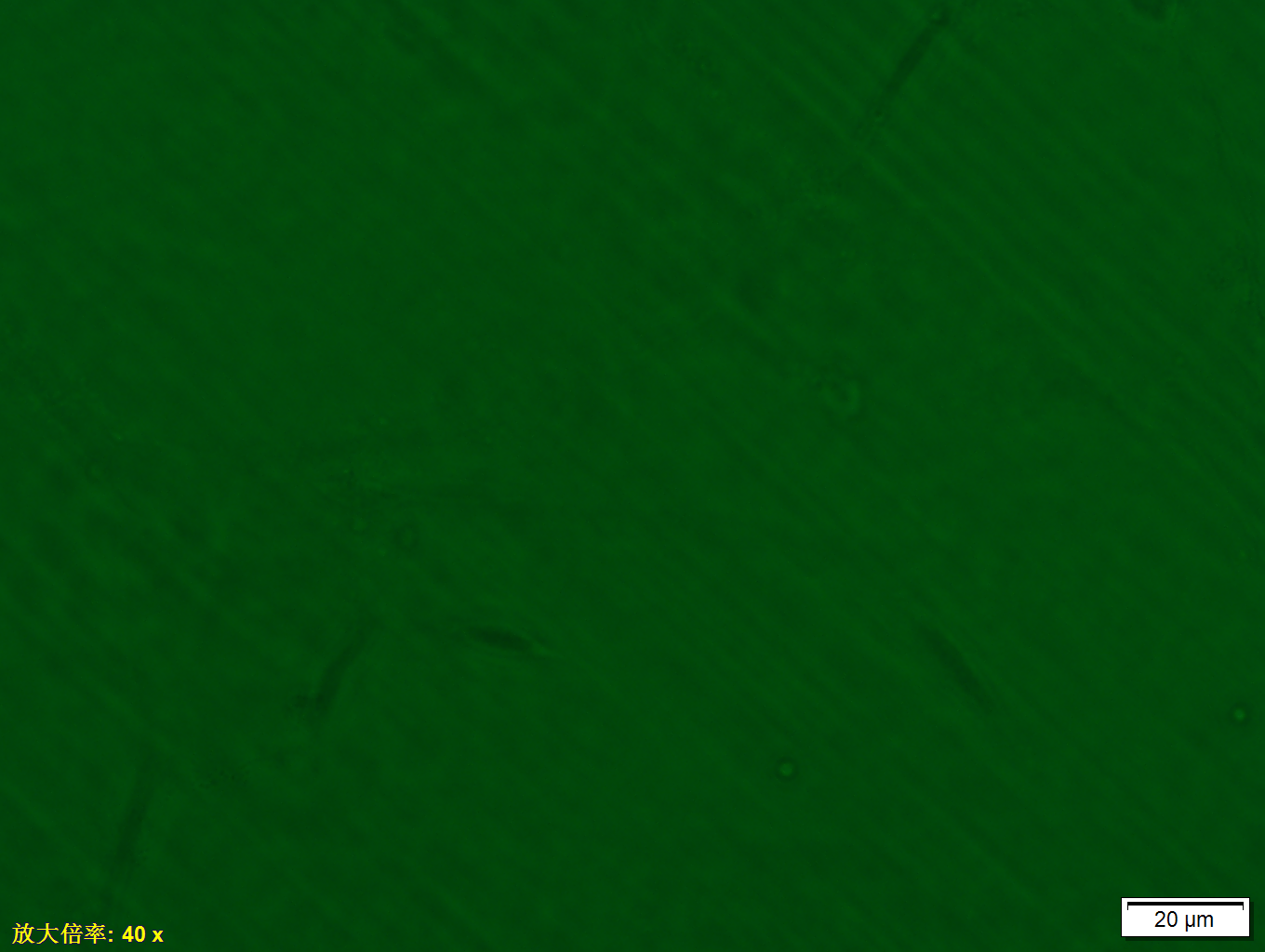

Supplement: S3 File — (ZIP) [file pone.0191616.s003.zip › Original data underlying the findings described in manuscript-Internalization of DiI-Labeled exosomes into CSCs/A42 (4).tif]

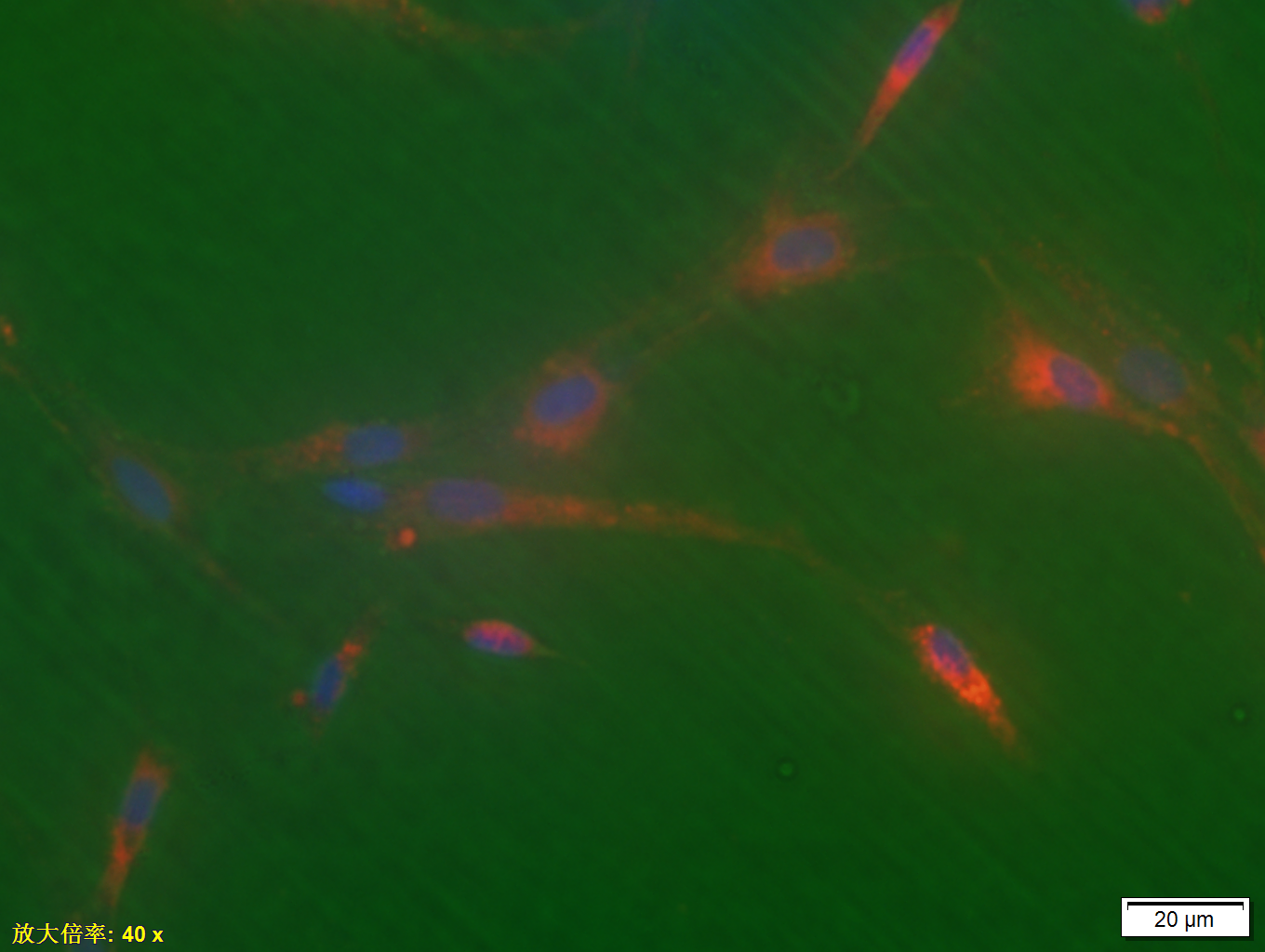

Supplement: S3 File — (ZIP) [file pone.0191616.s003.zip › Original data underlying the findings described in manuscript-Internalization of DiI-Labeled exosomes into CSCs/A42 (5).tif]

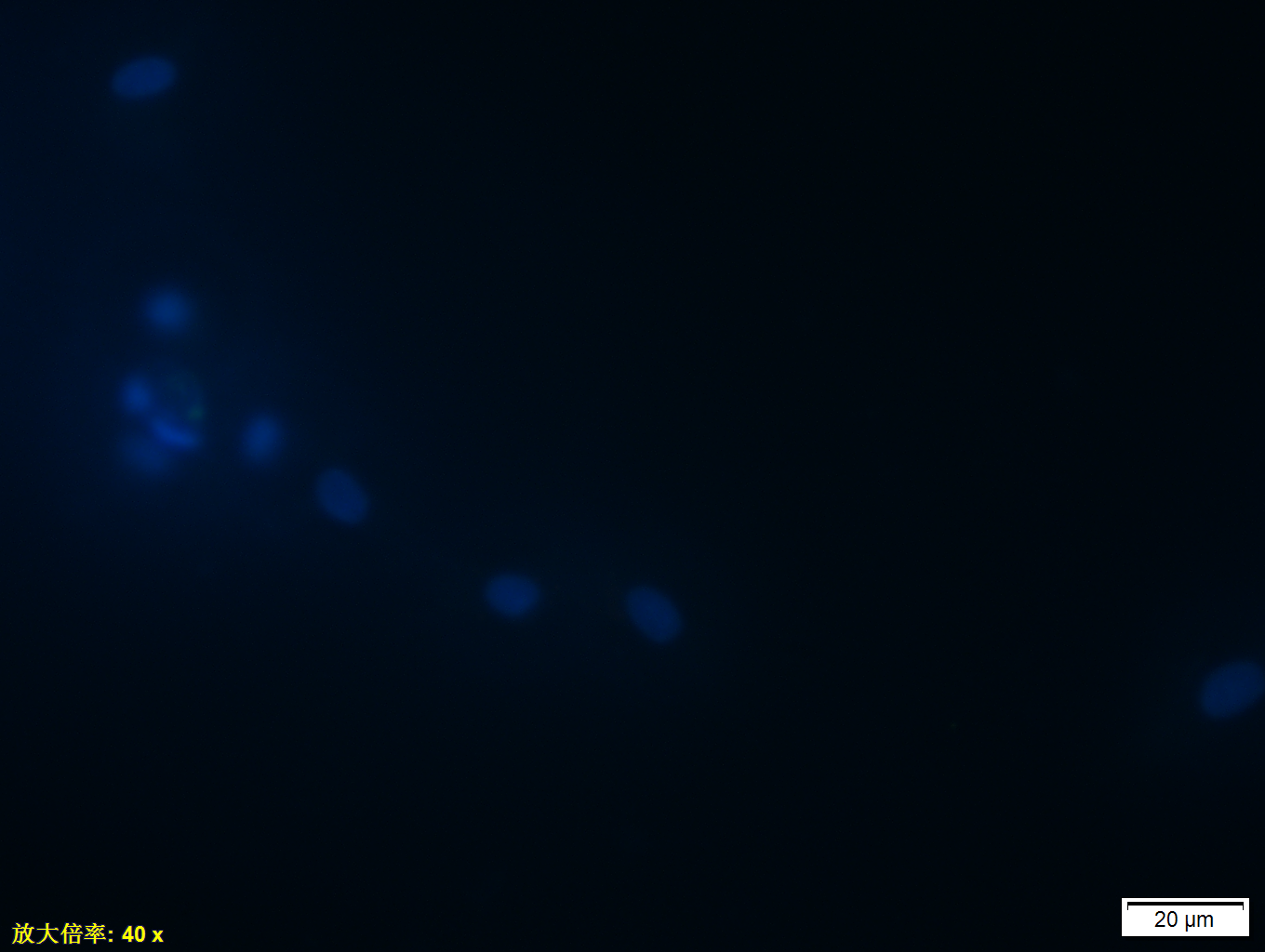

Supplement: S3 File — (ZIP) [file pone.0191616.s003.zip › Original data underlying the findings described in manuscript-Internalization of DiI-Labeled exosomes into CSCs/A43 (1).tif]

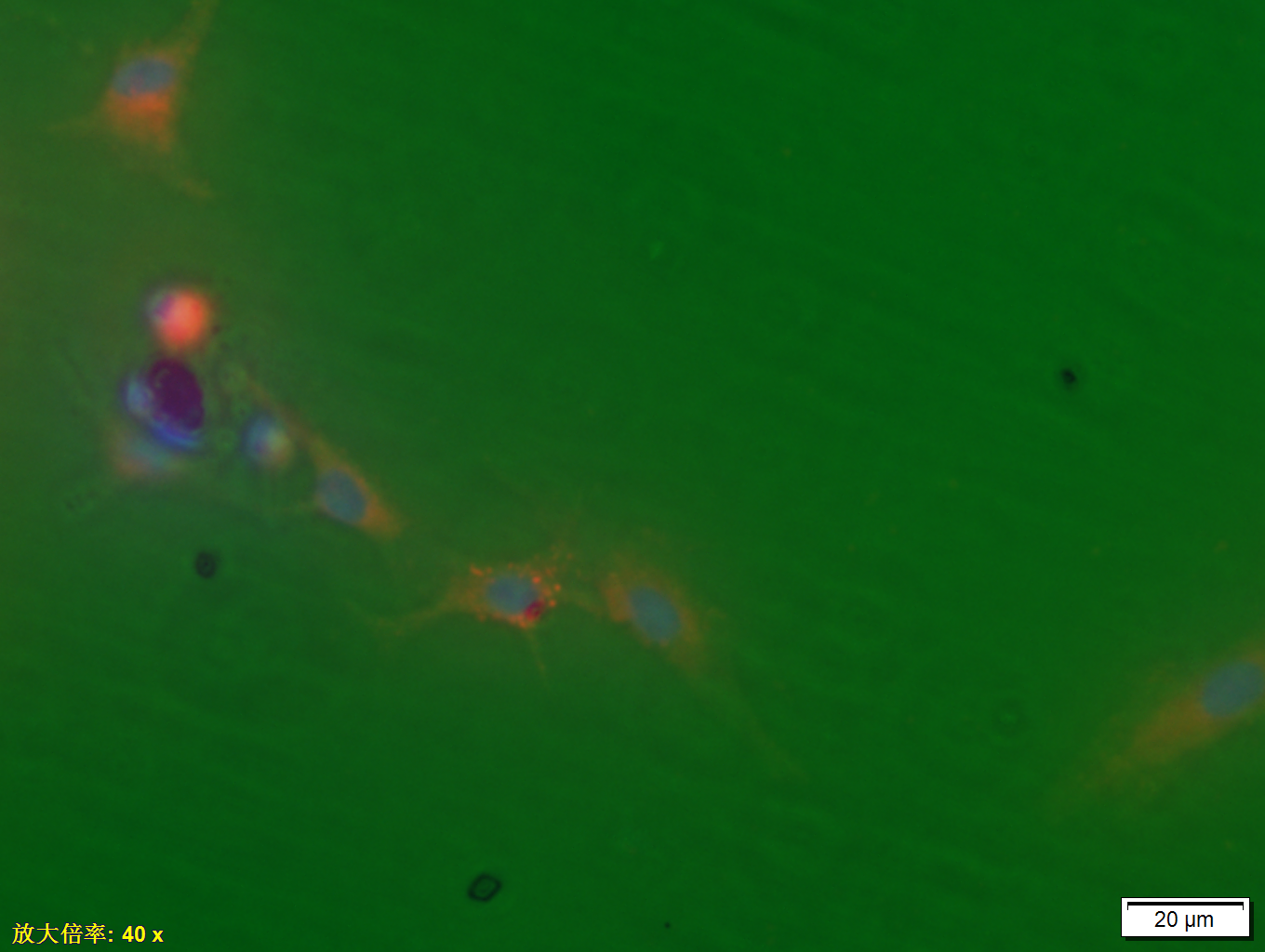

Supplement: S3 File — (ZIP) [file pone.0191616.s003.zip › Original data underlying the findings described in manuscript-Internalization of DiI-Labeled exosomes into CSCs/A43 (2).tif]

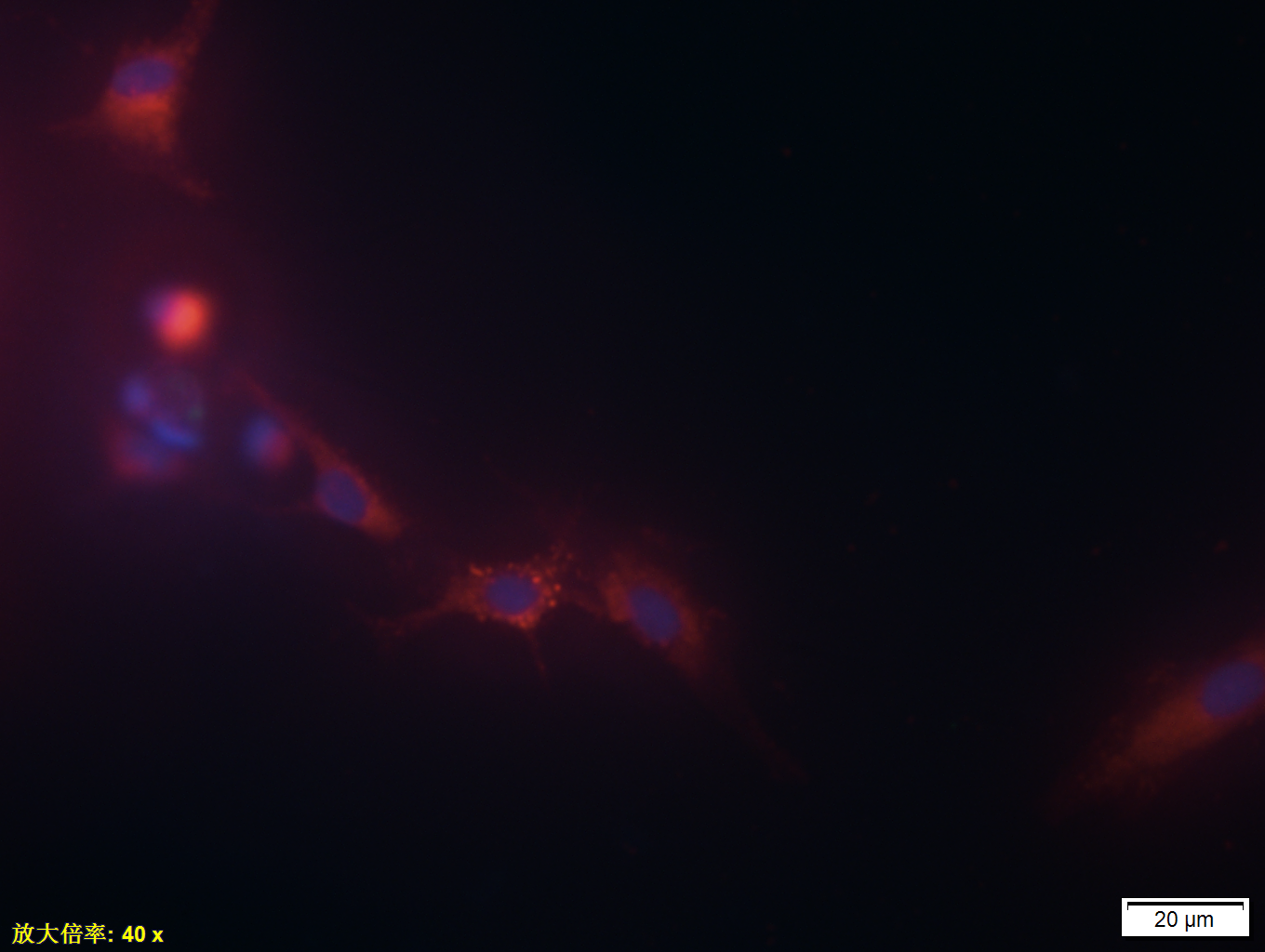

Supplement: S3 File — (ZIP) [file pone.0191616.s003.zip › Original data underlying the findings described in manuscript-Internalization of DiI-Labeled exosomes into CSCs/A43 (3).tif]

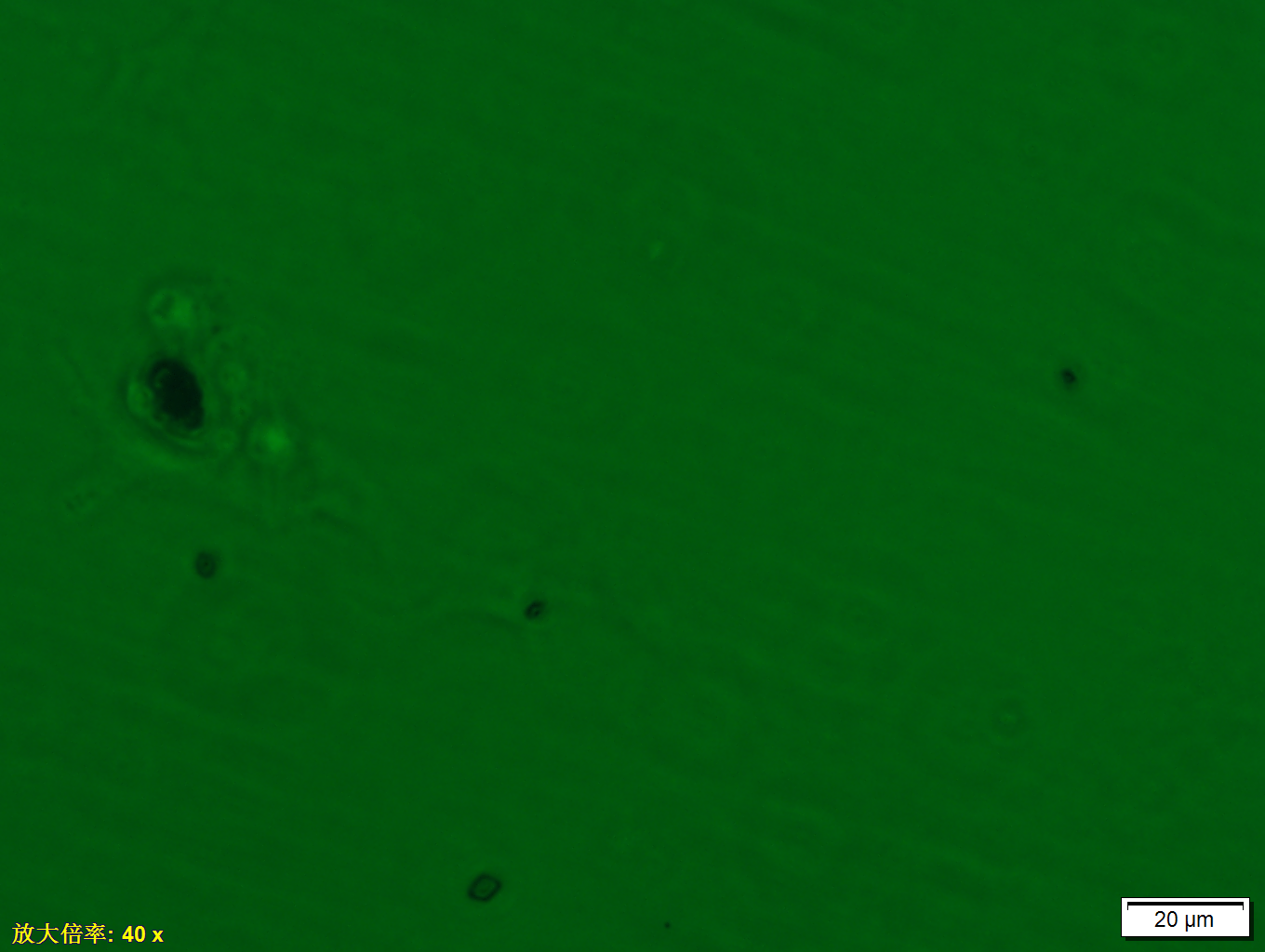

Supplement: S3 File — (ZIP) [file pone.0191616.s003.zip › Original data underlying the findings described in manuscript-Internalization of DiI-Labeled exosomes into CSCs/A43 (4).tif]

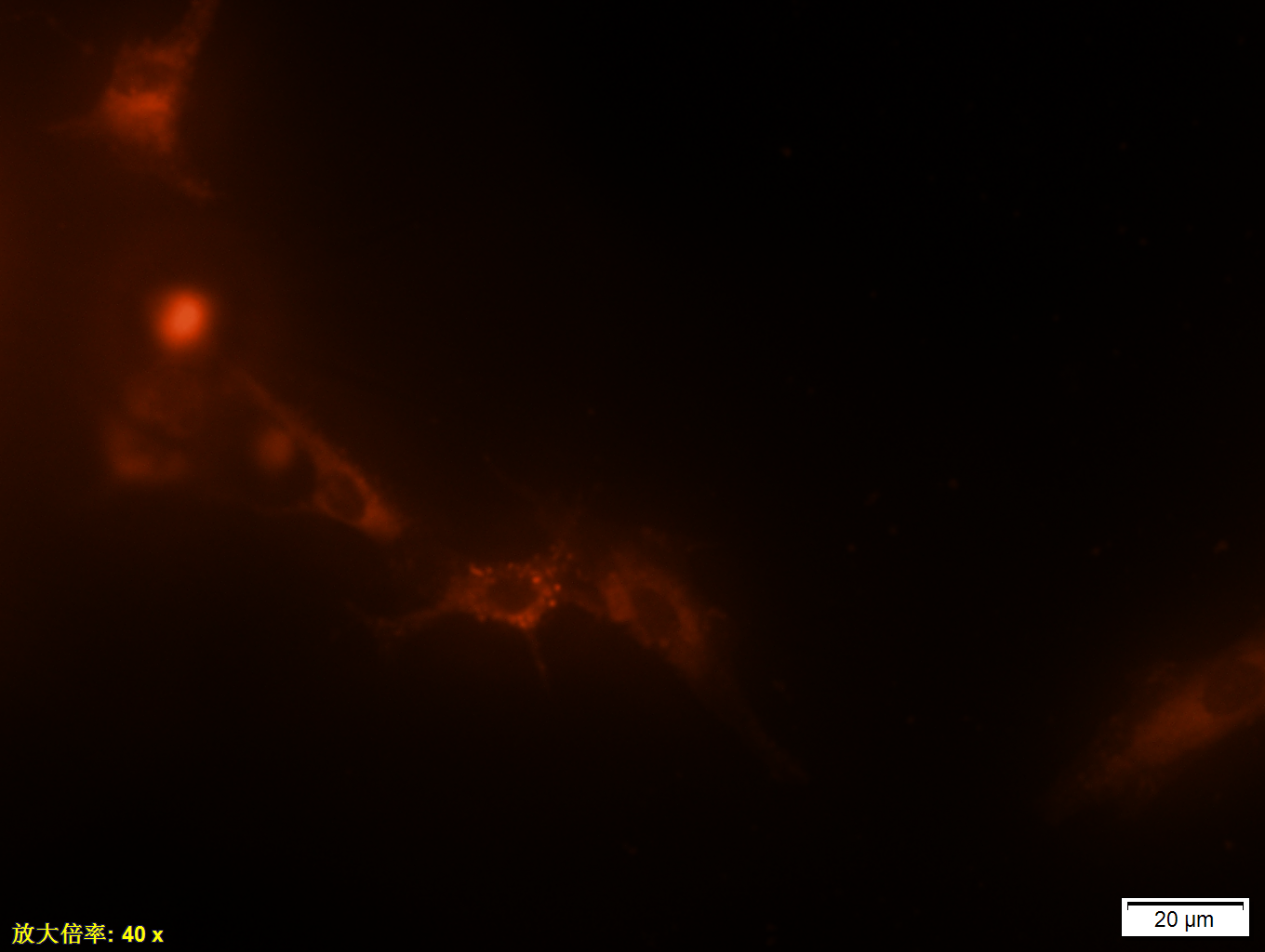

Supplement: S3 File — (ZIP) [file pone.0191616.s003.zip › Original data underlying the findings described in manuscript-Internalization of DiI-Labeled exosomes into CSCs/A43 (5).tif]

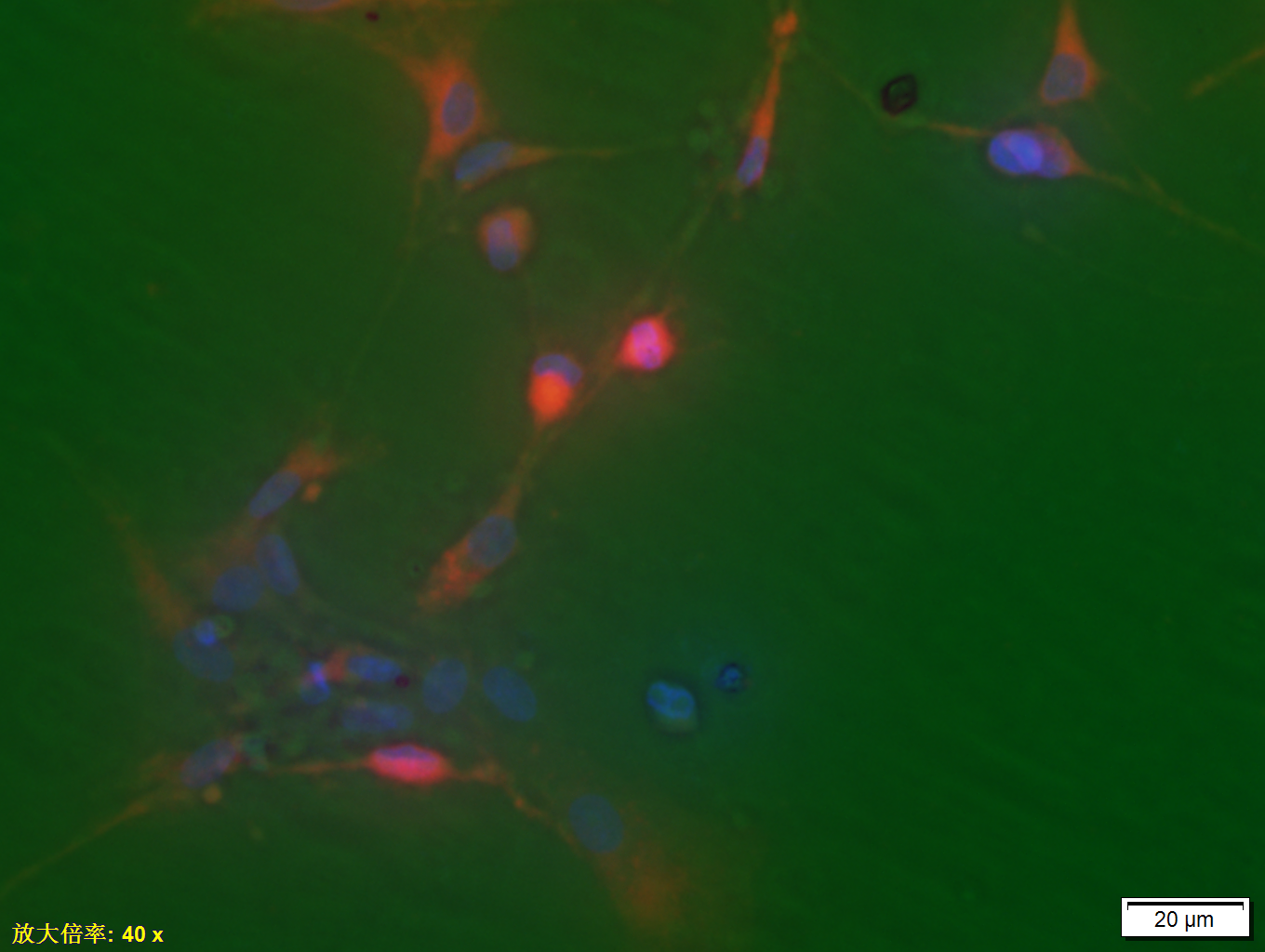

Supplement: S3 File — (ZIP) [file pone.0191616.s003.zip › Original data underlying the findings described in manuscript-Internalization of DiI-Labeled exosomes into CSCs/A44 (1).tif]

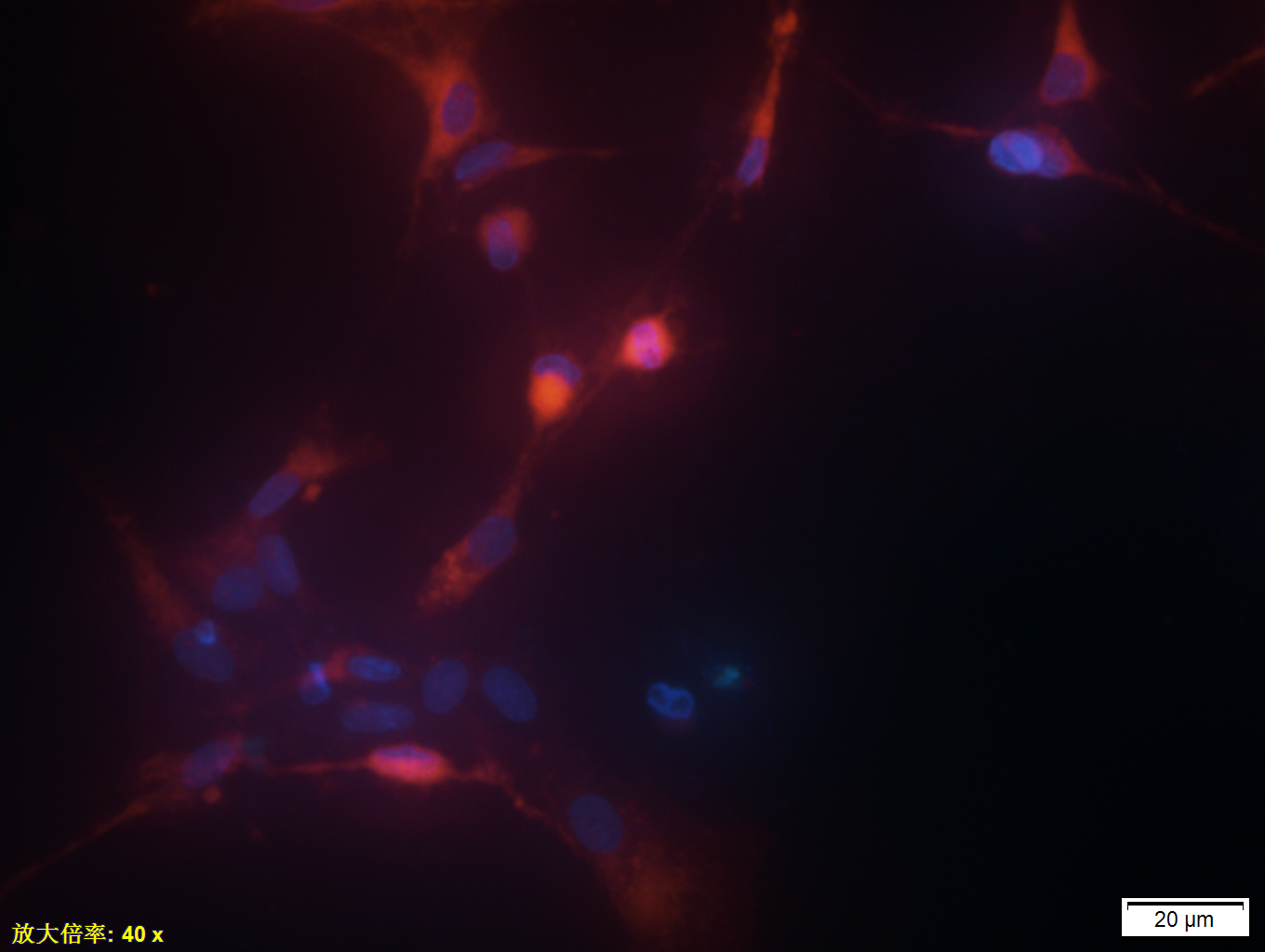

Supplement: S3 File — (ZIP) [file pone.0191616.s003.zip › Original data underlying the findings described in manuscript-Internalization of DiI-Labeled exosomes into CSCs/A44 (2).tif]

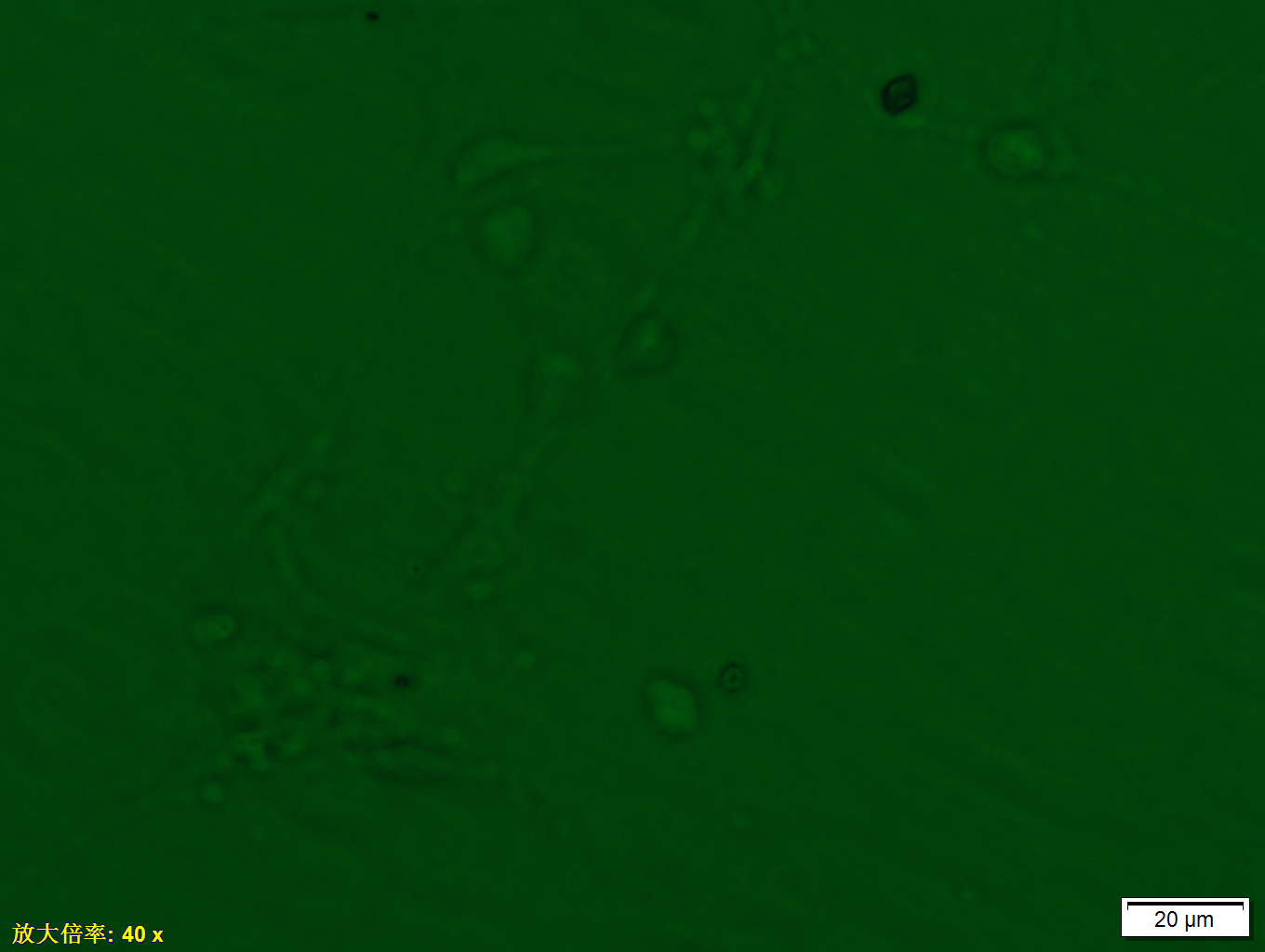

Supplement: S3 File — (ZIP) [file pone.0191616.s003.zip › Original data underlying the findings described in manuscript-Internalization of DiI-Labeled exosomes into CSCs/A44 (3).tif]

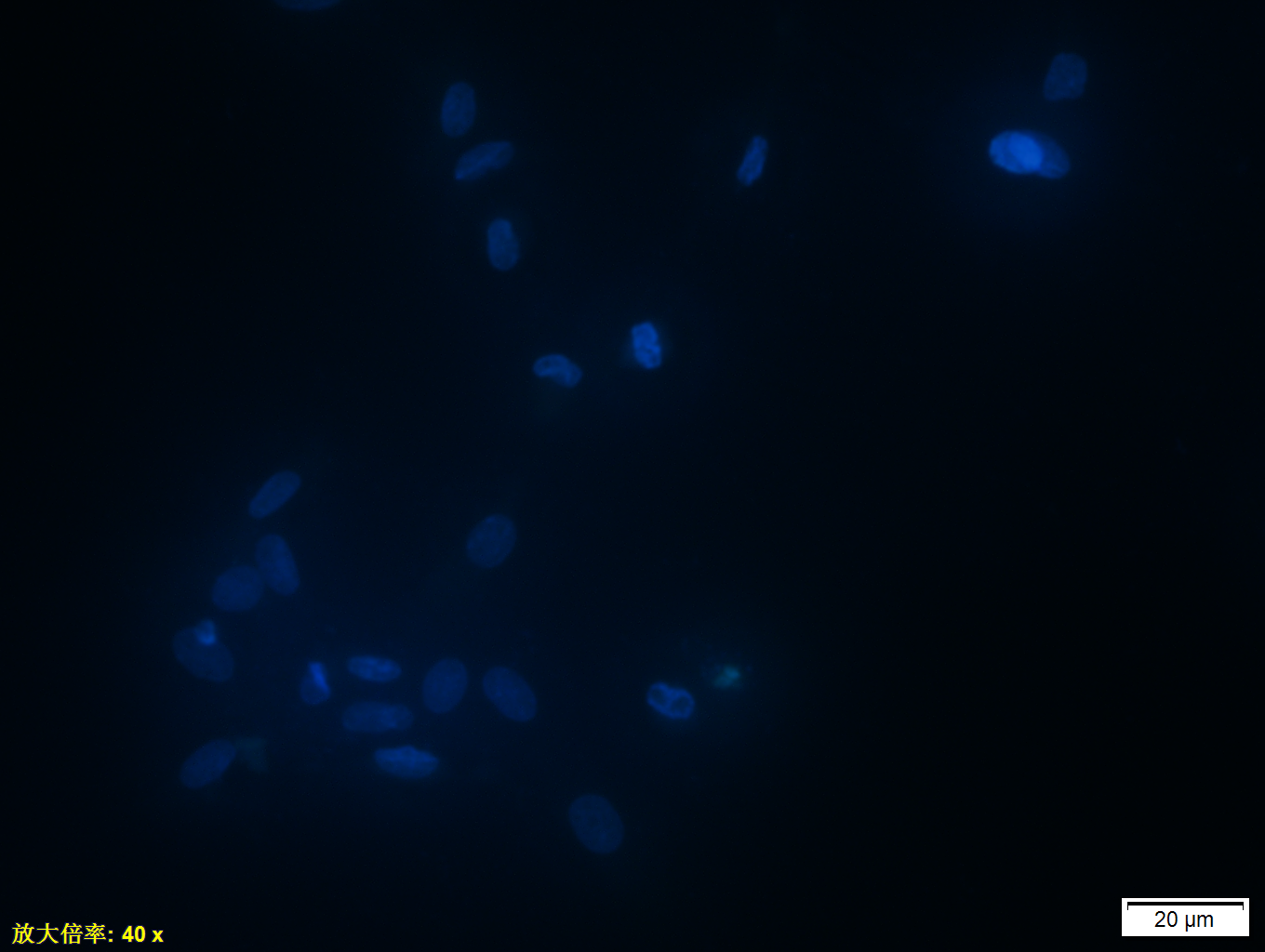

Supplement: S3 File — (ZIP) [file pone.0191616.s003.zip › Original data underlying the findings described in manuscript-Internalization of DiI-Labeled exosomes into CSCs/A44 (4).tif]

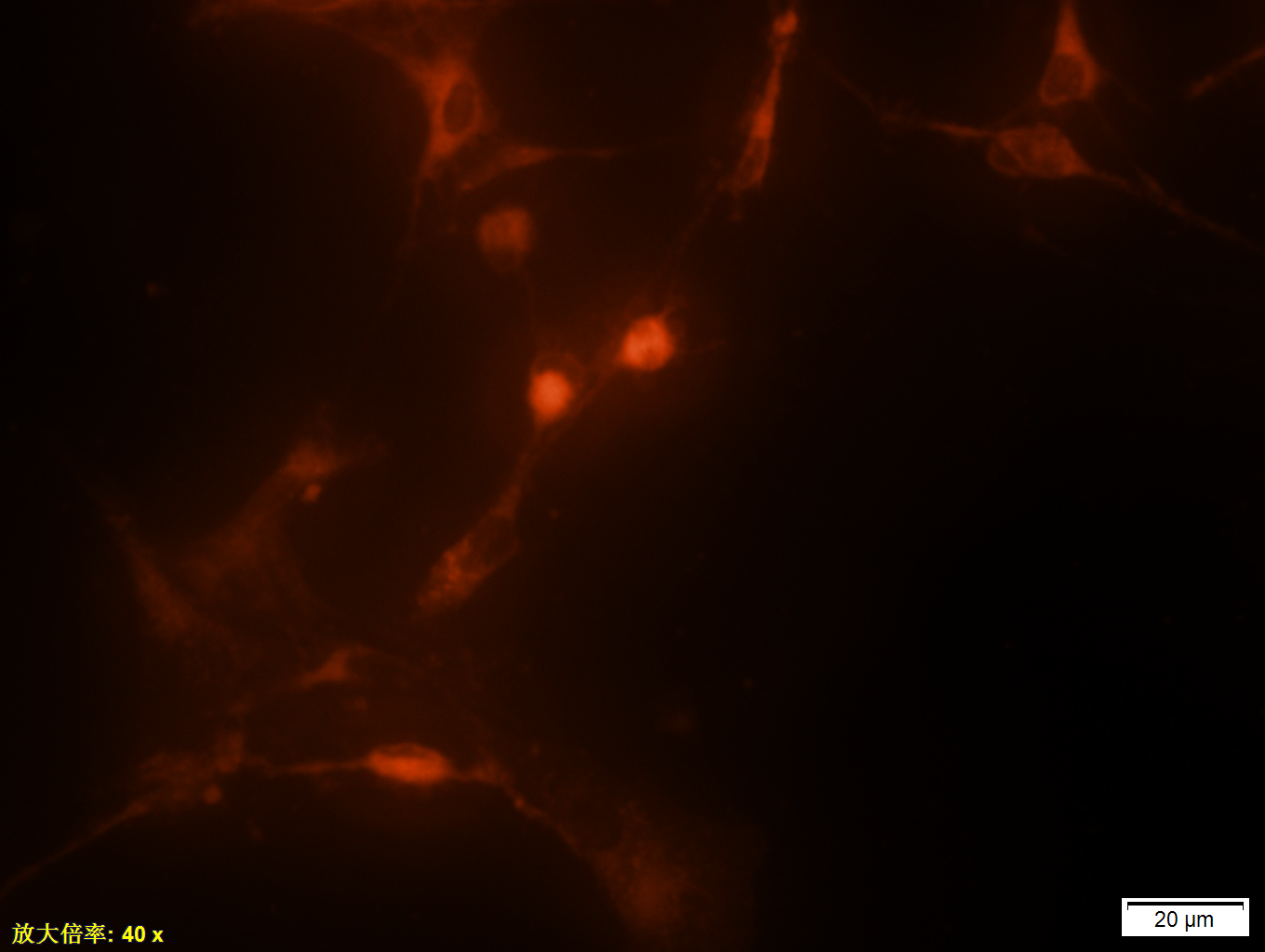

Supplement: S3 File — (ZIP) [file pone.0191616.s003.zip › Original data underlying the findings described in manuscript-Internalization of DiI-Labeled exosomes into CSCs/A44 (5).tif]

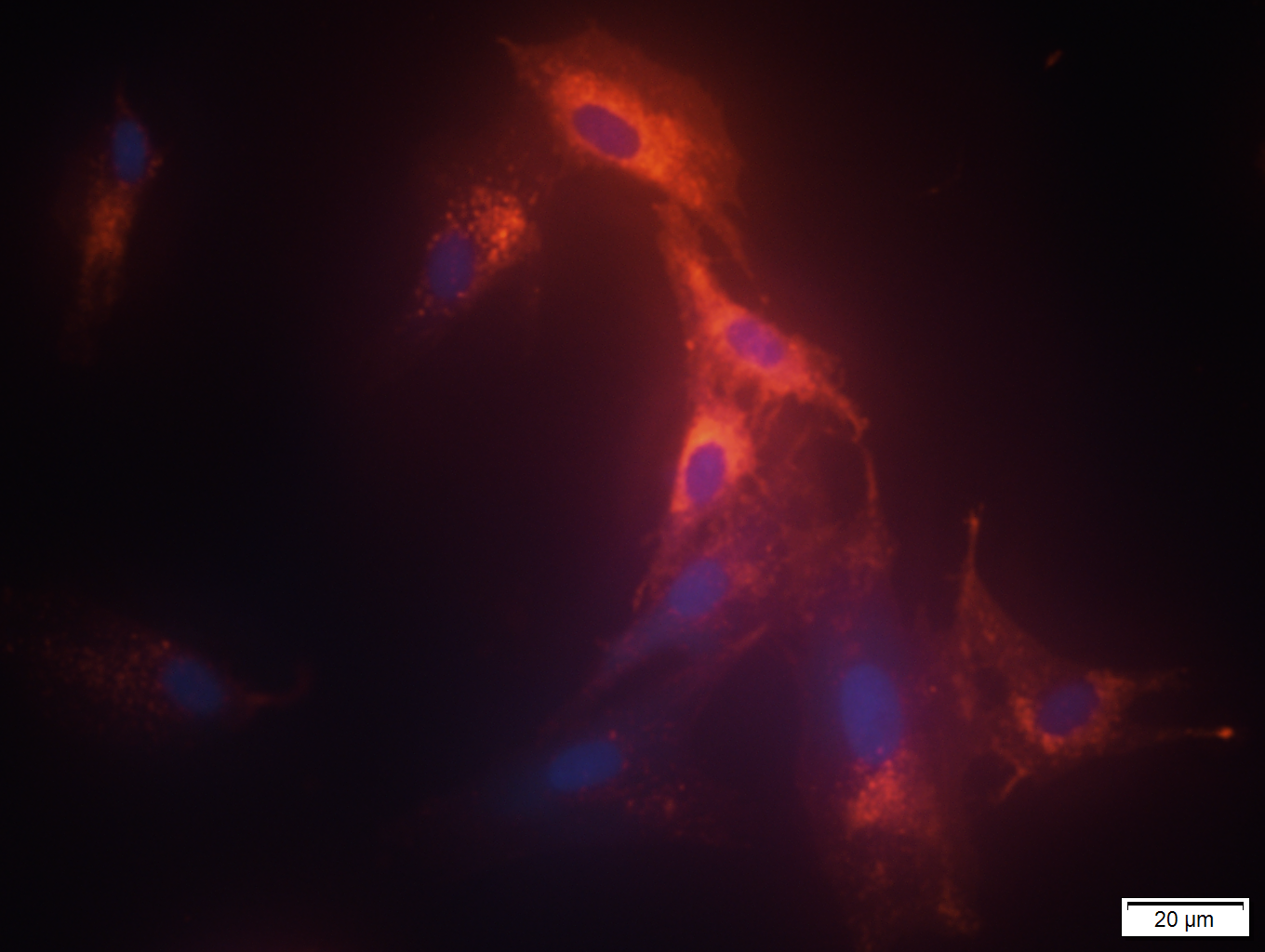

Supplement: S3 File — (ZIP) [file pone.0191616.s003.zip › Original data underlying the findings described in manuscript-Internalization of DiI-Labeled exosomes into CSCs/A45 (1).tif]

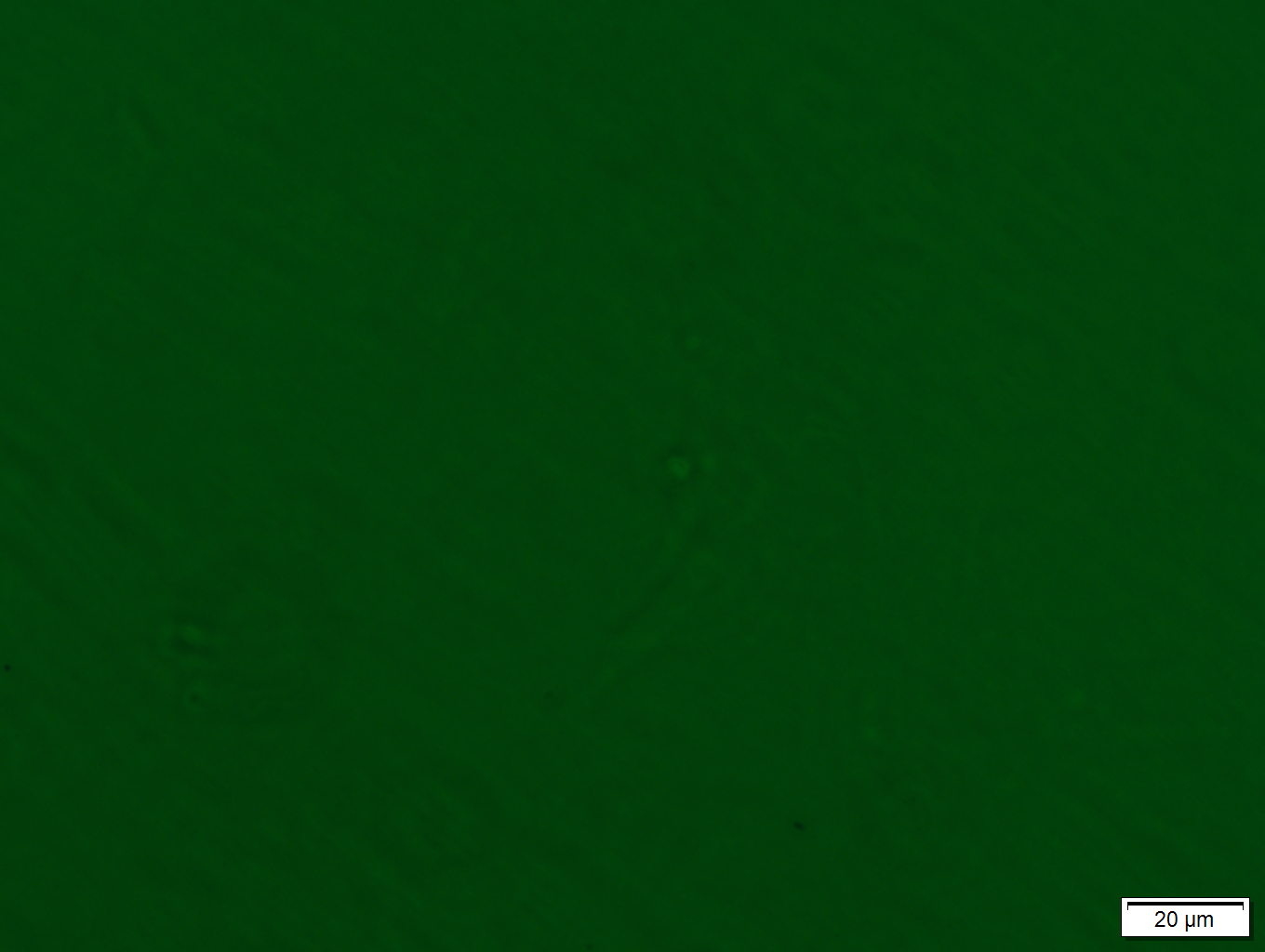

Supplement: S3 File — (ZIP) [file pone.0191616.s003.zip › Original data underlying the findings described in manuscript-Internalization of DiI-Labeled exosomes into CSCs/A45 (2).tif]

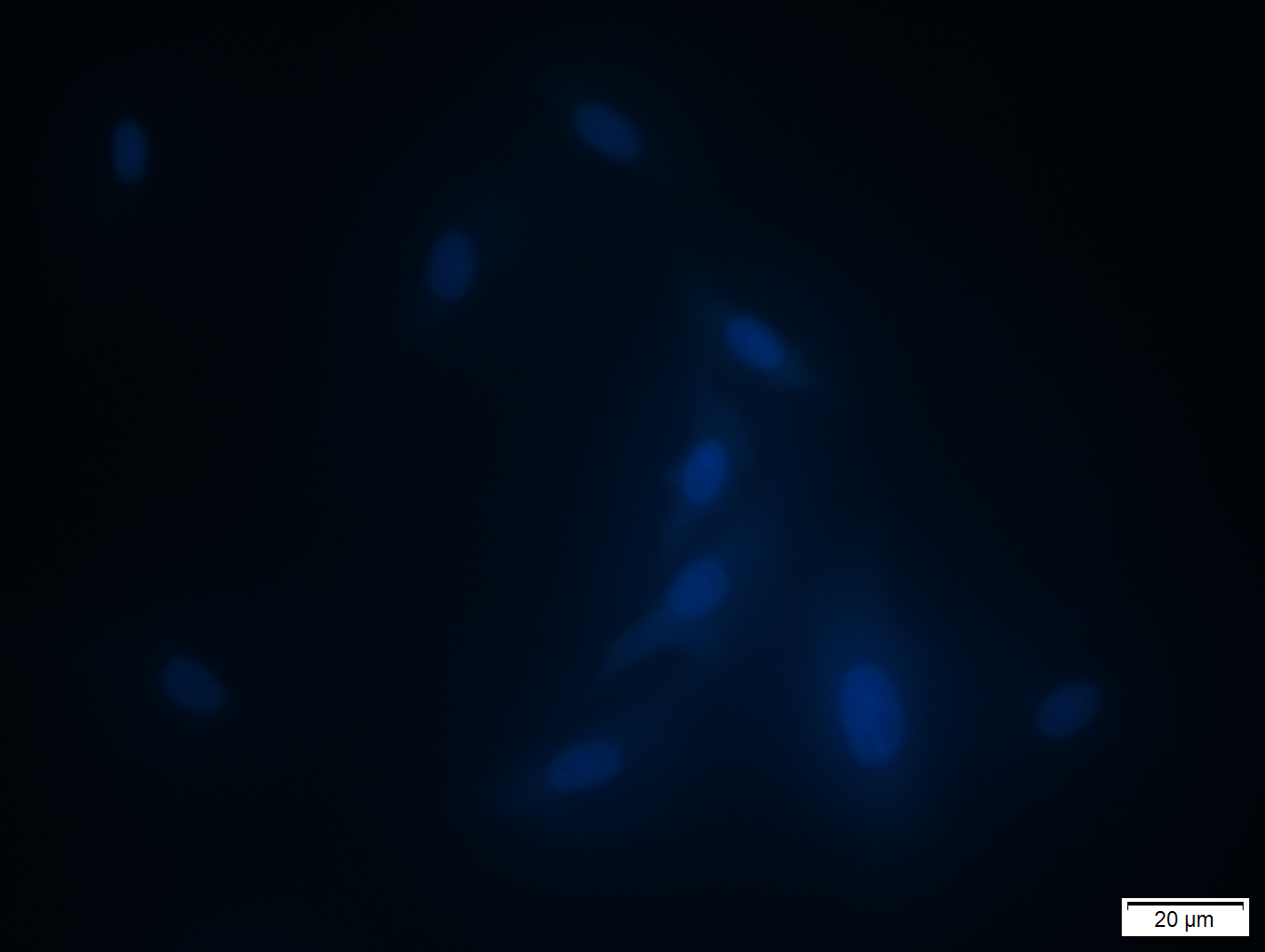

Supplement: S3 File — (ZIP) [file pone.0191616.s003.zip › Original data underlying the findings described in manuscript-Internalization of DiI-Labeled exosomes into CSCs/A45 (3).tif]

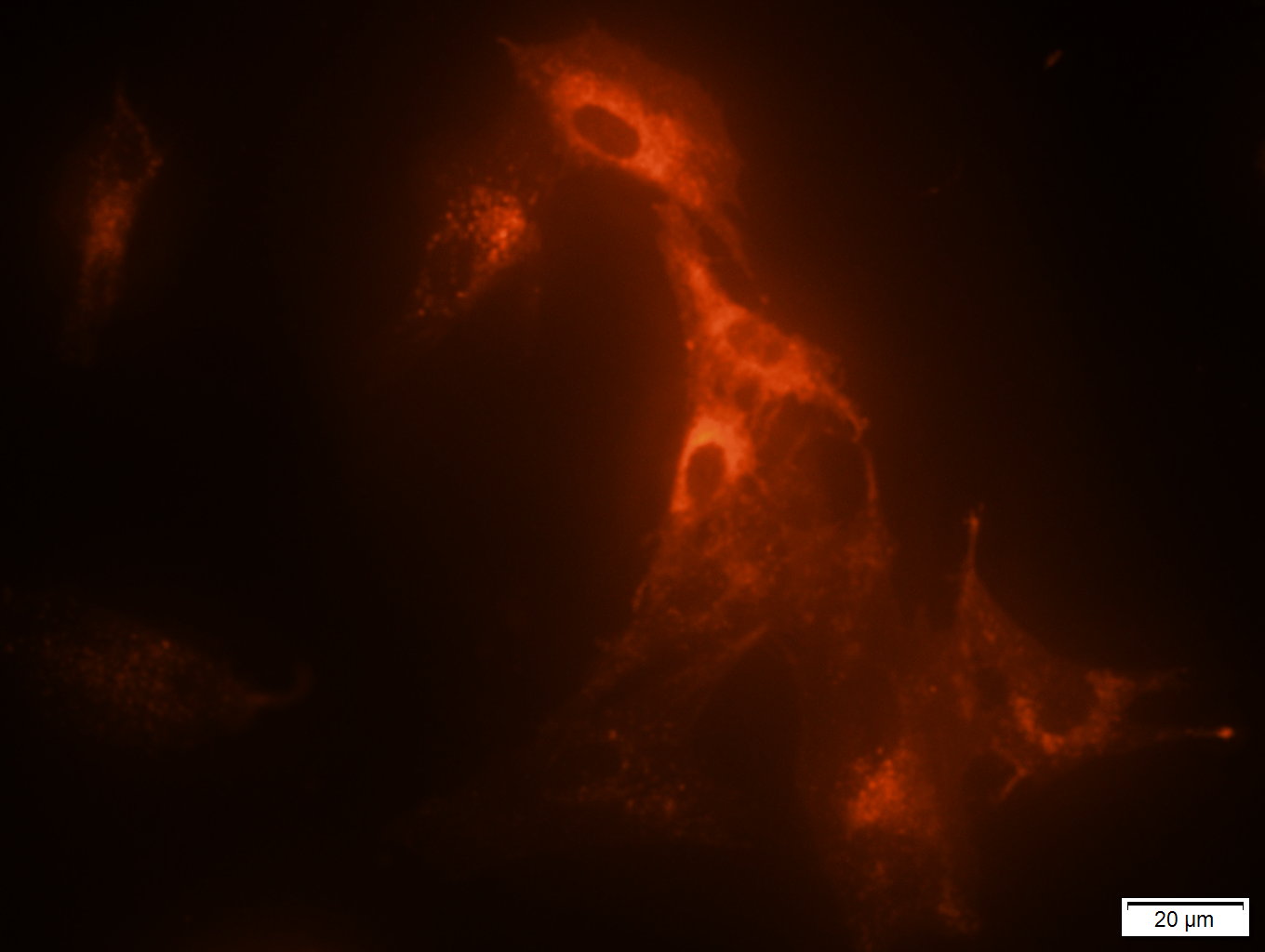

Supplement: S3 File — (ZIP) [file pone.0191616.s003.zip › Original data underlying the findings described in manuscript-Internalization of DiI-Labeled exosomes into CSCs/A45 (4).tif]

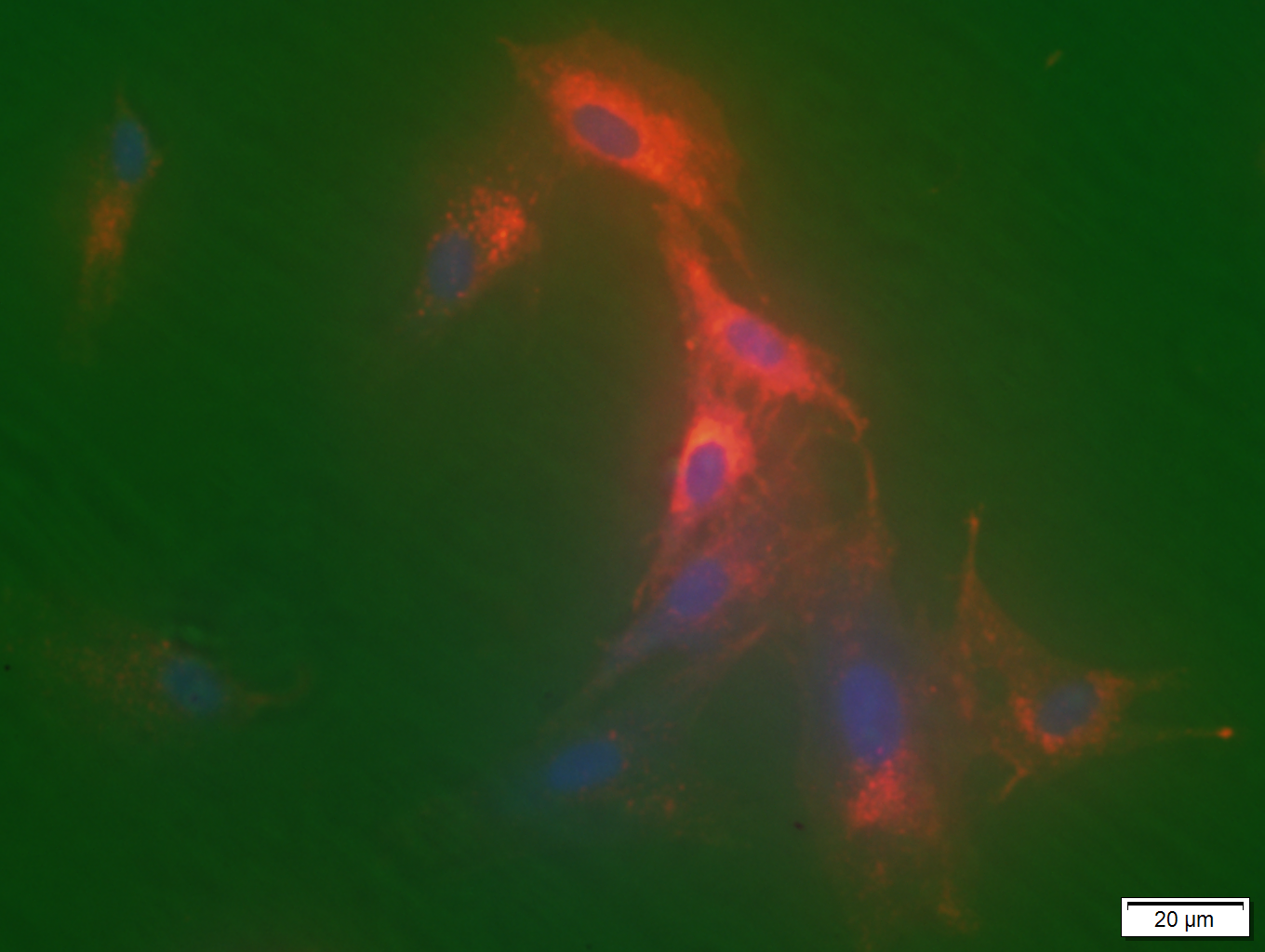

Supplement: S3 File — (ZIP) [file pone.0191616.s003.zip › Original data underlying the findings described in manuscript-Internalization of DiI-Labeled exosomes into CSCs/A45 (5).tif]

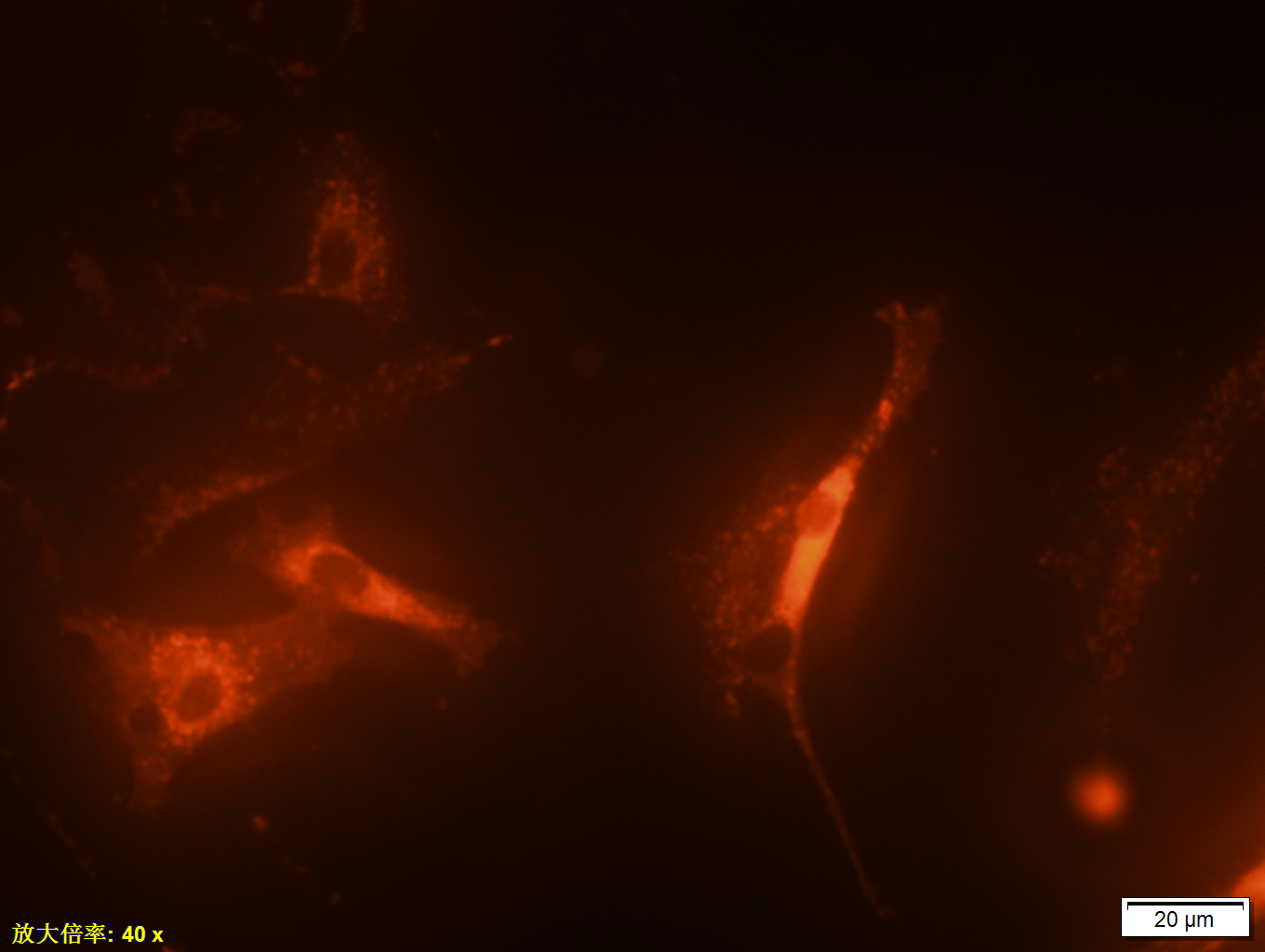

Supplement: S3 File — (ZIP) [file pone.0191616.s003.zip › Original data underlying the findings described in manuscript-Internalization of DiI-Labeled exosomes into CSCs/Relevant data underlying the findingsdescribed in manuscript.-2.tif]

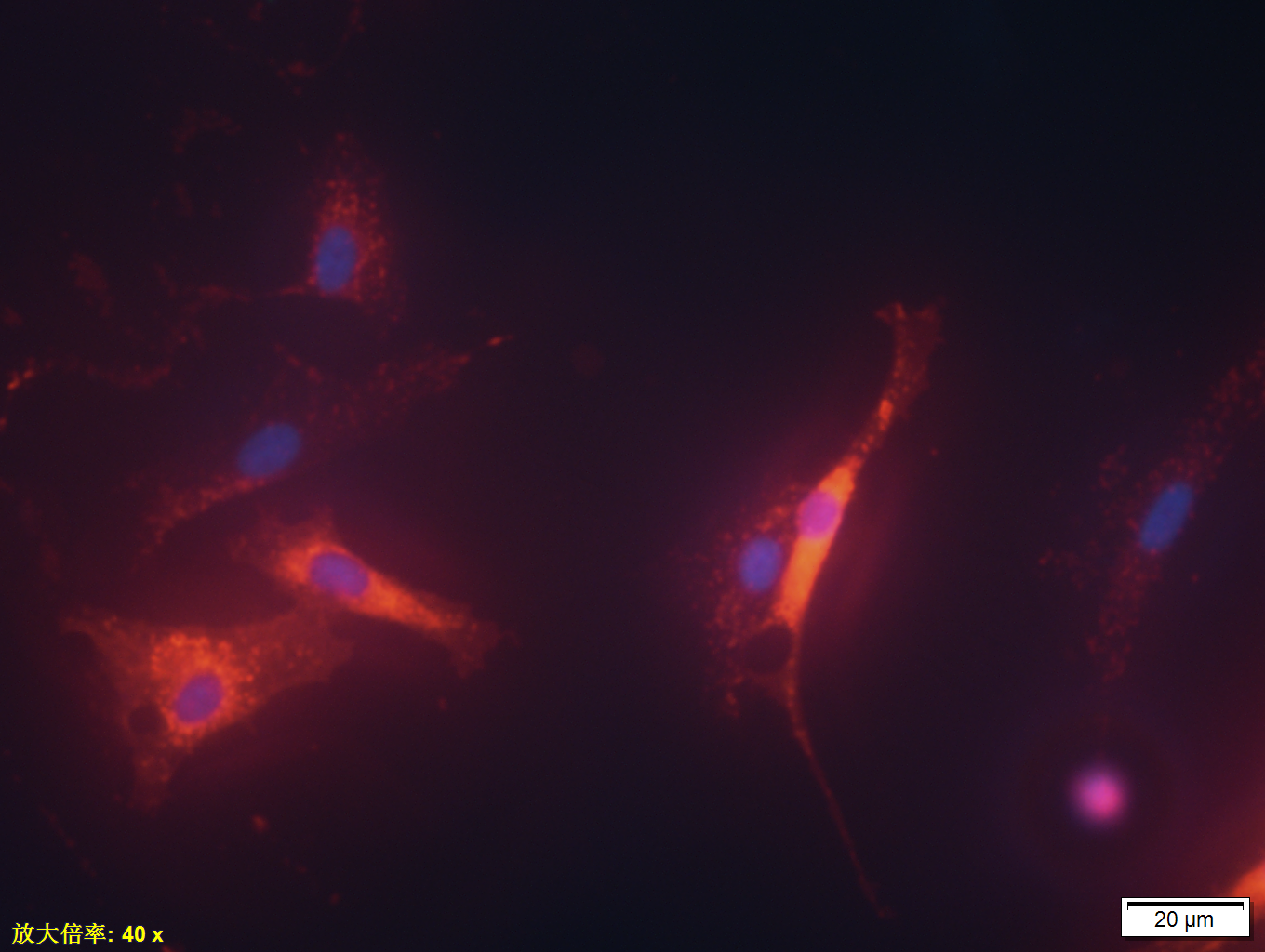

Supplement: S3 File — (ZIP) [file pone.0191616.s003.zip › Original data underlying the findings described in manuscript-Internalization of DiI-Labeled exosomes into CSCs/Relevant data underlying the findingsdescribed in manuscript.-3.tif]

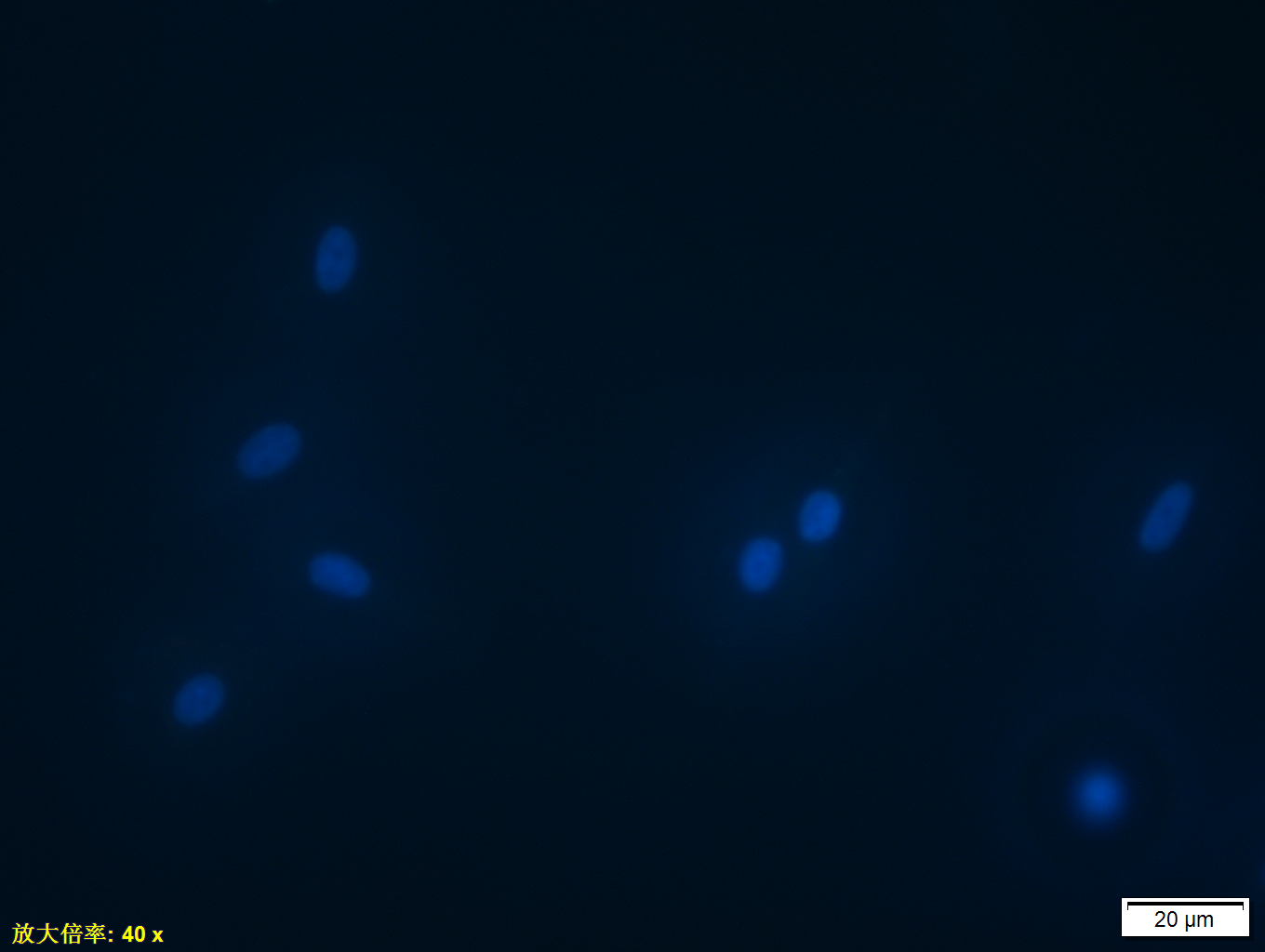

Supplement: S3 File — (ZIP) [file pone.0191616.s003.zip › Original data underlying the findings described in manuscript-Internalization of DiI-Labeled exosomes into CSCs/Relevant data underlying the findingsdescribed in manuscript..tif]

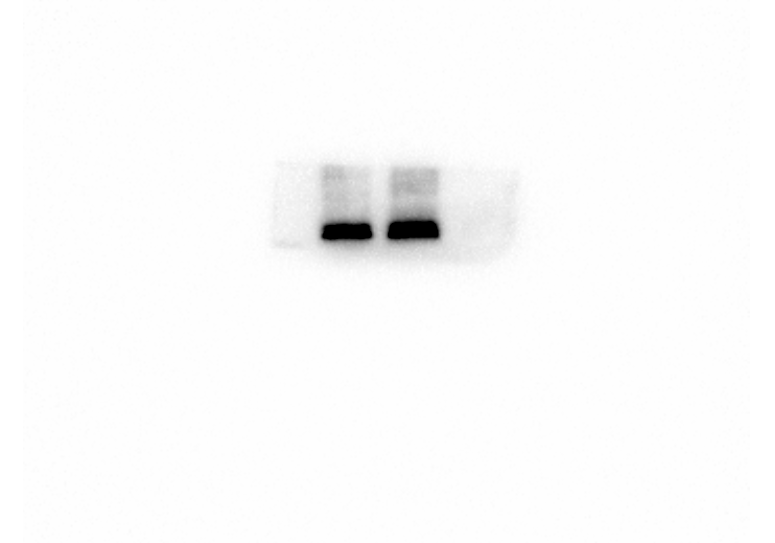

Supplement: S5 File — (ZIP) [file pone.0191616.s005.zip › Original data underlying the findings described in manuscript-The levels of cell apoptotic related genes were detected by western blotting/figure-2/actin.tif]

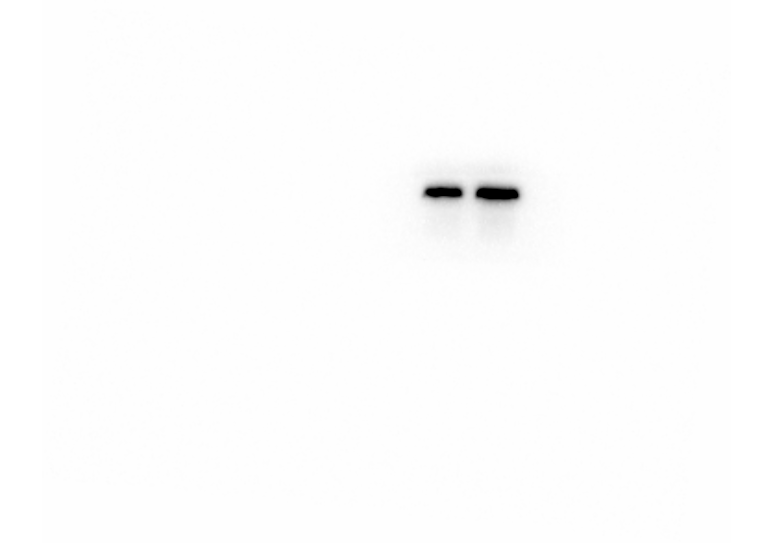

Supplement: S5 File — (ZIP) [file pone.0191616.s005.zip › Original data underlying the findings described in manuscript-The levels of cell apoptotic related genes were detected by western blotting/figure-2/caspase.tif]

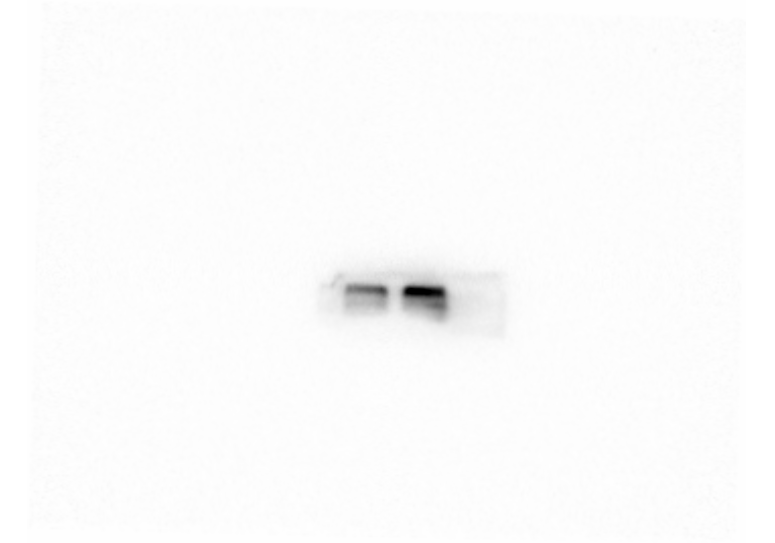

Supplement: S5 File — (ZIP) [file pone.0191616.s005.zip › Original data underlying the findings described in manuscript-The levels of cell apoptotic related genes were detected by western blotting/figure-2/cleaved caspase-3.tif]

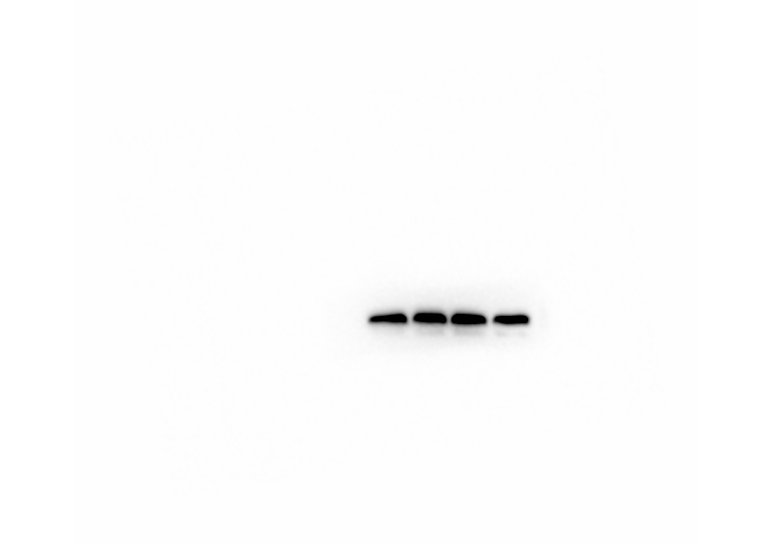

Supplement: S5 File — (ZIP) [file pone.0191616.s005.zip › Original data underlying the findings described in manuscript-The levels of cell apoptotic related genes were detected by western blotting/figure-3/Caespase-3.tif]

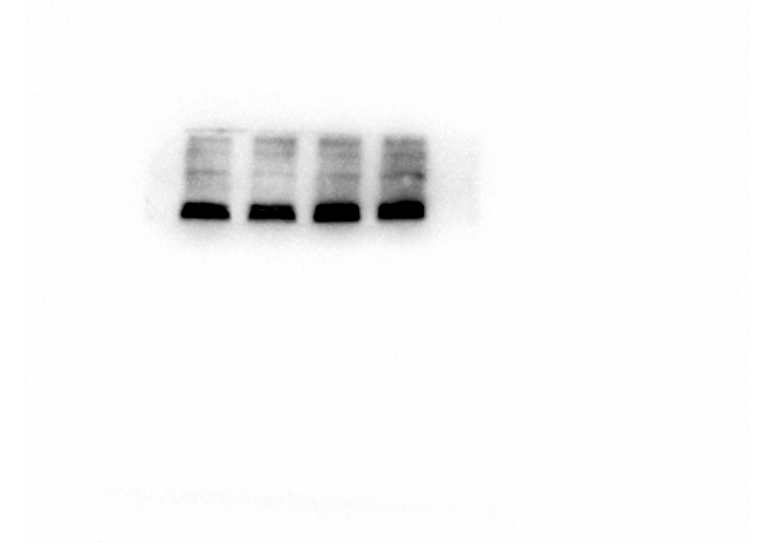

Supplement: S5 File — (ZIP) [file pone.0191616.s005.zip › Original data underlying the findings described in manuscript-The levels of cell apoptotic related genes were detected by western blotting/figure-3/actin.tif]

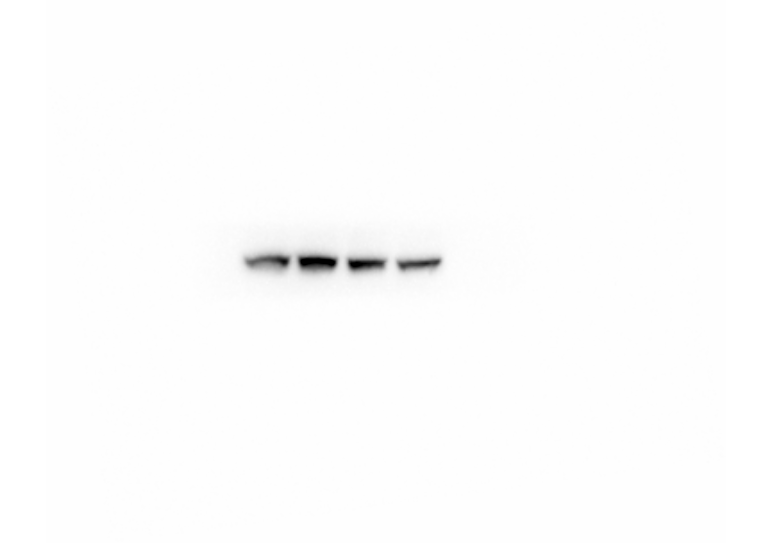

Supplement: S5 File — (ZIP) [file pone.0191616.s005.zip › Original data underlying the findings described in manuscript-The levels of cell apoptotic related genes were detected by western blotting/figure-3/cleaved caspase-3.tif]

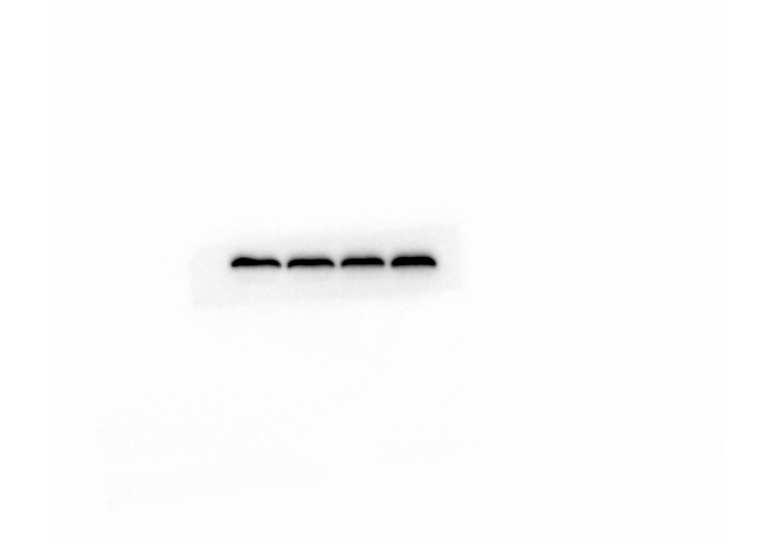

Supplement: S5 File — (ZIP) [file pone.0191616.s005.zip › Original data underlying the findings described in manuscript-The levels of cell apoptotic related genes were detected by western blotting/figure-4/actin.tif]

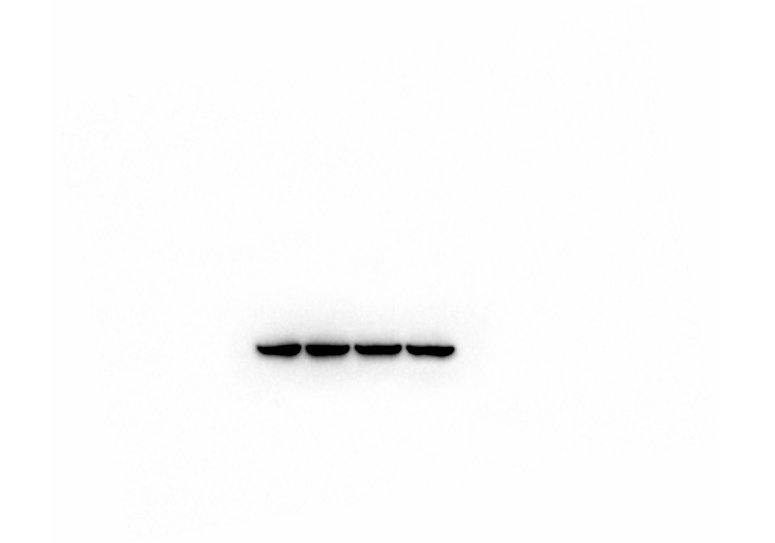

Supplement: S5 File — (ZIP) [file pone.0191616.s005.zip › Original data underlying the findings described in manuscript-The levels of cell apoptotic related genes were detected by western blotting/figure-4/caspase-3.tif]

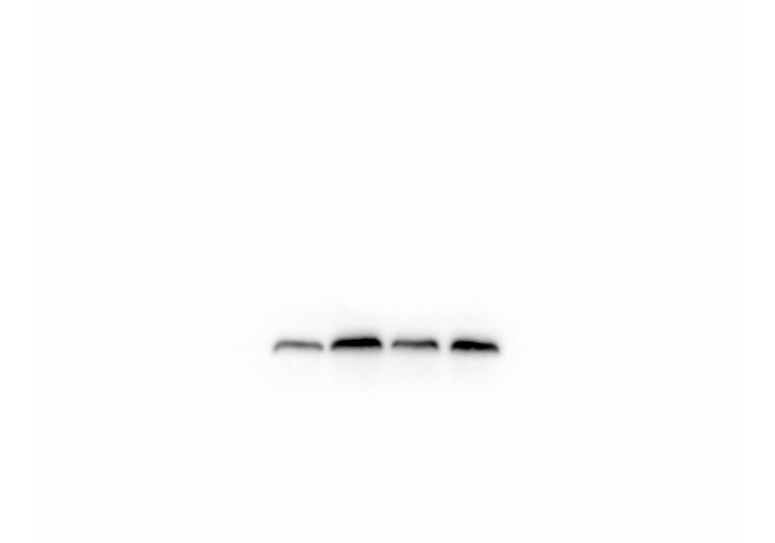

Supplement: S5 File — (ZIP) [file pone.0191616.s005.zip › Original data underlying the findings described in manuscript-The levels of cell apoptotic related genes were detected by western blotting/figure-4/cleaved caspase-3.tif]

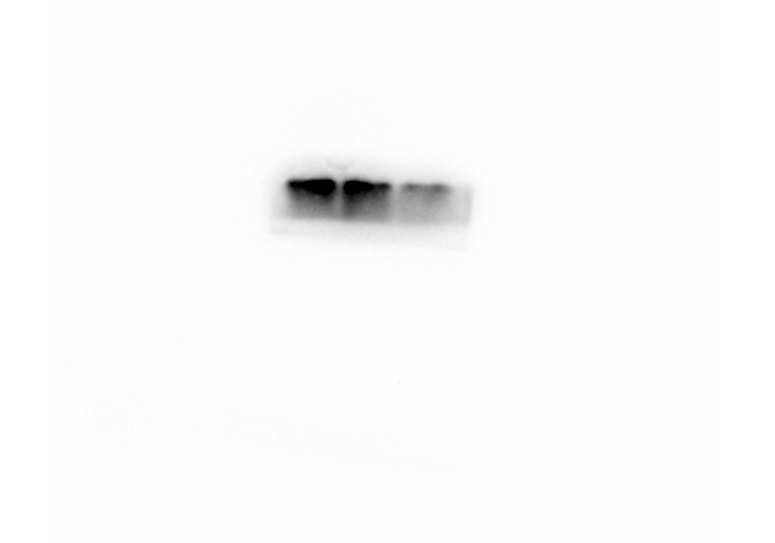

Supplement: S5 File — (ZIP) [file pone.0191616.s005.zip › Original data underlying the findings described in manuscript-The levels of cell apoptotic related genes were detected by western blotting/figure-5/PTEN (siR).tif]

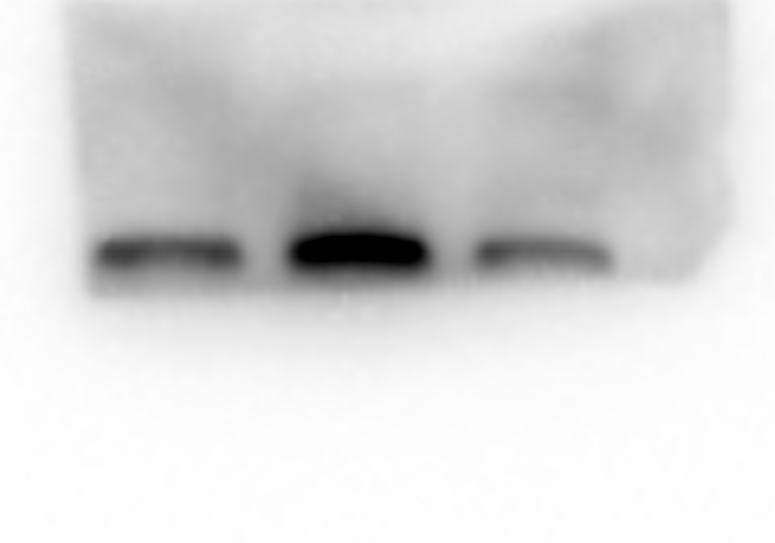

Supplement: S5 File — (ZIP) [file pone.0191616.s005.zip › Original data underlying the findings described in manuscript-The levels of cell apoptotic related genes were detected by western blotting/figure-5/PTEN(miR).tif]

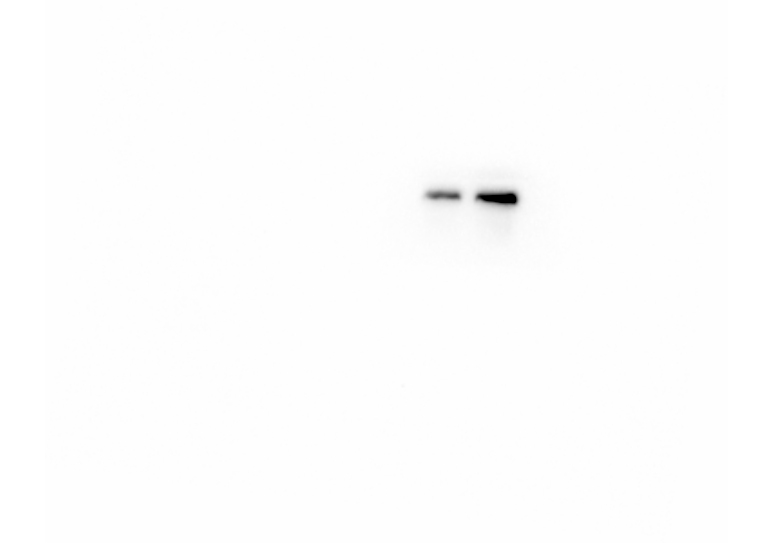

Supplement: S5 File — (ZIP) [file pone.0191616.s005.zip › Original data underlying the findings described in manuscript-The levels of cell apoptotic related genes were detected by western blotting/figure-5/PTEN.tif]
