# Supplementary figures and images for: Bone marrow mesenchymal stem cell-derived exosomal miR-21 protects C-kit+ cardiac stem cells from oxidative injury through the PTEN/PI3K/Akt axis (part 2 of 4)
Source: PLoS One. 2018 Feb 14;13(2):e0191616. doi: 10.1371/journal.pone.0191616 (PMC5812567; doi:10.1371/journal.pone.0191616)

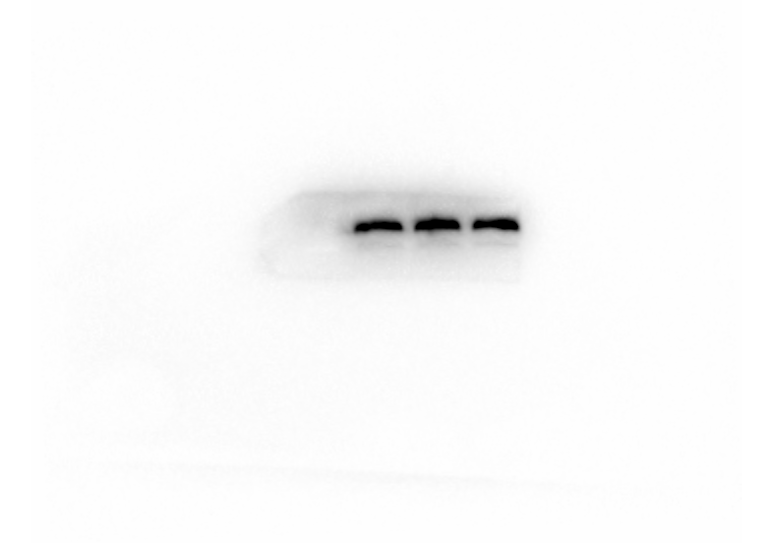

Supplement: S5 File — (ZIP) [file pone.0191616.s005.zip › Original data underlying the findings described in manuscript-The levels of cell apoptotic related genes were detected by western blotting/figure-5/actin(miR-21 ).tif]

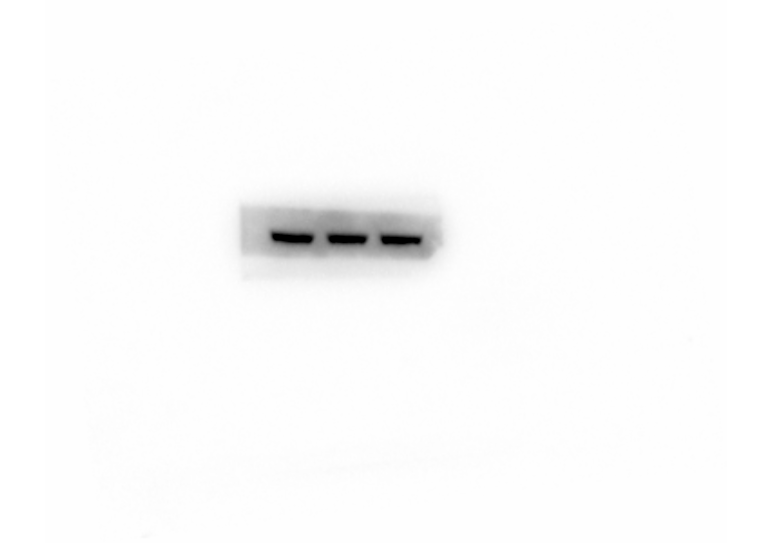

Supplement: S5 File — (ZIP) [file pone.0191616.s005.zip › Original data underlying the findings described in manuscript-The levels of cell apoptotic related genes were detected by western blotting/figure-5/actin(siR).tif]

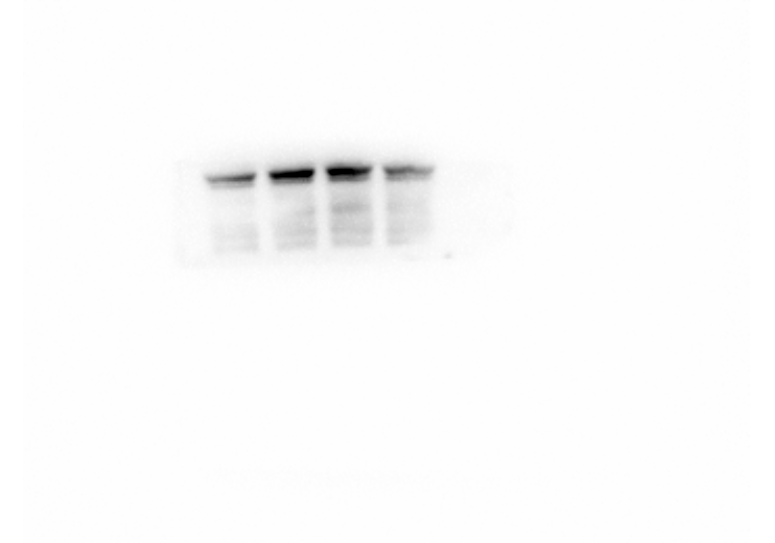

Supplement: S5 File — (ZIP) [file pone.0191616.s005.zip › Original data underlying the findings described in manuscript-The levels of cell apoptotic related genes were detected by western blotting/figure-6/PTEN.tif]

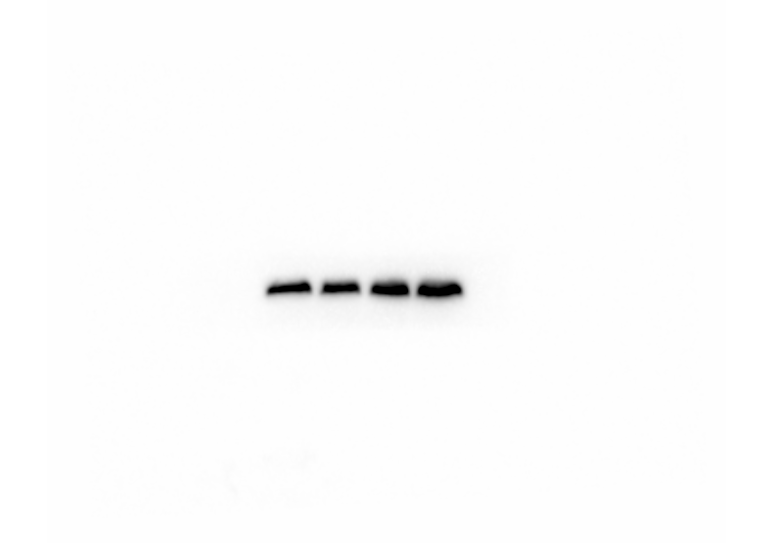

Supplement: S5 File — (ZIP) [file pone.0191616.s005.zip › Original data underlying the findings described in manuscript-The levels of cell apoptotic related genes were detected by western blotting/figure-6/actin.tif]

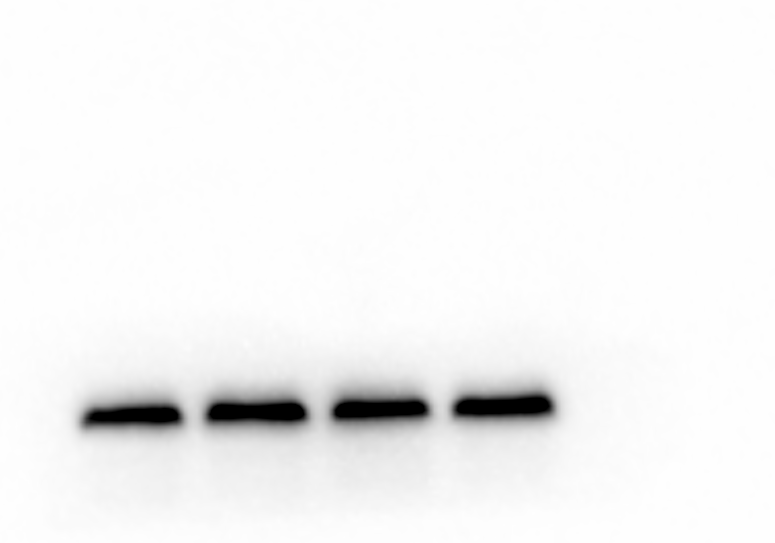

Supplement: S5 File — (ZIP) [file pone.0191616.s005.zip › Original data underlying the findings described in manuscript-The levels of cell apoptotic related genes were detected by western blotting/figure-6/caspase-3.tif]

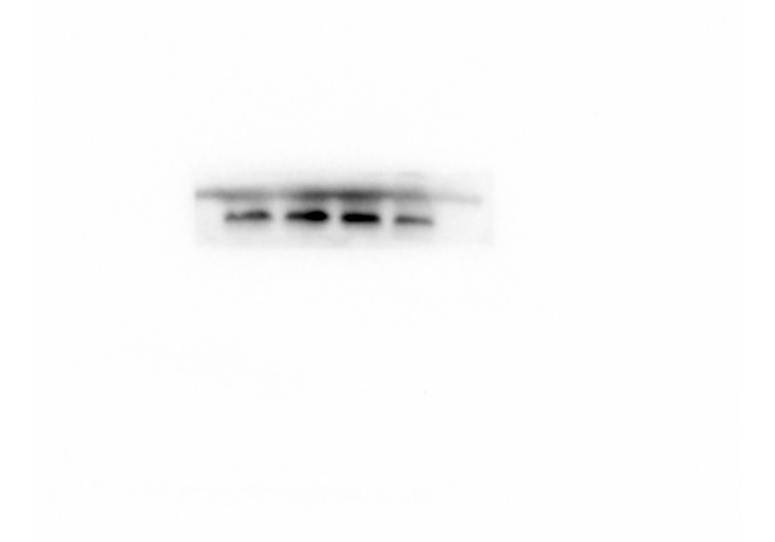

Supplement: S5 File — (ZIP) [file pone.0191616.s005.zip › Original data underlying the findings described in manuscript-The levels of cell apoptotic related genes were detected by western blotting/figure-6/cleaved caspase-3.tif]

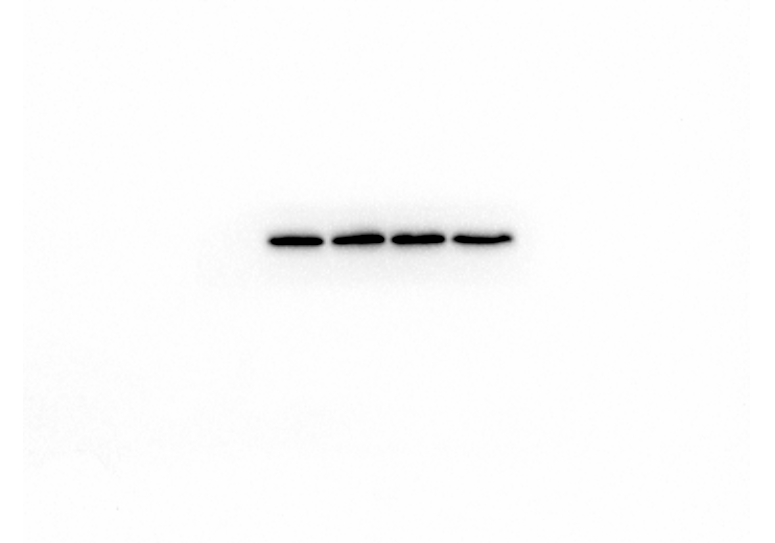

Supplement: S5 File — (ZIP) [file pone.0191616.s005.zip › Original data underlying the findings described in manuscript-The levels of cell apoptotic related genes were detected by western blotting/figure-7/Caspase-3.tif]

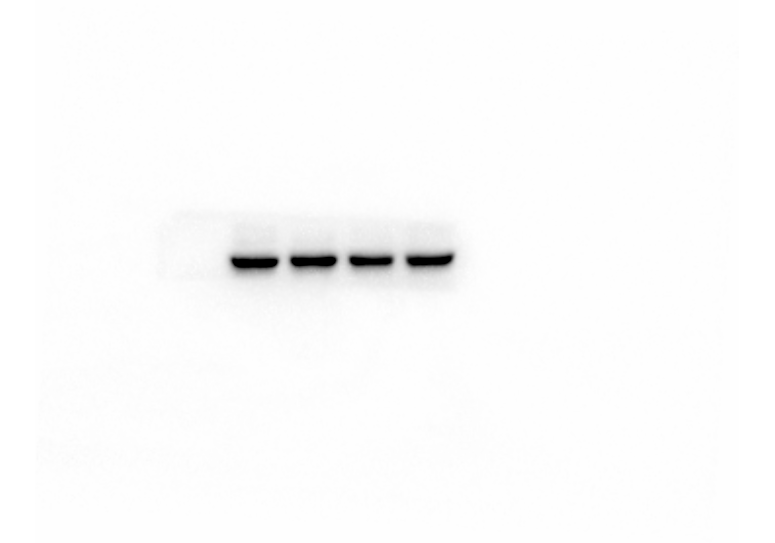

Supplement: S5 File — (ZIP) [file pone.0191616.s005.zip › Original data underlying the findings described in manuscript-The levels of cell apoptotic related genes were detected by western blotting/figure-7/actin.tif]

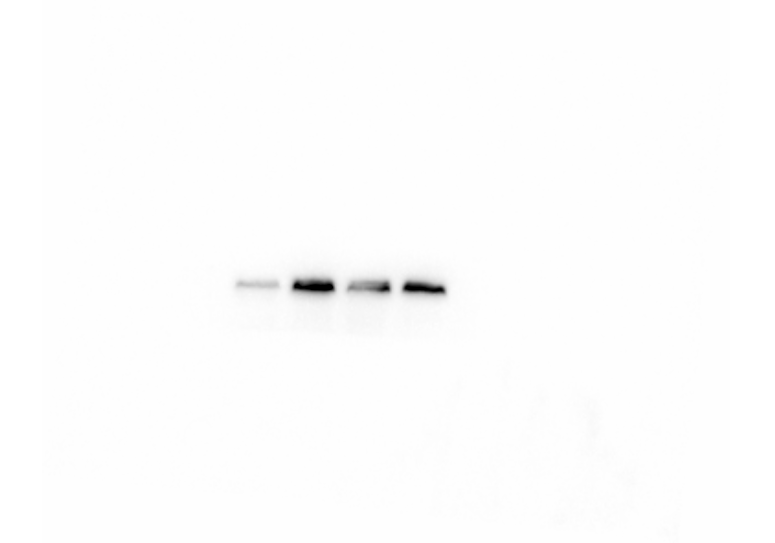

Supplement: S5 File — (ZIP) [file pone.0191616.s005.zip › Original data underlying the findings described in manuscript-The levels of cell apoptotic related genes were detected by western blotting/figure-7/cleaved caspse-3.tif]

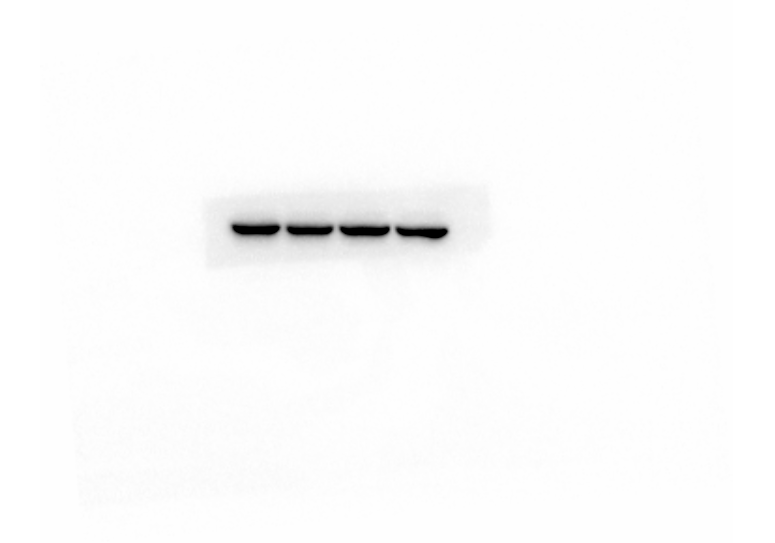

Supplement: S5 File — (ZIP) [file pone.0191616.s005.zip › Original data underlying the findings described in manuscript-The levels of cell apoptotic related genes were detected by western blotting/figure-8/GAPDH(ly294002).tif]

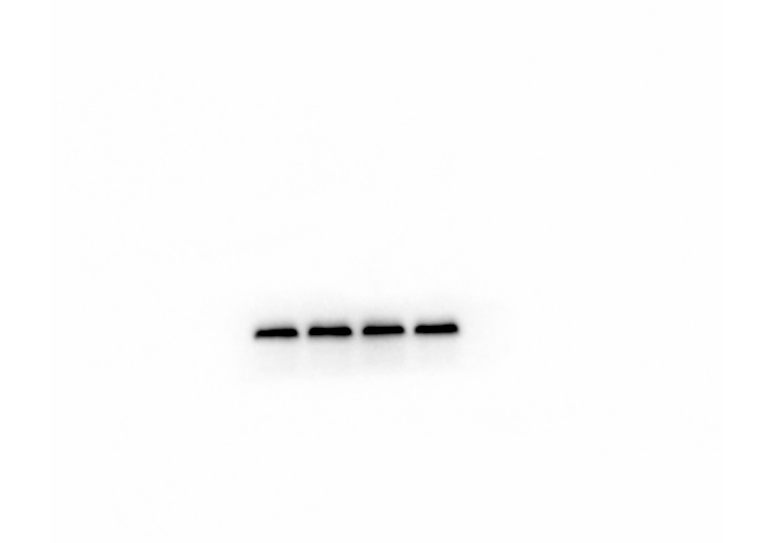

Supplement: S5 File — (ZIP) [file pone.0191616.s005.zip › Original data underlying the findings described in manuscript-The levels of cell apoptotic related genes were detected by western blotting/figure-8/GAPDH(miR-21 inhibiter).tif]

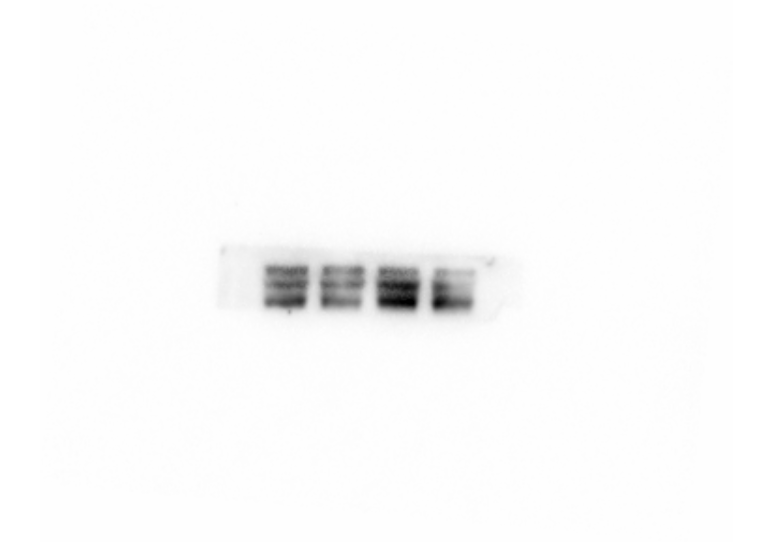

Supplement: S5 File — (ZIP) [file pone.0191616.s005.zip › Original data underlying the findings described in manuscript-The levels of cell apoptotic related genes were detected by western blotting/figure-8/P-AKT(LY294002).tif]

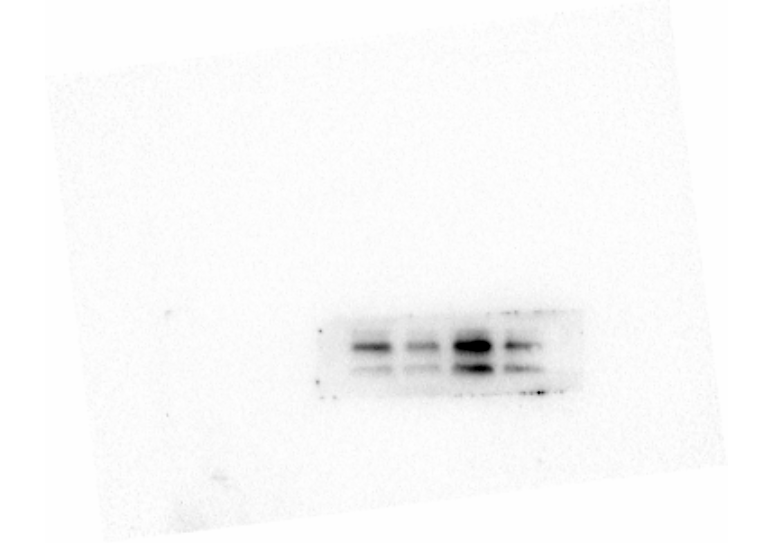

Supplement: S5 File — (ZIP) [file pone.0191616.s005.zip › Original data underlying the findings described in manuscript-The levels of cell apoptotic related genes were detected by western blotting/figure-8/P-AKT(miR-21 inhibiter).tif]

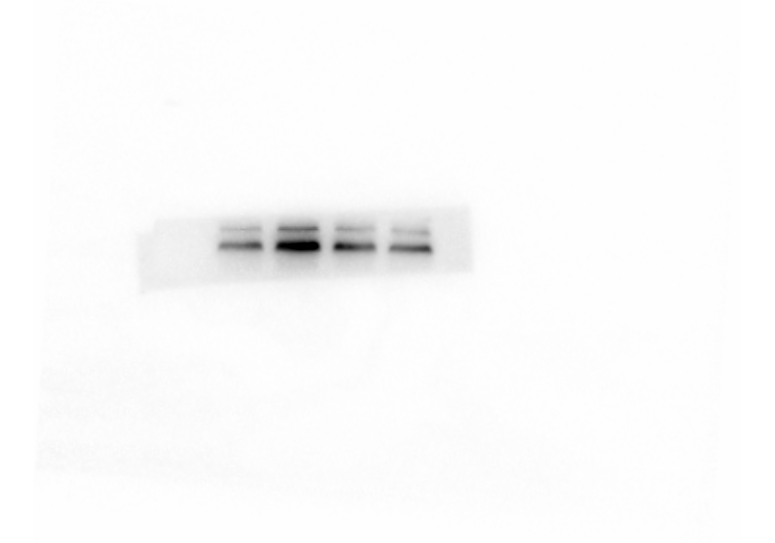

Supplement: S5 File — (ZIP) [file pone.0191616.s005.zip › Original data underlying the findings described in manuscript-The levels of cell apoptotic related genes were detected by western blotting/figure-8/PTEN(ly294002).tif]

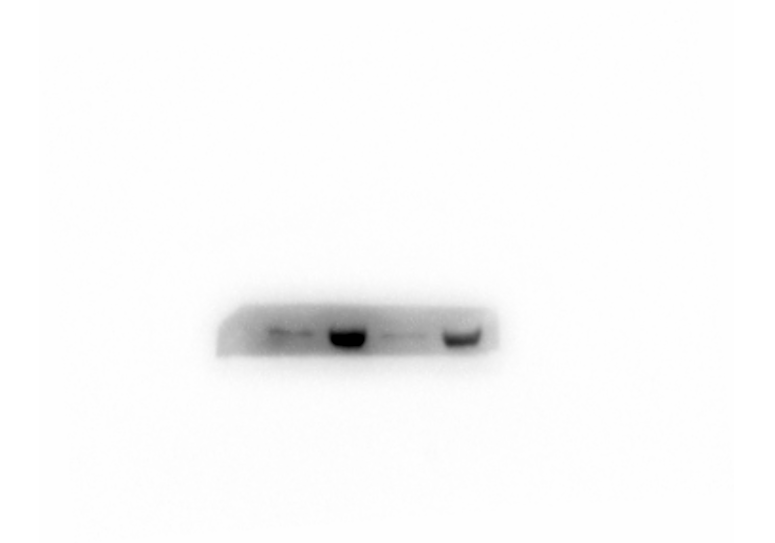

Supplement: S5 File — (ZIP) [file pone.0191616.s005.zip › Original data underlying the findings described in manuscript-The levels of cell apoptotic related genes were detected by western blotting/figure-8/PTEN(miR-21 inhibiter).tif]

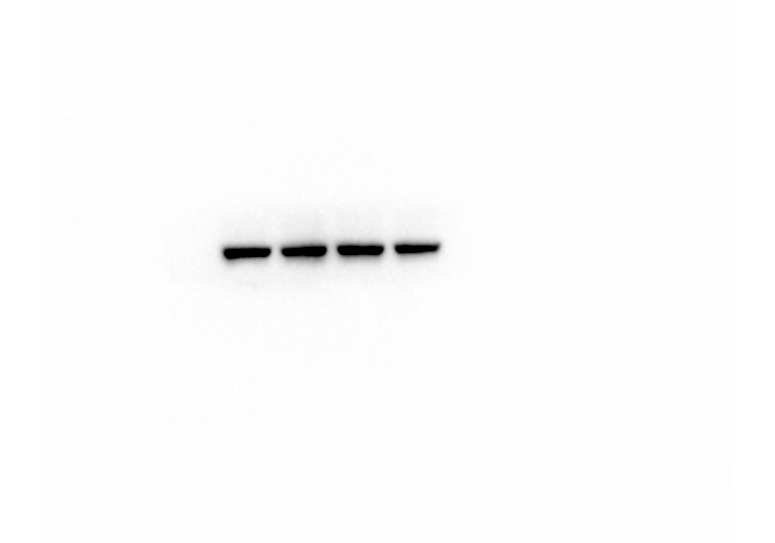

Supplement: S5 File — (ZIP) [file pone.0191616.s005.zip › Original data underlying the findings described in manuscript-The levels of cell apoptotic related genes were detected by western blotting/figure-8/T-AKT(ly294002).tif]

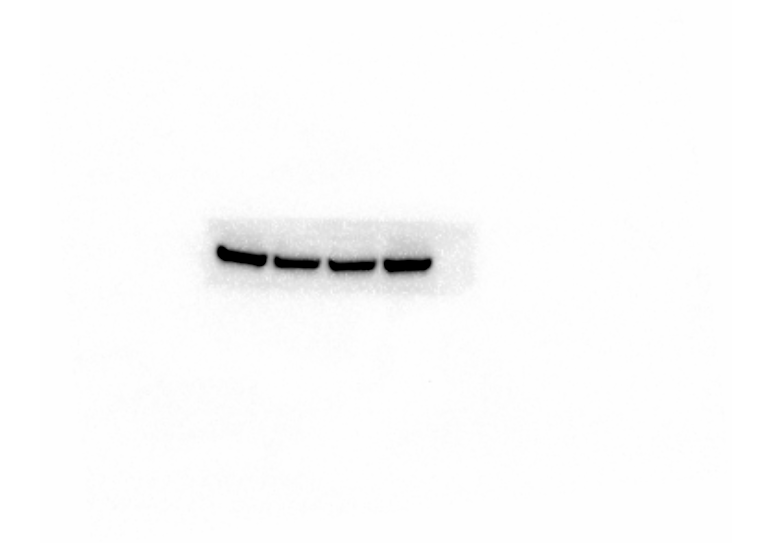

Supplement: S5 File — (ZIP) [file pone.0191616.s005.zip › Original data underlying the findings described in manuscript-The levels of cell apoptotic related genes were detected by western blotting/figure-8/T-AKT(miR-21 inhibiter).tif]

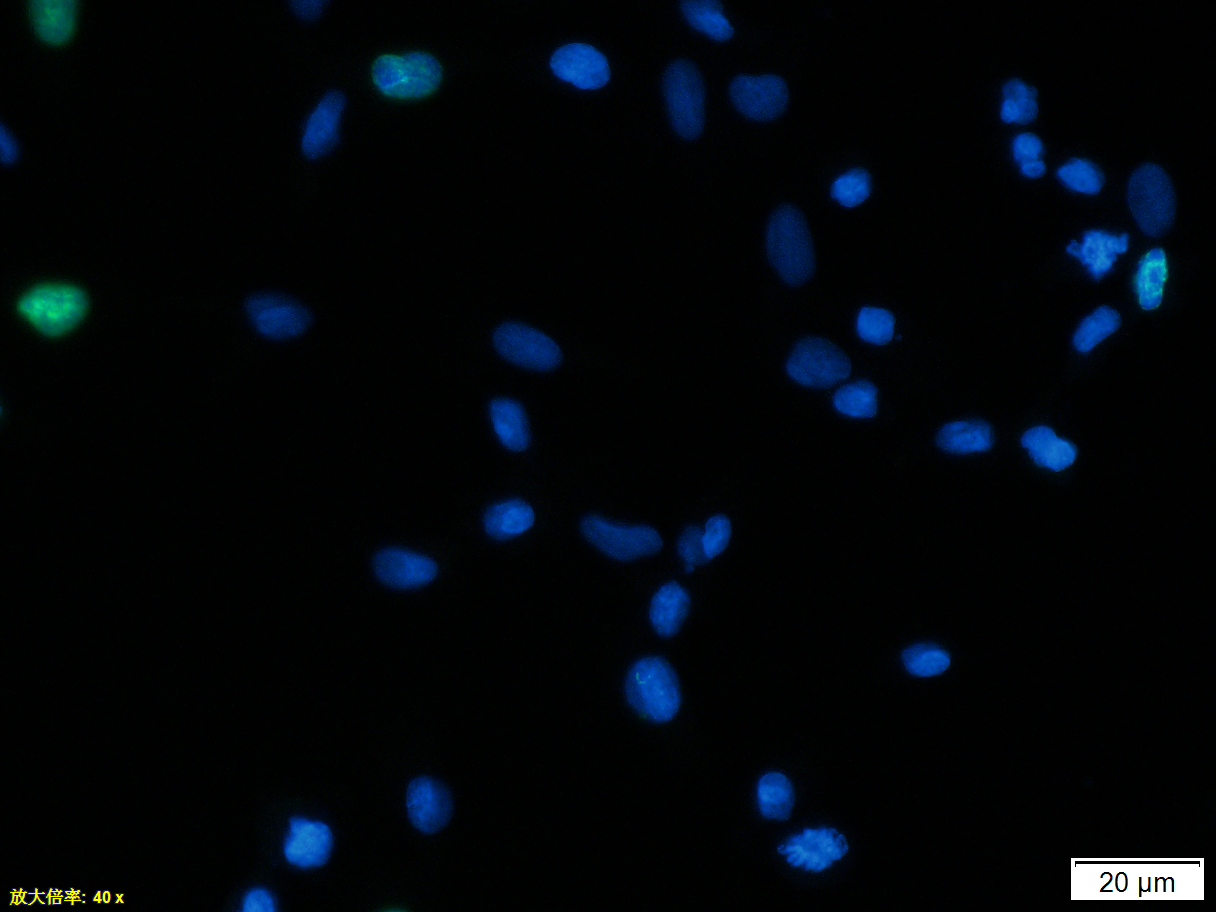

Supplement: S6 File — (ZIP) [file pone.0191616.s006.zip › Original data underlying the findings described in manuscript-TUNEL staining for detecting the apoptosis of CSCs-1/H-Exo group/fig_01.tif]

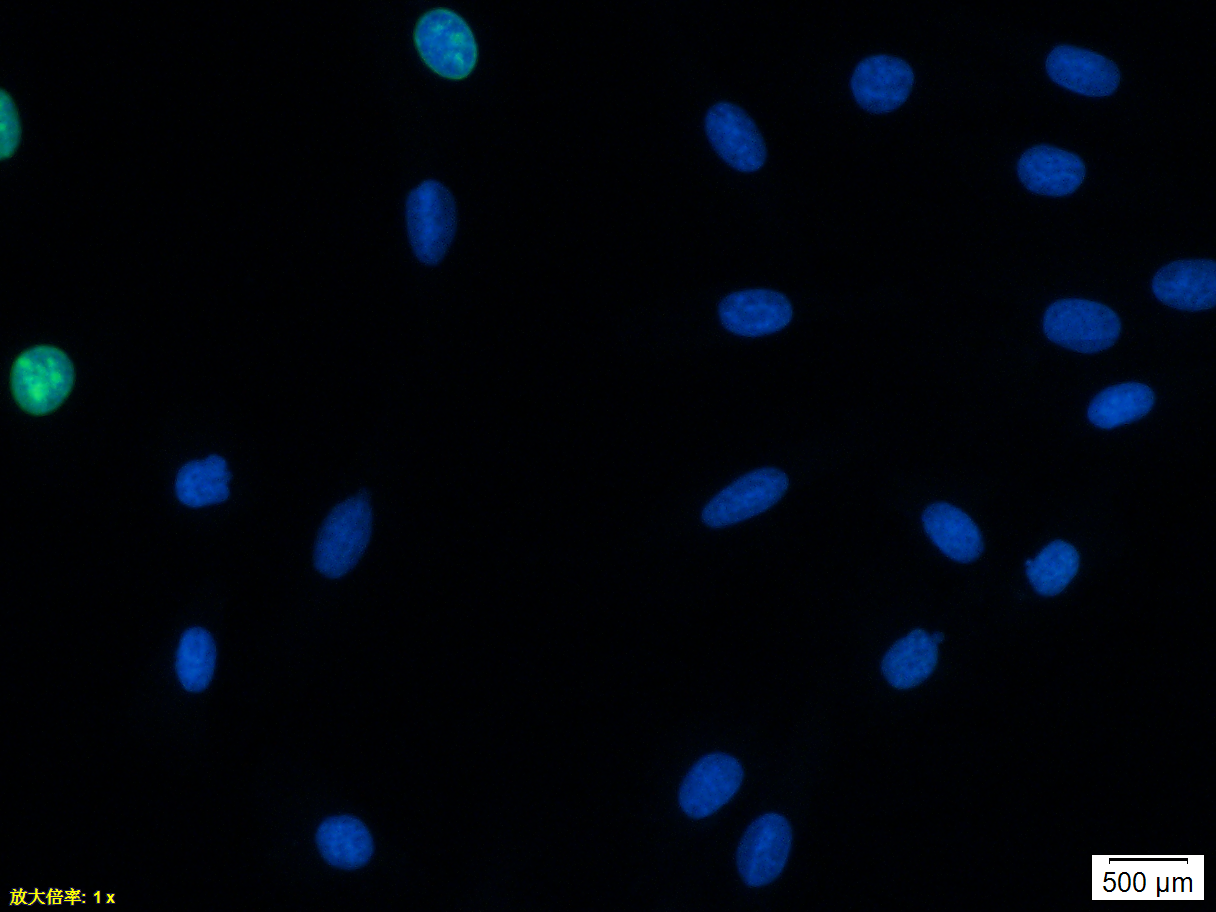

Supplement: S6 File — (ZIP) [file pone.0191616.s006.zip › Original data underlying the findings described in manuscript-TUNEL staining for detecting the apoptosis of CSCs-1/H-Exo group/fig_02.tif]

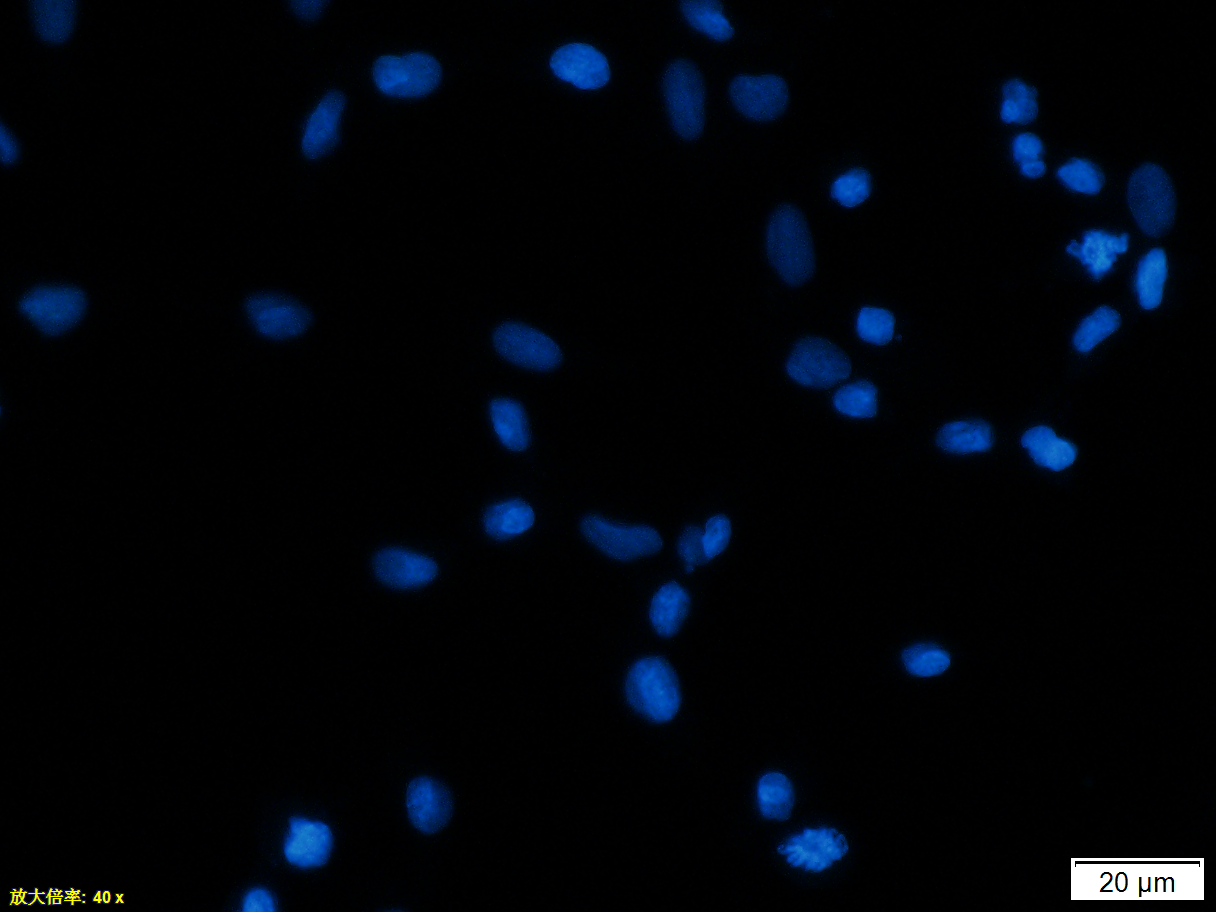

Supplement: S6 File — (ZIP) [file pone.0191616.s006.zip › Original data underlying the findings described in manuscript-TUNEL staining for detecting the apoptosis of CSCs-1/H-Exo group/fig_1-1.tif]

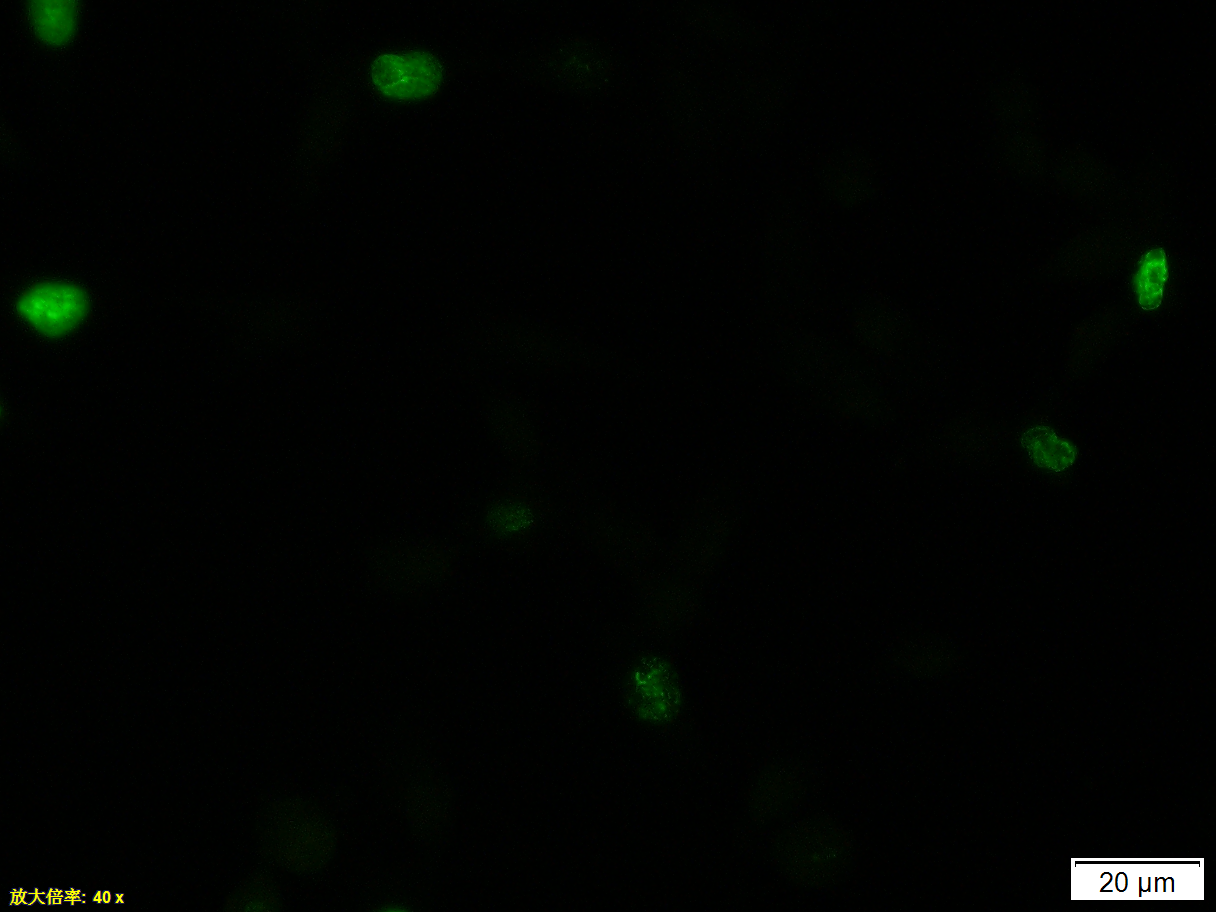

Supplement: S6 File — (ZIP) [file pone.0191616.s006.zip › Original data underlying the findings described in manuscript-TUNEL staining for detecting the apoptosis of CSCs-1/H-Exo group/fig_1-2.tif]

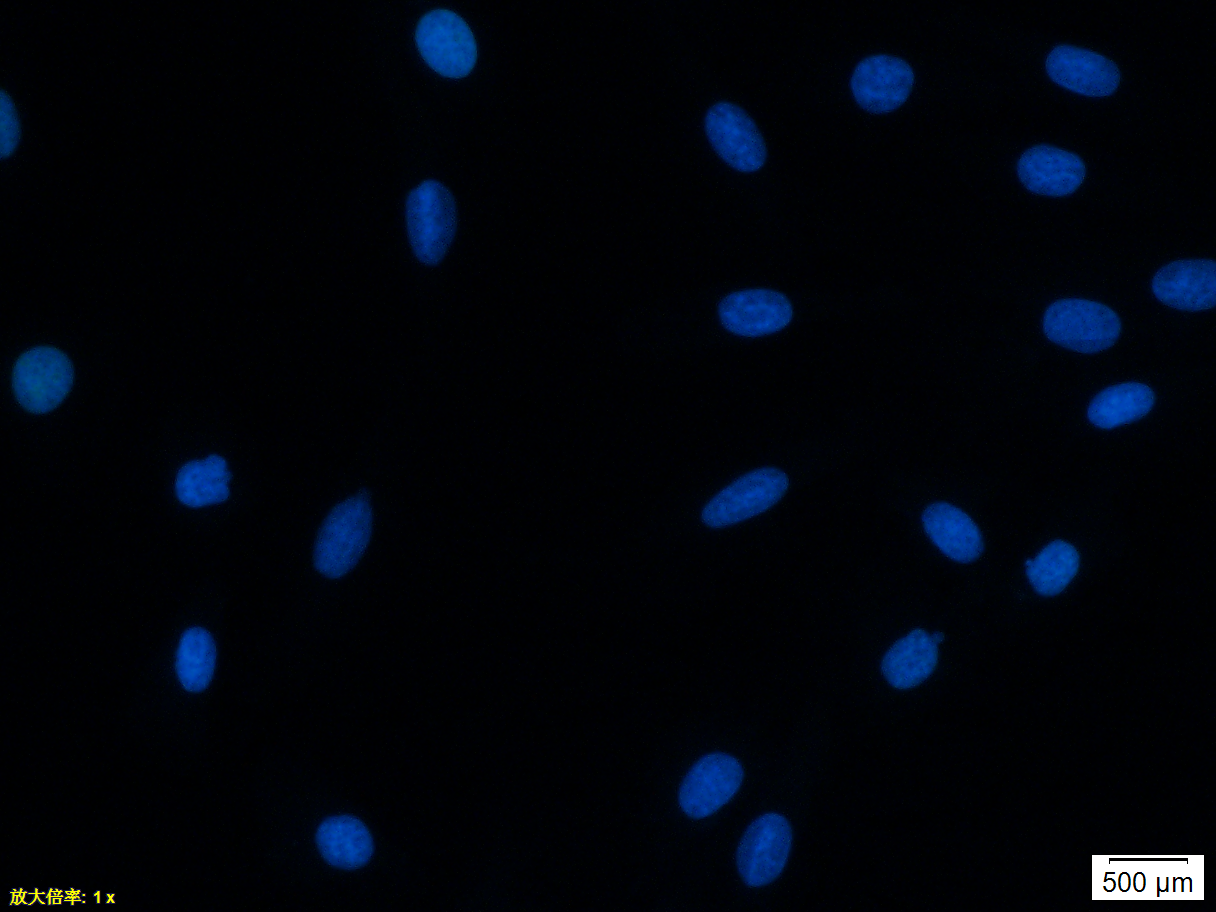

Supplement: S6 File — (ZIP) [file pone.0191616.s006.zip › Original data underlying the findings described in manuscript-TUNEL staining for detecting the apoptosis of CSCs-1/H-Exo group/fig_2-1.tif]

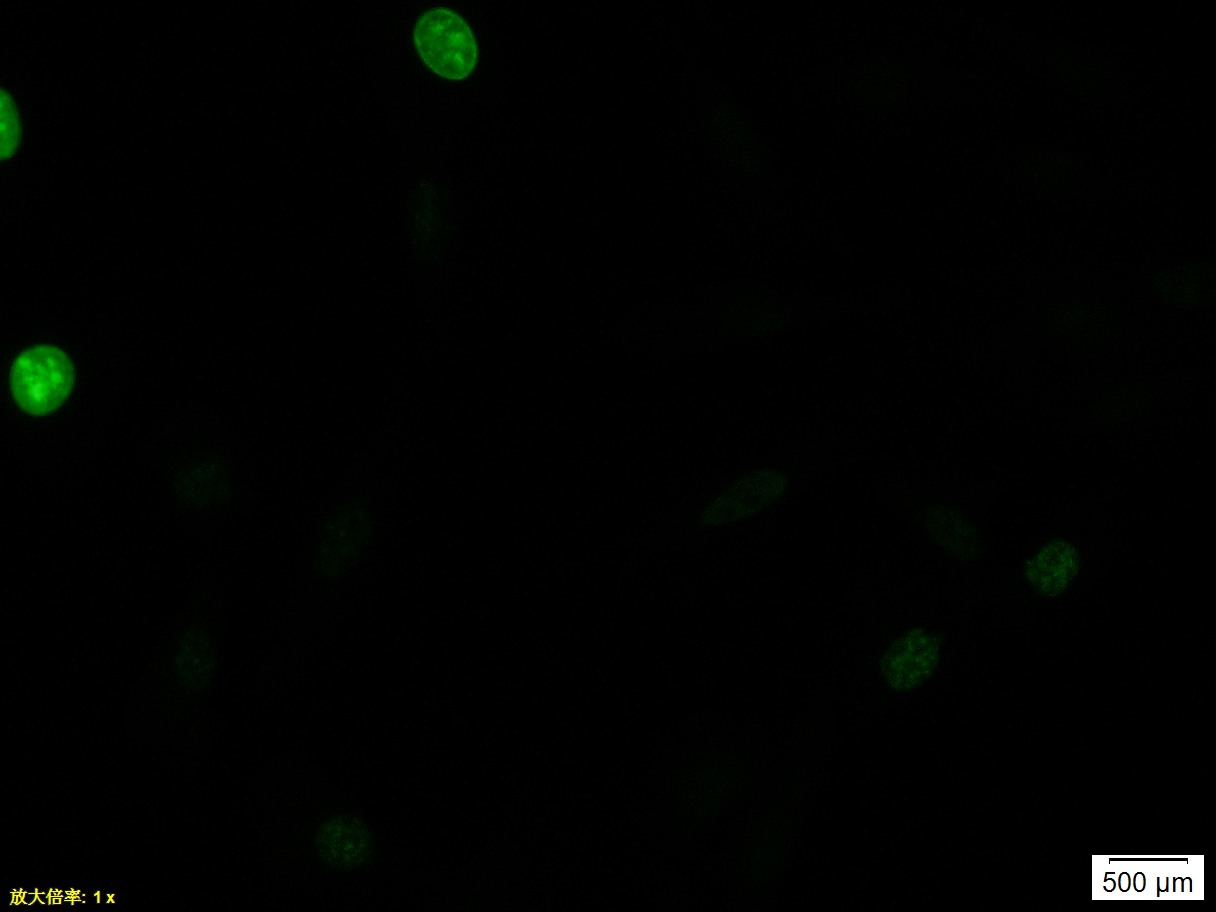

Supplement: S6 File — (ZIP) [file pone.0191616.s006.zip › Original data underlying the findings described in manuscript-TUNEL staining for detecting the apoptosis of CSCs-1/H-Exo group/fig_2-2.tif]

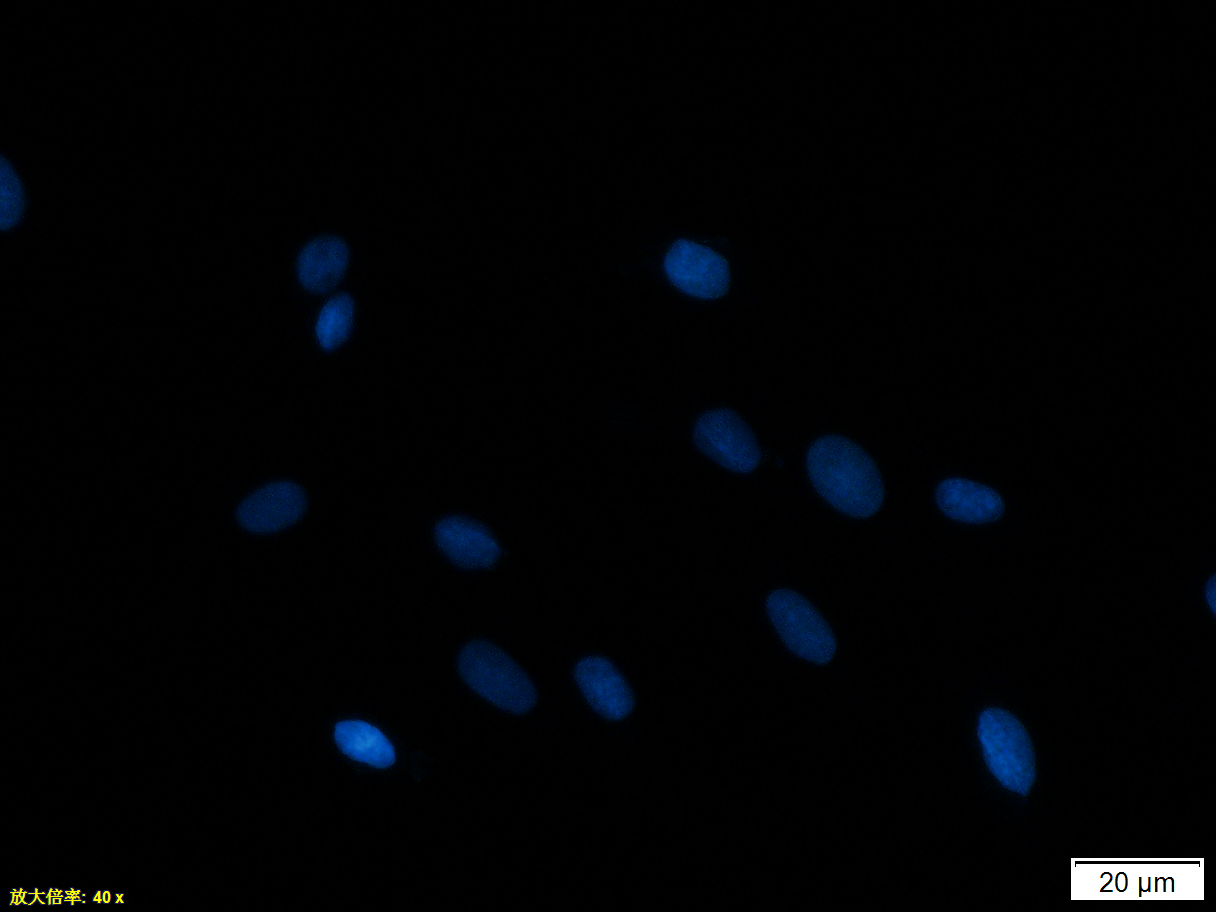

Supplement: S6 File — (ZIP) [file pone.0191616.s006.zip › Original data underlying the findings described in manuscript-TUNEL staining for detecting the apoptosis of CSCs-1/H-Exo group/fig_3-1.tif]

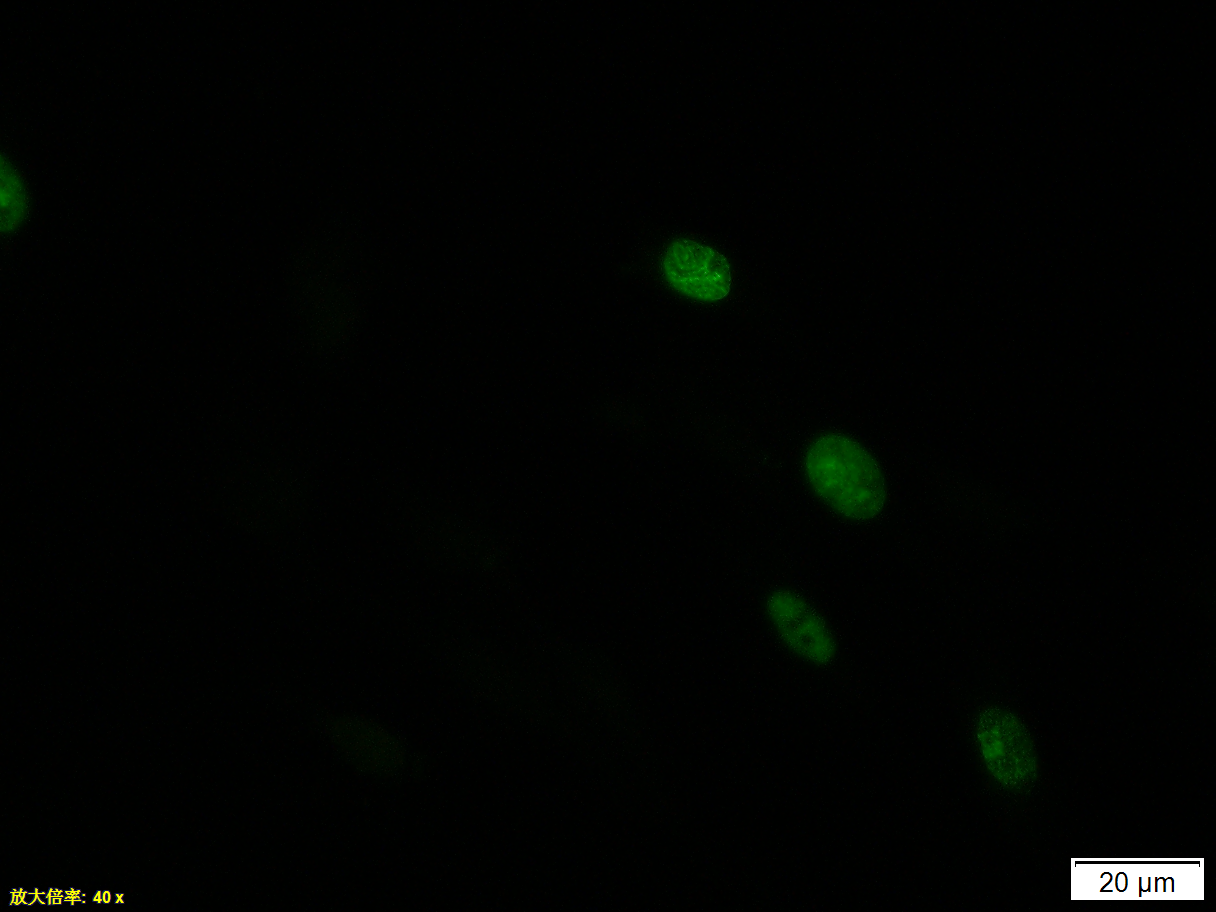

Supplement: S6 File — (ZIP) [file pone.0191616.s006.zip › Original data underlying the findings described in manuscript-TUNEL staining for detecting the apoptosis of CSCs-1/H-Exo group/fig_3-2.tif]

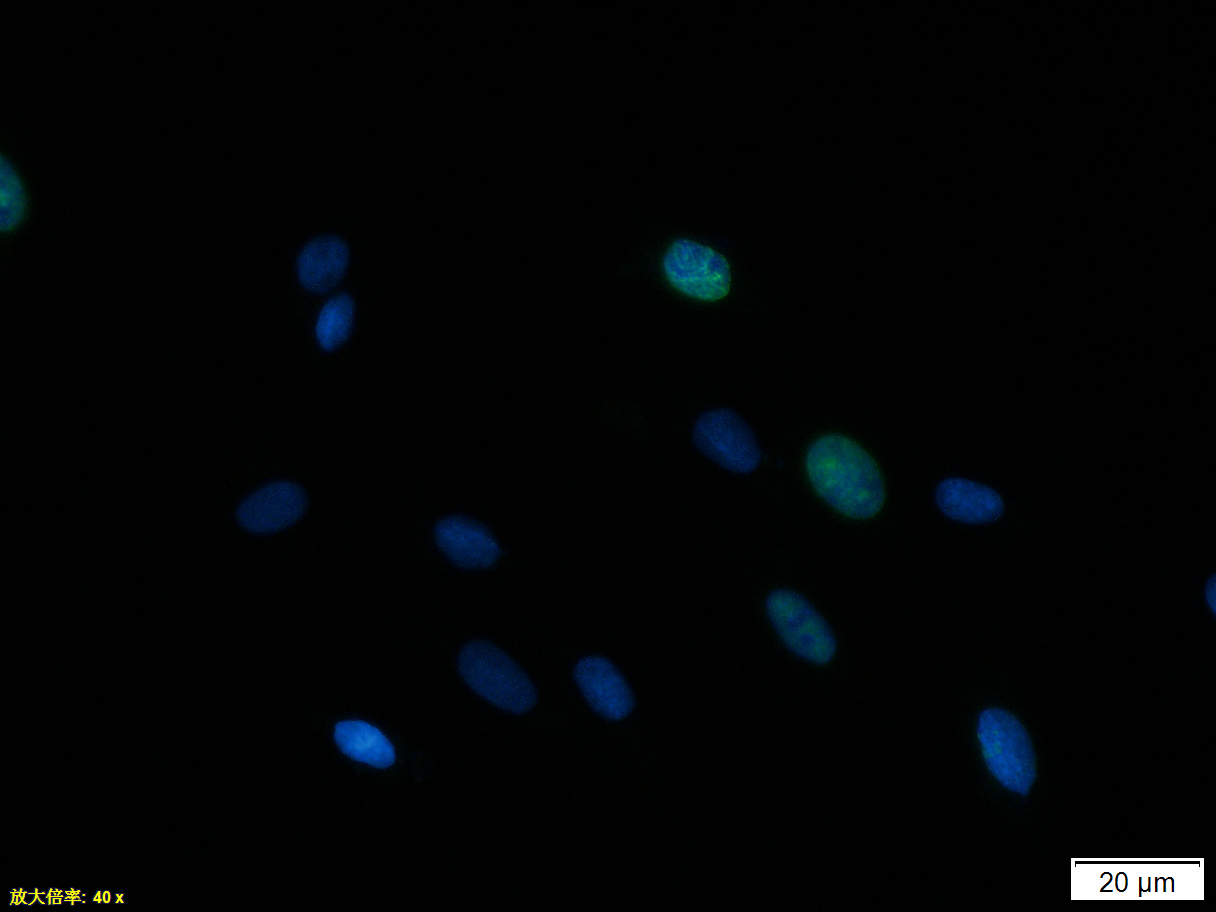

Supplement: S6 File — (ZIP) [file pone.0191616.s006.zip › Original data underlying the findings described in manuscript-TUNEL staining for detecting the apoptosis of CSCs-1/H-Exo group/fig_3.tif]

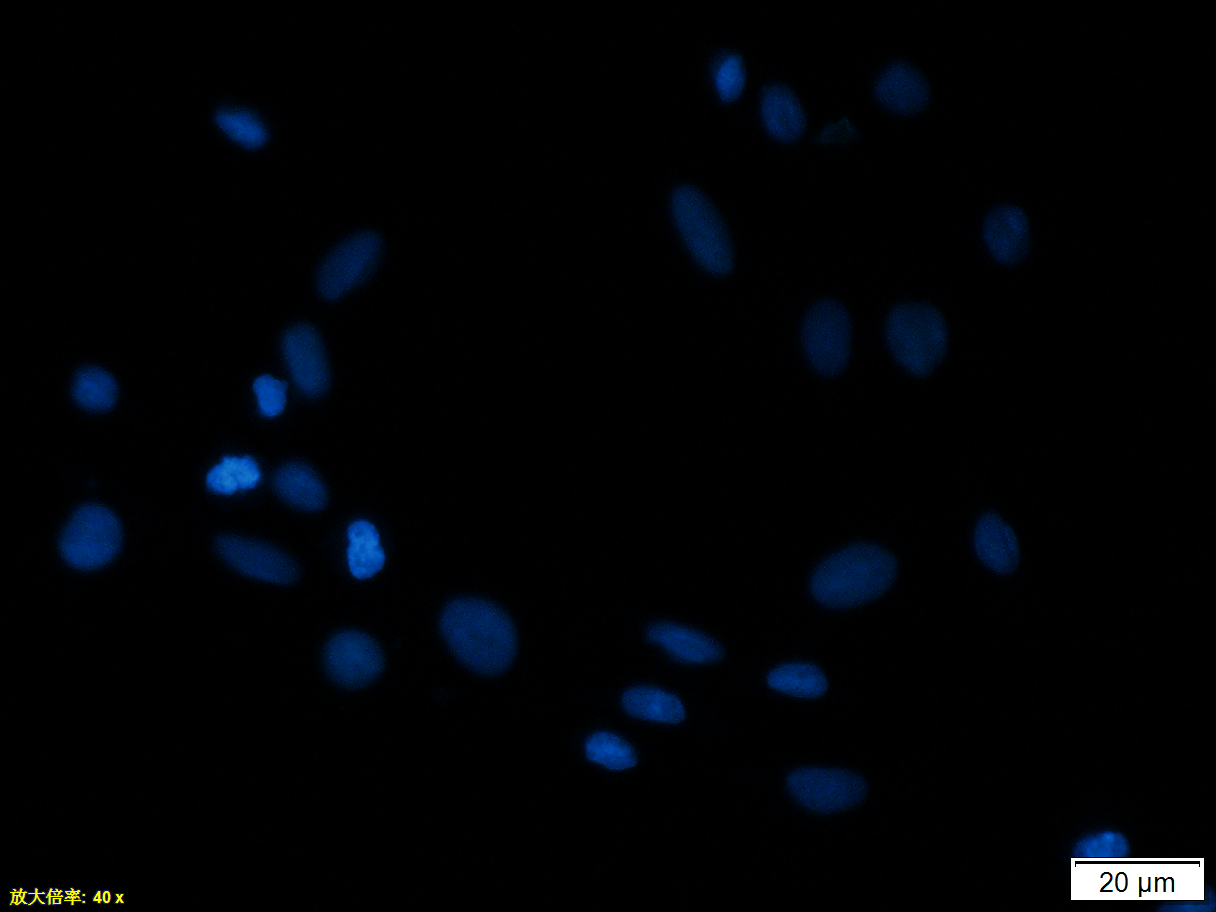

Supplement: S6 File — (ZIP) [file pone.0191616.s006.zip › Original data underlying the findings described in manuscript-TUNEL staining for detecting the apoptosis of CSCs-1/H-Exo group/fig_4-1.tif]

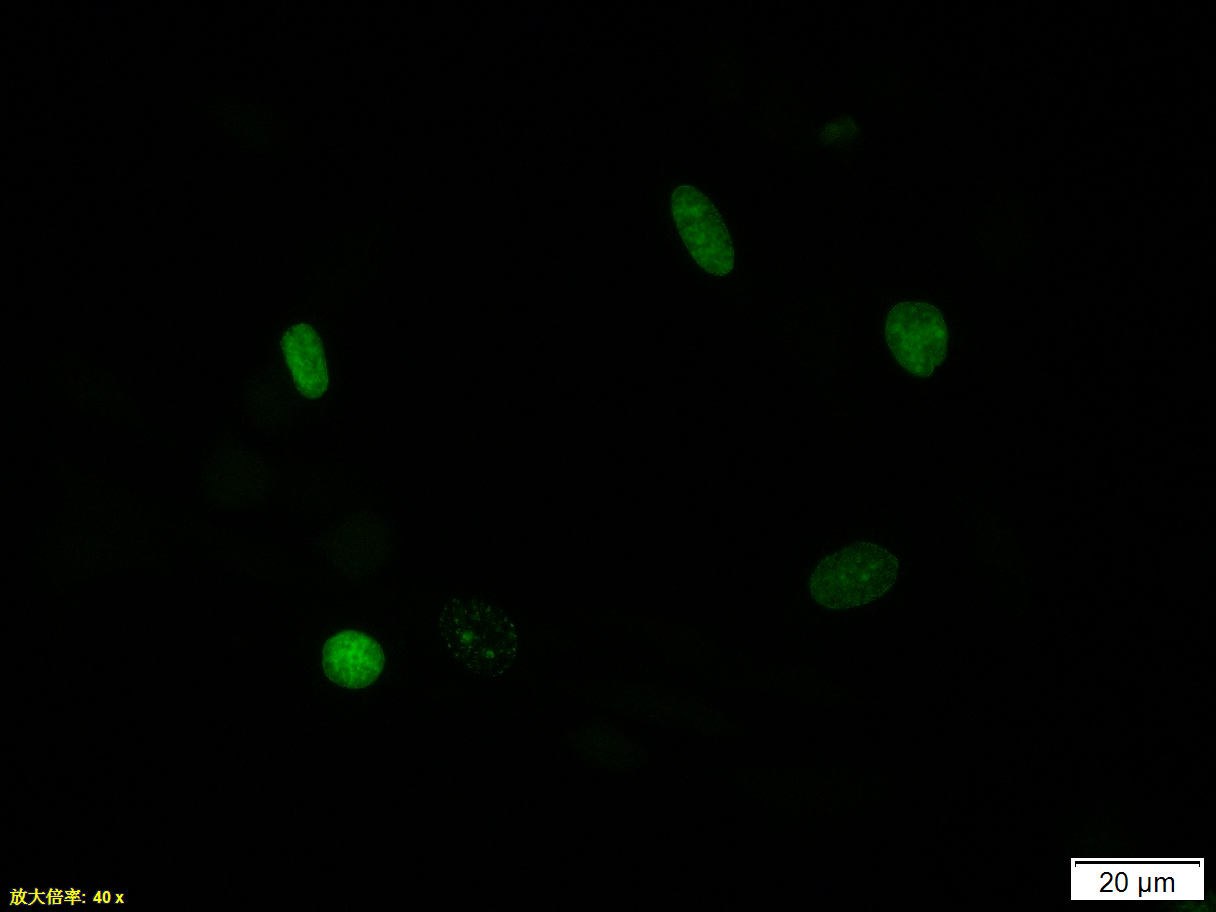

Supplement: S6 File — (ZIP) [file pone.0191616.s006.zip › Original data underlying the findings described in manuscript-TUNEL staining for detecting the apoptosis of CSCs-1/H-Exo group/fig_4-2.tif]

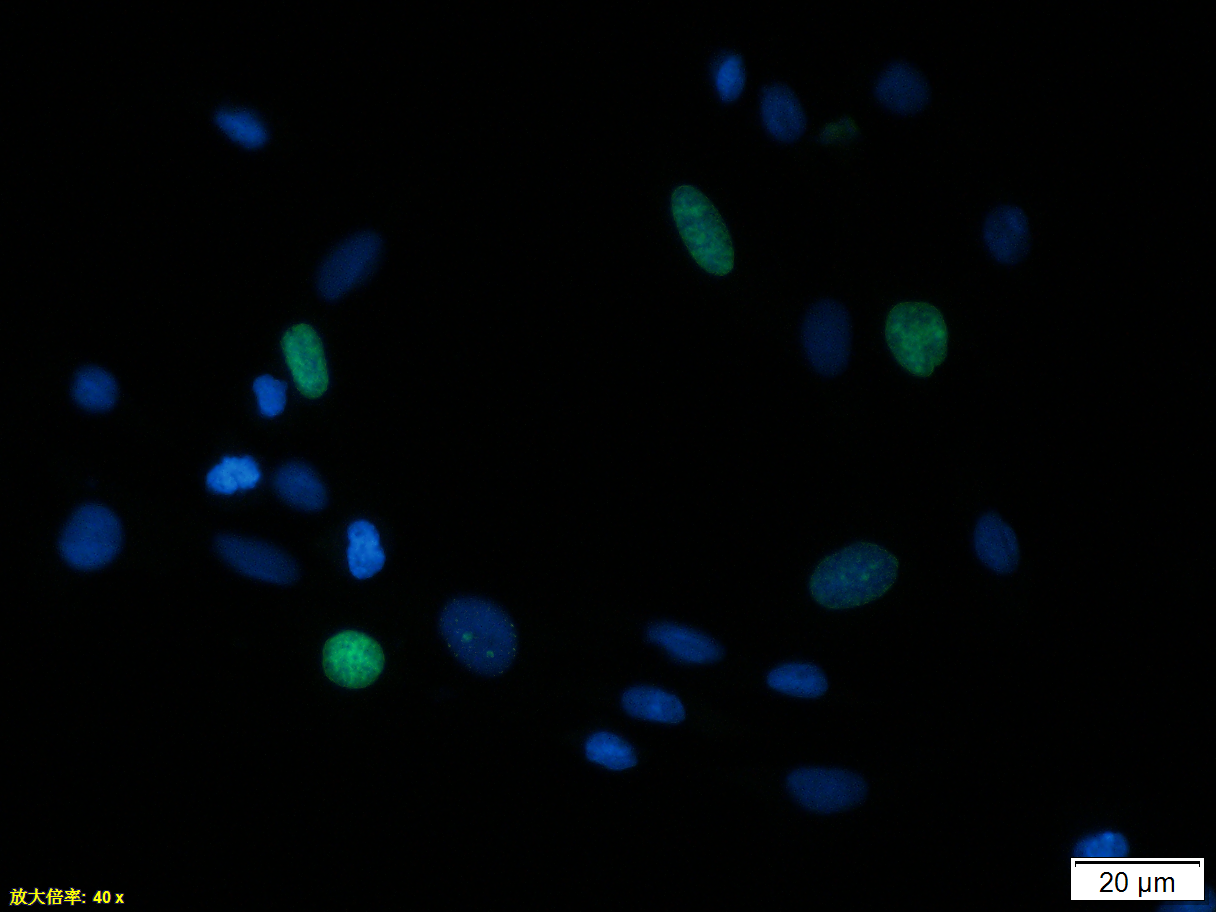

Supplement: S6 File — (ZIP) [file pone.0191616.s006.zip › Original data underlying the findings described in manuscript-TUNEL staining for detecting the apoptosis of CSCs-1/H-Exo group/fig_4.tif]

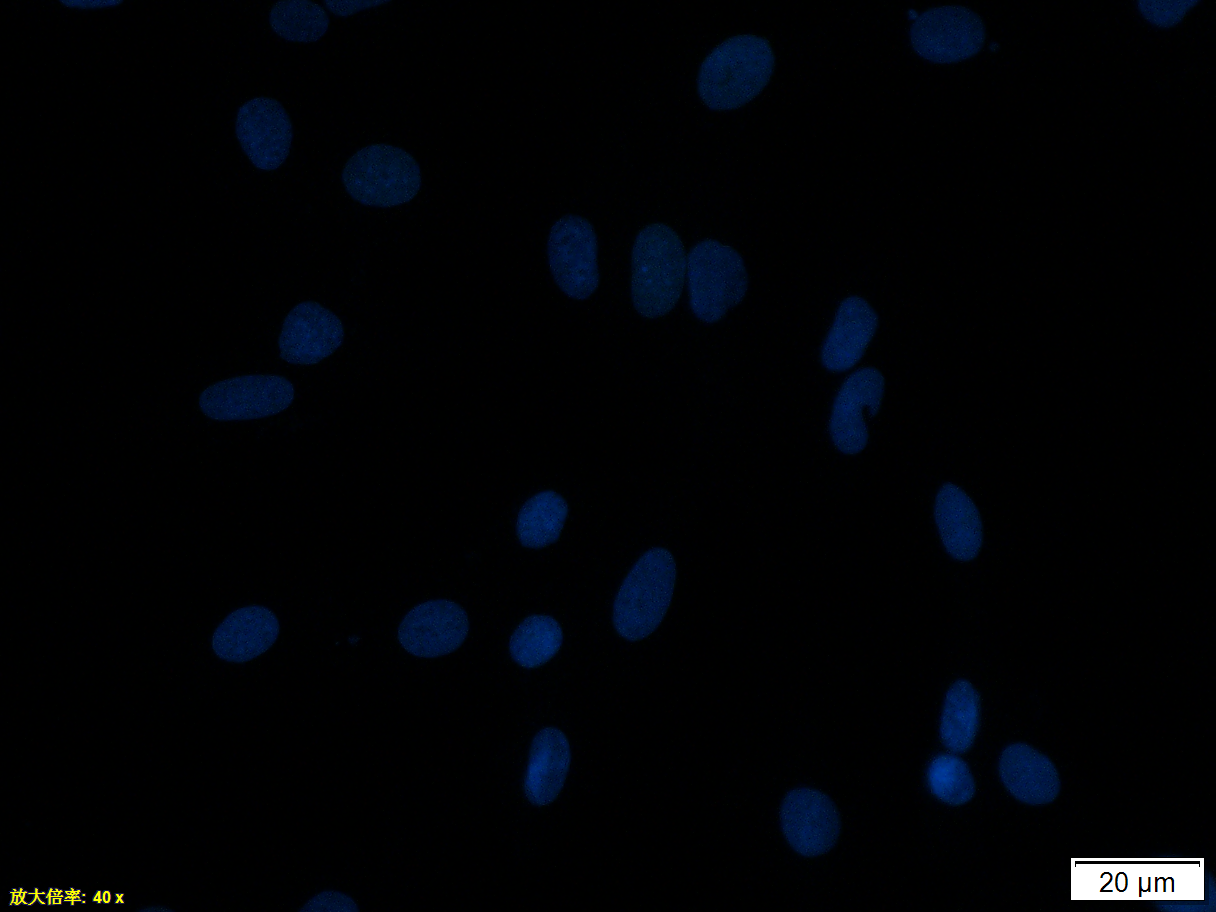

Supplement: S6 File — (ZIP) [file pone.0191616.s006.zip › Original data underlying the findings described in manuscript-TUNEL staining for detecting the apoptosis of CSCs-1/H-Exo group/fig_5-1.tif]

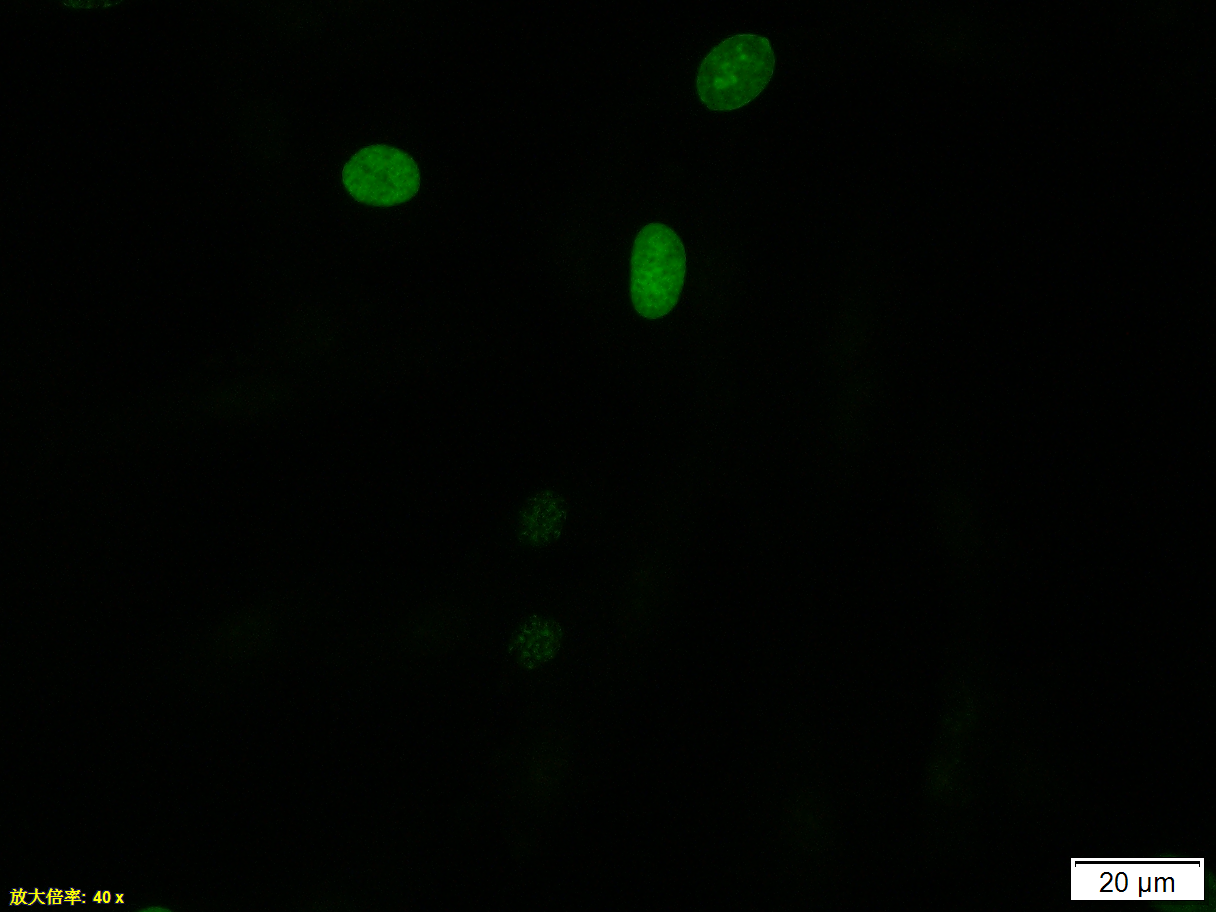

Supplement: S6 File — (ZIP) [file pone.0191616.s006.zip › Original data underlying the findings described in manuscript-TUNEL staining for detecting the apoptosis of CSCs-1/H-Exo group/fig_5-2.tif]

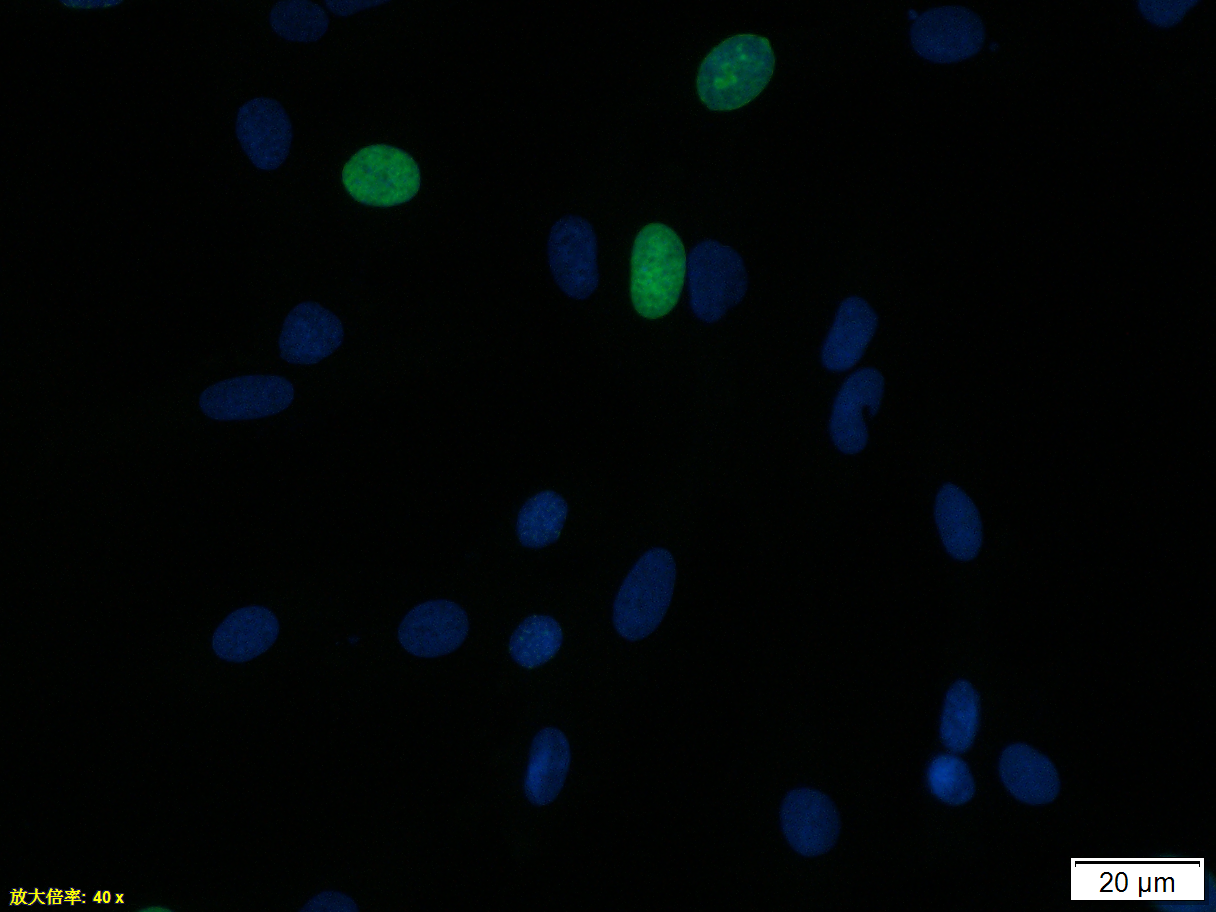

Supplement: S6 File — (ZIP) [file pone.0191616.s006.zip › Original data underlying the findings described in manuscript-TUNEL staining for detecting the apoptosis of CSCs-1/H-Exo group/fig_5.tif]

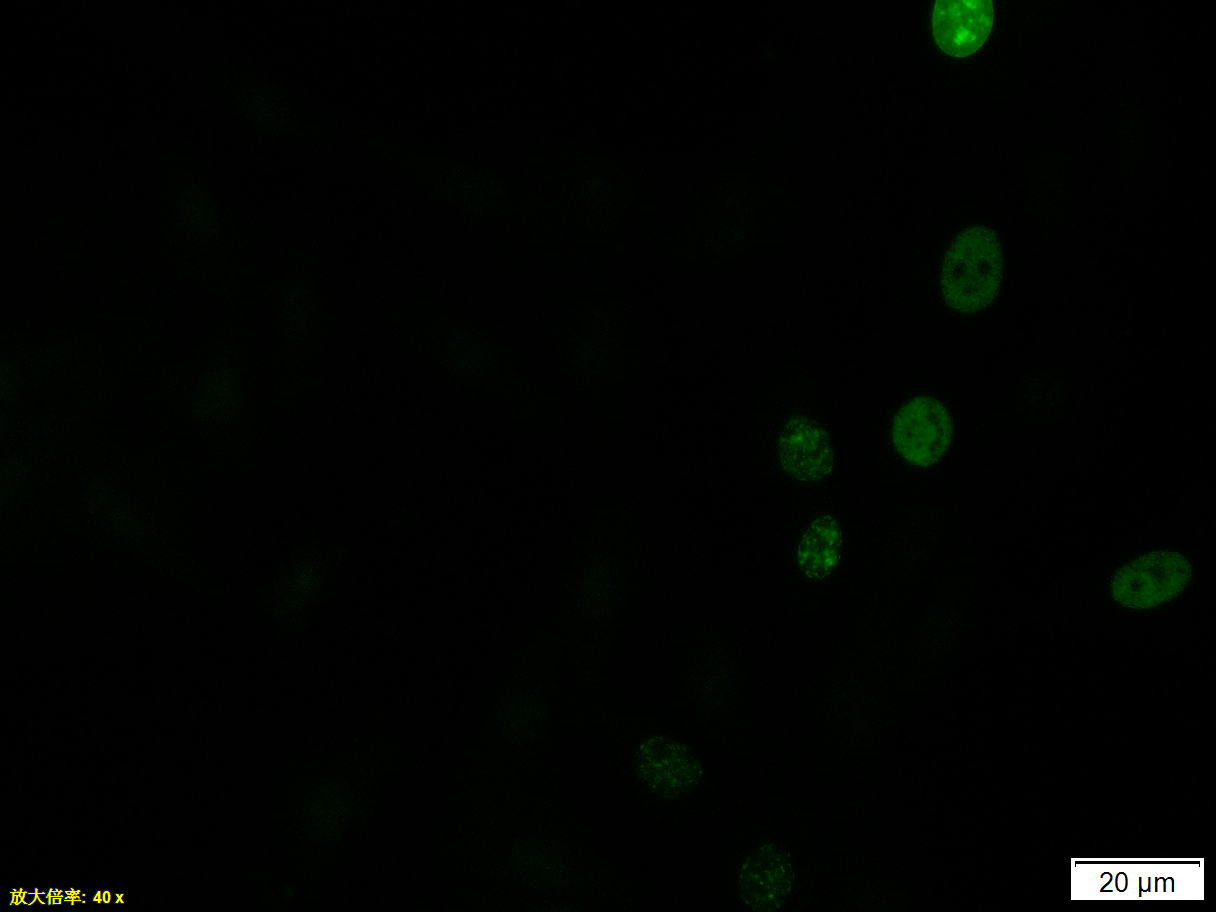

Supplement: S6 File — (ZIP) [file pone.0191616.s006.zip › Original data underlying the findings described in manuscript-TUNEL staining for detecting the apoptosis of CSCs-1/H-Exo group/fig_6-1.tif]

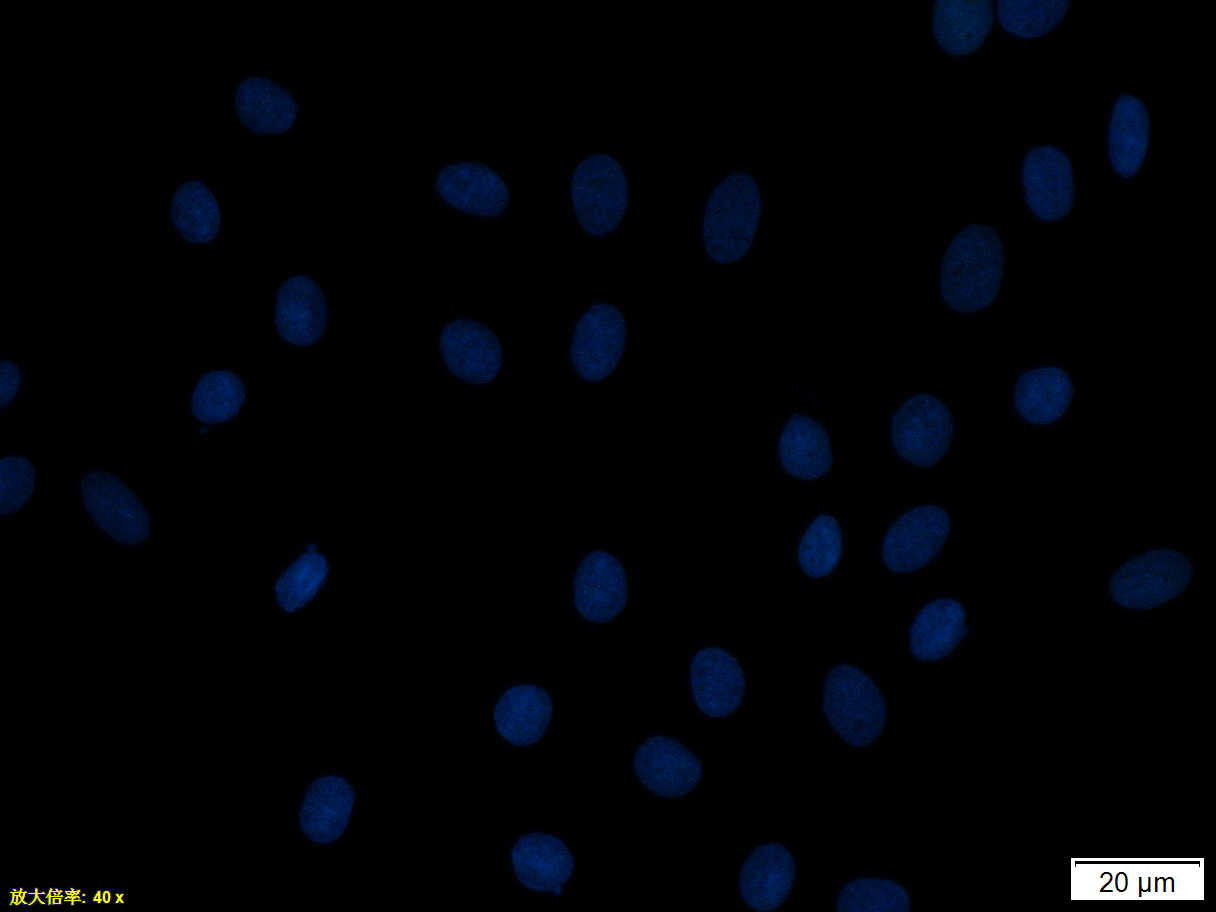

Supplement: S6 File — (ZIP) [file pone.0191616.s006.zip › Original data underlying the findings described in manuscript-TUNEL staining for detecting the apoptosis of CSCs-1/H-Exo group/fig_6-2.tif]

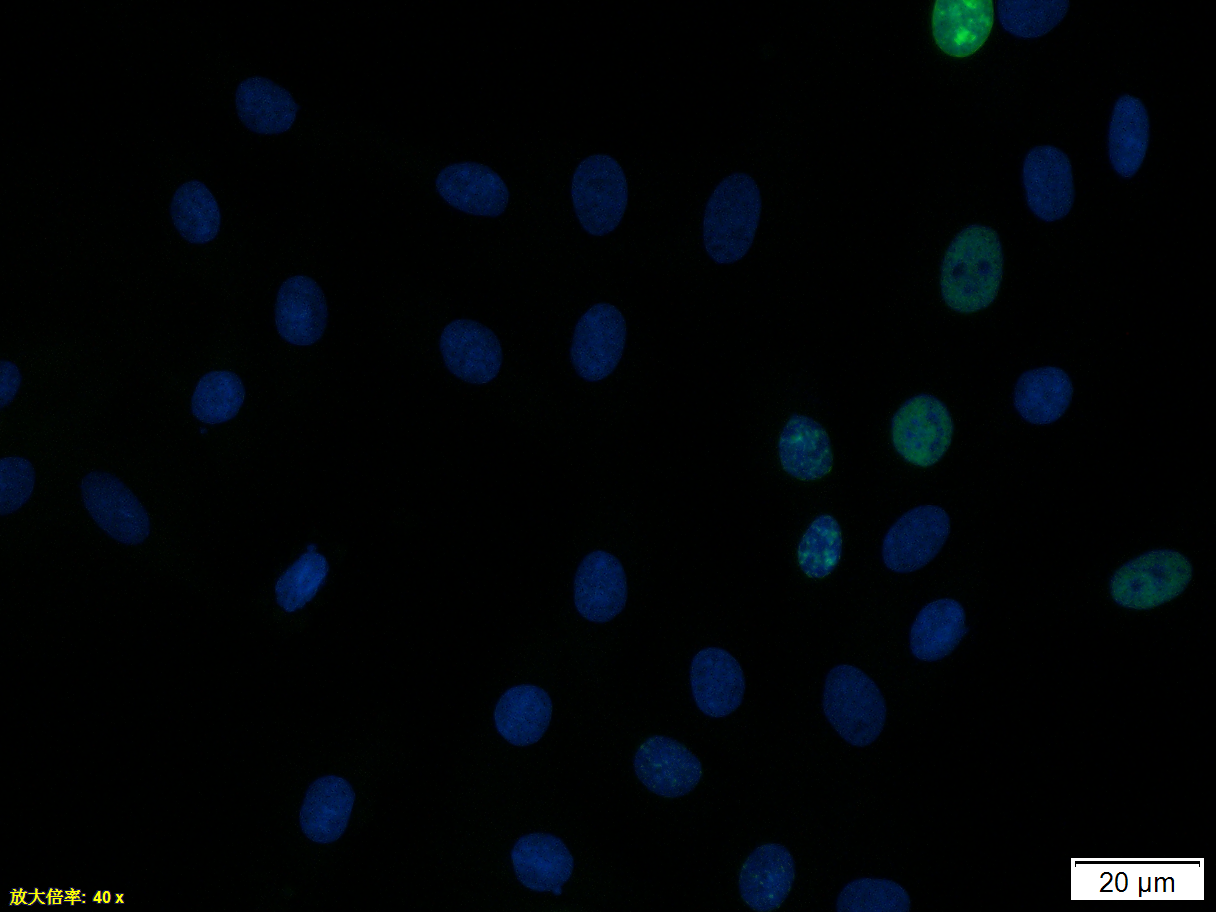

Supplement: S6 File — (ZIP) [file pone.0191616.s006.zip › Original data underlying the findings described in manuscript-TUNEL staining for detecting the apoptosis of CSCs-1/H-Exo group/fig_6.tif]

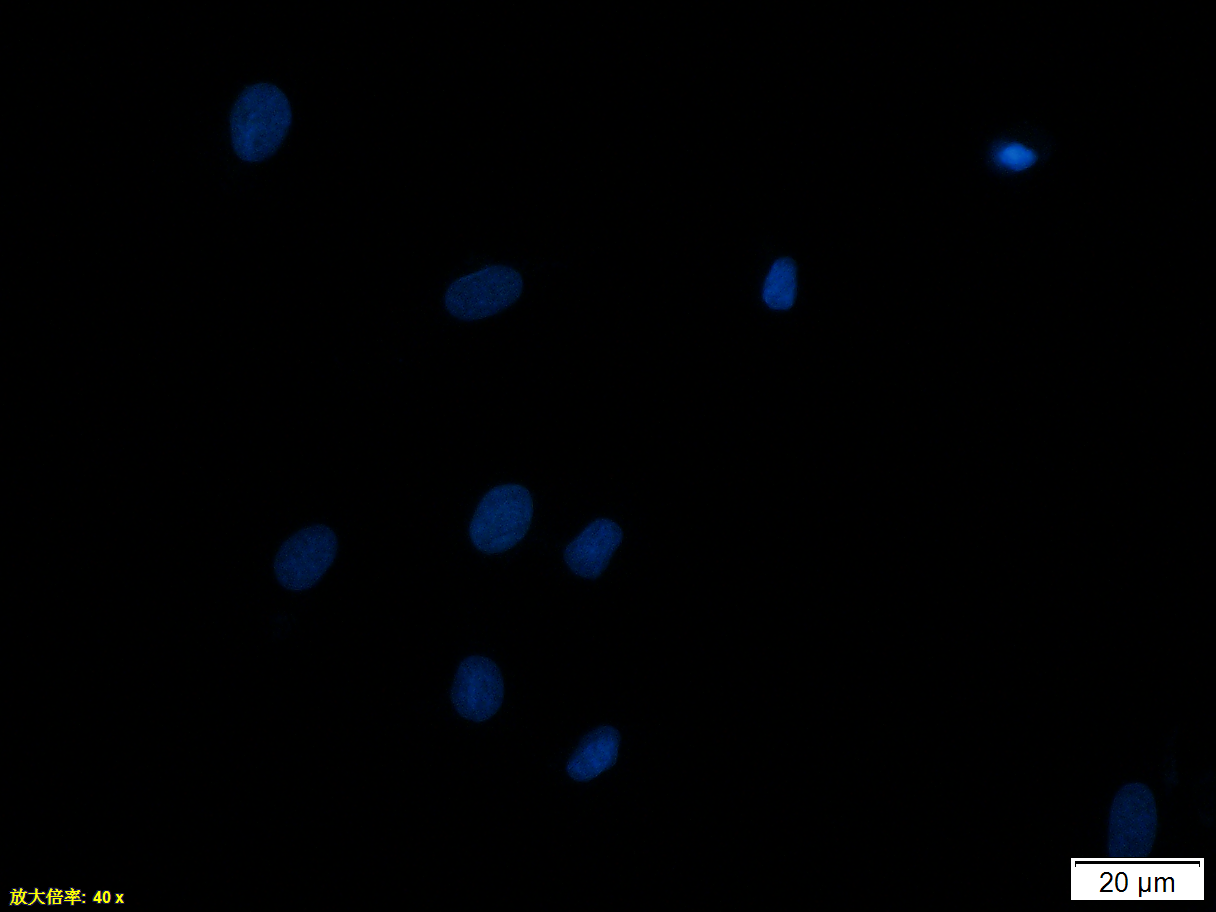

Supplement: S6 File — (ZIP) [file pone.0191616.s006.zip › Original data underlying the findings described in manuscript-TUNEL staining for detecting the apoptosis of CSCs-1/H-Exo group/fig_7-1.tif]

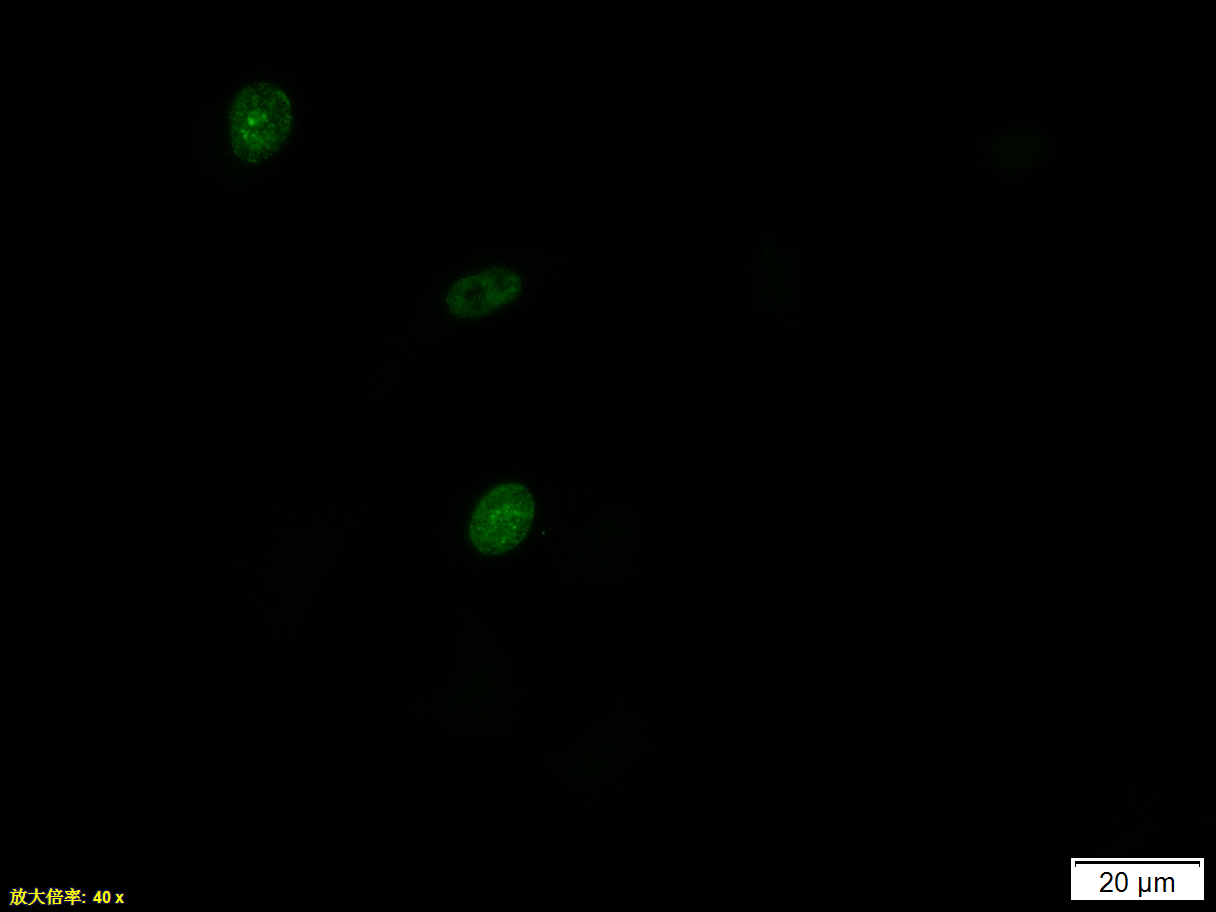

Supplement: S6 File — (ZIP) [file pone.0191616.s006.zip › Original data underlying the findings described in manuscript-TUNEL staining for detecting the apoptosis of CSCs-1/H-Exo group/fig_7-2.tif]

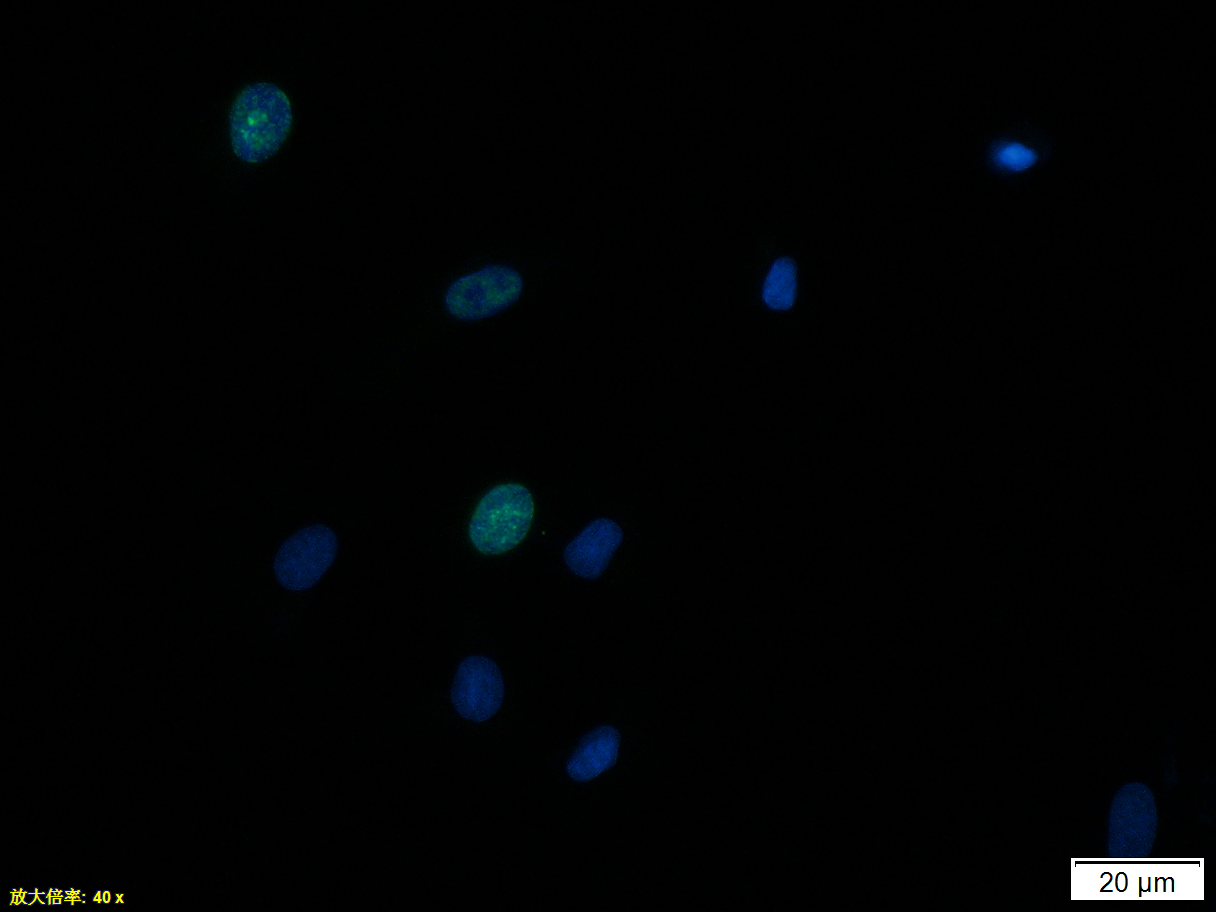

Supplement: S6 File — (ZIP) [file pone.0191616.s006.zip › Original data underlying the findings described in manuscript-TUNEL staining for detecting the apoptosis of CSCs-1/H-Exo group/fig_7.tif]

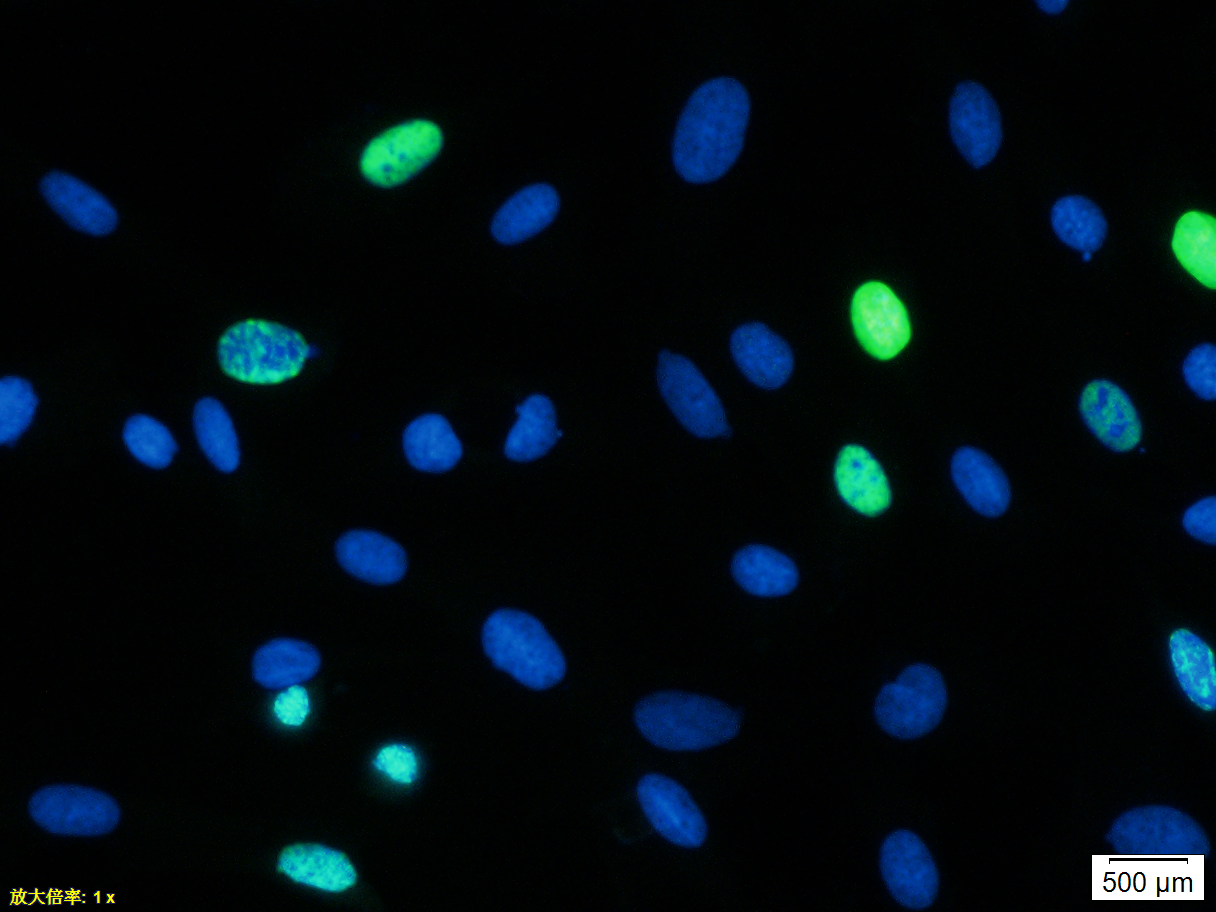

Supplement: S6 File — (ZIP) [file pone.0191616.s006.zip › Original data underlying the findings described in manuscript-TUNEL staining for detecting the apoptosis of CSCs-1/H-Exo+ly294002 group/fig_02.tif]

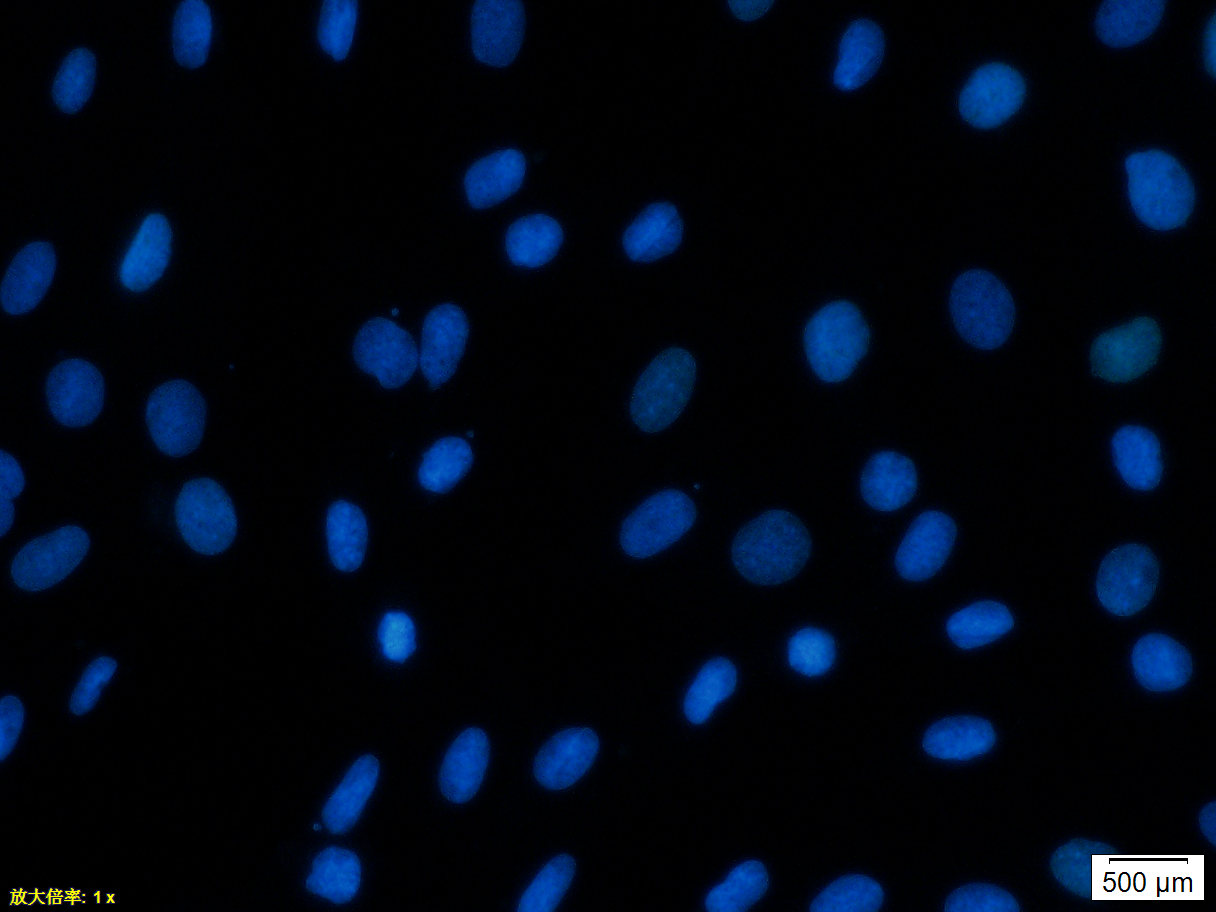

Supplement: S6 File — (ZIP) [file pone.0191616.s006.zip › Original data underlying the findings described in manuscript-TUNEL staining for detecting the apoptosis of CSCs-1/H-Exo+ly294002 group/fig_1-1.tif]

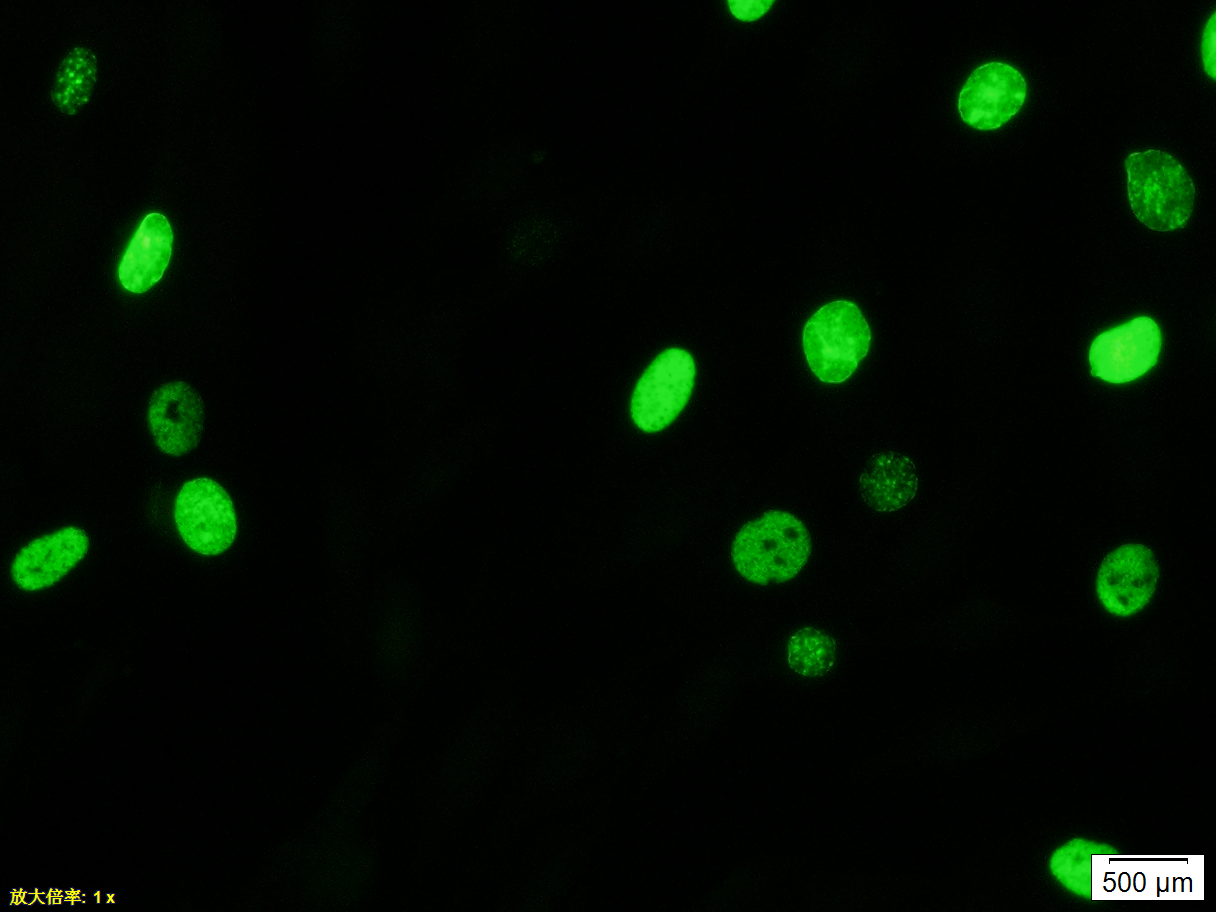

Supplement: S6 File — (ZIP) [file pone.0191616.s006.zip › Original data underlying the findings described in manuscript-TUNEL staining for detecting the apoptosis of CSCs-1/H-Exo+ly294002 group/fig_1-2.tif]

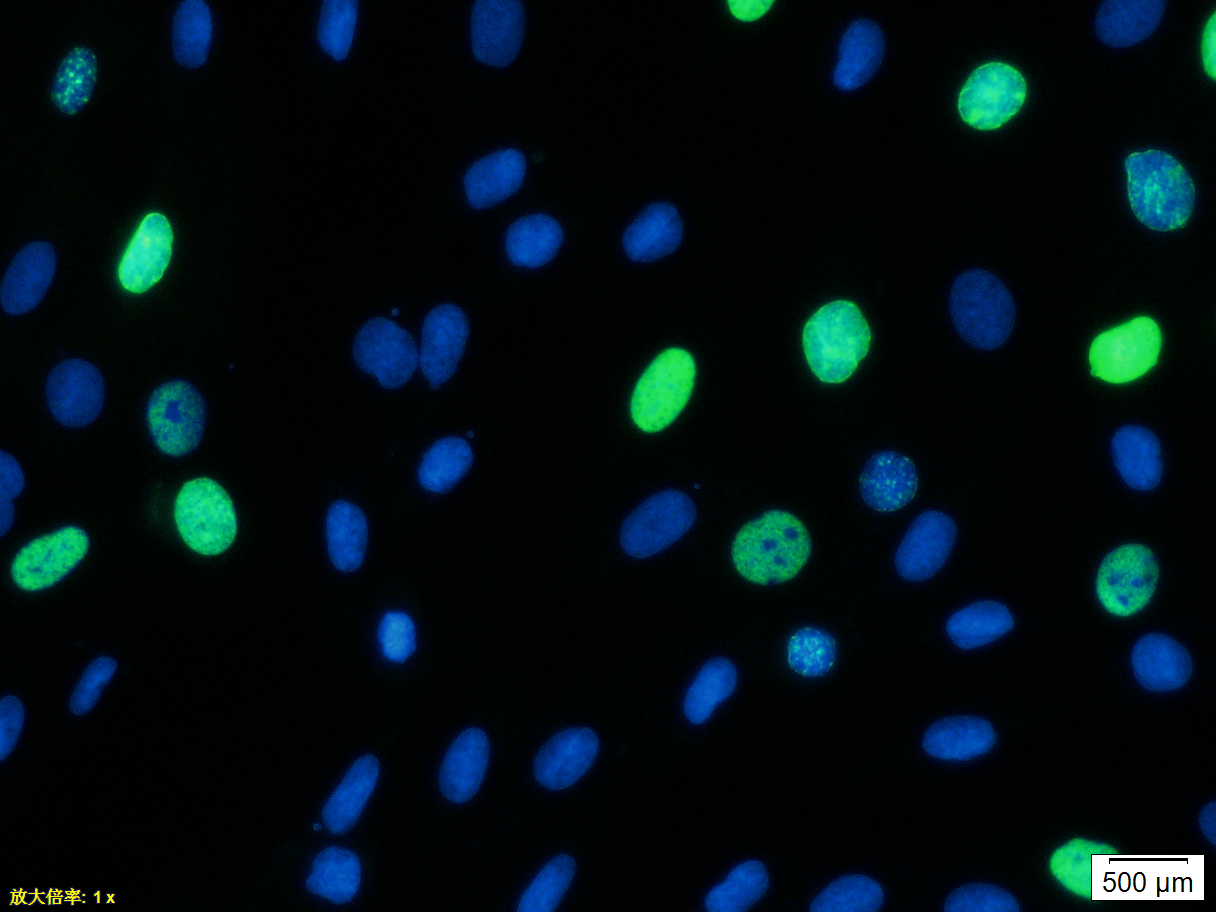

Supplement: S6 File — (ZIP) [file pone.0191616.s006.zip › Original data underlying the findings described in manuscript-TUNEL staining for detecting the apoptosis of CSCs-1/H-Exo+ly294002 group/fig_1.tif]

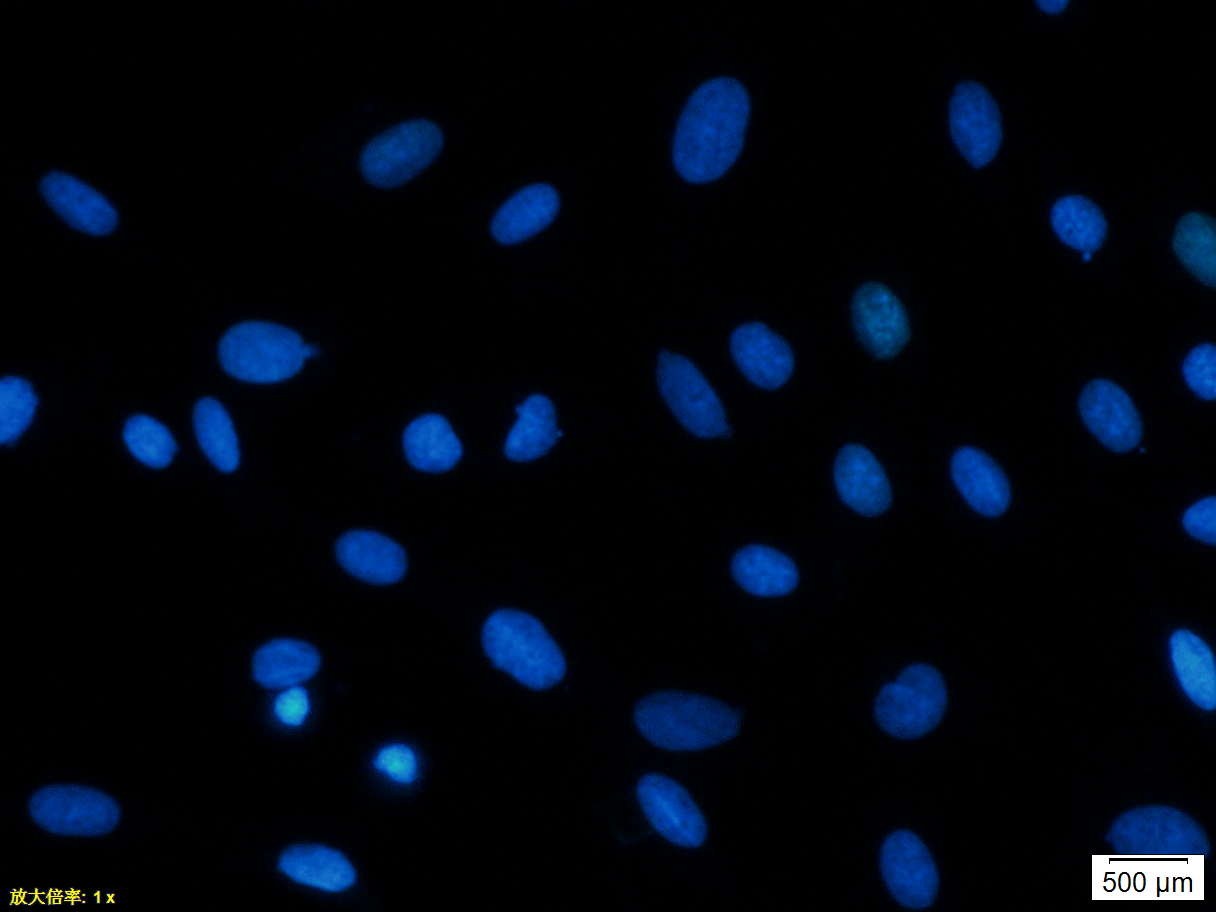

Supplement: S6 File — (ZIP) [file pone.0191616.s006.zip › Original data underlying the findings described in manuscript-TUNEL staining for detecting the apoptosis of CSCs-1/H-Exo+ly294002 group/fig_2-1.tif]

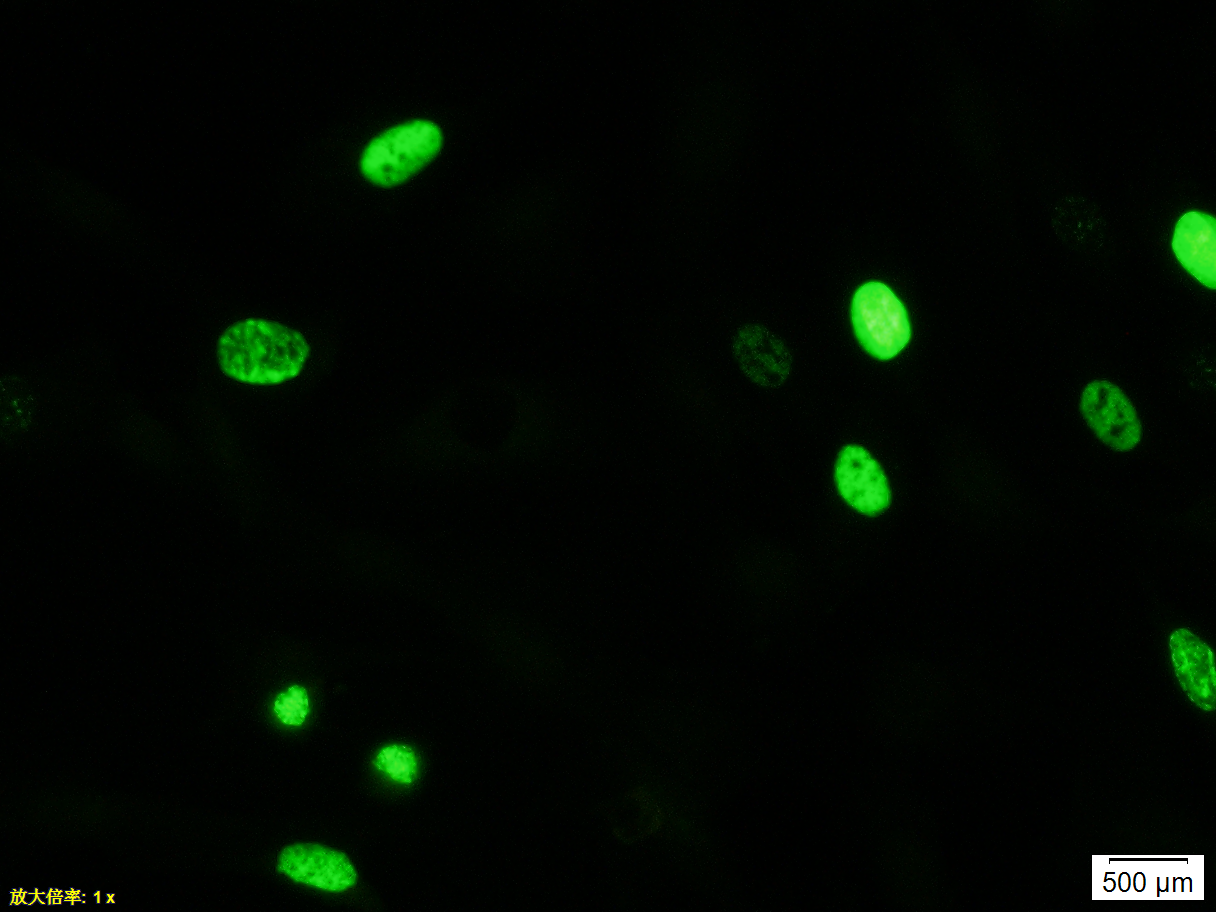

Supplement: S6 File — (ZIP) [file pone.0191616.s006.zip › Original data underlying the findings described in manuscript-TUNEL staining for detecting the apoptosis of CSCs-1/H-Exo+ly294002 group/fig_2-2.tif]

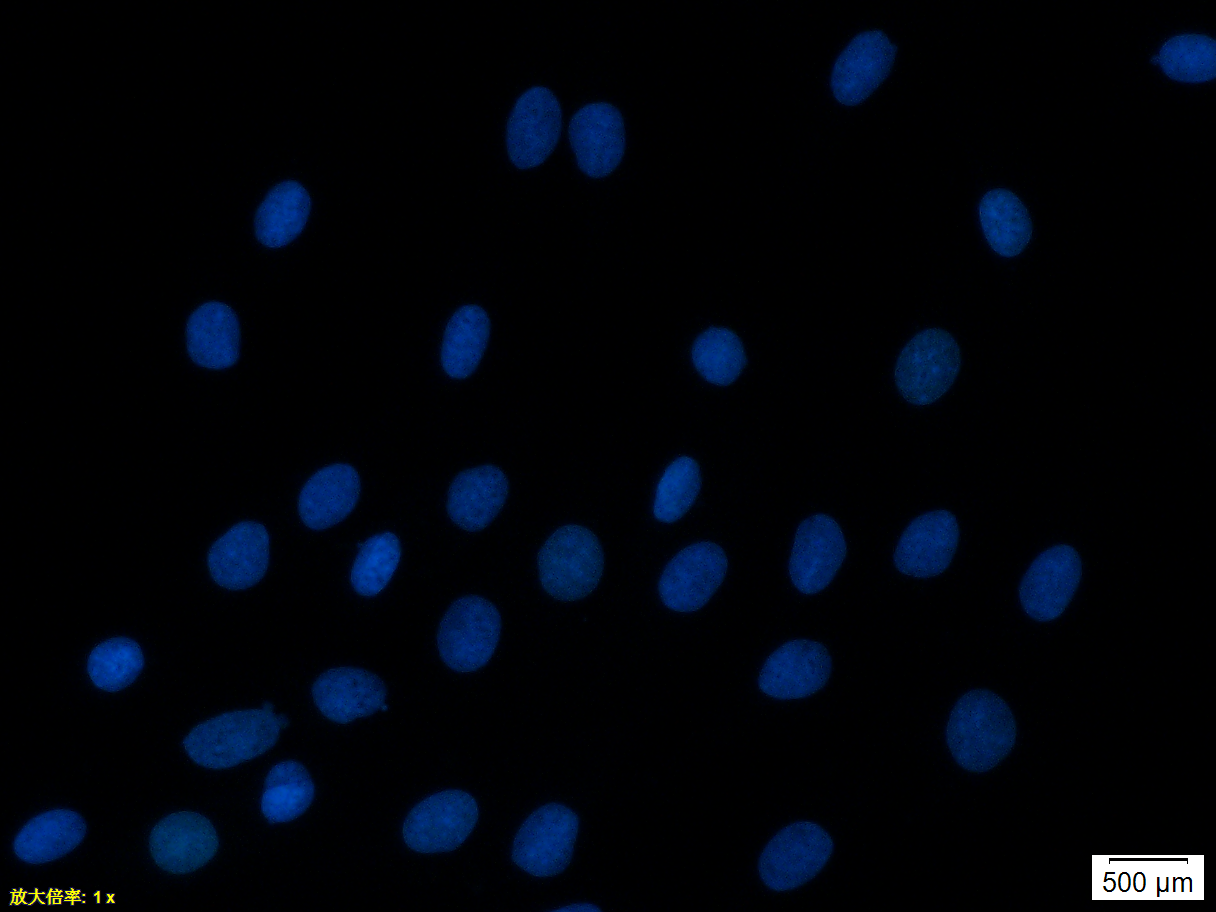

Supplement: S6 File — (ZIP) [file pone.0191616.s006.zip › Original data underlying the findings described in manuscript-TUNEL staining for detecting the apoptosis of CSCs-1/H-Exo+ly294002 group/fig_3-1.tif]

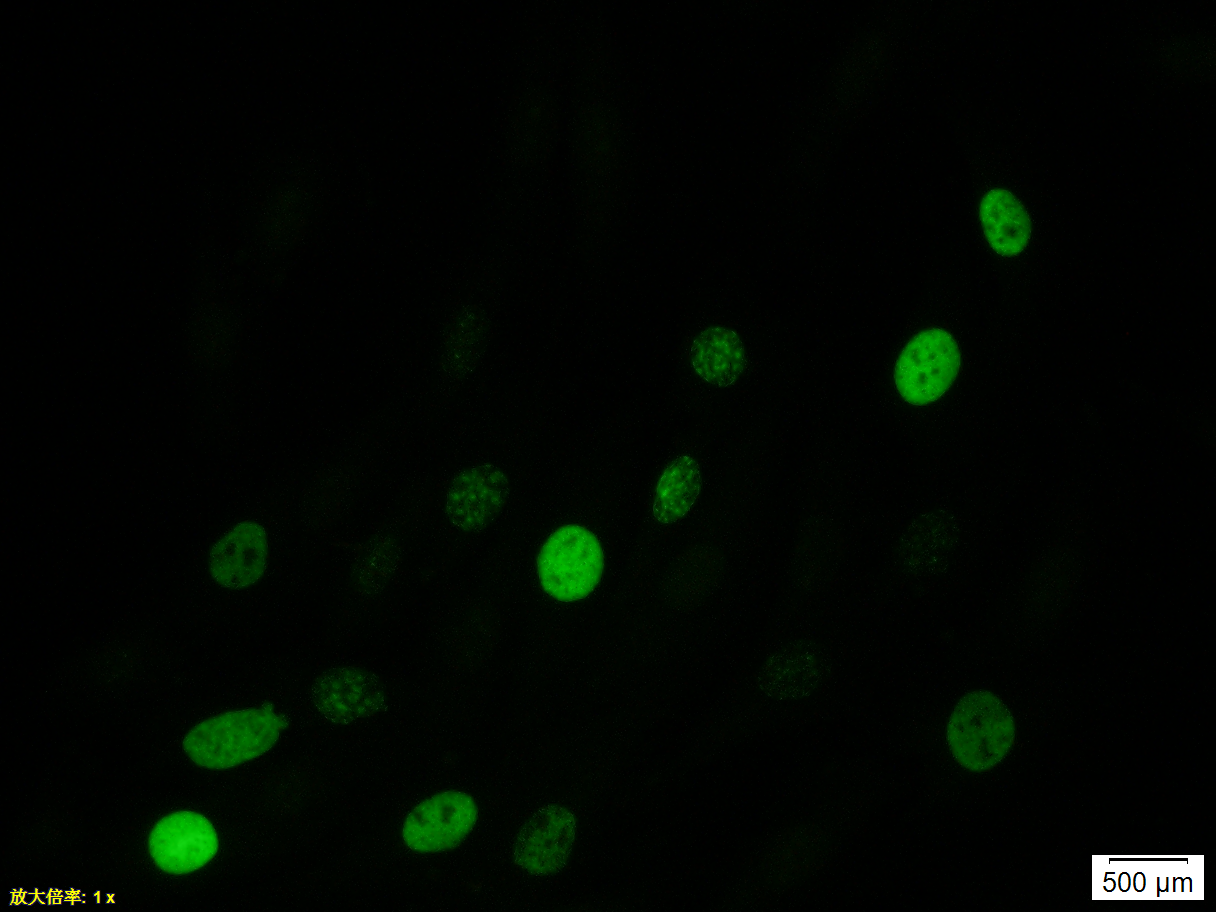

Supplement: S6 File — (ZIP) [file pone.0191616.s006.zip › Original data underlying the findings described in manuscript-TUNEL staining for detecting the apoptosis of CSCs-1/H-Exo+ly294002 group/fig_3-2.tif]

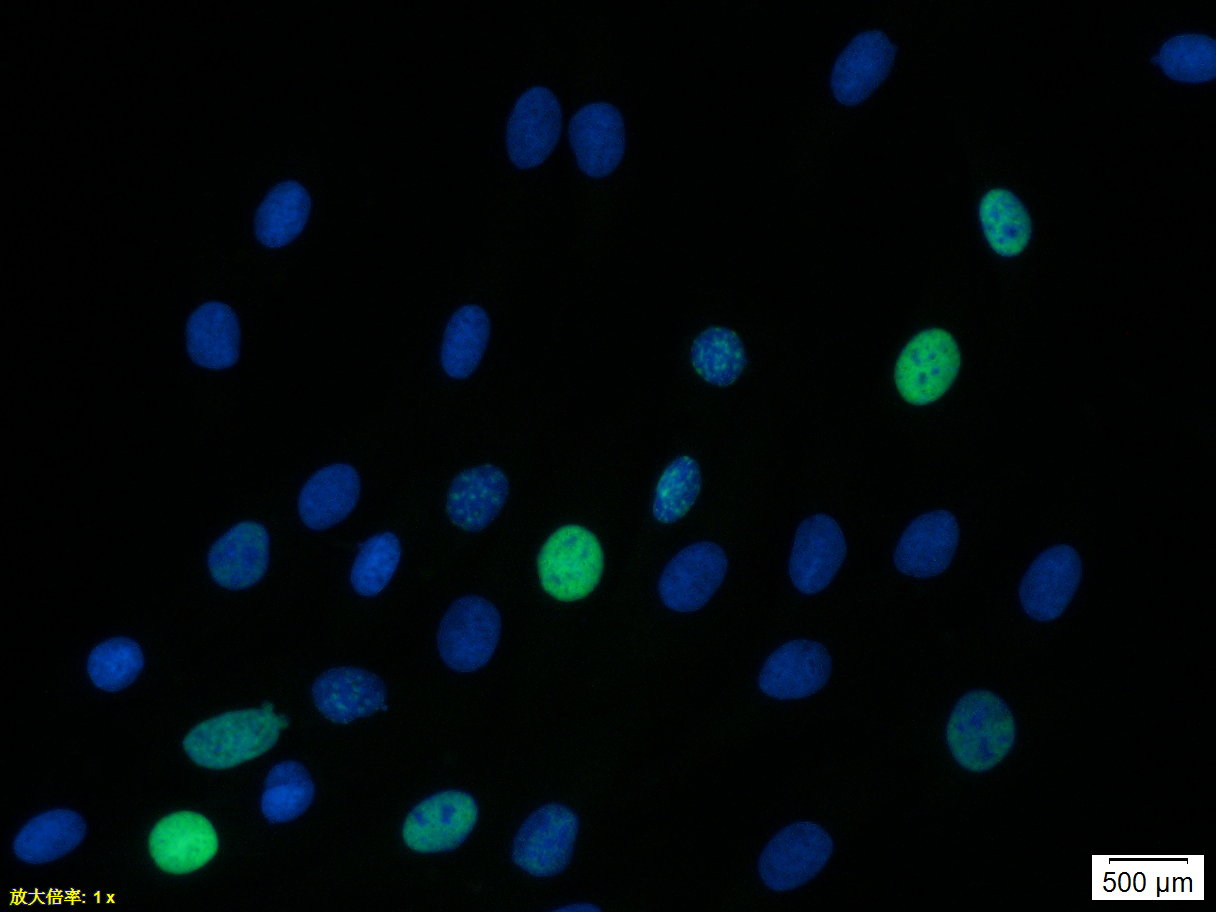

Supplement: S6 File — (ZIP) [file pone.0191616.s006.zip › Original data underlying the findings described in manuscript-TUNEL staining for detecting the apoptosis of CSCs-1/H-Exo+ly294002 group/fig_3.tif]

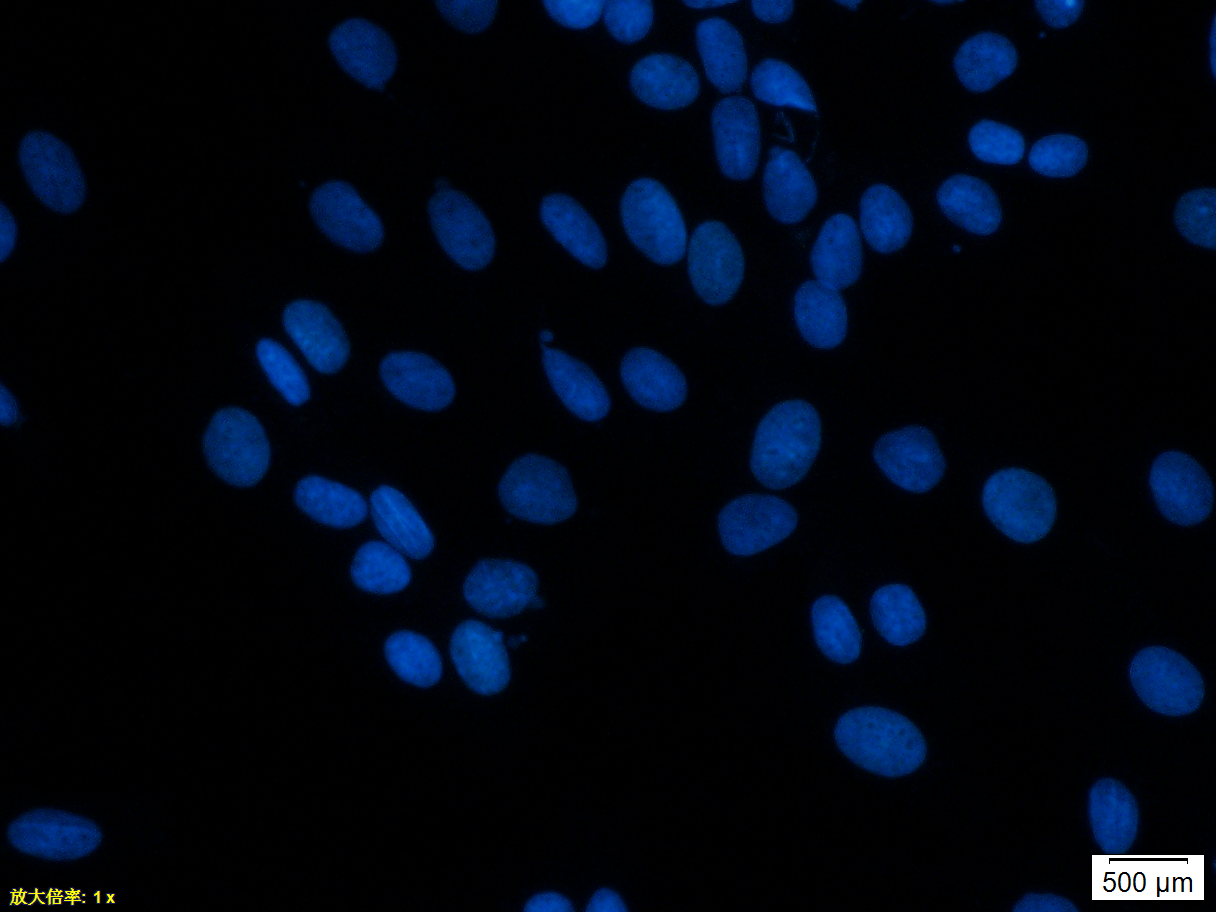

Supplement: S6 File — (ZIP) [file pone.0191616.s006.zip › Original data underlying the findings described in manuscript-TUNEL staining for detecting the apoptosis of CSCs-1/H-Exo+ly294002 group/fig_4-1.tif]

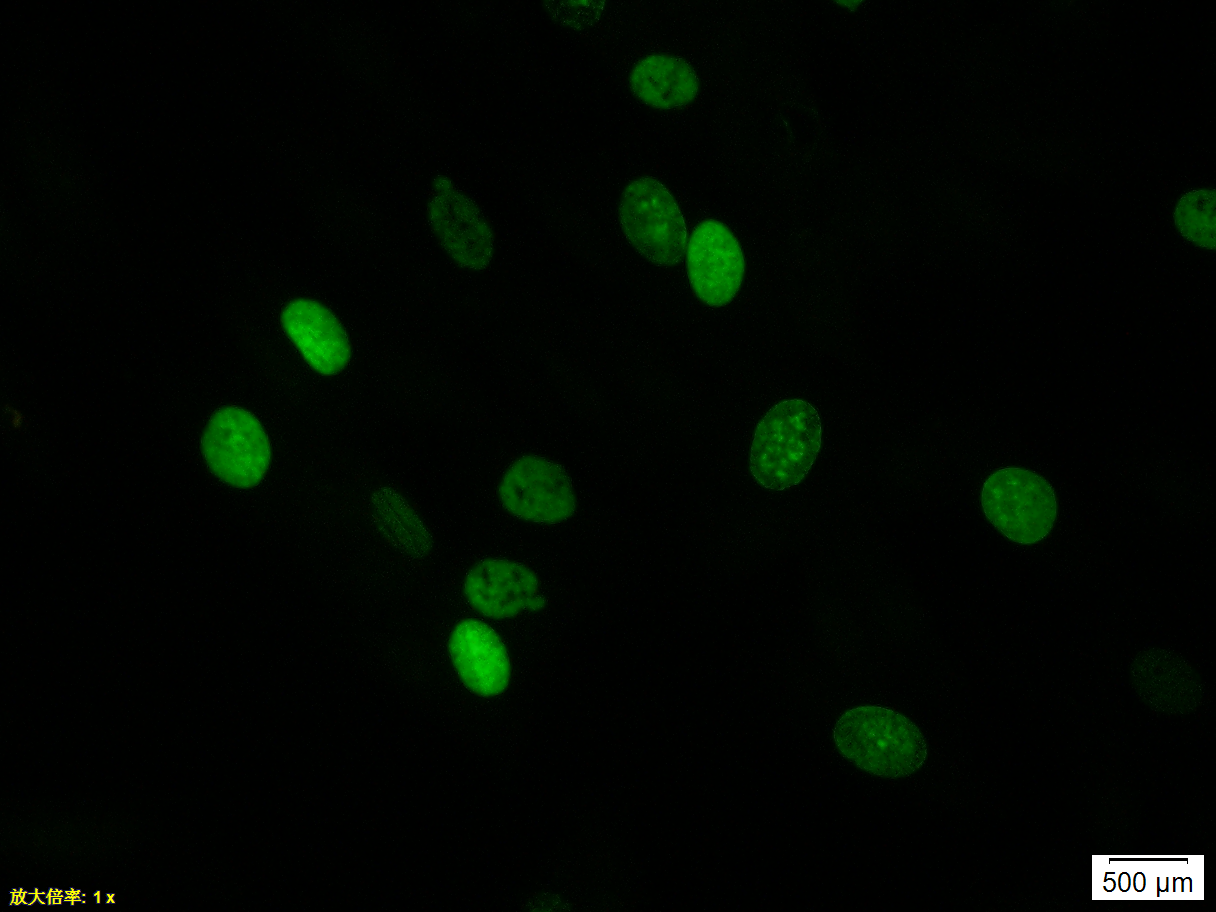

Supplement: S6 File — (ZIP) [file pone.0191616.s006.zip › Original data underlying the findings described in manuscript-TUNEL staining for detecting the apoptosis of CSCs-1/H-Exo+ly294002 group/fig_4-2.tif]

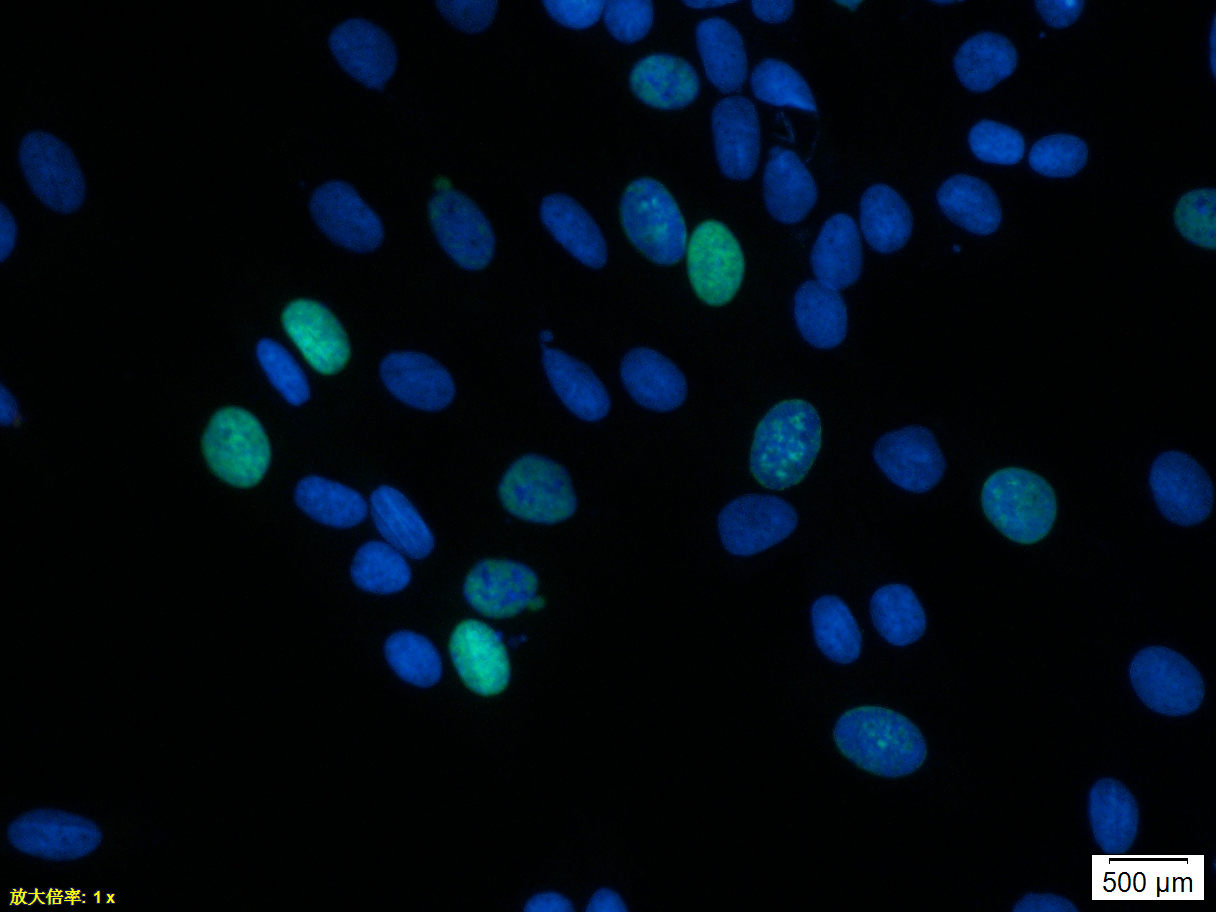

Supplement: S6 File — (ZIP) [file pone.0191616.s006.zip › Original data underlying the findings described in manuscript-TUNEL staining for detecting the apoptosis of CSCs-1/H-Exo+ly294002 group/fig_4.tif]

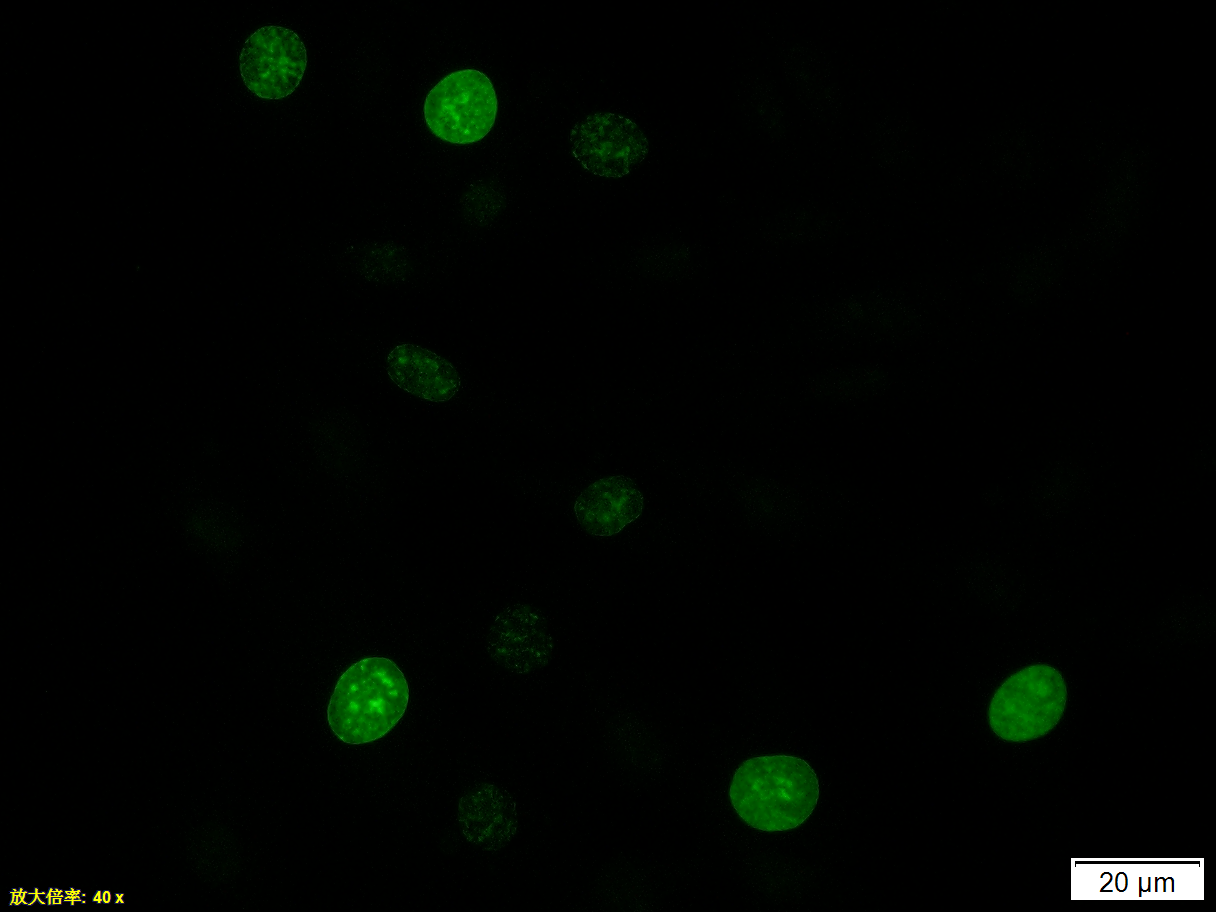

Supplement: S6 File — (ZIP) [file pone.0191616.s006.zip › Original data underlying the findings described in manuscript-TUNEL staining for detecting the apoptosis of CSCs-1/H-Exo+ly294002 group/fig_5-1.tif]

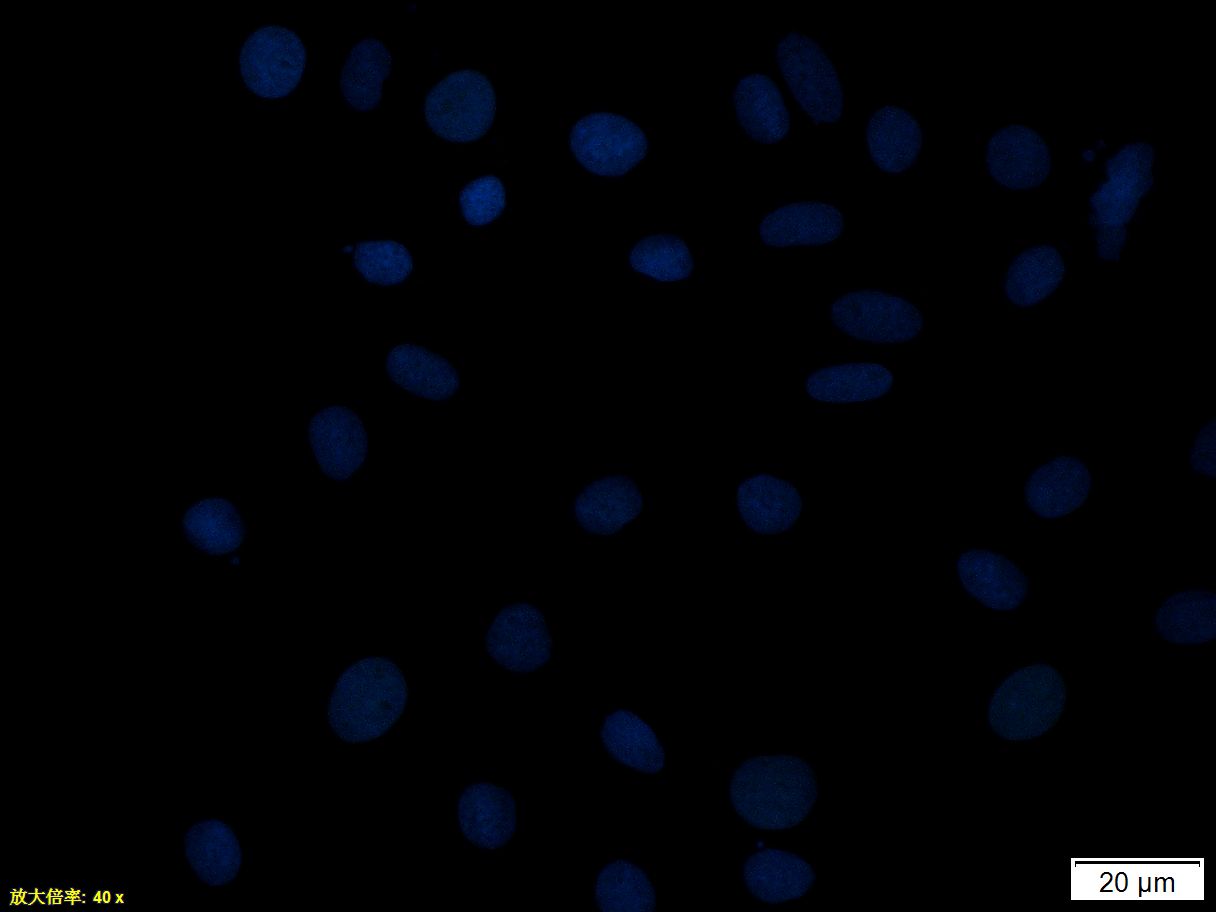

Supplement: S6 File — (ZIP) [file pone.0191616.s006.zip › Original data underlying the findings described in manuscript-TUNEL staining for detecting the apoptosis of CSCs-1/H-Exo+ly294002 group/fig_5-2.tif]

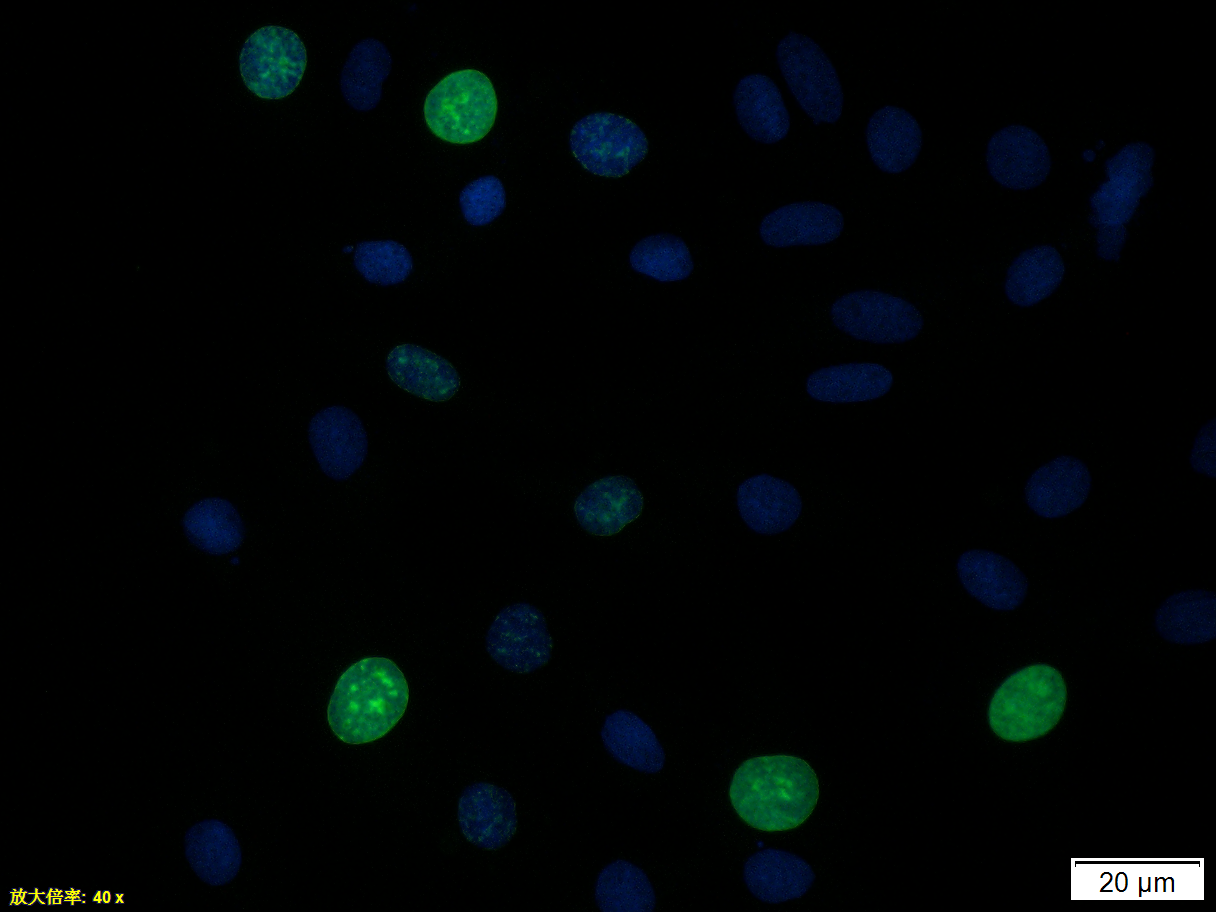

Supplement: S6 File — (ZIP) [file pone.0191616.s006.zip › Original data underlying the findings described in manuscript-TUNEL staining for detecting the apoptosis of CSCs-1/H-Exo+ly294002 group/fig_5.tif]

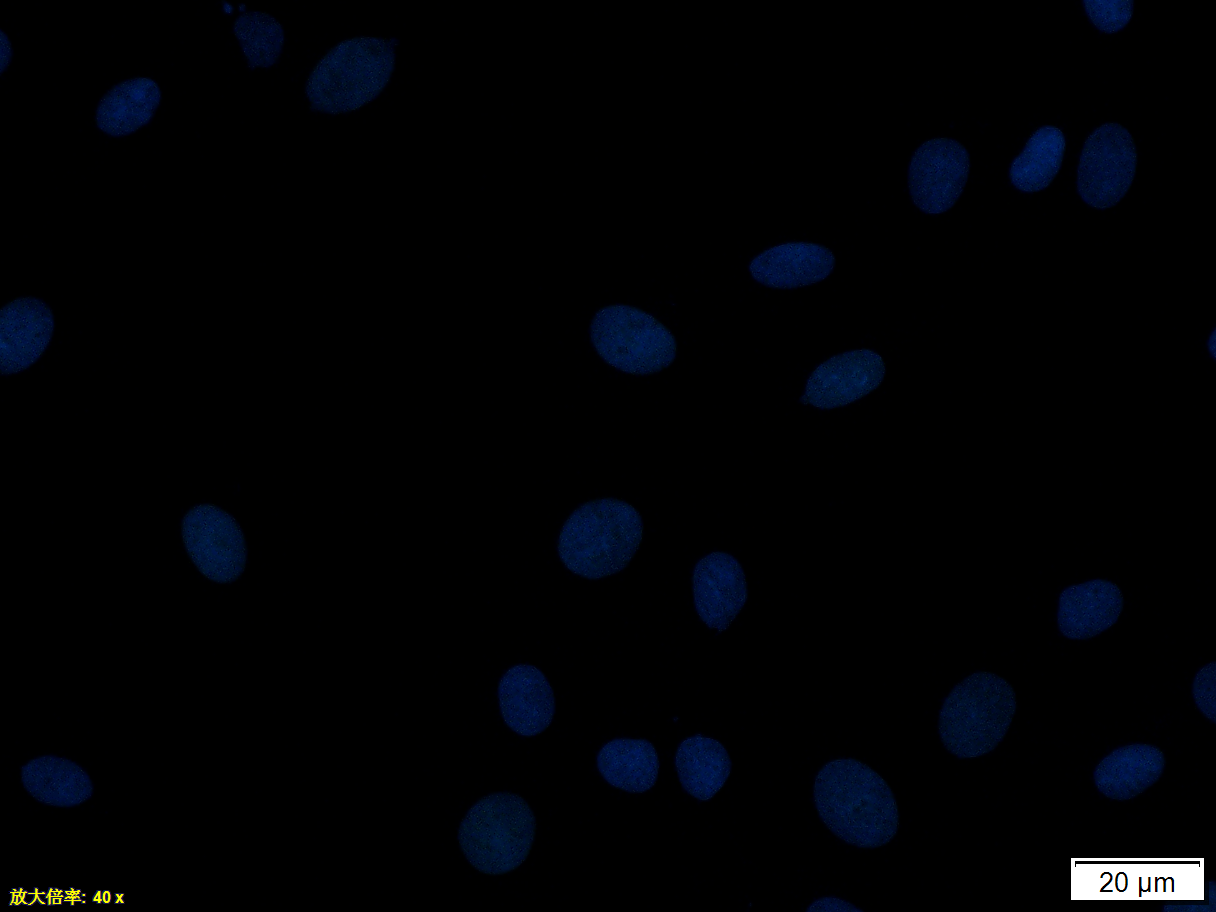

Supplement: S6 File — (ZIP) [file pone.0191616.s006.zip › Original data underlying the findings described in manuscript-TUNEL staining for detecting the apoptosis of CSCs-1/H-Exo+ly294002 group/fig_6-1.tif]

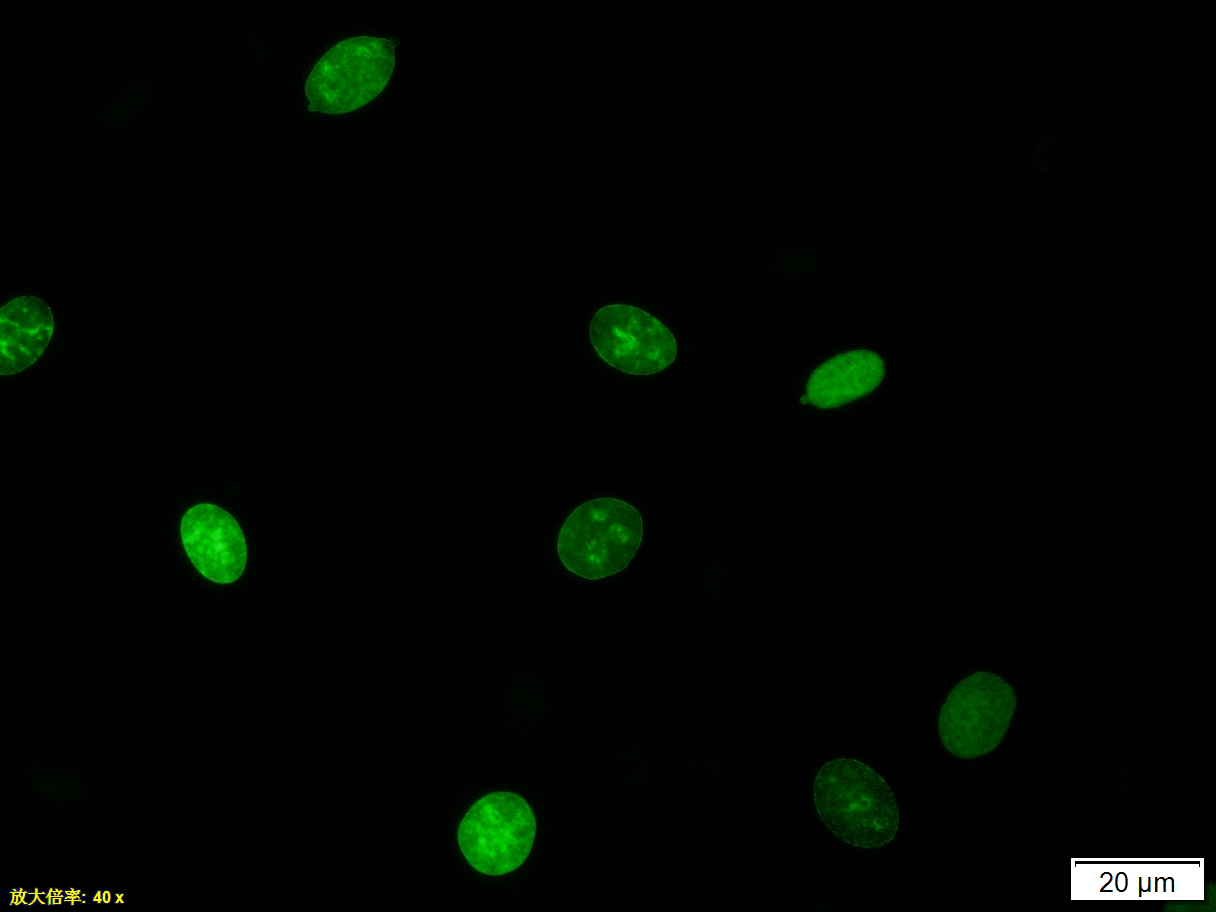

Supplement: S6 File — (ZIP) [file pone.0191616.s006.zip › Original data underlying the findings described in manuscript-TUNEL staining for detecting the apoptosis of CSCs-1/H-Exo+ly294002 group/fig_6-2.tif]

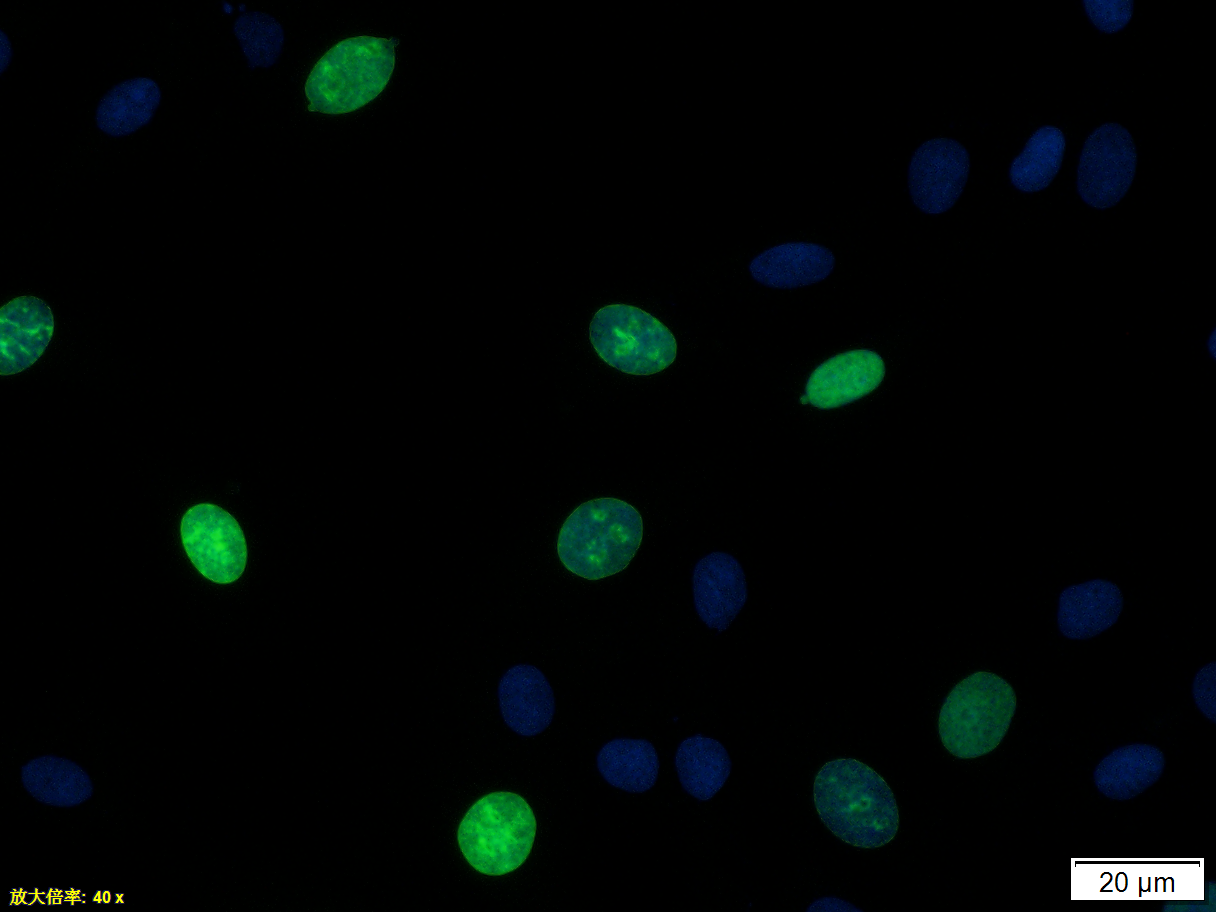

Supplement: S6 File — (ZIP) [file pone.0191616.s006.zip › Original data underlying the findings described in manuscript-TUNEL staining for detecting the apoptosis of CSCs-1/H-Exo+ly294002 group/fig_6.tif]

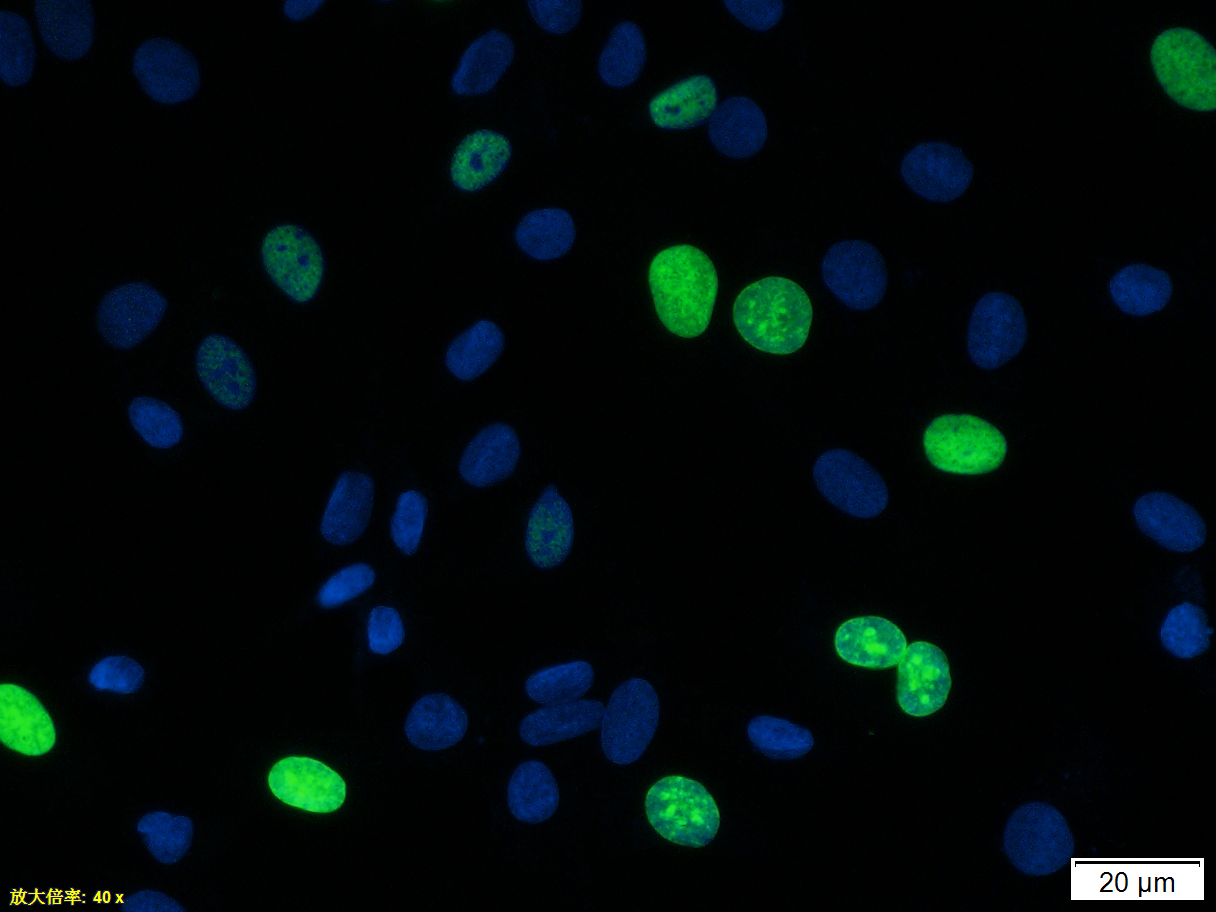

Supplement: S6 File — (ZIP) [file pone.0191616.s006.zip › Original data underlying the findings described in manuscript-TUNEL staining for detecting the apoptosis of CSCs-1/H2O2 group/fig_01.tif]

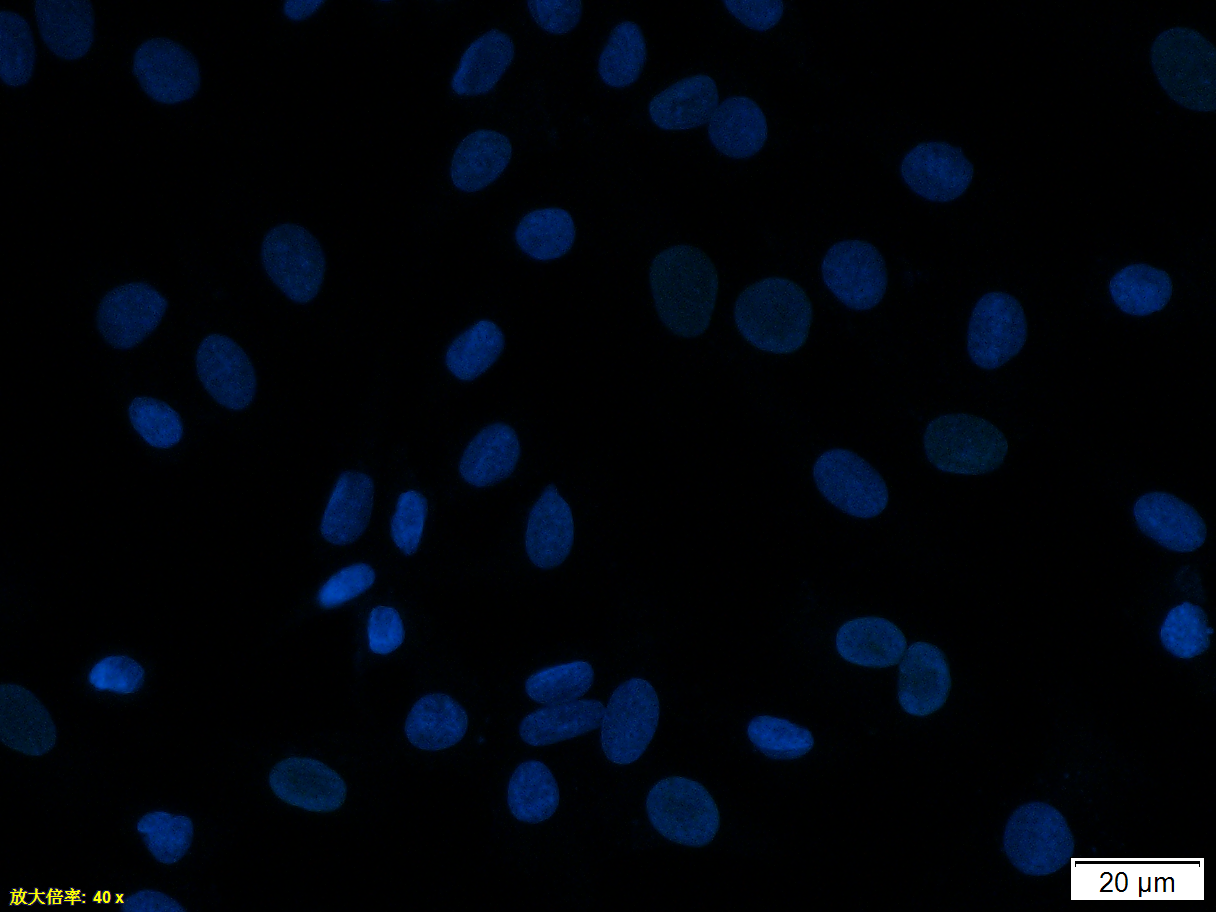

Supplement: S6 File — (ZIP) [file pone.0191616.s006.zip › Original data underlying the findings described in manuscript-TUNEL staining for detecting the apoptosis of CSCs-1/H2O2 group/fig_1-1.tif]

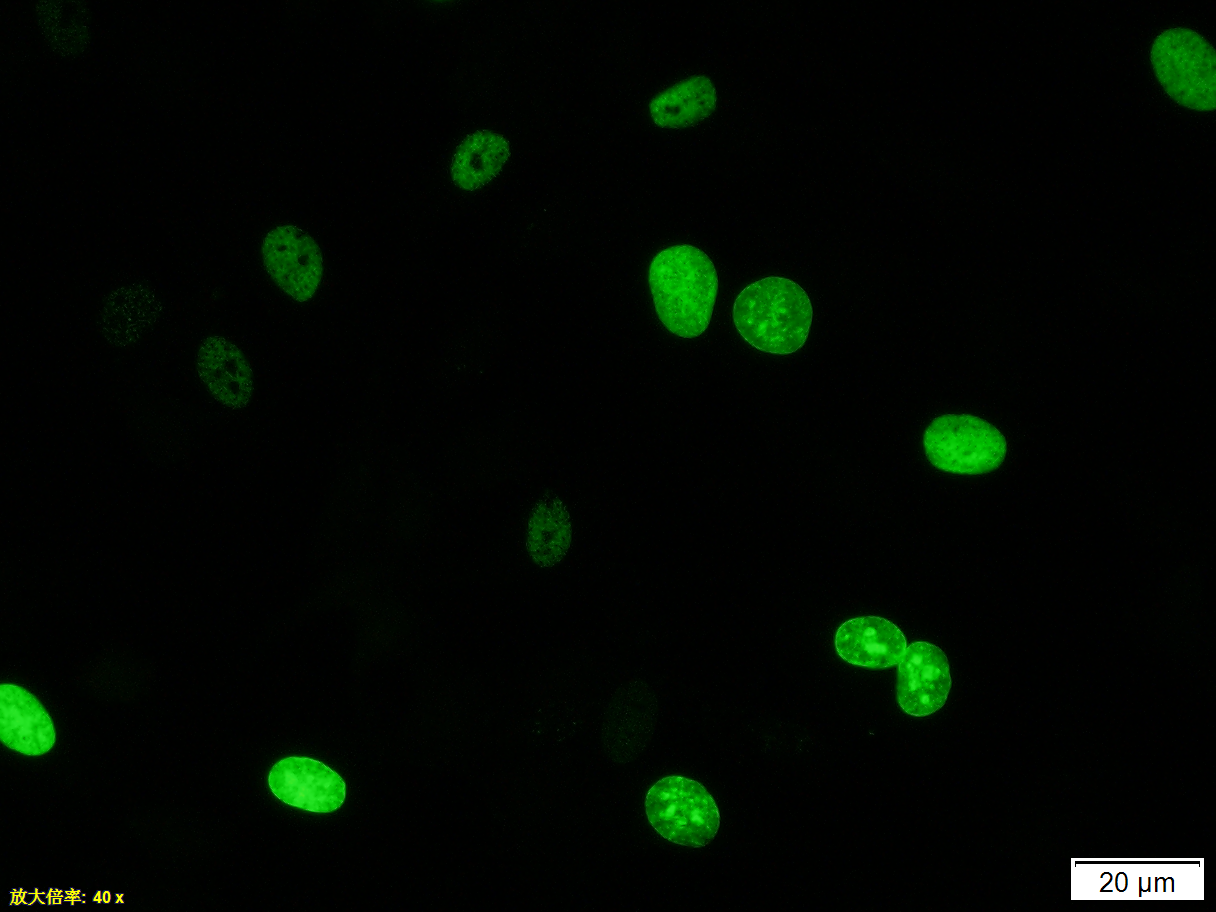

Supplement: S6 File — (ZIP) [file pone.0191616.s006.zip › Original data underlying the findings described in manuscript-TUNEL staining for detecting the apoptosis of CSCs-1/H2O2 group/fig_1-2.tif]

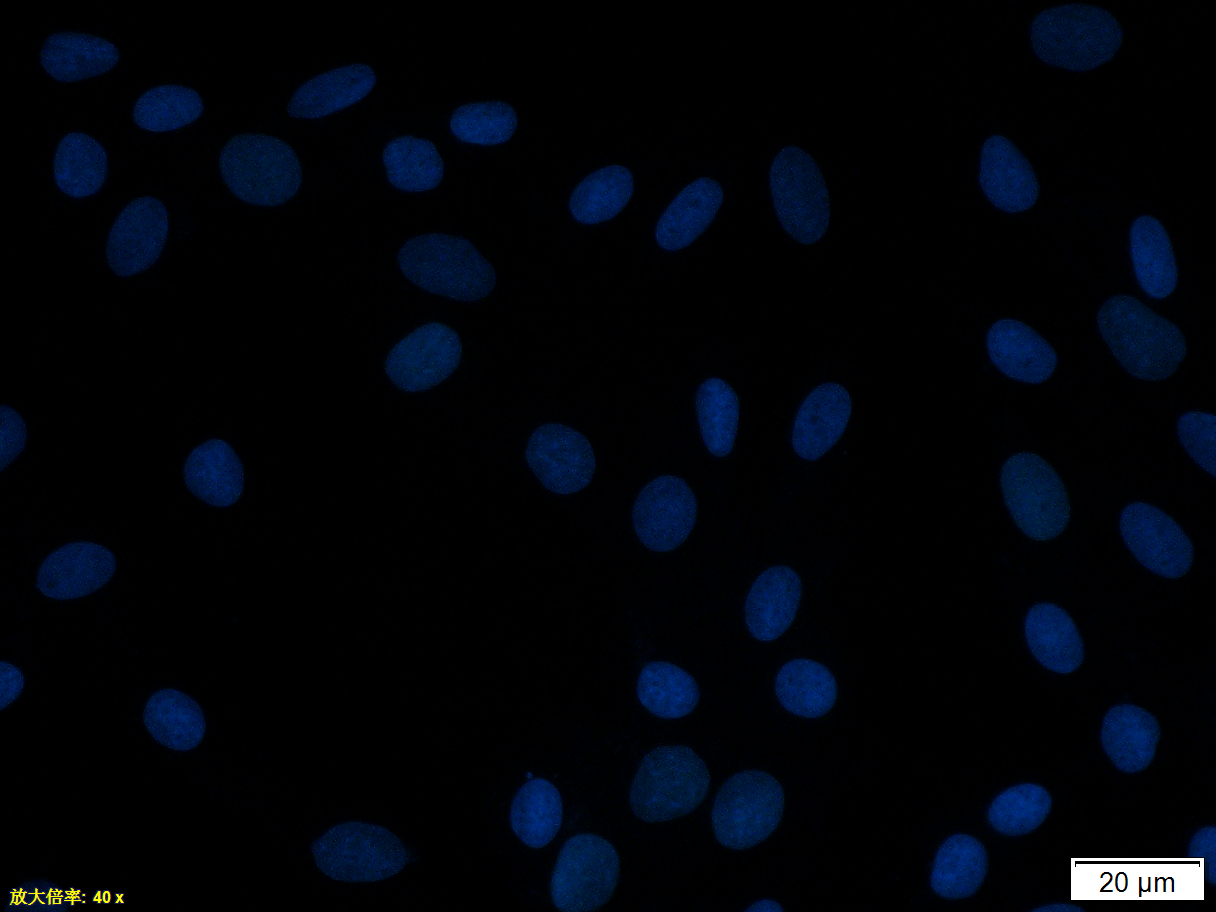

Supplement: S6 File — (ZIP) [file pone.0191616.s006.zip › Original data underlying the findings described in manuscript-TUNEL staining for detecting the apoptosis of CSCs-1/H2O2 group/fig_2-1.tif]

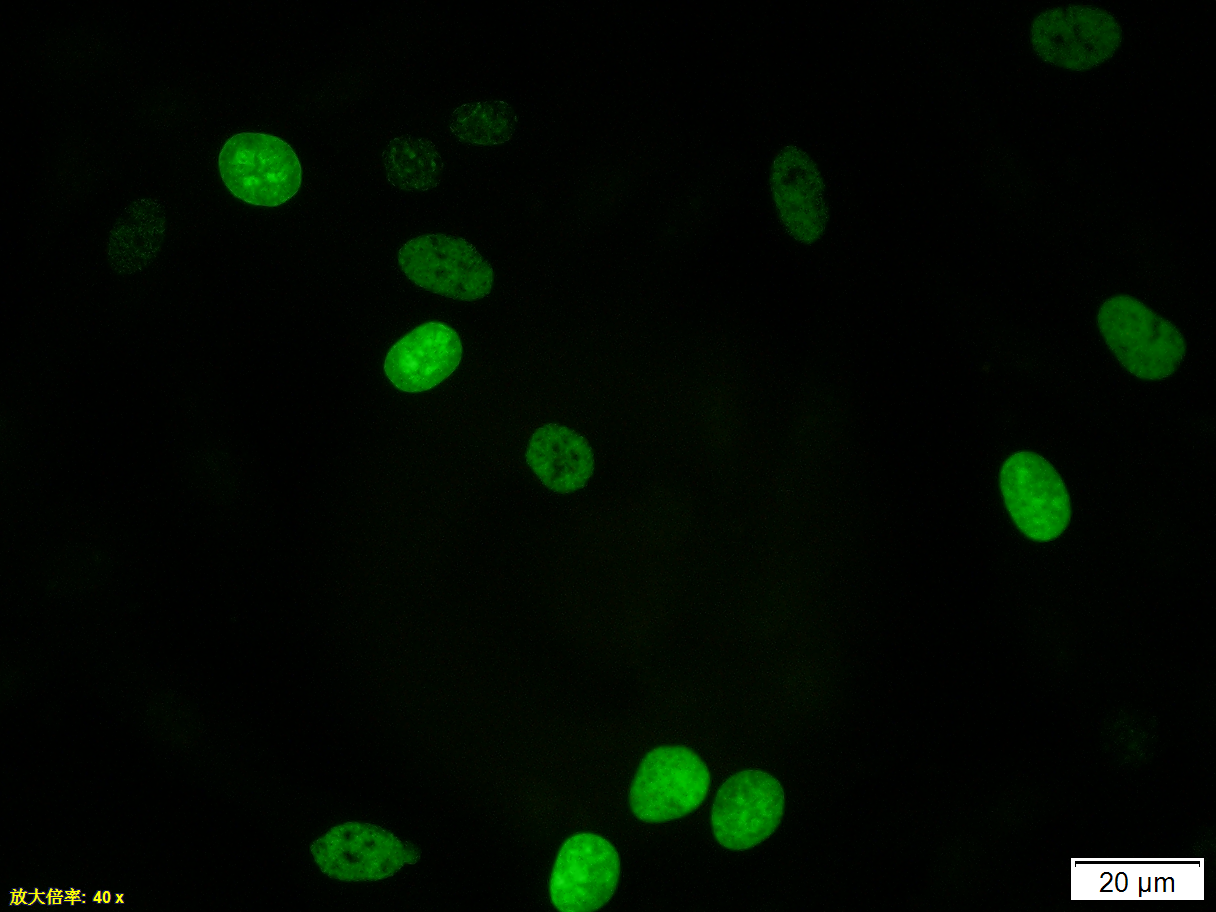

Supplement: S6 File — (ZIP) [file pone.0191616.s006.zip › Original data underlying the findings described in manuscript-TUNEL staining for detecting the apoptosis of CSCs-1/H2O2 group/fig_2-2.tif]

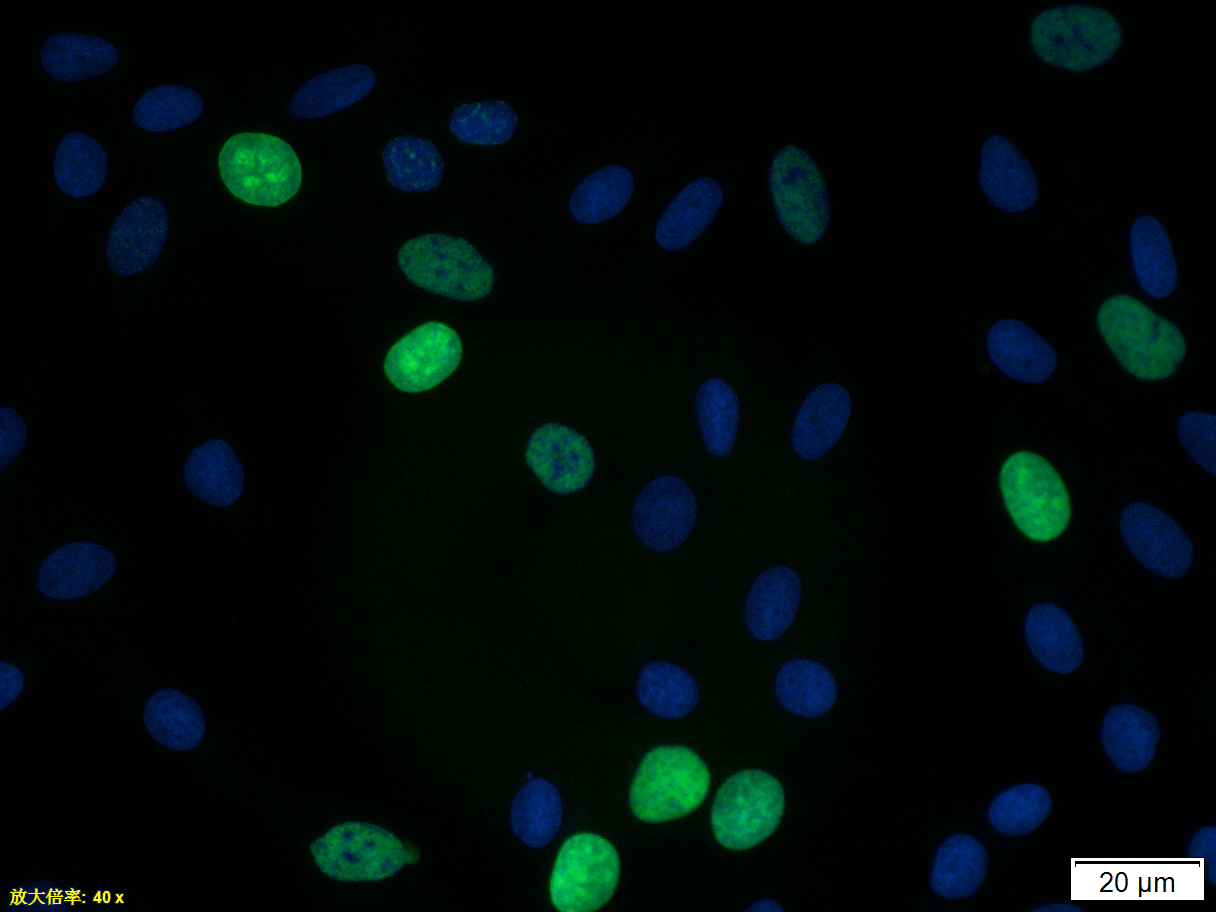

Supplement: S6 File — (ZIP) [file pone.0191616.s006.zip › Original data underlying the findings described in manuscript-TUNEL staining for detecting the apoptosis of CSCs-1/H2O2 group/fig_2.tif]

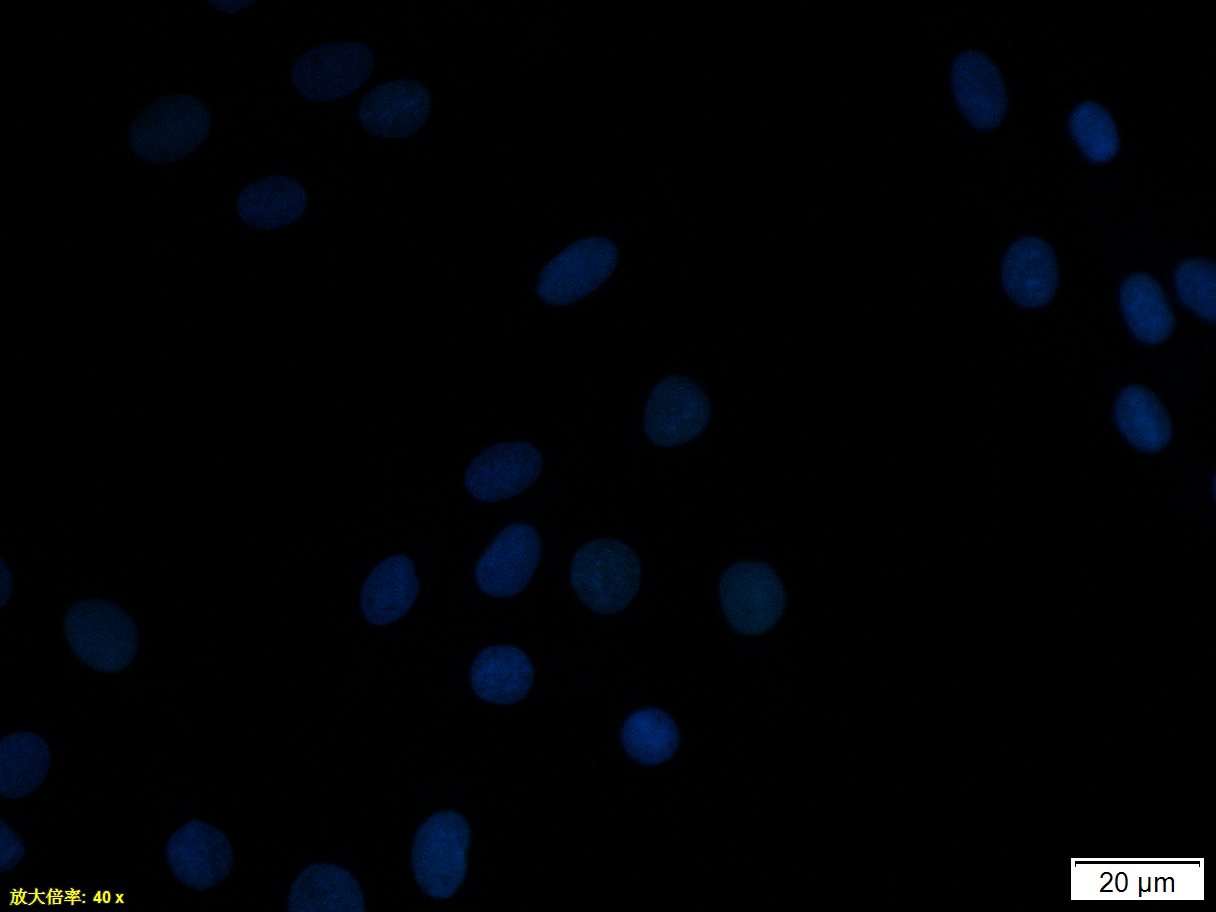

Supplement: S6 File — (ZIP) [file pone.0191616.s006.zip › Original data underlying the findings described in manuscript-TUNEL staining for detecting the apoptosis of CSCs-1/H2O2 group/fig_3-1.tif]

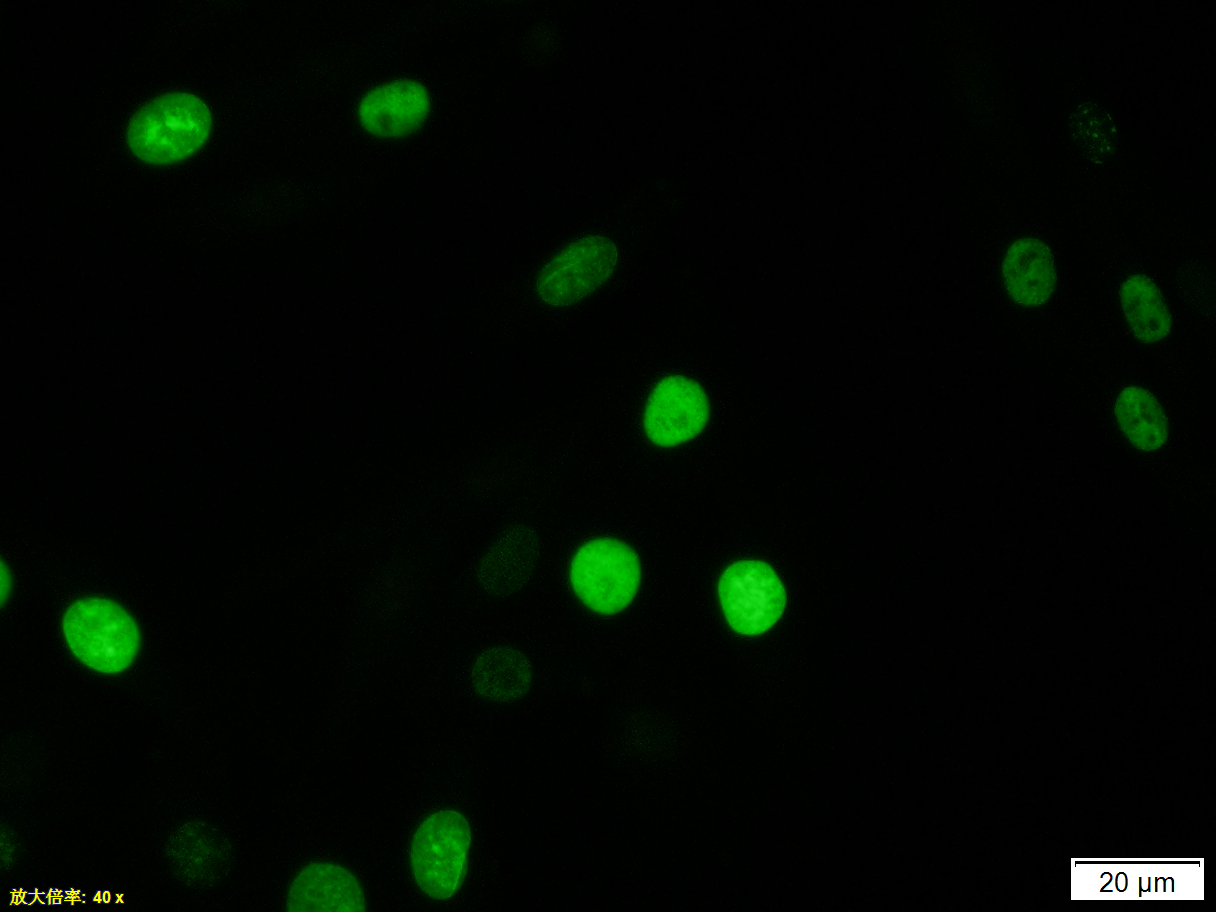

Supplement: S6 File — (ZIP) [file pone.0191616.s006.zip › Original data underlying the findings described in manuscript-TUNEL staining for detecting the apoptosis of CSCs-1/H2O2 group/fig_3-2.tif]

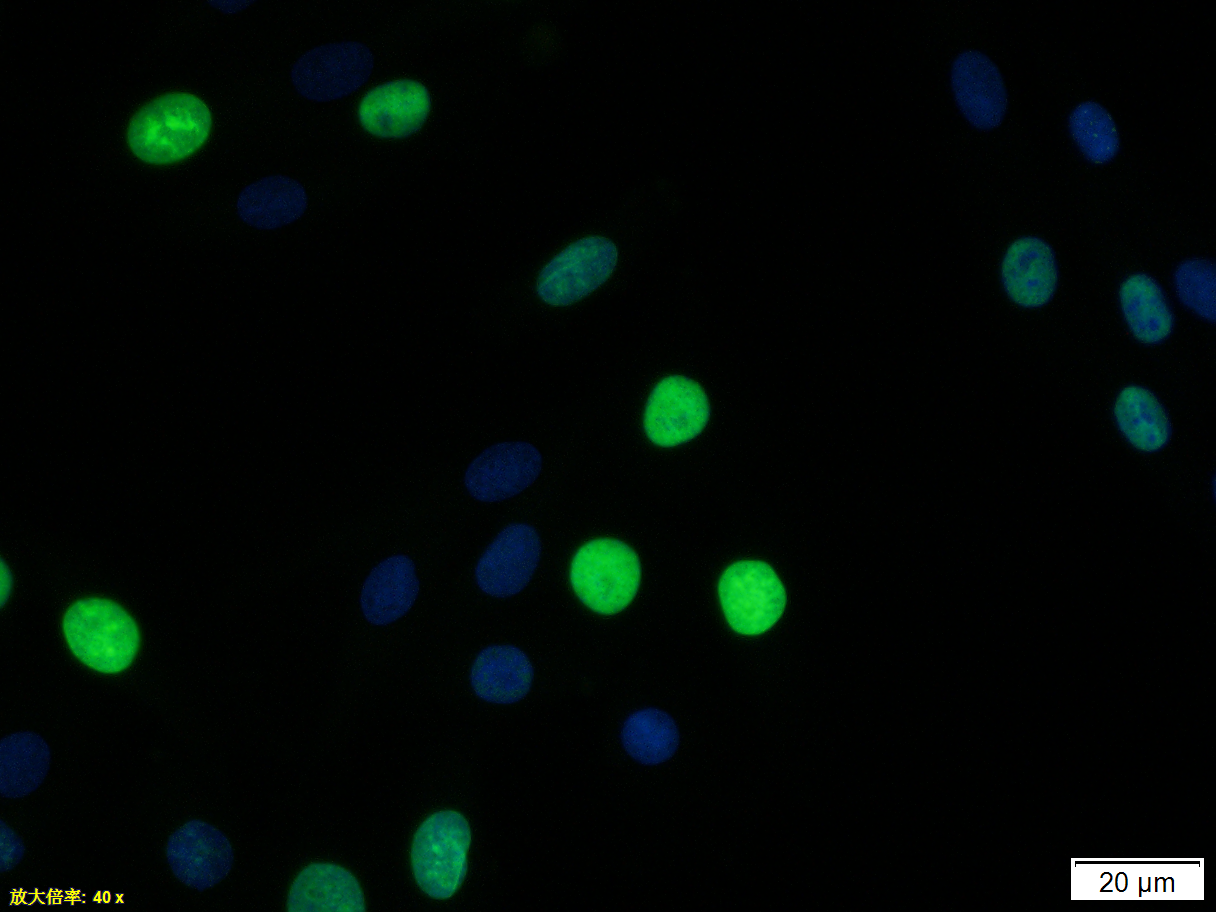

Supplement: S6 File — (ZIP) [file pone.0191616.s006.zip › Original data underlying the findings described in manuscript-TUNEL staining for detecting the apoptosis of CSCs-1/H2O2 group/fig_3.tif]

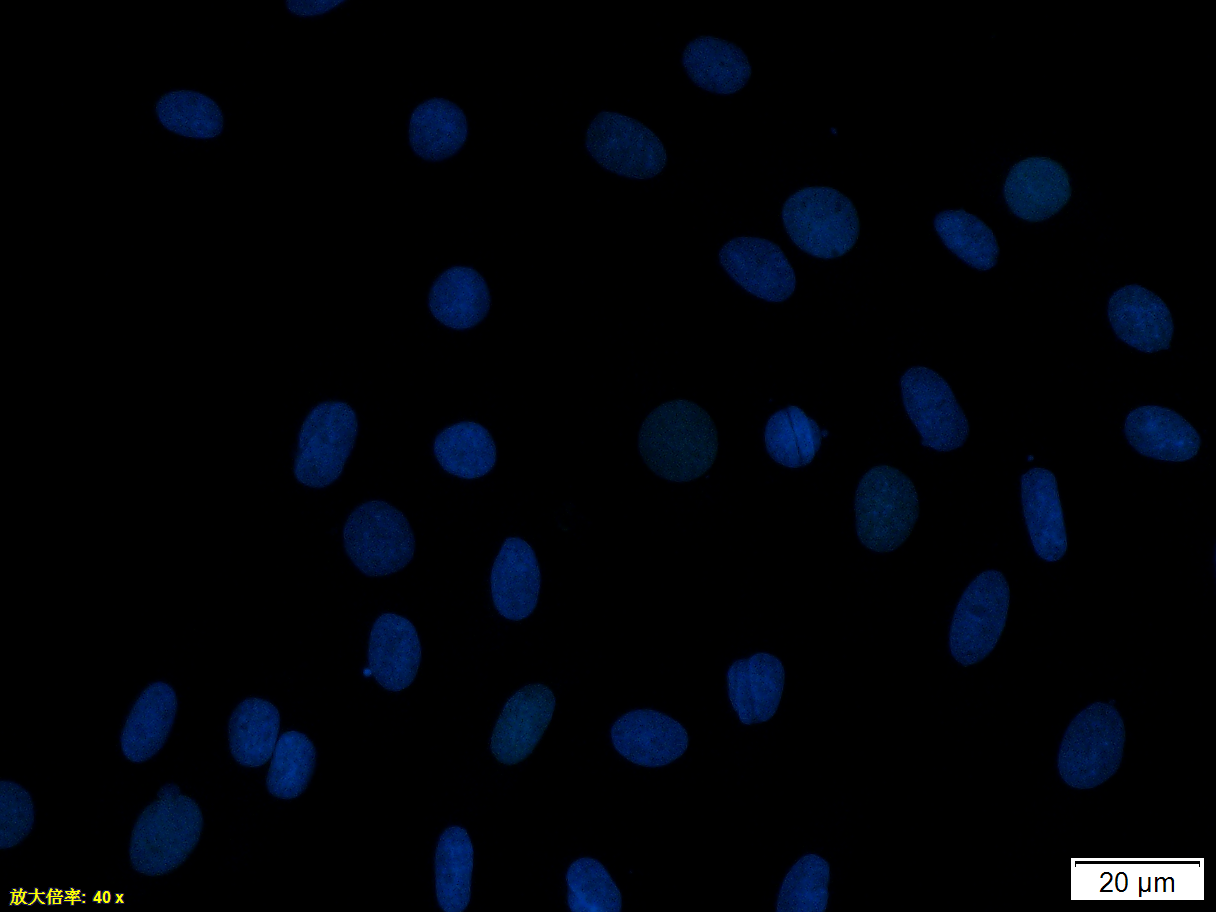

Supplement: S6 File — (ZIP) [file pone.0191616.s006.zip › Original data underlying the findings described in manuscript-TUNEL staining for detecting the apoptosis of CSCs-1/H2O2 group/fig_4-1.tif]

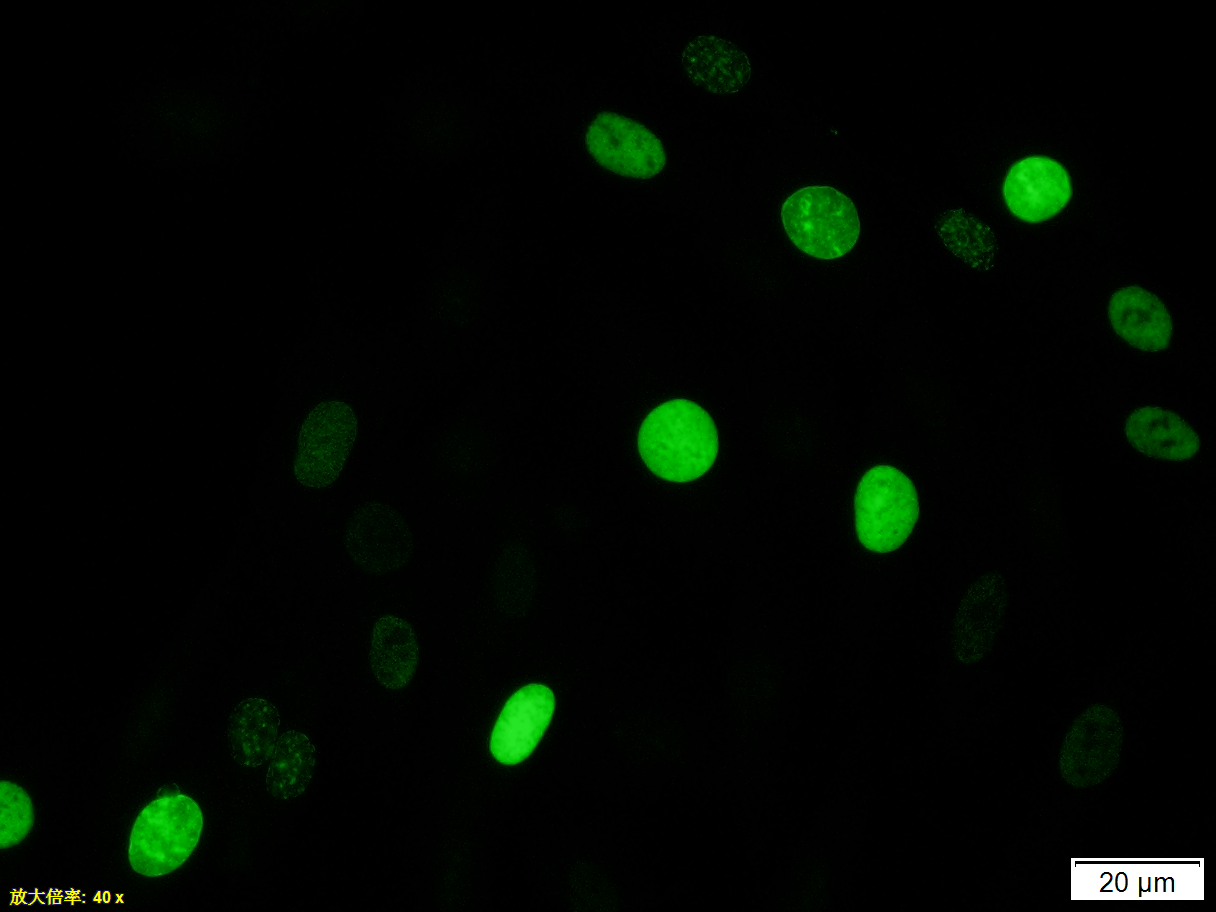

Supplement: S6 File — (ZIP) [file pone.0191616.s006.zip › Original data underlying the findings described in manuscript-TUNEL staining for detecting the apoptosis of CSCs-1/H2O2 group/fig_4-2.tif]

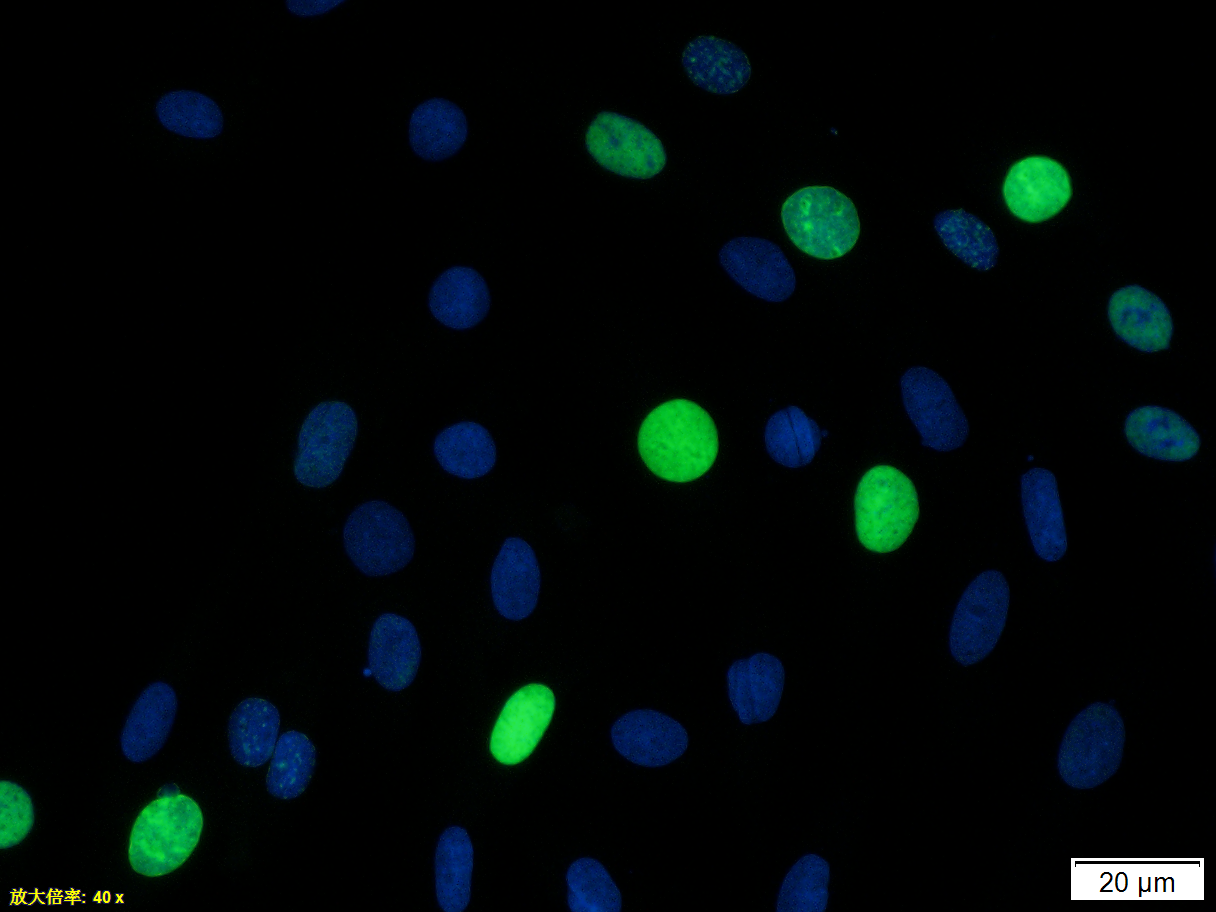

Supplement: S6 File — (ZIP) [file pone.0191616.s006.zip › Original data underlying the findings described in manuscript-TUNEL staining for detecting the apoptosis of CSCs-1/H2O2 group/fig_4.tif]

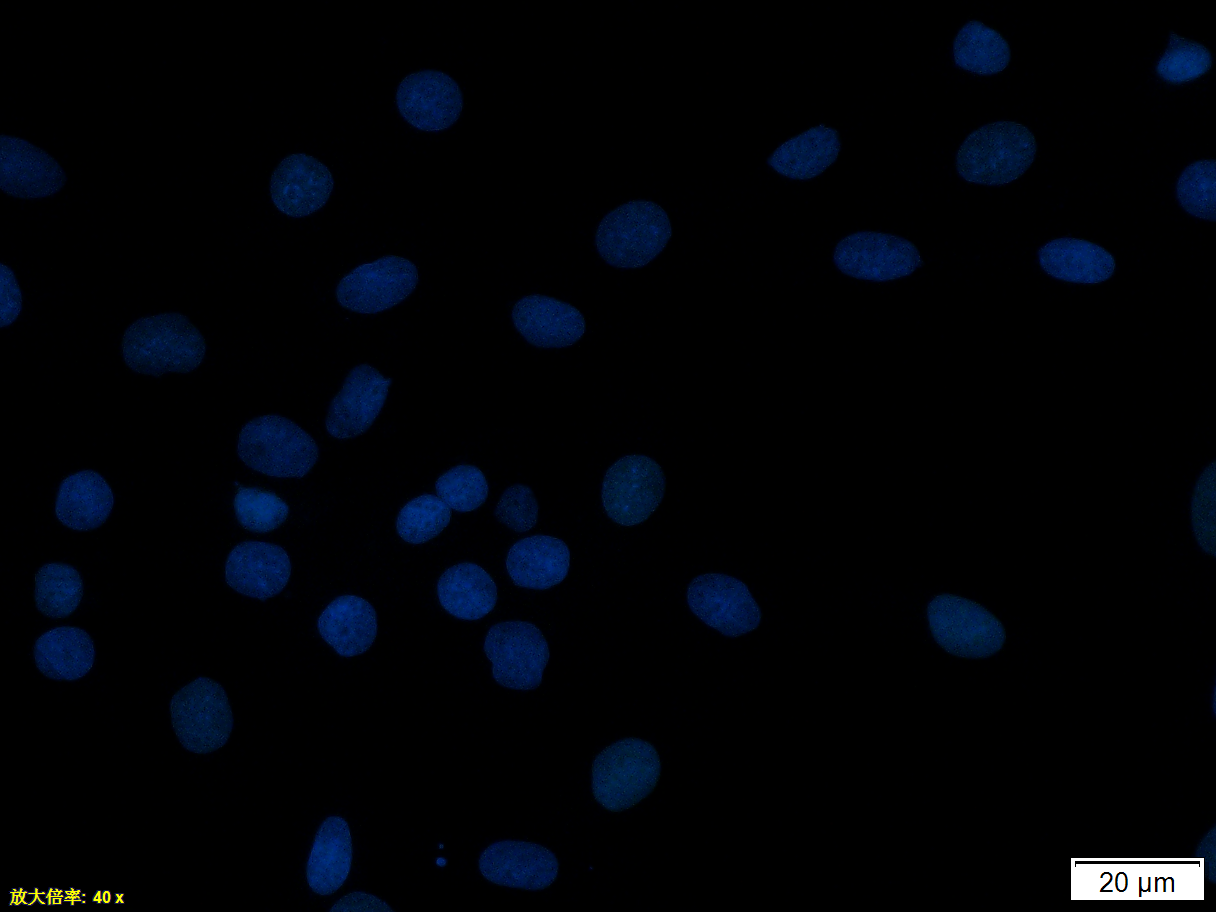

Supplement: S6 File — (ZIP) [file pone.0191616.s006.zip › Original data underlying the findings described in manuscript-TUNEL staining for detecting the apoptosis of CSCs-1/H2O2 group/fig_5-1.tif]

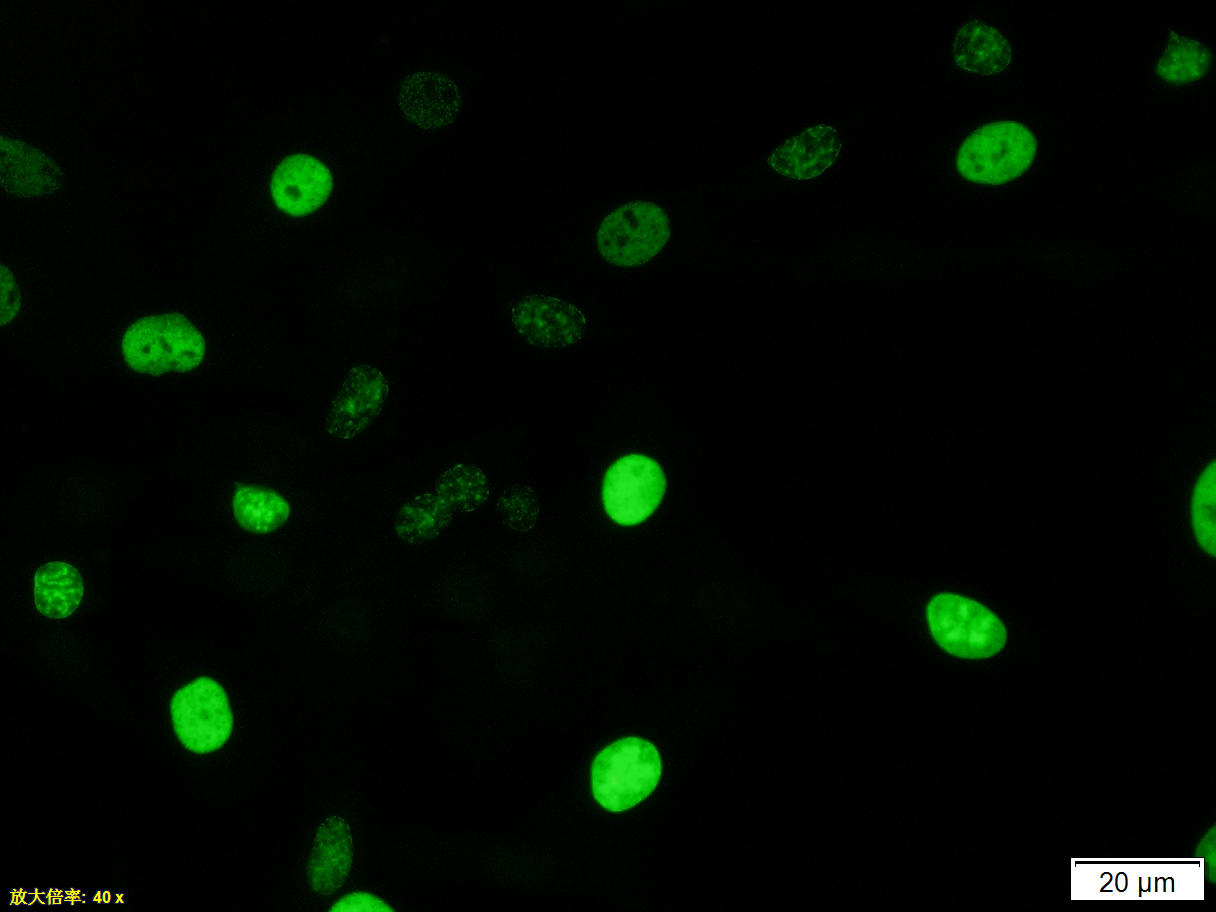

Supplement: S6 File — (ZIP) [file pone.0191616.s006.zip › Original data underlying the findings described in manuscript-TUNEL staining for detecting the apoptosis of CSCs-1/H2O2 group/fig_5-2.tif]

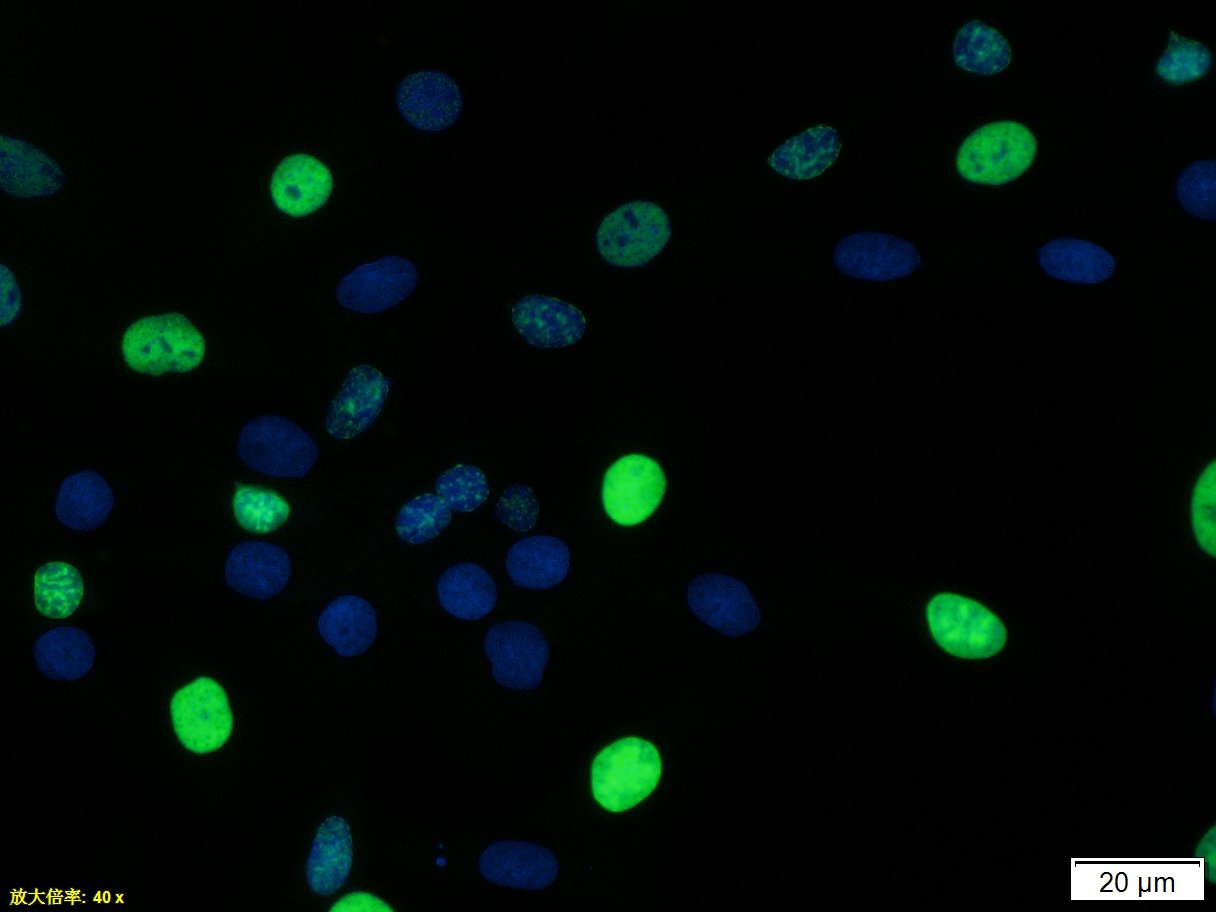

Supplement: S6 File — (ZIP) [file pone.0191616.s006.zip › Original data underlying the findings described in manuscript-TUNEL staining for detecting the apoptosis of CSCs-1/H2O2 group/fig_5.tif]

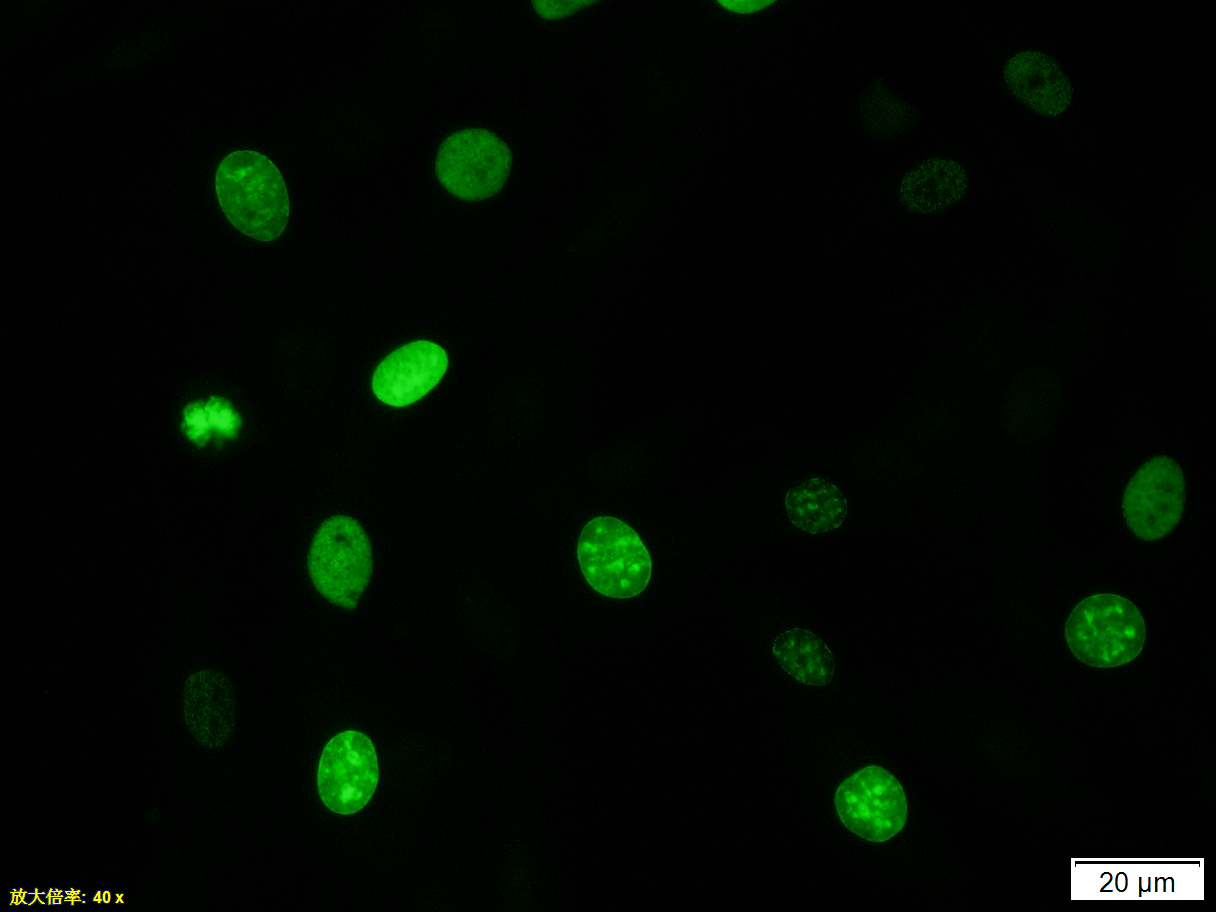

Supplement: S6 File — (ZIP) [file pone.0191616.s006.zip › Original data underlying the findings described in manuscript-TUNEL staining for detecting the apoptosis of CSCs-1/H2O2 group/fig_6-1.tif]

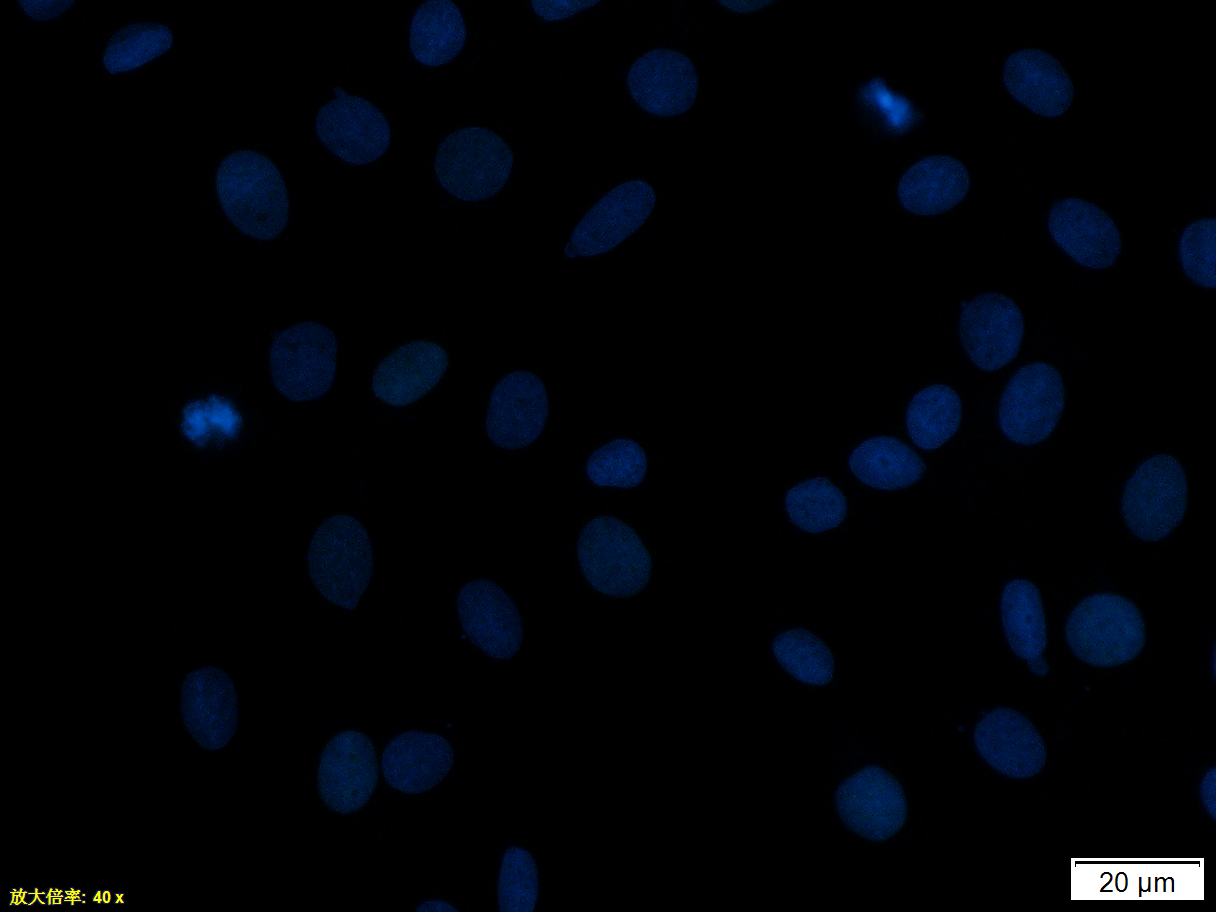

Supplement: S6 File — (ZIP) [file pone.0191616.s006.zip › Original data underlying the findings described in manuscript-TUNEL staining for detecting the apoptosis of CSCs-1/H2O2 group/fig_6-2.tif]

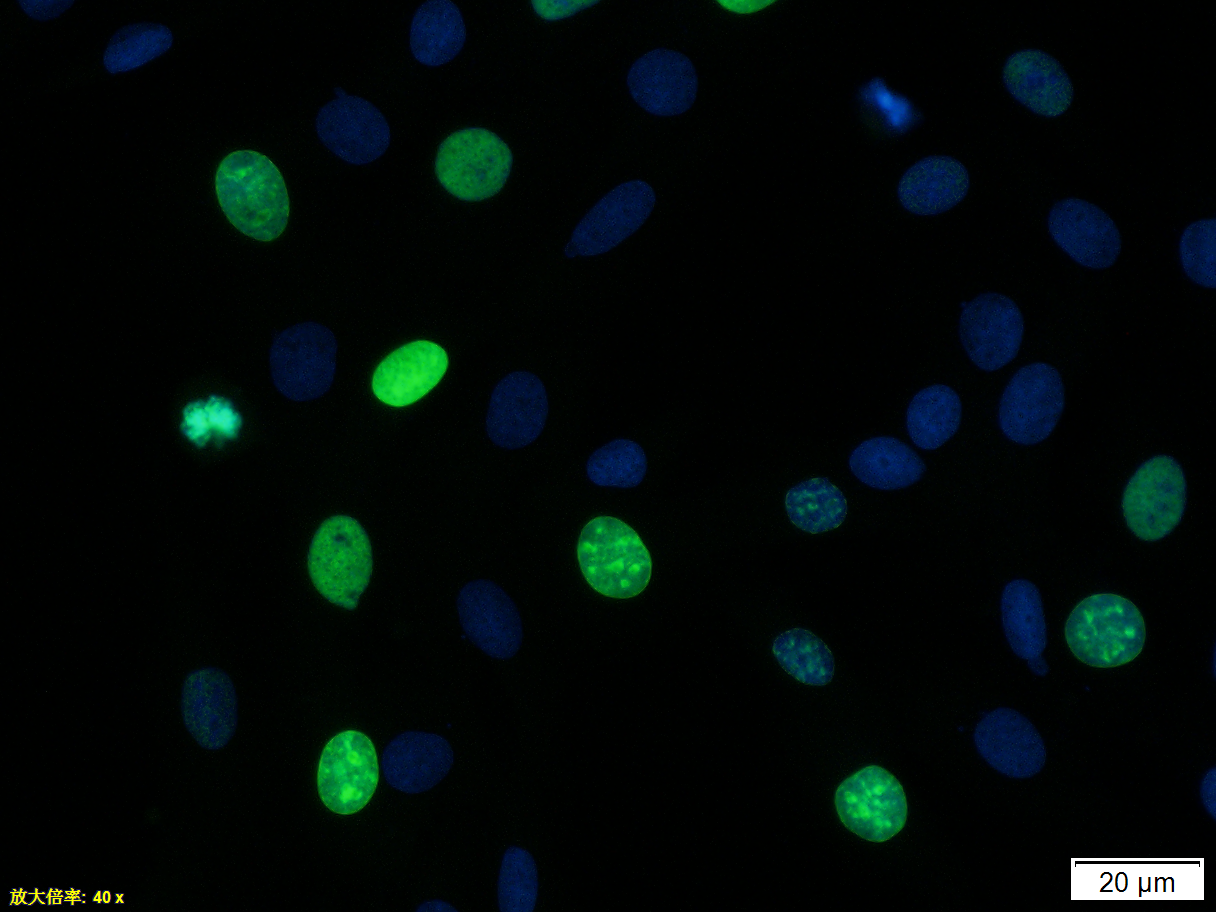

Supplement: S6 File — (ZIP) [file pone.0191616.s006.zip › Original data underlying the findings described in manuscript-TUNEL staining for detecting the apoptosis of CSCs-1/H2O2 group/fig_6.tif]

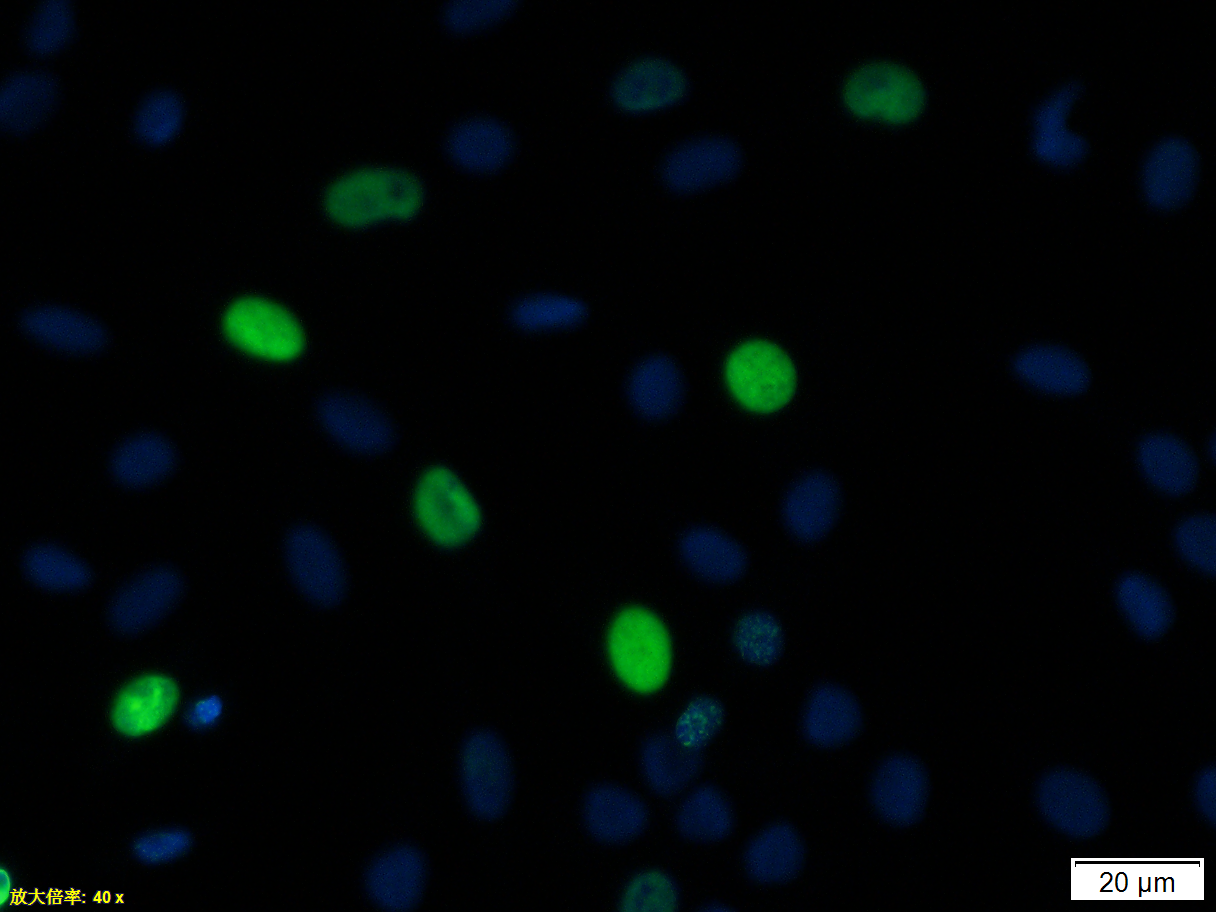

Supplement: S6 File — (ZIP) [file pone.0191616.s006.zip › Original data underlying the findings described in manuscript-TUNEL staining for detecting the apoptosis of CSCs-1/I-Exo group/fig_02.tif]

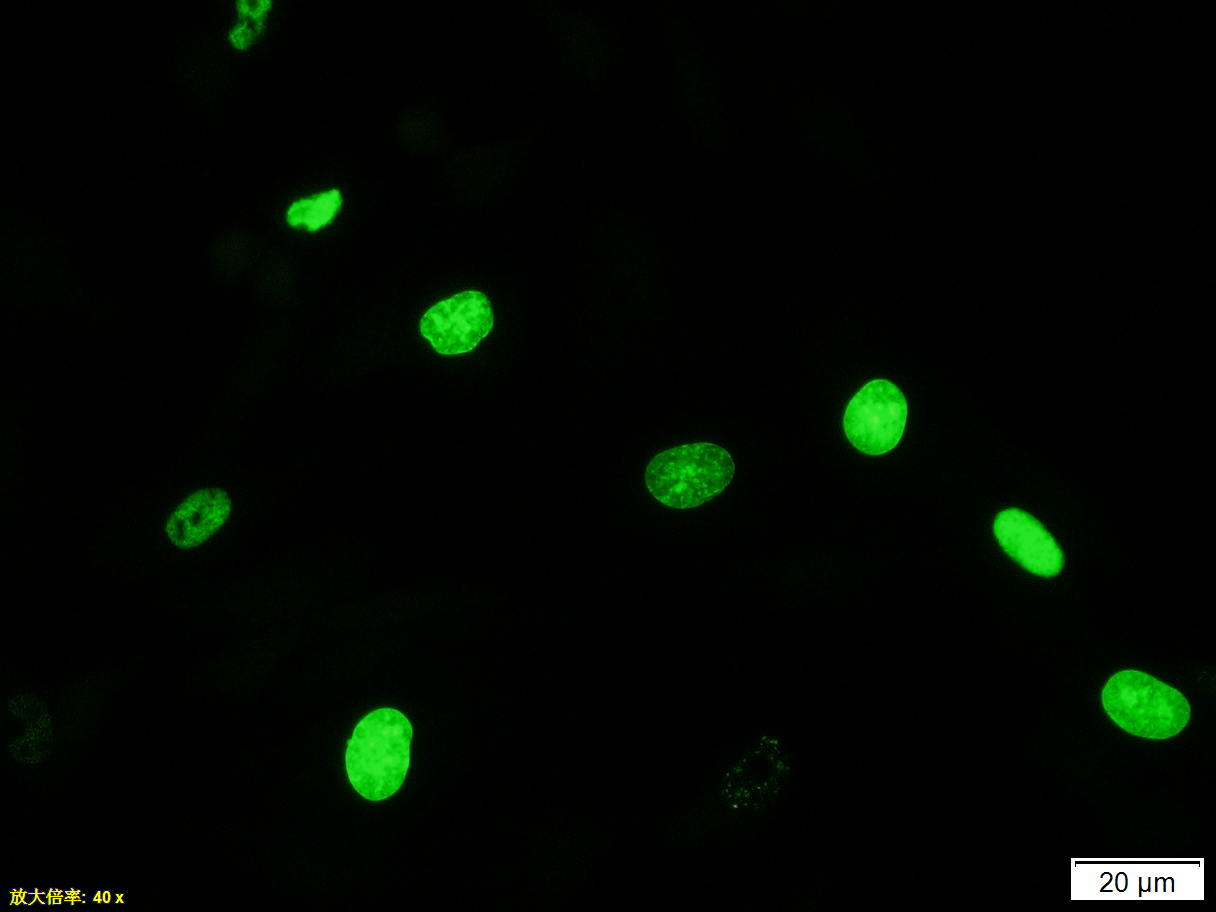

Supplement: S6 File — (ZIP) [file pone.0191616.s006.zip › Original data underlying the findings described in manuscript-TUNEL staining for detecting the apoptosis of CSCs-1/I-Exo group/fig_1-1.tif]

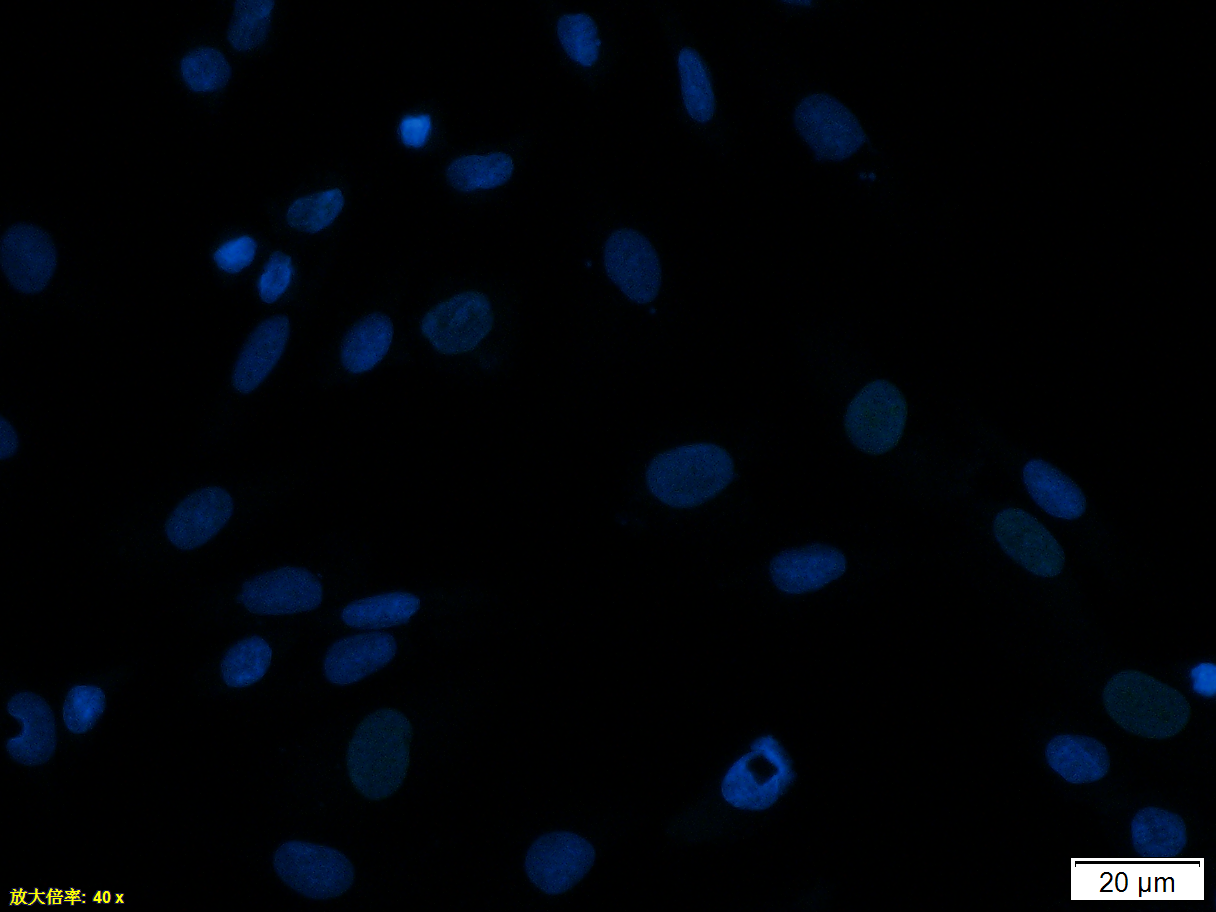

Supplement: S6 File — (ZIP) [file pone.0191616.s006.zip › Original data underlying the findings described in manuscript-TUNEL staining for detecting the apoptosis of CSCs-1/I-Exo group/fig_1-2.tif]

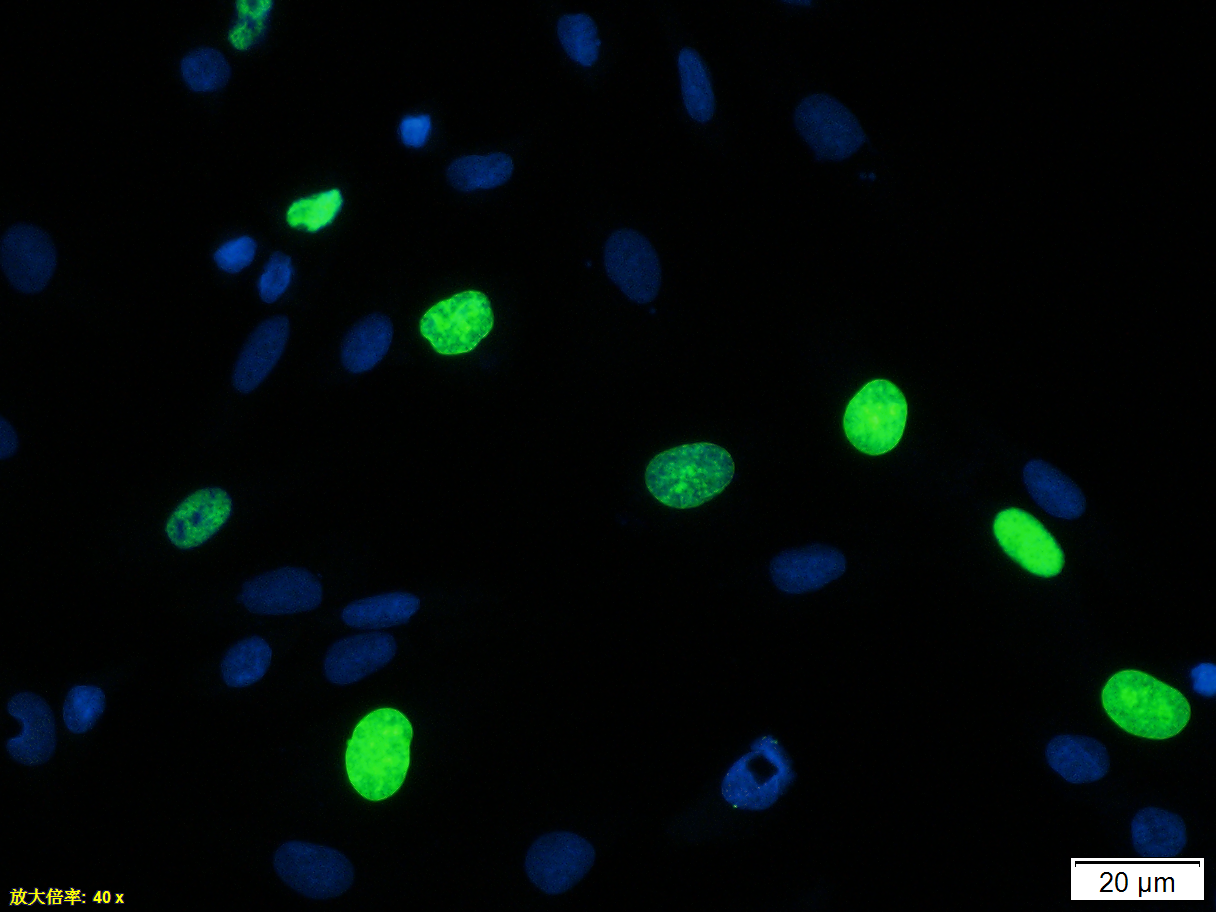

Supplement: S6 File — (ZIP) [file pone.0191616.s006.zip › Original data underlying the findings described in manuscript-TUNEL staining for detecting the apoptosis of CSCs-1/I-Exo group/fig_1.tif]

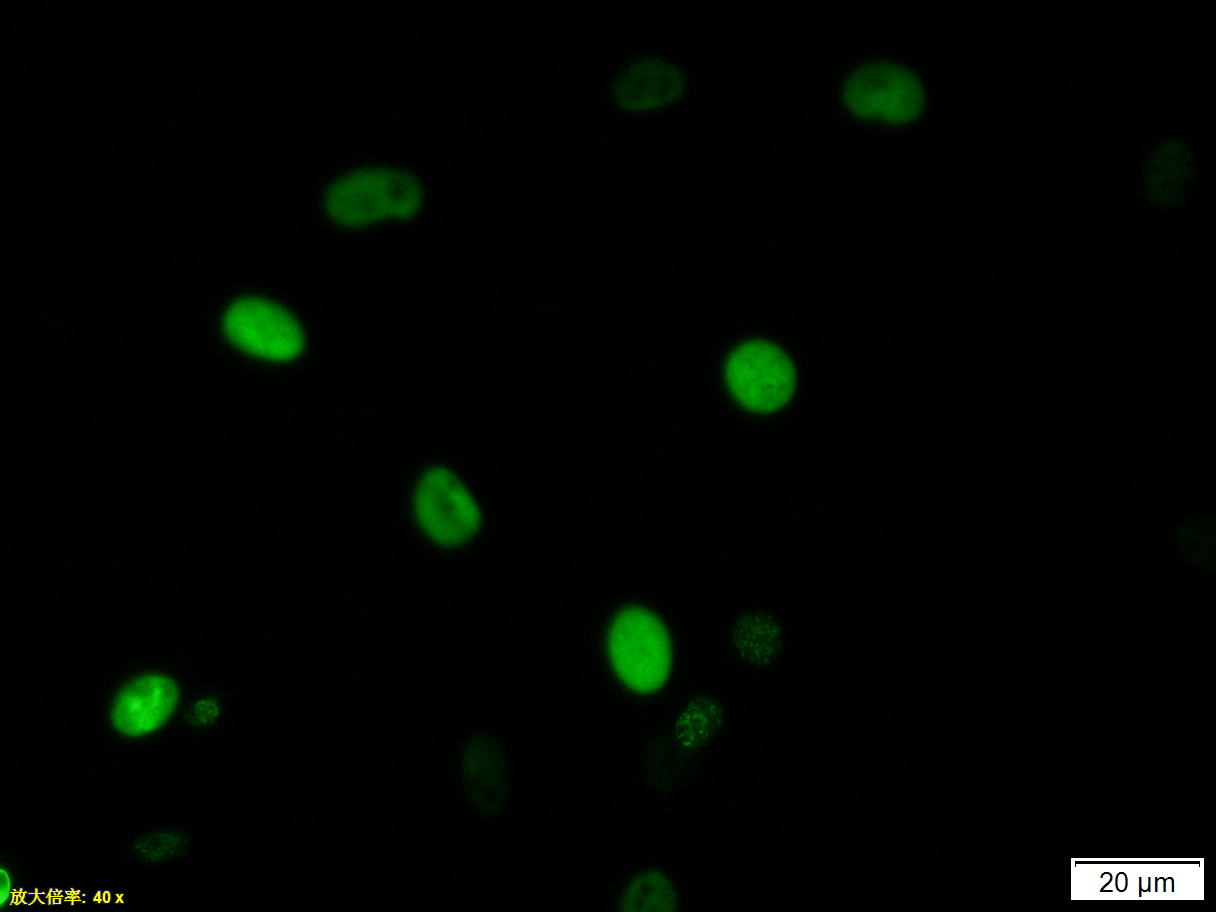

Supplement: S6 File — (ZIP) [file pone.0191616.s006.zip › Original data underlying the findings described in manuscript-TUNEL staining for detecting the apoptosis of CSCs-1/I-Exo group/fig_2-1.tif]

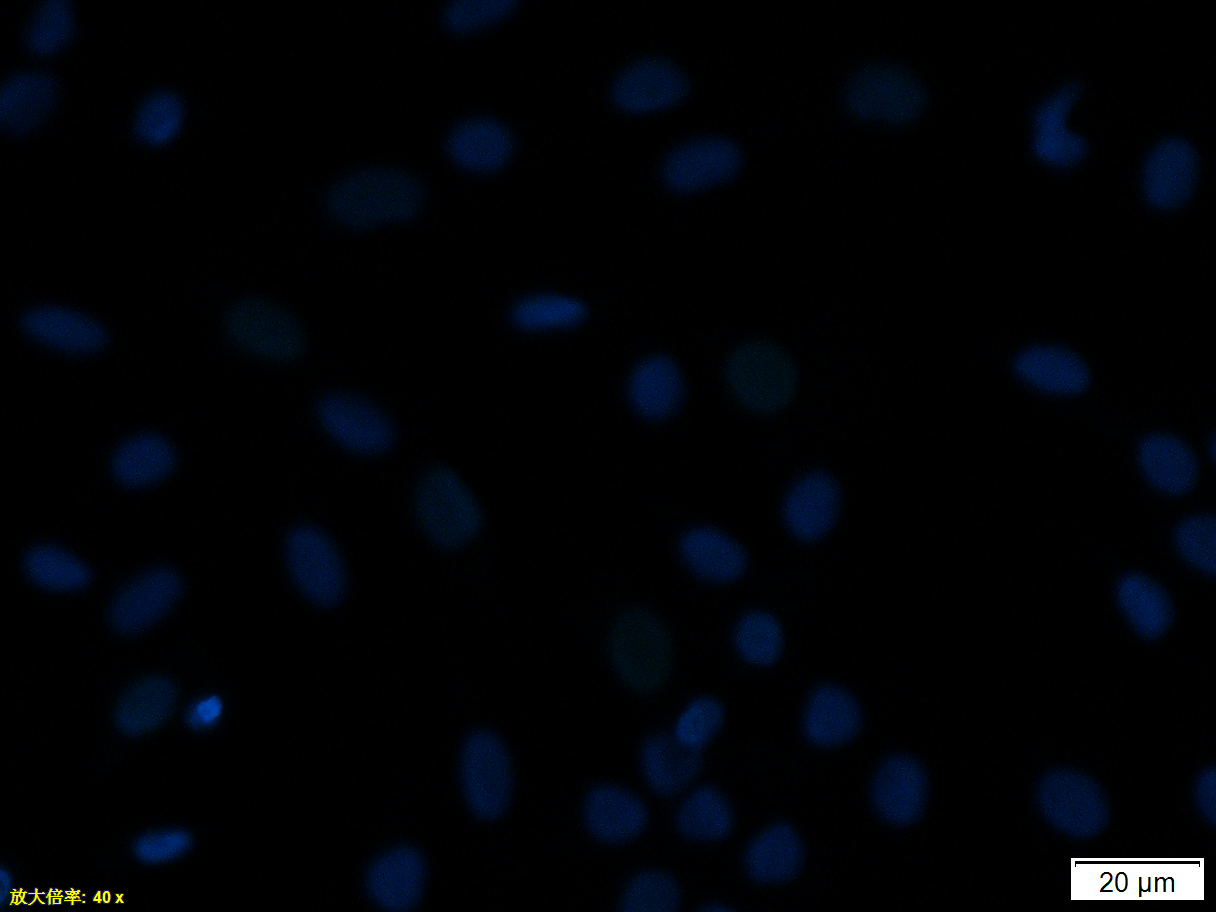

Supplement: S6 File — (ZIP) [file pone.0191616.s006.zip › Original data underlying the findings described in manuscript-TUNEL staining for detecting the apoptosis of CSCs-1/I-Exo group/fig_2-2.tif]

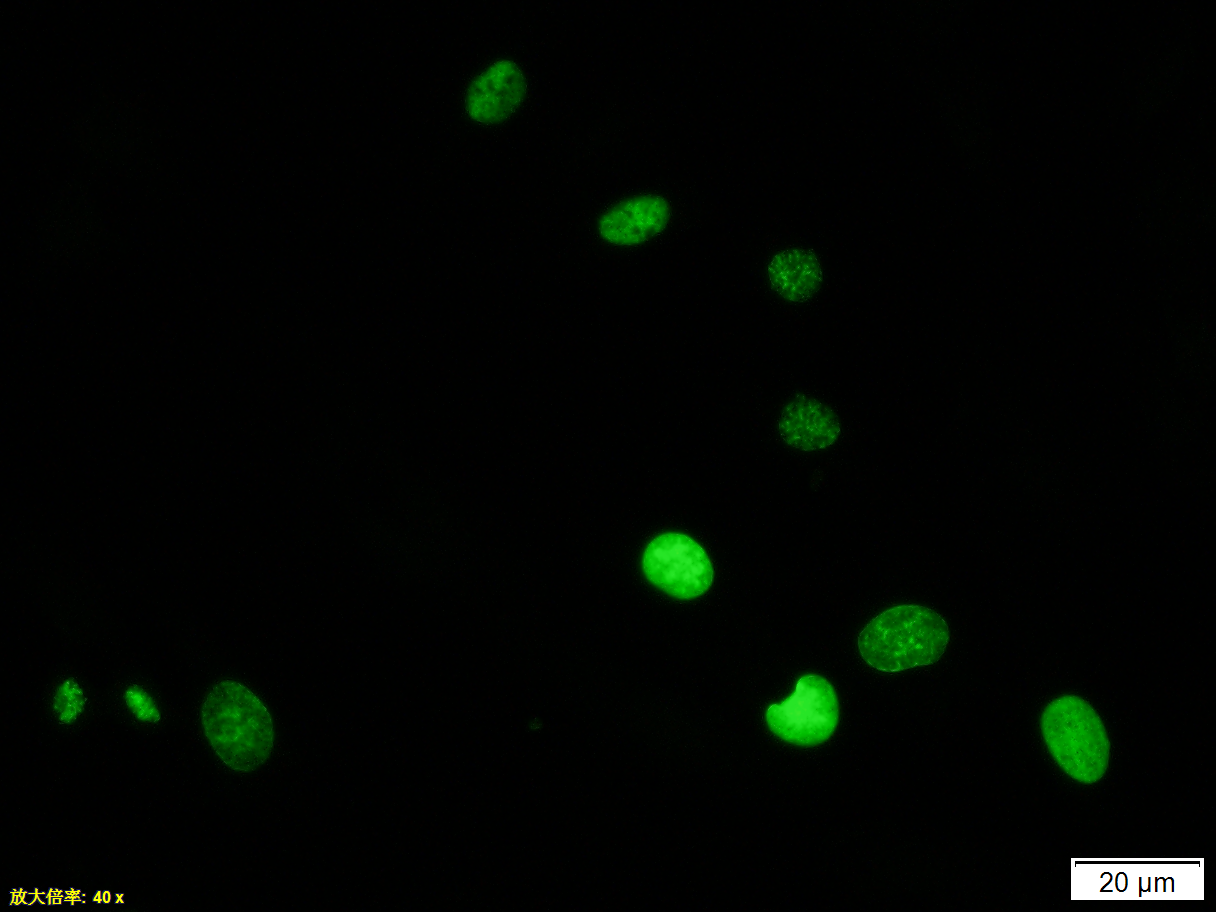

Supplement: S6 File — (ZIP) [file pone.0191616.s006.zip › Original data underlying the findings described in manuscript-TUNEL staining for detecting the apoptosis of CSCs-1/I-Exo group/fig_3-1.tif]

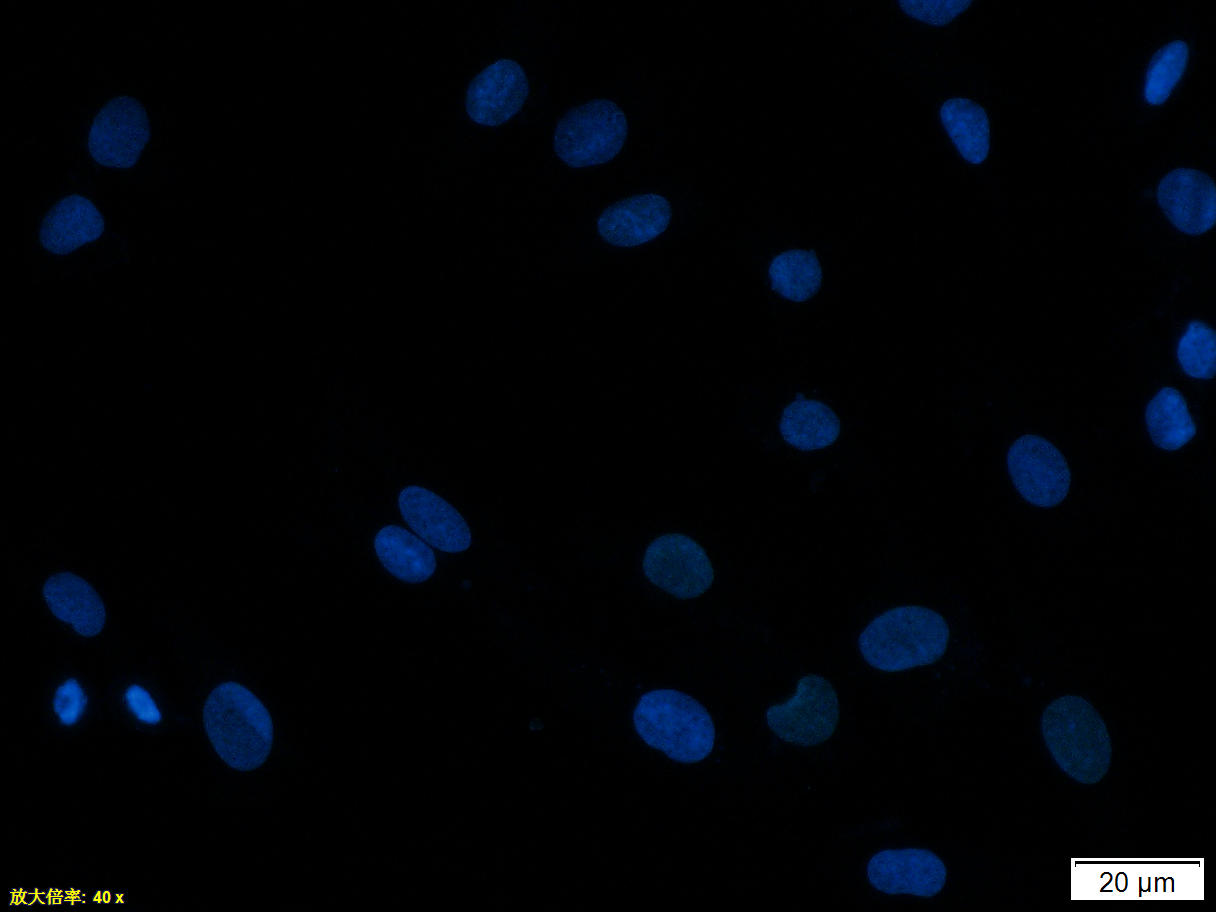

Supplement: S6 File — (ZIP) [file pone.0191616.s006.zip › Original data underlying the findings described in manuscript-TUNEL staining for detecting the apoptosis of CSCs-1/I-Exo group/fig_3-2.tif]

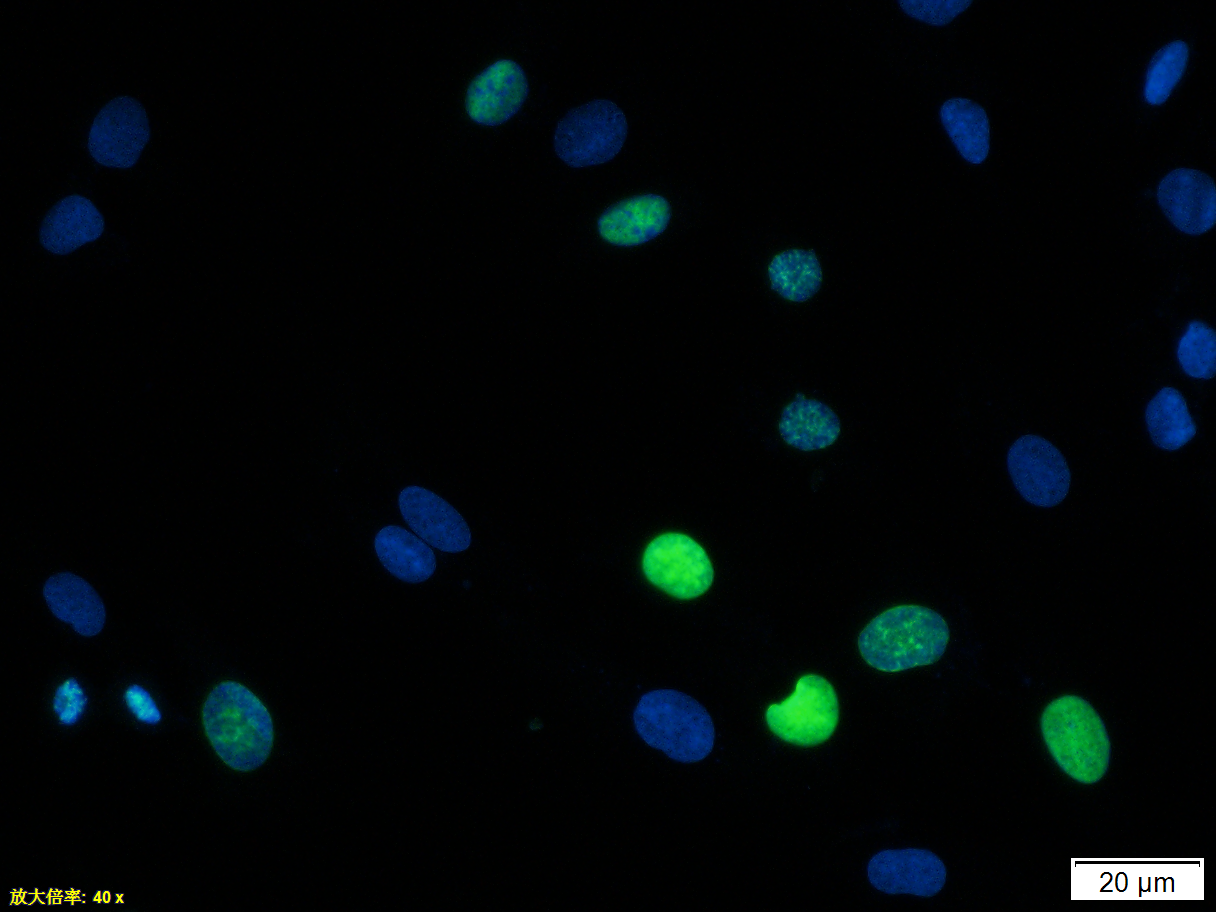

Supplement: S6 File — (ZIP) [file pone.0191616.s006.zip › Original data underlying the findings described in manuscript-TUNEL staining for detecting the apoptosis of CSCs-1/I-Exo group/fig_3.tif]

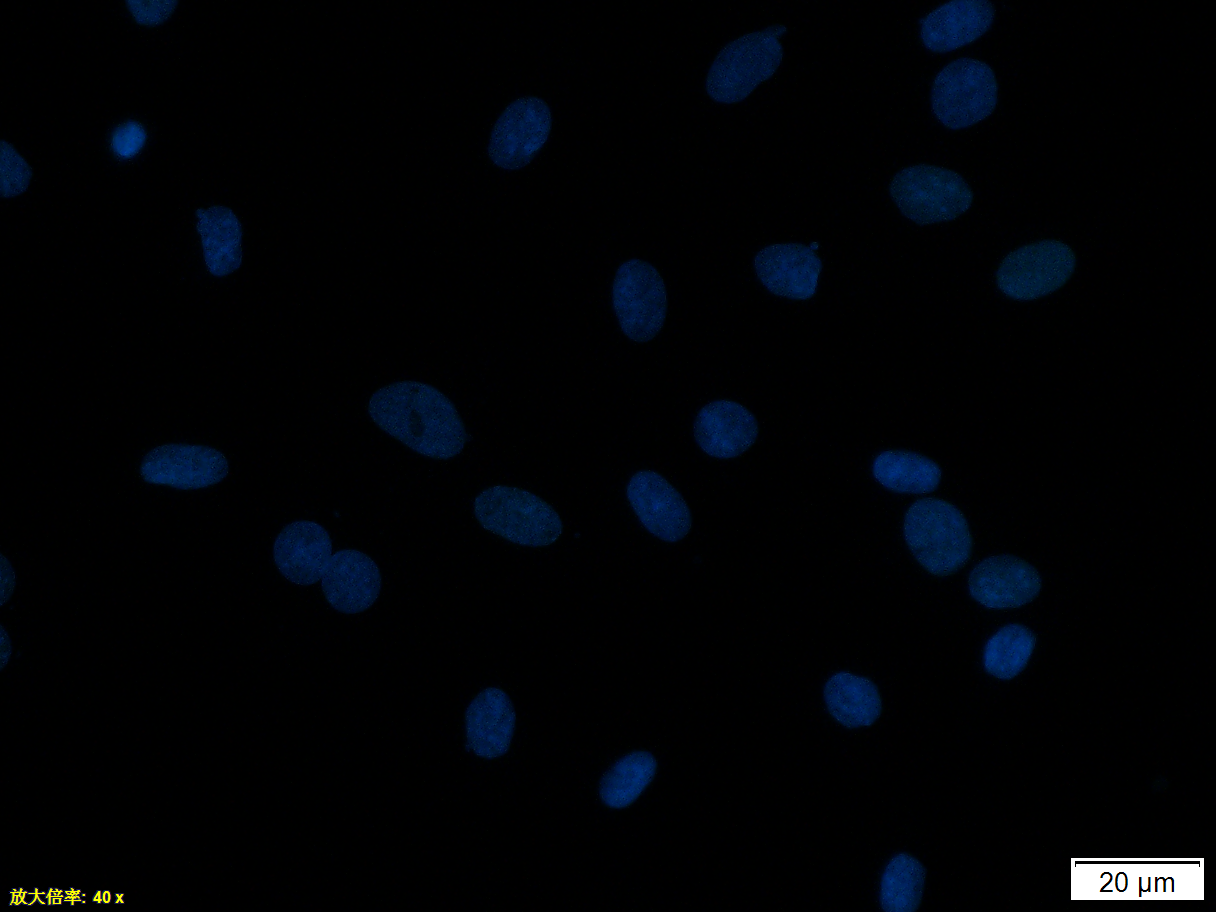

Supplement: S6 File — (ZIP) [file pone.0191616.s006.zip › Original data underlying the findings described in manuscript-TUNEL staining for detecting the apoptosis of CSCs-1/I-Exo group/fig_4-1.tif]

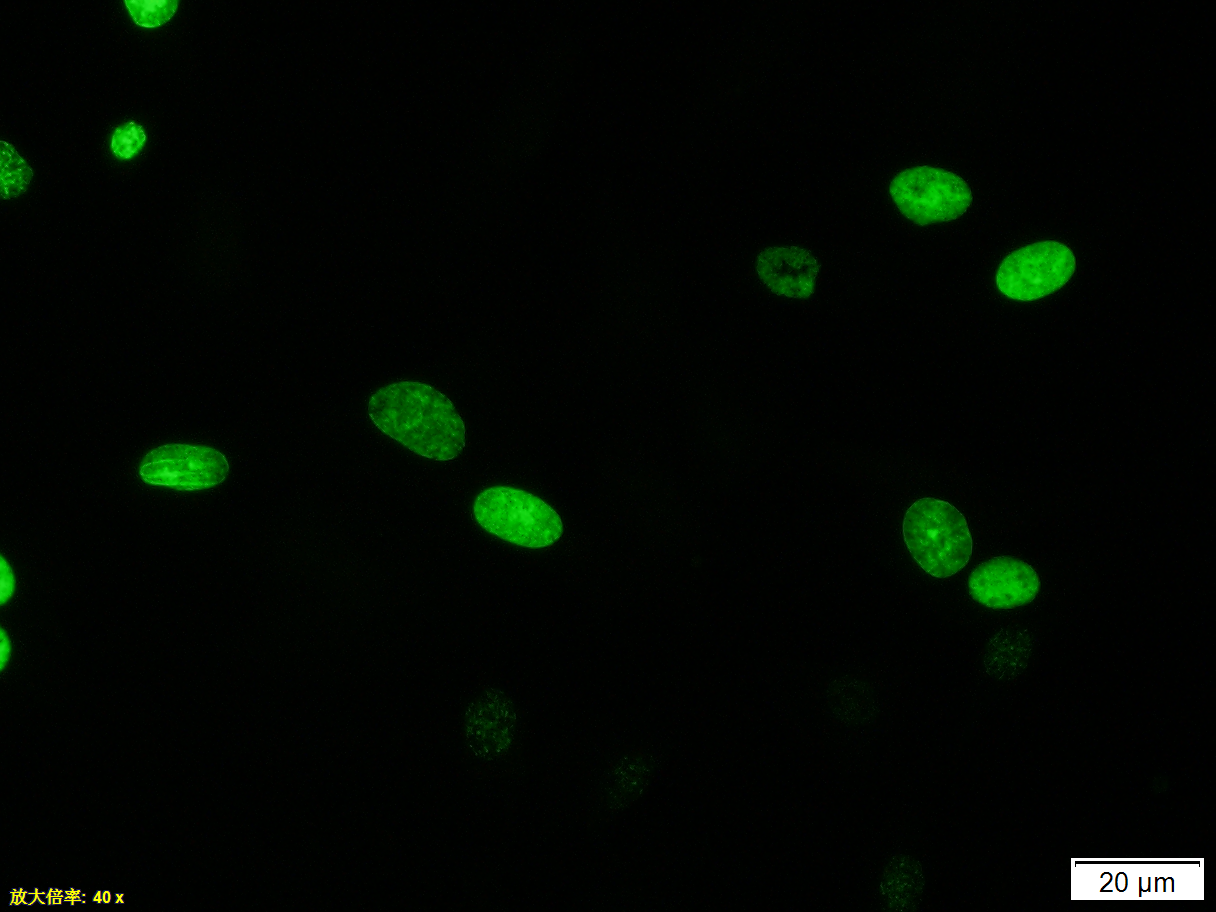

Supplement: S6 File — (ZIP) [file pone.0191616.s006.zip › Original data underlying the findings described in manuscript-TUNEL staining for detecting the apoptosis of CSCs-1/I-Exo group/fig_4-2.tif]

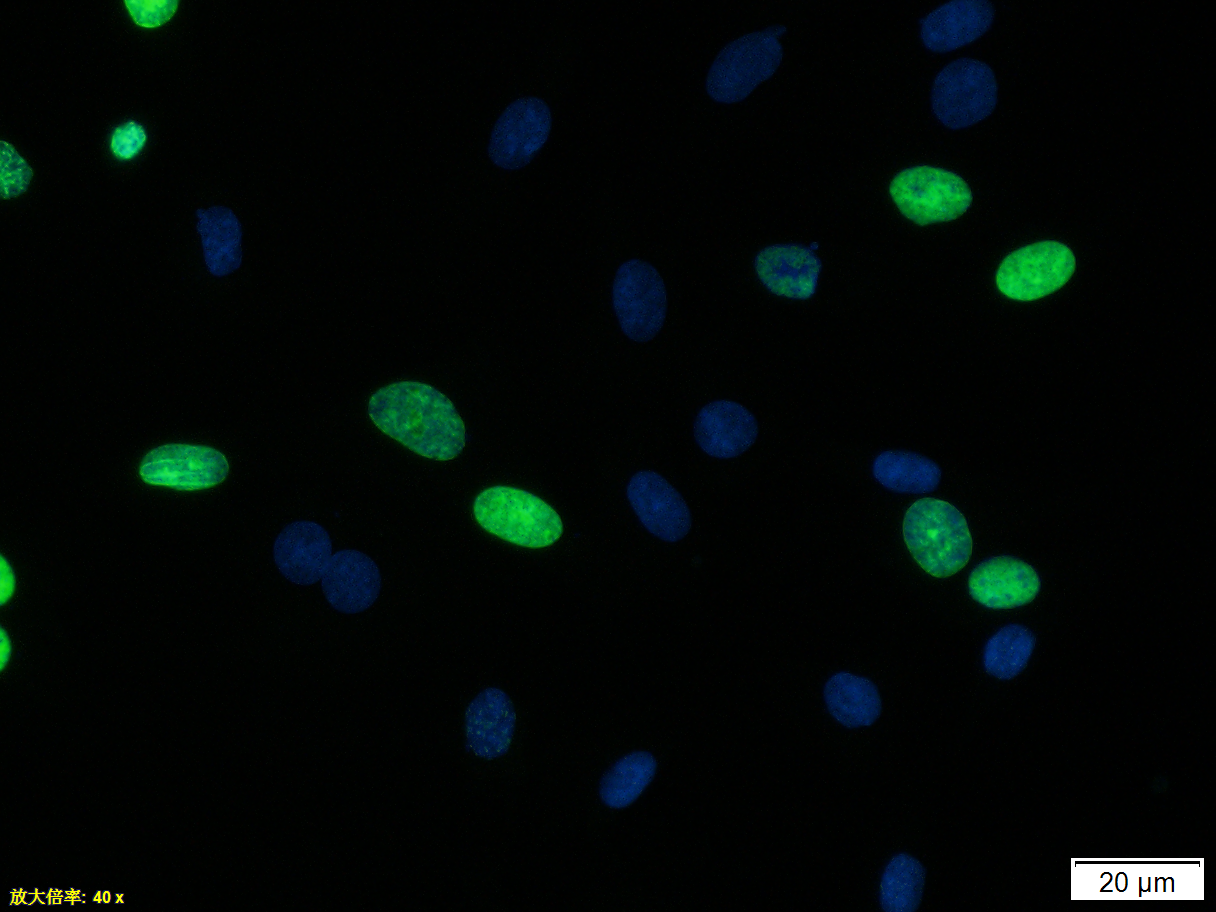

Supplement: S6 File — (ZIP) [file pone.0191616.s006.zip › Original data underlying the findings described in manuscript-TUNEL staining for detecting the apoptosis of CSCs-1/I-Exo group/fig_4.tif]

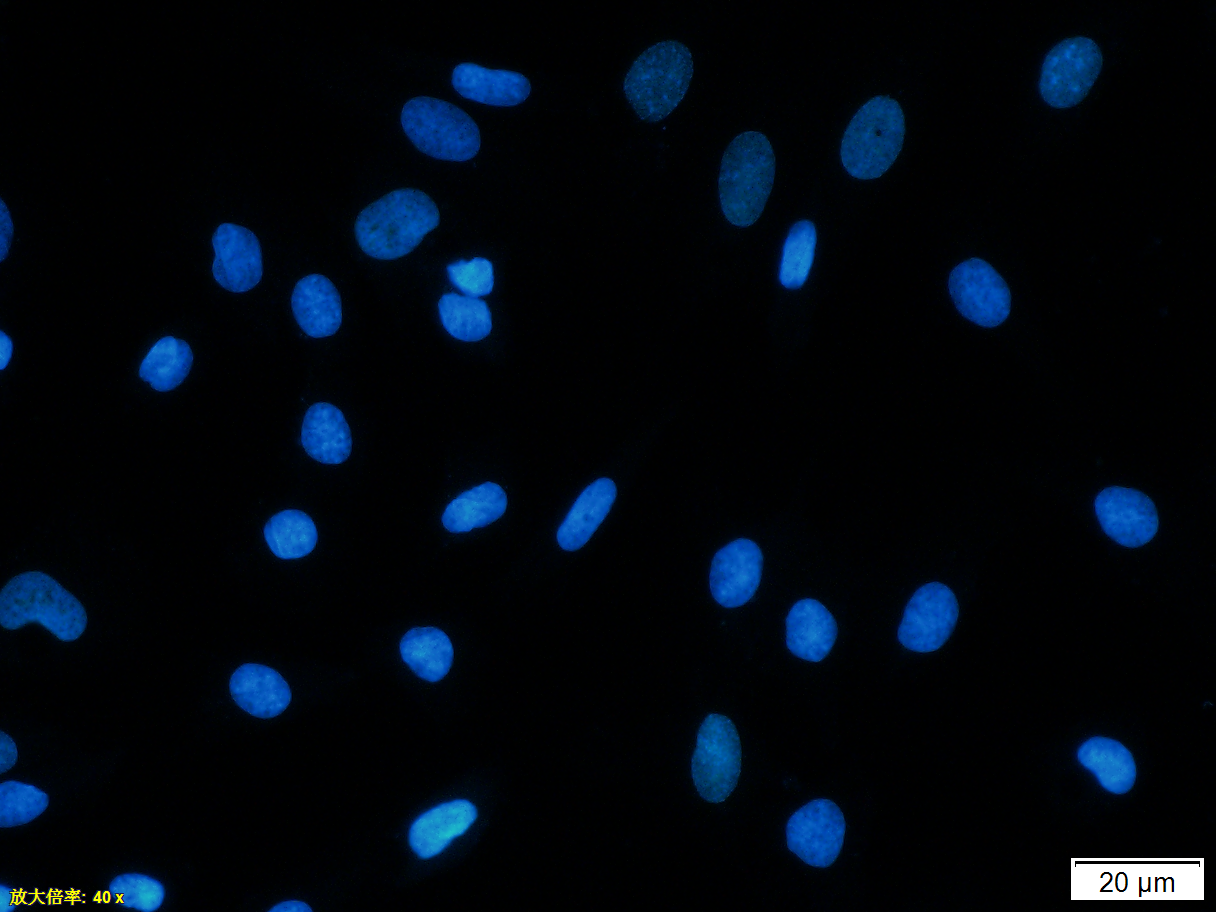

Supplement: S6 File — (ZIP) [file pone.0191616.s006.zip › Original data underlying the findings described in manuscript-TUNEL staining for detecting the apoptosis of CSCs-1/I-Exo group/fig_5-1.tif]

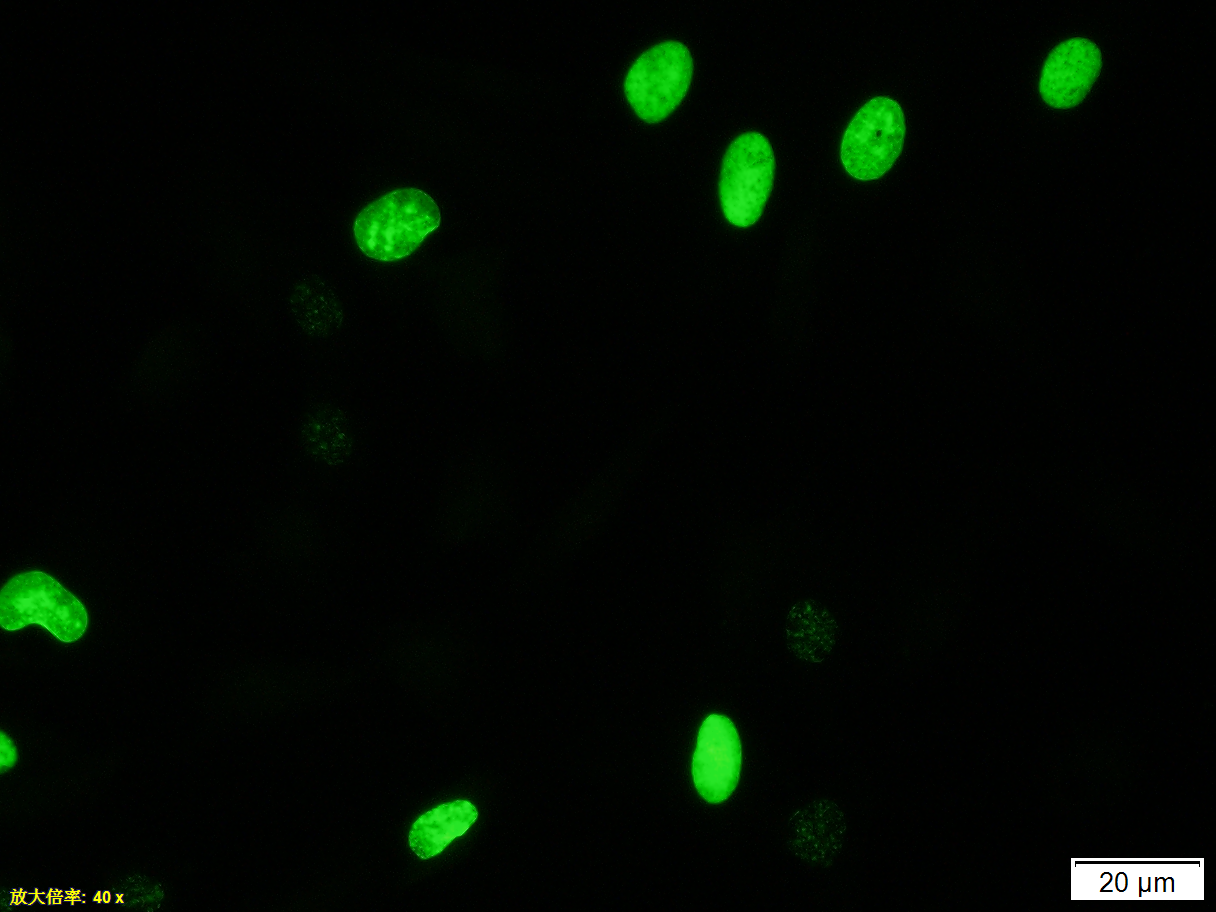

Supplement: S6 File — (ZIP) [file pone.0191616.s006.zip › Original data underlying the findings described in manuscript-TUNEL staining for detecting the apoptosis of CSCs-1/I-Exo group/fig_5-2.tif]

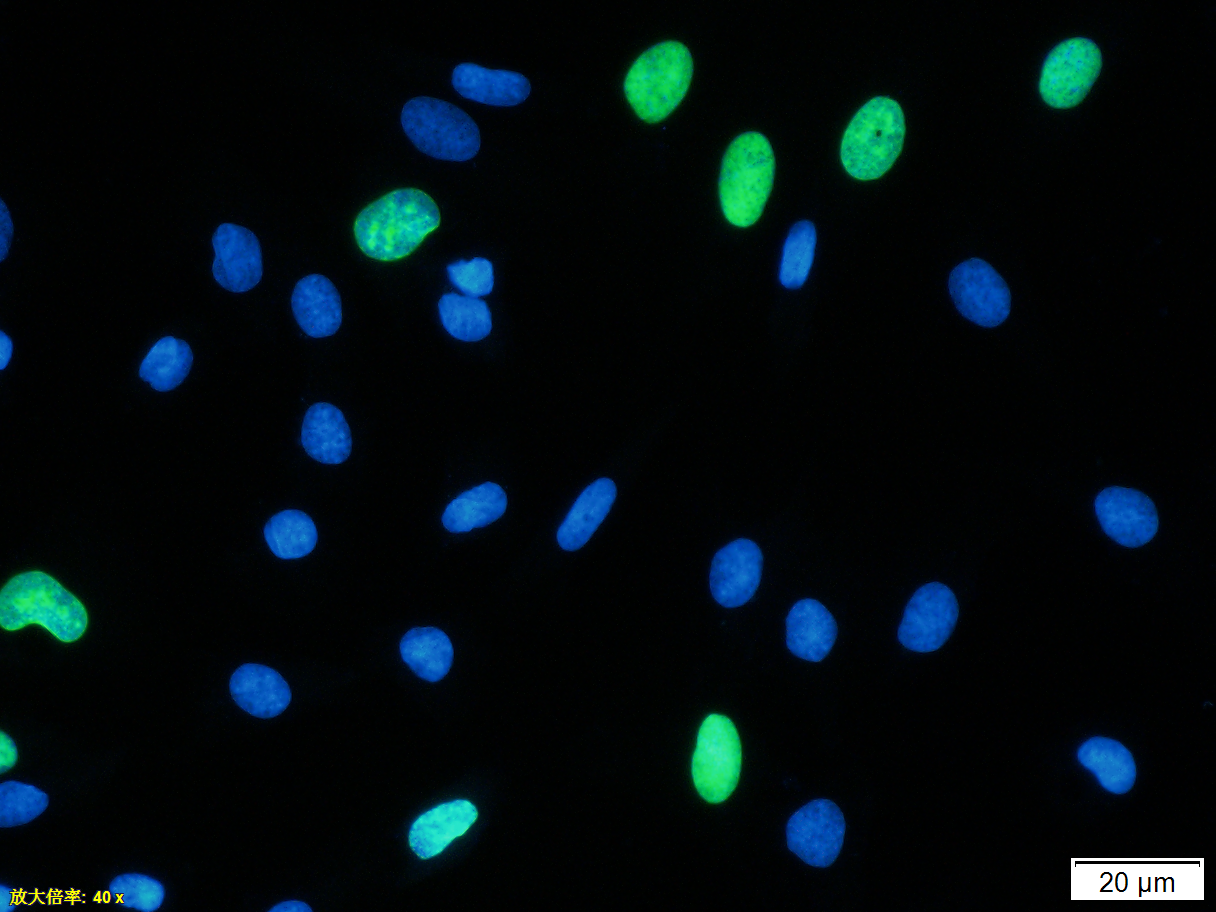

Supplement: S6 File — (ZIP) [file pone.0191616.s006.zip › Original data underlying the findings described in manuscript-TUNEL staining for detecting the apoptosis of CSCs-1/I-Exo group/fig_5.tif]

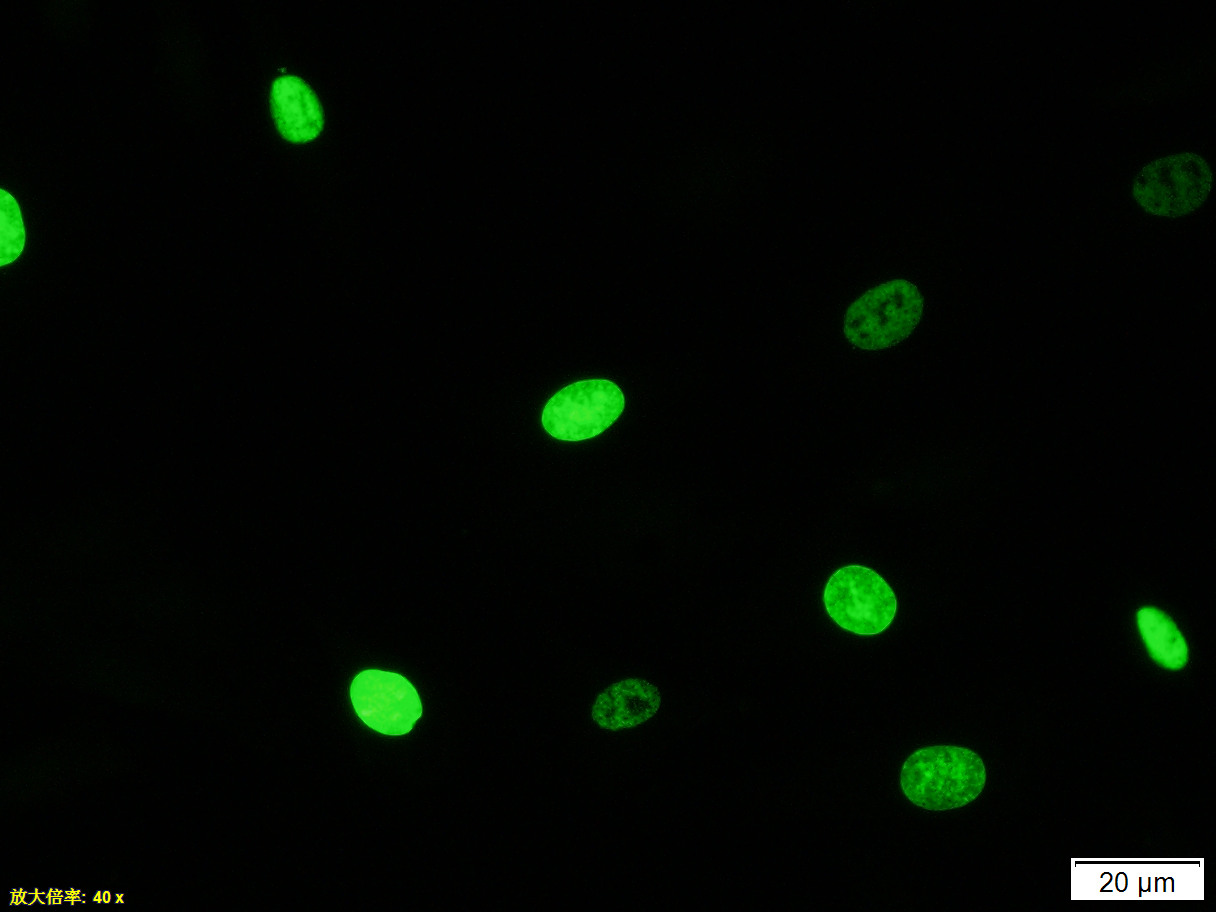

Supplement: S6 File — (ZIP) [file pone.0191616.s006.zip › Original data underlying the findings described in manuscript-TUNEL staining for detecting the apoptosis of CSCs-1/I-Exo group/fig_6-1.tif]

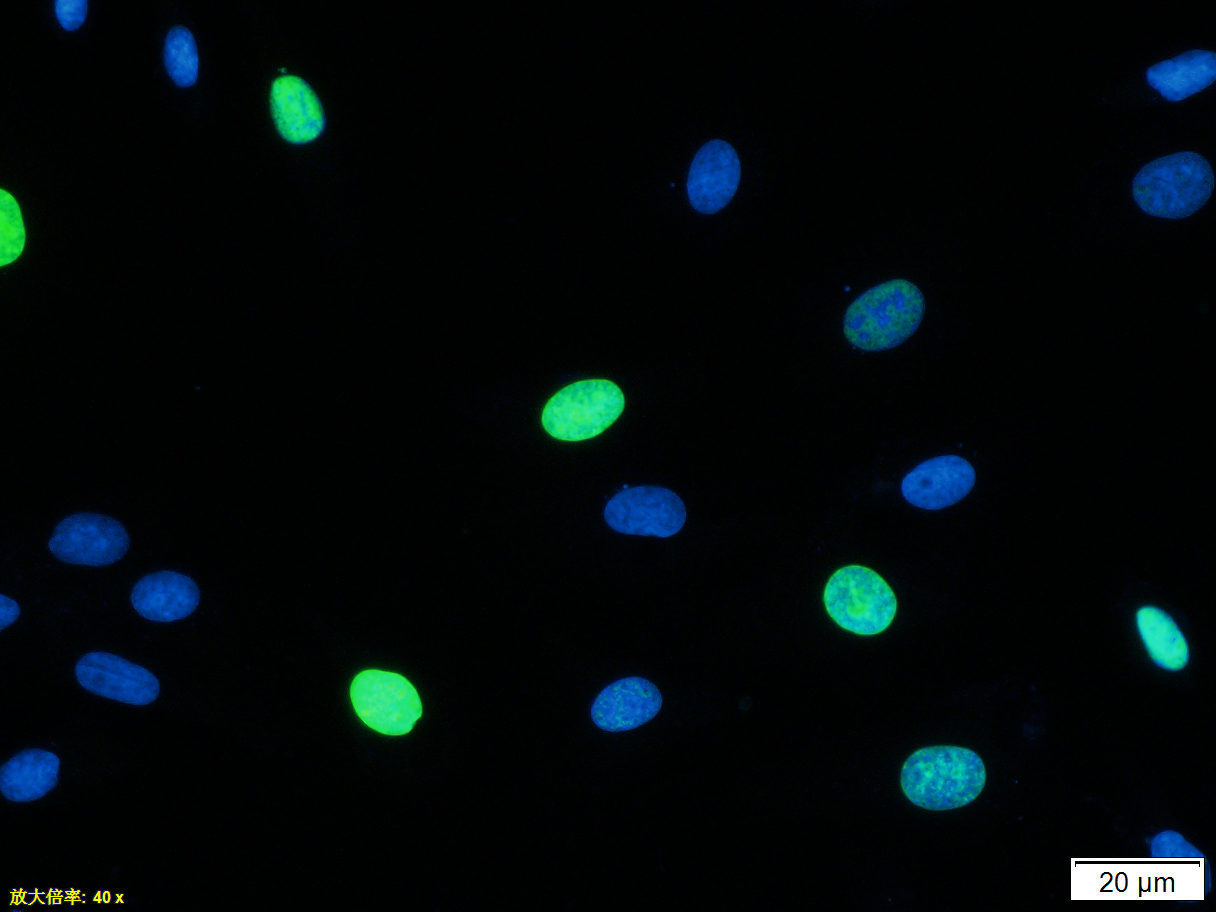

Supplement: S6 File — (ZIP) [file pone.0191616.s006.zip › Original data underlying the findings described in manuscript-TUNEL staining for detecting the apoptosis of CSCs-1/I-Exo group/fig_6.tif]

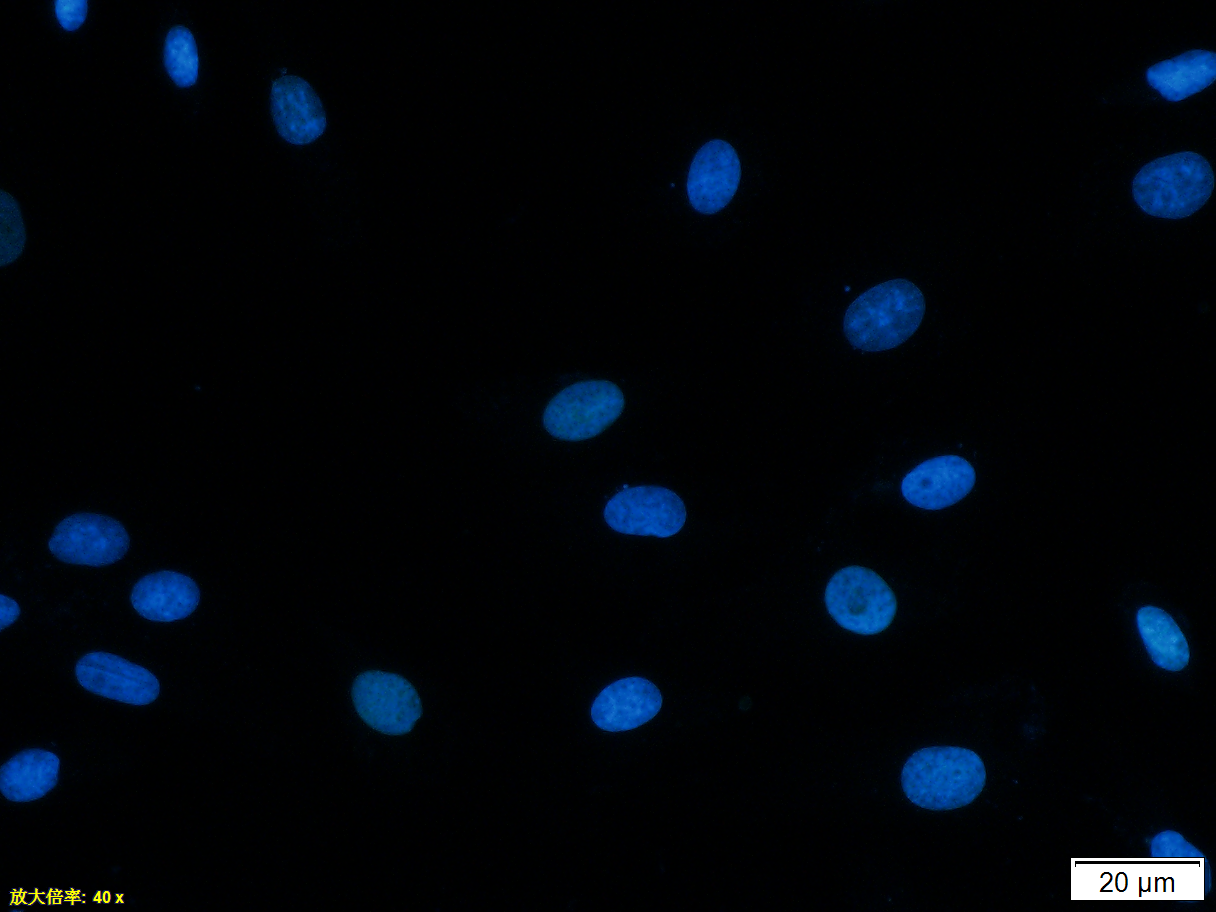

Supplement: S6 File — (ZIP) [file pone.0191616.s006.zip › Original data underlying the findings described in manuscript-TUNEL staining for detecting the apoptosis of CSCs-1/I-Exo group/═╝╧±_6-2.tif]

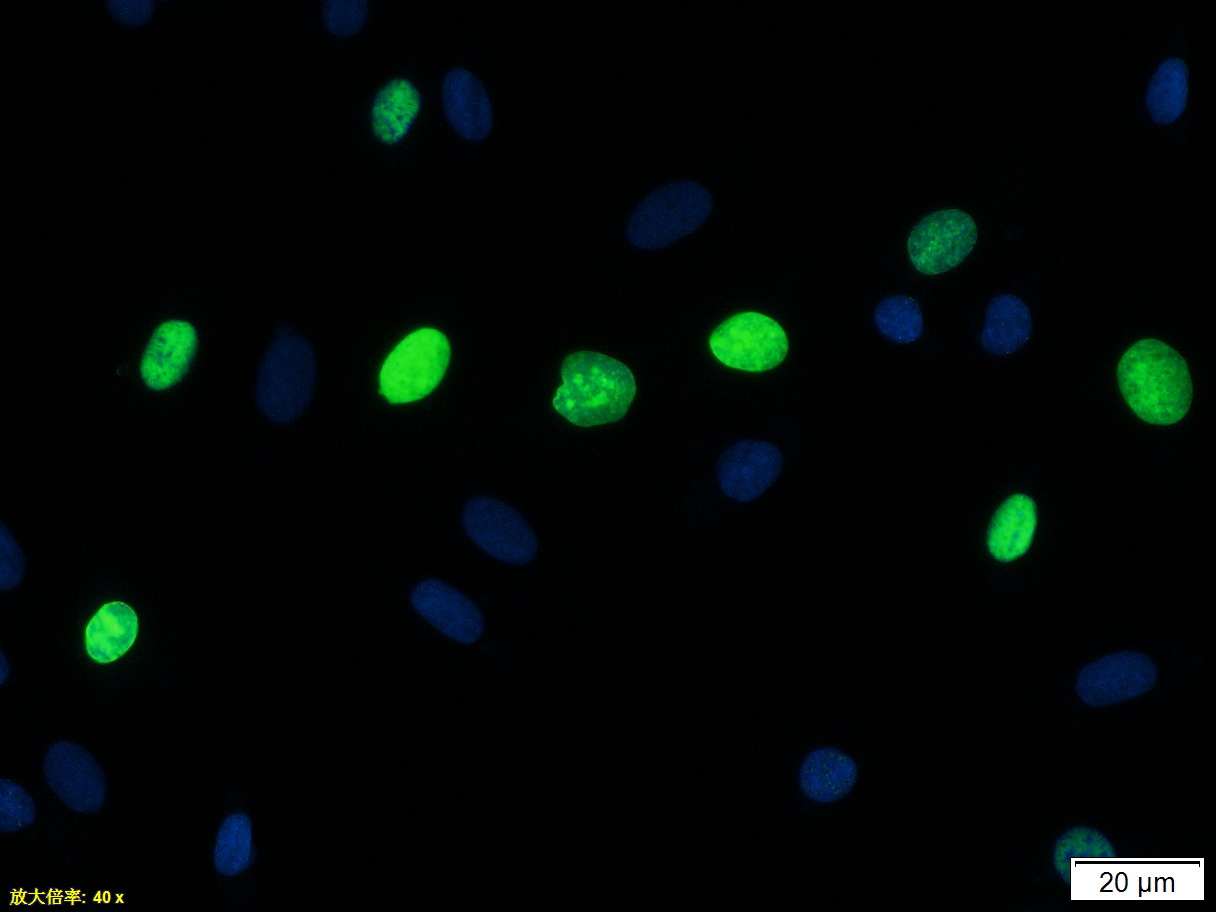

Supplement: S6 File — (ZIP) [file pone.0191616.s006.zip › Original data underlying the findings described in manuscript-TUNEL staining for detecting the apoptosis of CSCs-1/I-Exo+inhibiter group/fig_02.tif]

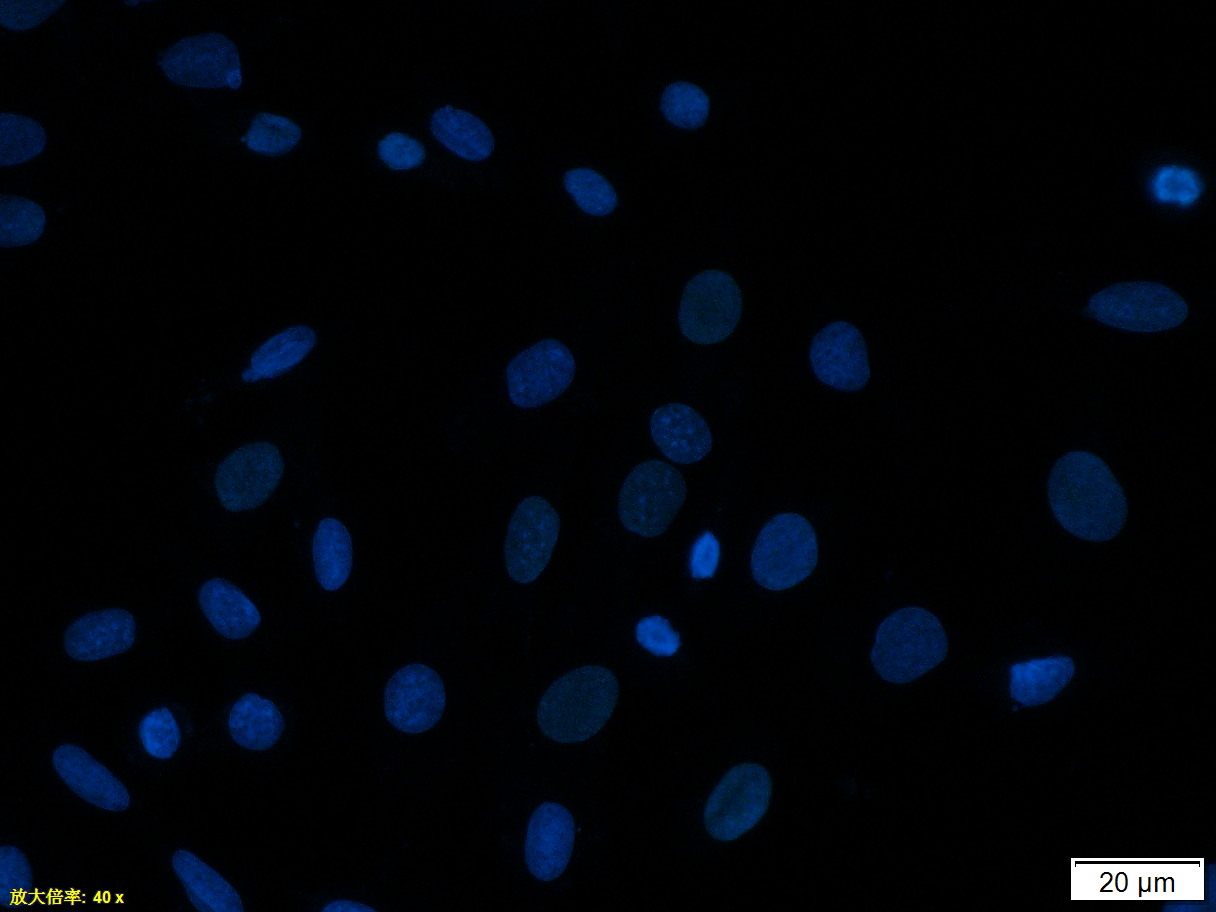

Supplement: S6 File — (ZIP) [file pone.0191616.s006.zip › Original data underlying the findings described in manuscript-TUNEL staining for detecting the apoptosis of CSCs-1/I-Exo+inhibiter group/fig_1-1.tif]

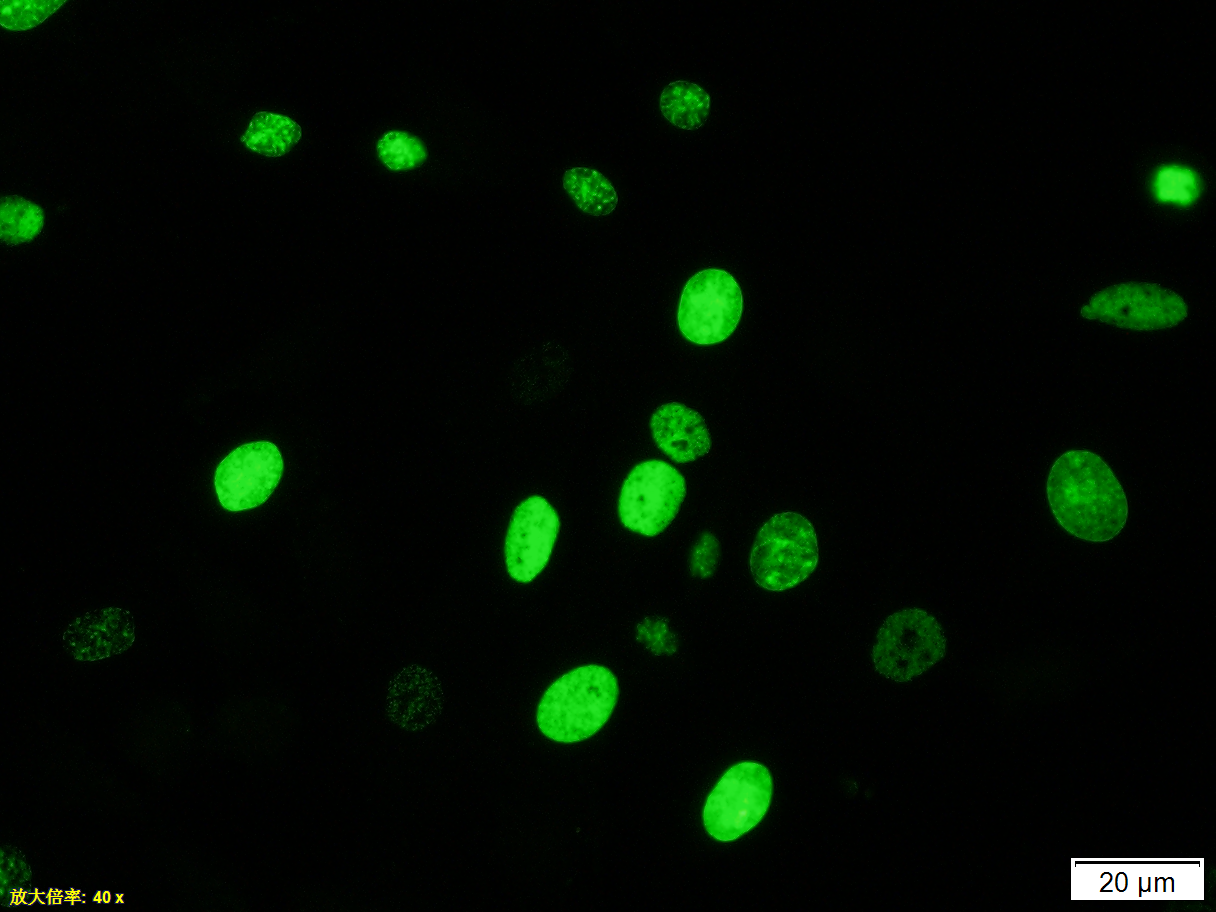

Supplement: S6 File — (ZIP) [file pone.0191616.s006.zip › Original data underlying the findings described in manuscript-TUNEL staining for detecting the apoptosis of CSCs-1/I-Exo+inhibiter group/fig_1-2.tif]

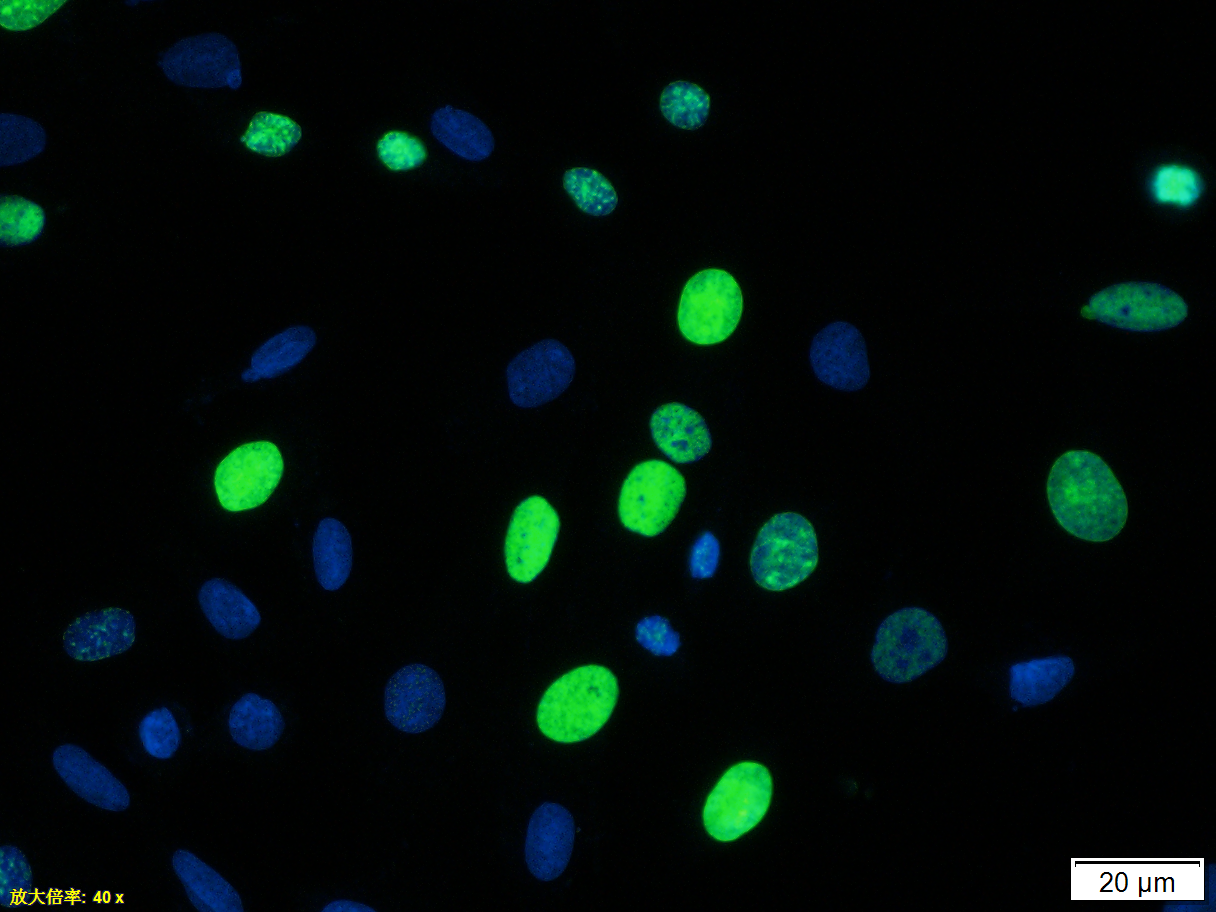

Supplement: S6 File — (ZIP) [file pone.0191616.s006.zip › Original data underlying the findings described in manuscript-TUNEL staining for detecting the apoptosis of CSCs-1/I-Exo+inhibiter group/fig_1.tif]

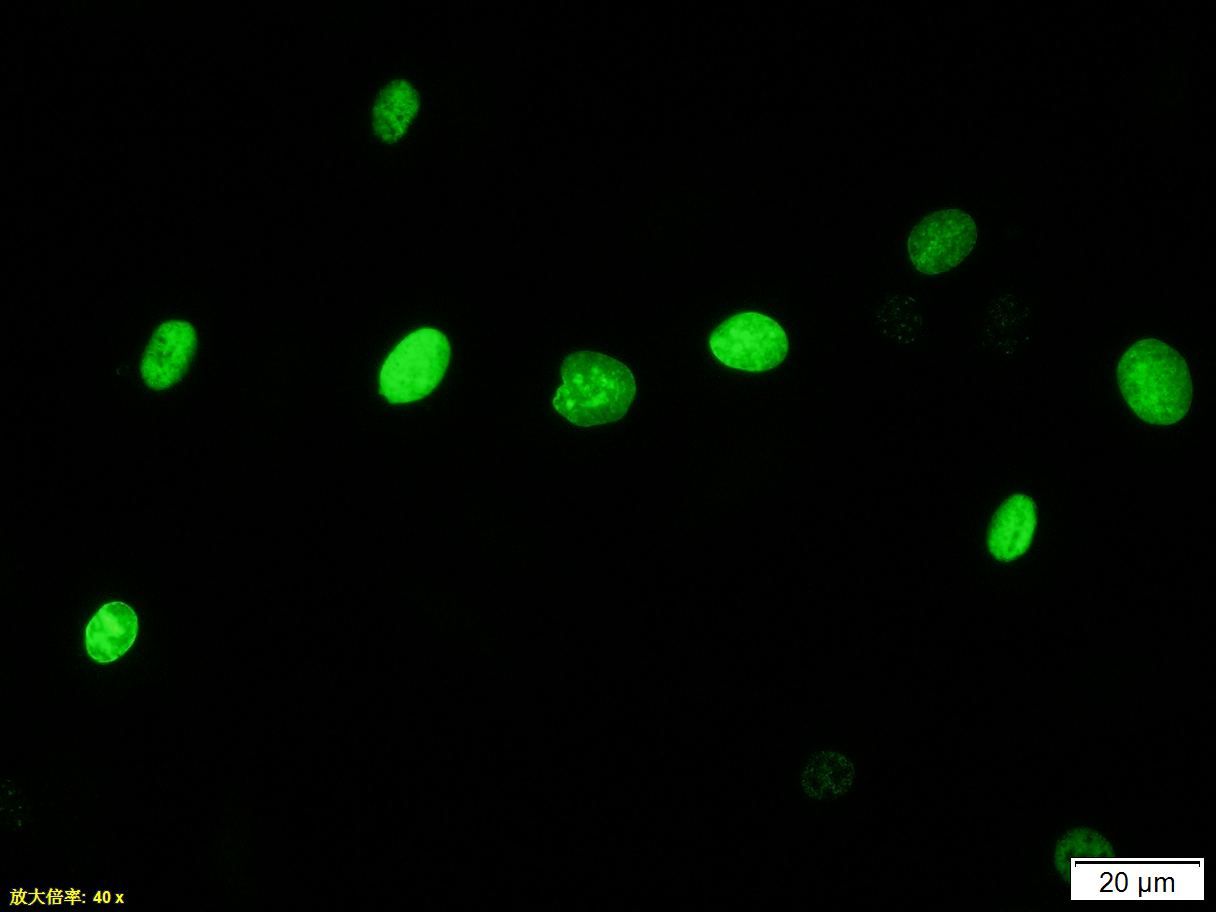

Supplement: S6 File — (ZIP) [file pone.0191616.s006.zip › Original data underlying the findings described in manuscript-TUNEL staining for detecting the apoptosis of CSCs-1/I-Exo+inhibiter group/fig_2-1.tif]

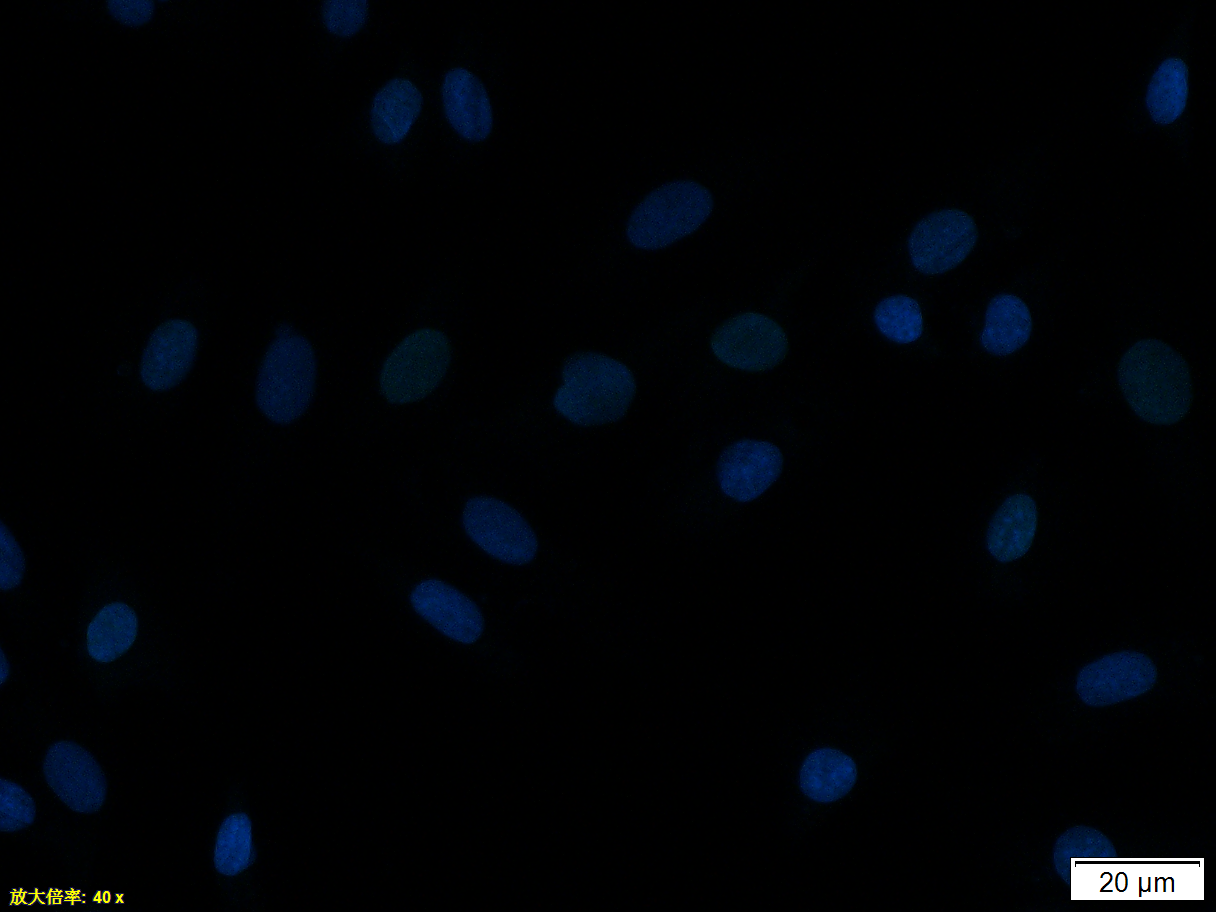

Supplement: S6 File — (ZIP) [file pone.0191616.s006.zip › Original data underlying the findings described in manuscript-TUNEL staining for detecting the apoptosis of CSCs-1/I-Exo+inhibiter group/fig_2-2.tif]

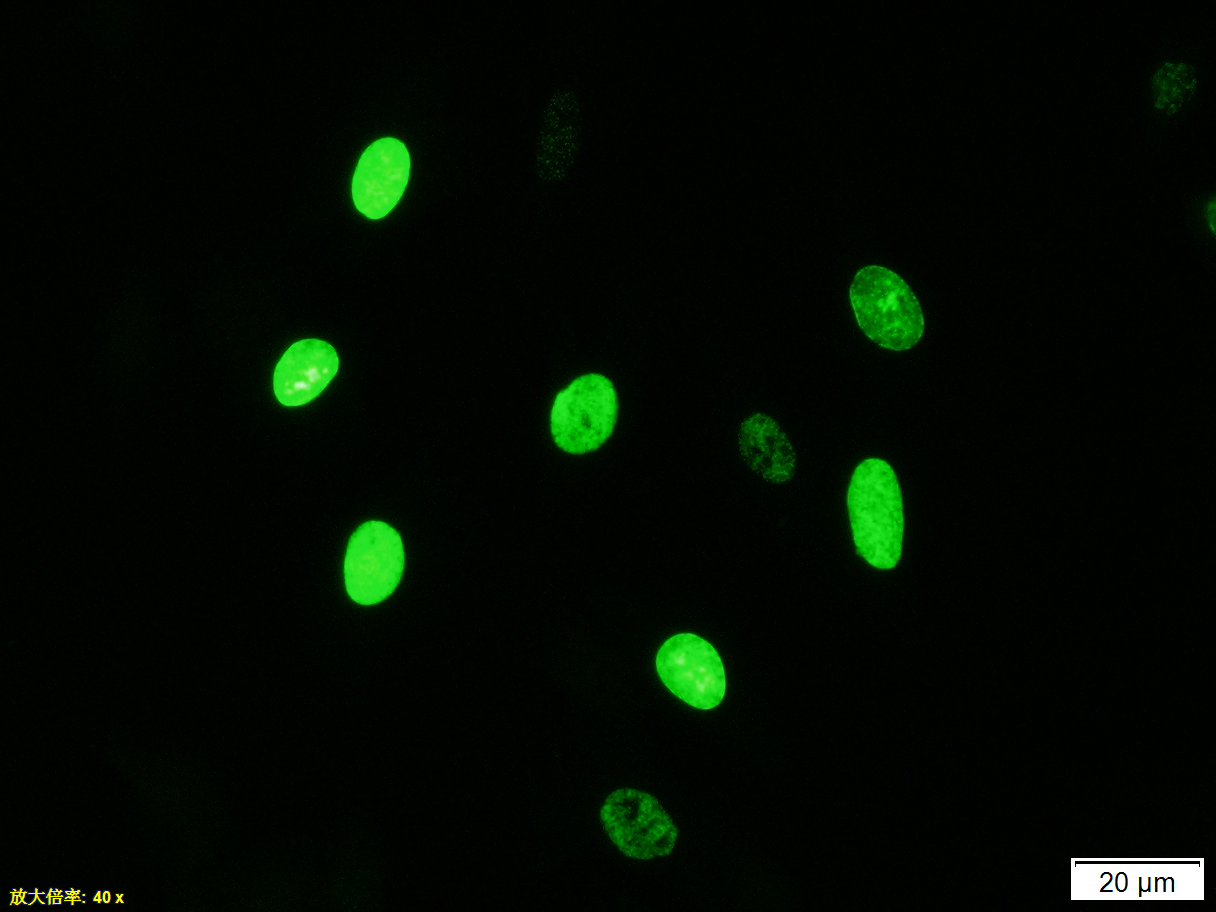

Supplement: S6 File — (ZIP) [file pone.0191616.s006.zip › Original data underlying the findings described in manuscript-TUNEL staining for detecting the apoptosis of CSCs-1/I-Exo+inhibiter group/fig_3-1.tif]

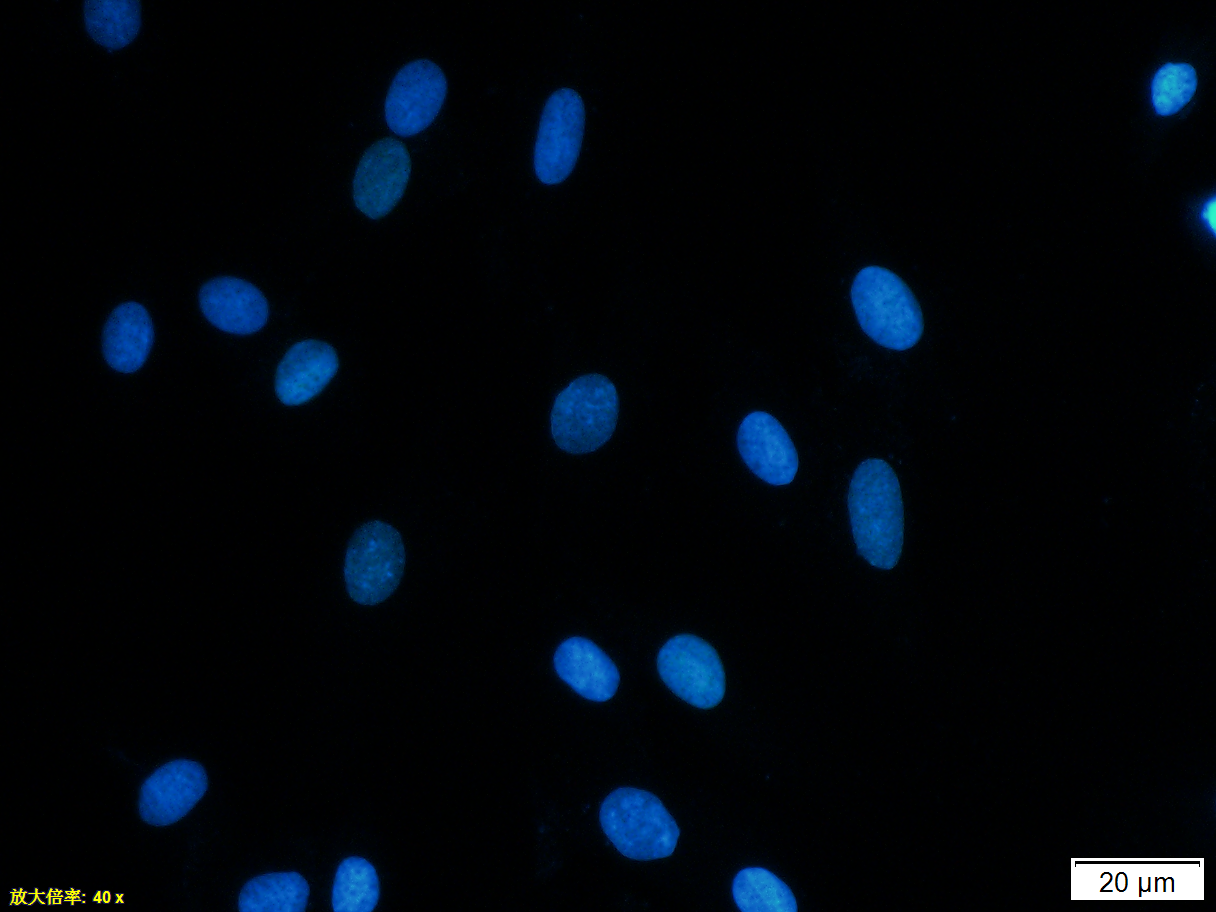

Supplement: S6 File — (ZIP) [file pone.0191616.s006.zip › Original data underlying the findings described in manuscript-TUNEL staining for detecting the apoptosis of CSCs-1/I-Exo+inhibiter group/fig_3-2.tif]
