# Supplementary figures and images for: Bone marrow mesenchymal stem cell-derived exosomal miR-21 protects C-kit+ cardiac stem cells from oxidative injury through the PTEN/PI3K/Akt axis (part 4 of 4)
Source: PLoS One. 2018 Feb 14;13(2):e0191616. doi: 10.1371/journal.pone.0191616 (PMC5812567; doi:10.1371/journal.pone.0191616)

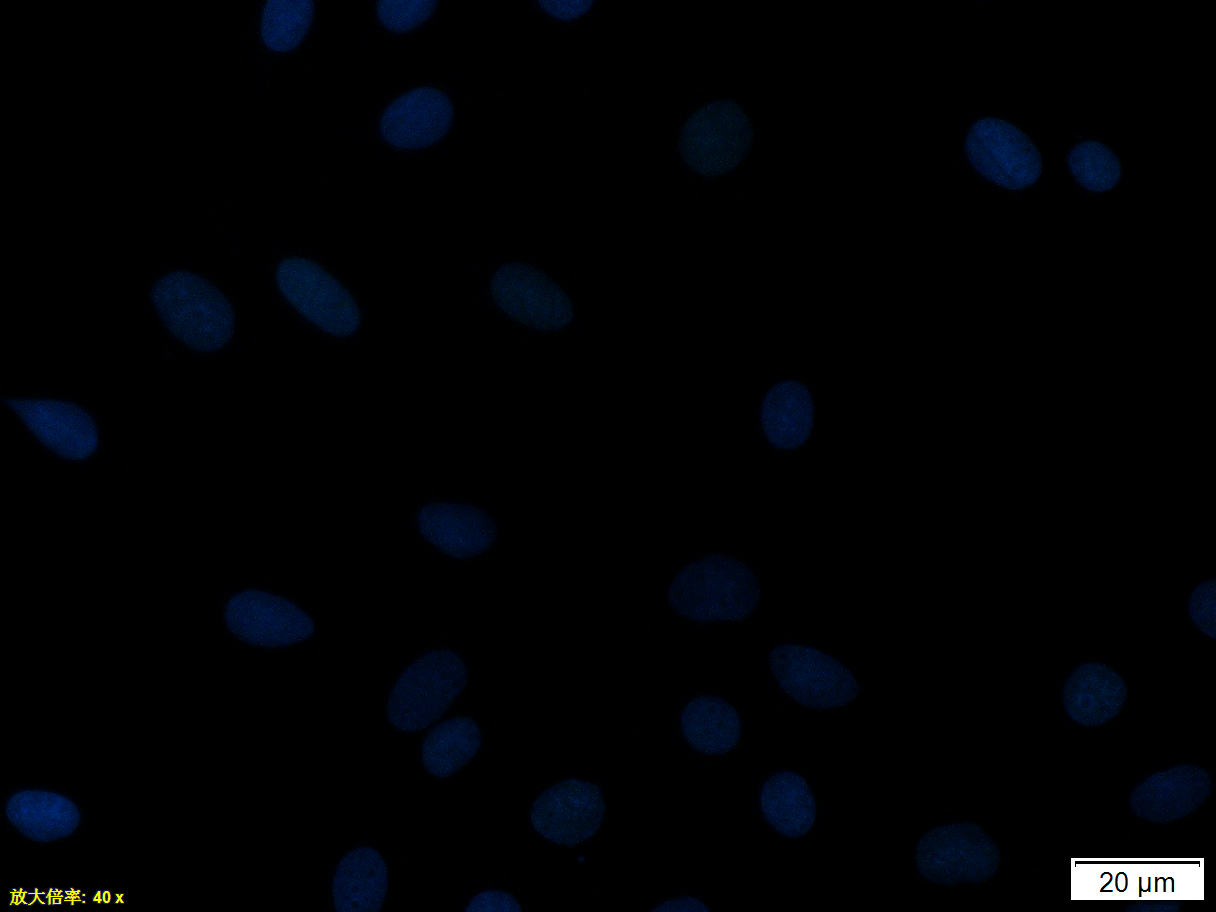

Supplement: S7 File — (ZIP) [file pone.0191616.s007.zip › Original data underlying the findings described in manuscript-TUNEL staining for detecting the apoptosis of CSCs-2/siRPTEN-INC group/fig_3-1.tif]

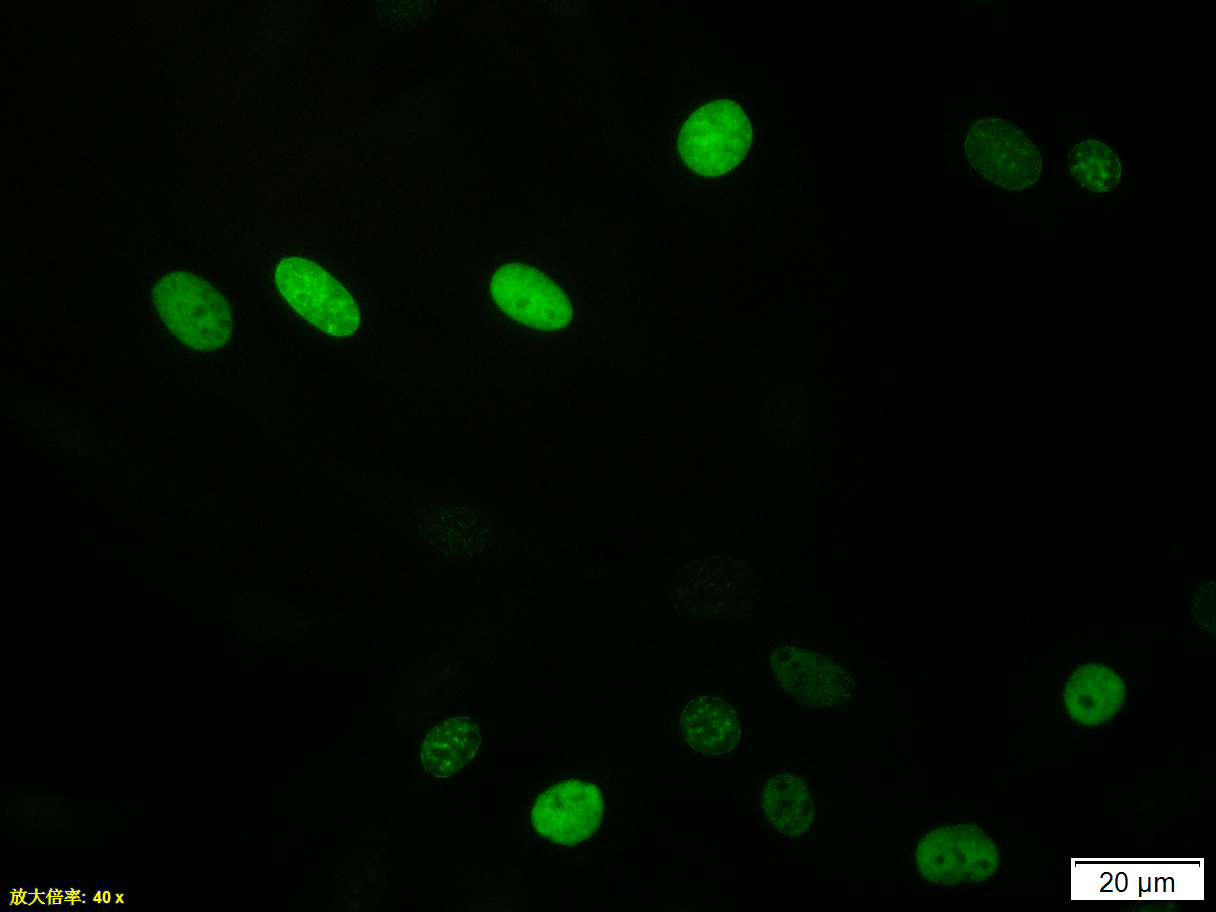

Supplement: S7 File — (ZIP) [file pone.0191616.s007.zip › Original data underlying the findings described in manuscript-TUNEL staining for detecting the apoptosis of CSCs-2/siRPTEN-INC group/fig_3-2.tif]

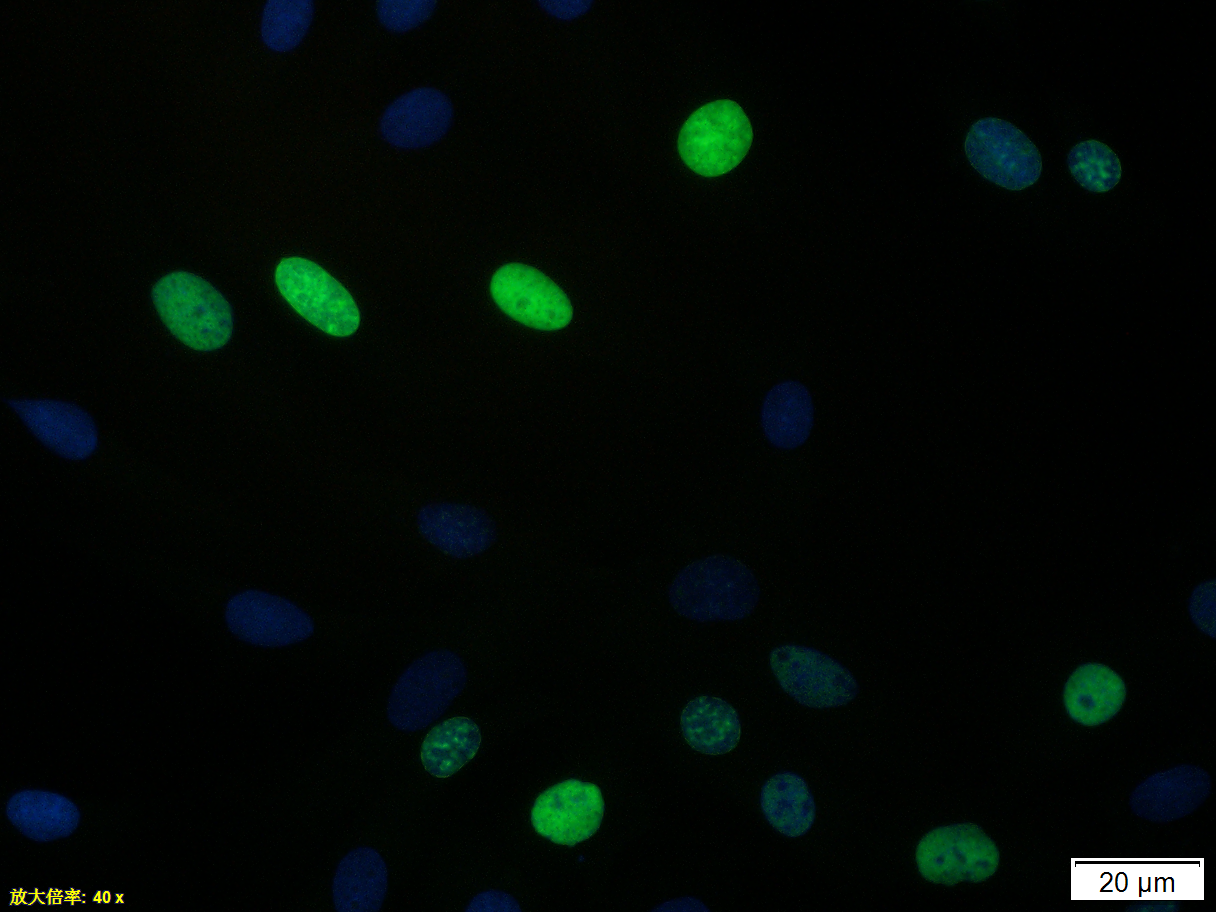

Supplement: S7 File — (ZIP) [file pone.0191616.s007.zip › Original data underlying the findings described in manuscript-TUNEL staining for detecting the apoptosis of CSCs-2/siRPTEN-INC group/fig_3.tif]

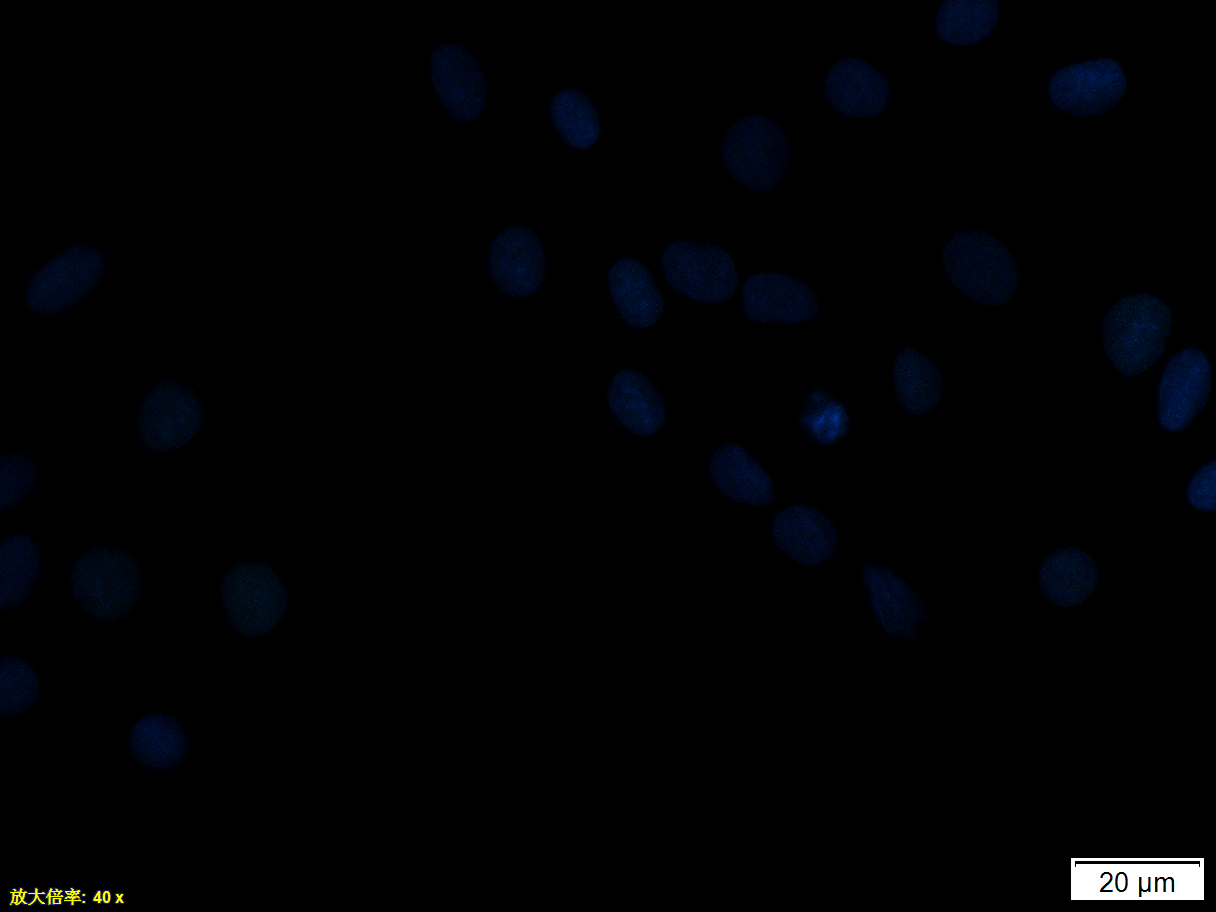

Supplement: S7 File — (ZIP) [file pone.0191616.s007.zip › Original data underlying the findings described in manuscript-TUNEL staining for detecting the apoptosis of CSCs-2/siRPTEN-INC group/fig_4-1.tif]

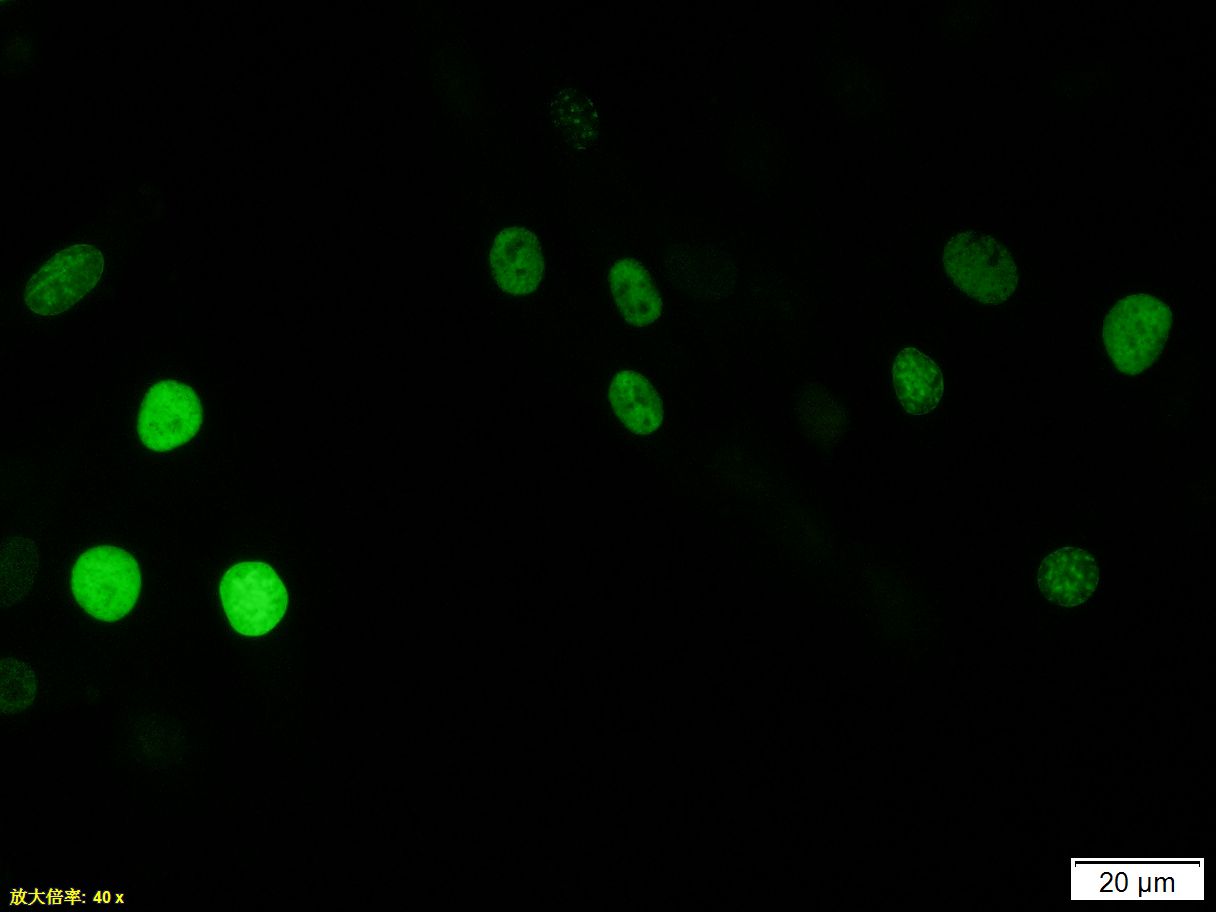

Supplement: S7 File — (ZIP) [file pone.0191616.s007.zip › Original data underlying the findings described in manuscript-TUNEL staining for detecting the apoptosis of CSCs-2/siRPTEN-INC group/fig_4-2.tif]

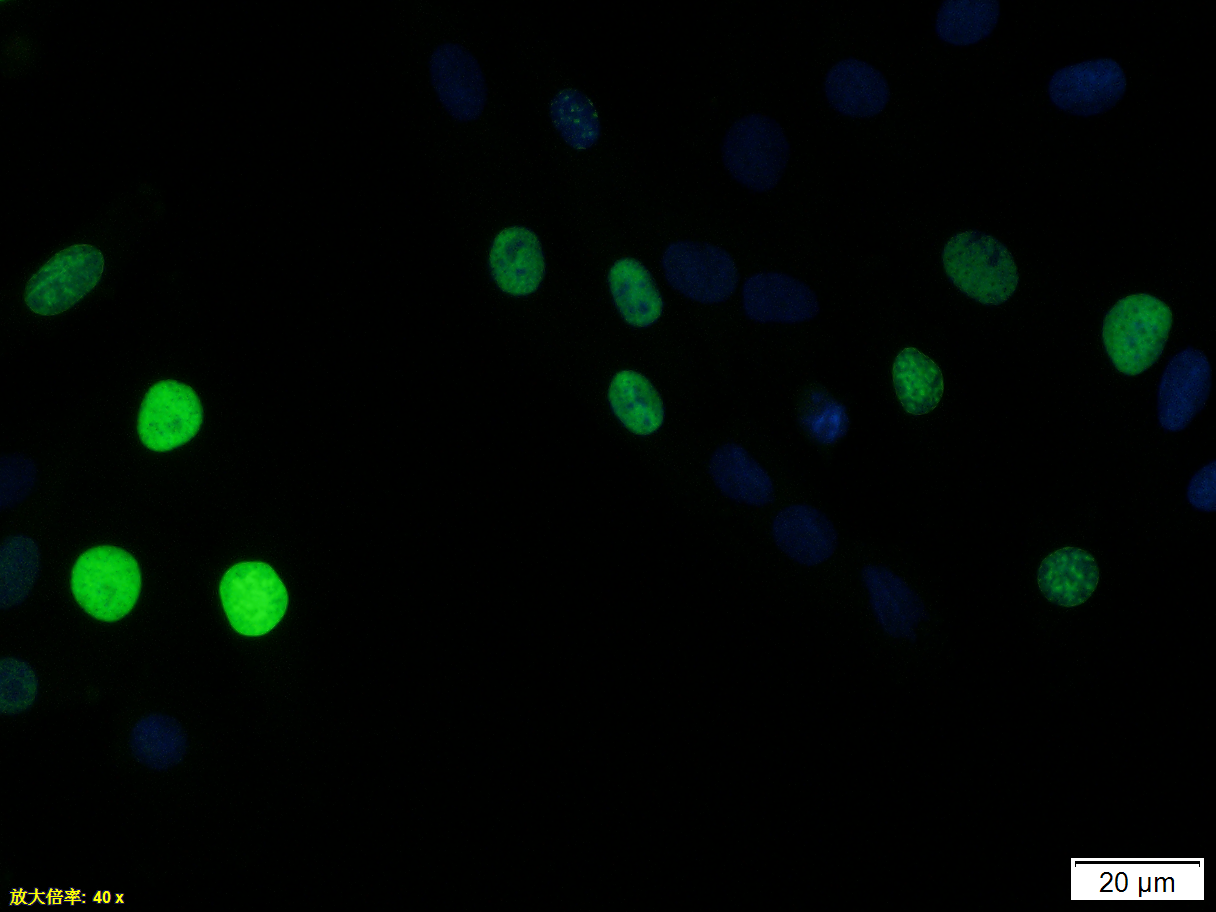

Supplement: S7 File — (ZIP) [file pone.0191616.s007.zip › Original data underlying the findings described in manuscript-TUNEL staining for detecting the apoptosis of CSCs-2/siRPTEN-INC group/fig_4.tif]

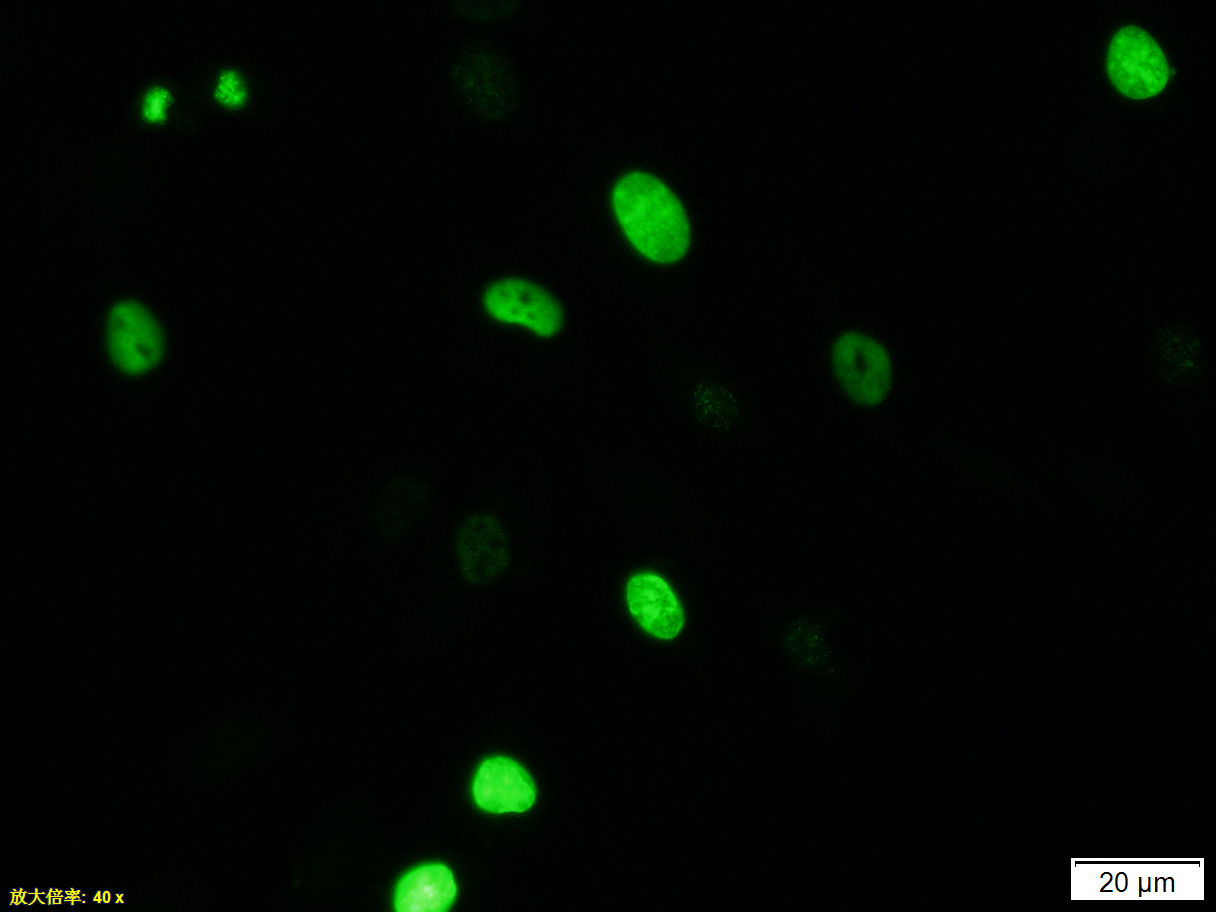

Supplement: S7 File — (ZIP) [file pone.0191616.s007.zip › Original data underlying the findings described in manuscript-TUNEL staining for detecting the apoptosis of CSCs-2/siRPTEN-INC group/fig_5-1.tif]

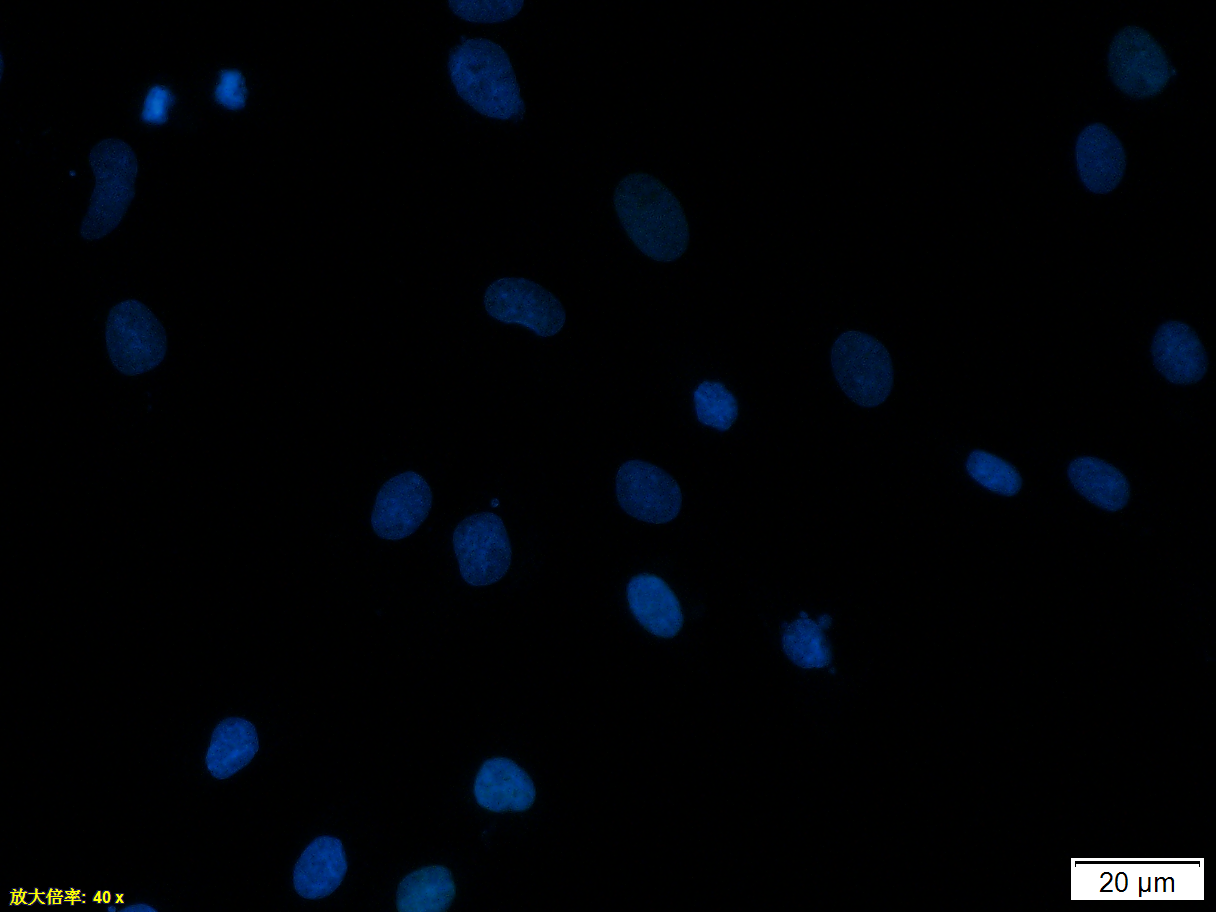

Supplement: S7 File — (ZIP) [file pone.0191616.s007.zip › Original data underlying the findings described in manuscript-TUNEL staining for detecting the apoptosis of CSCs-2/siRPTEN-INC group/fig_5-2.tif]

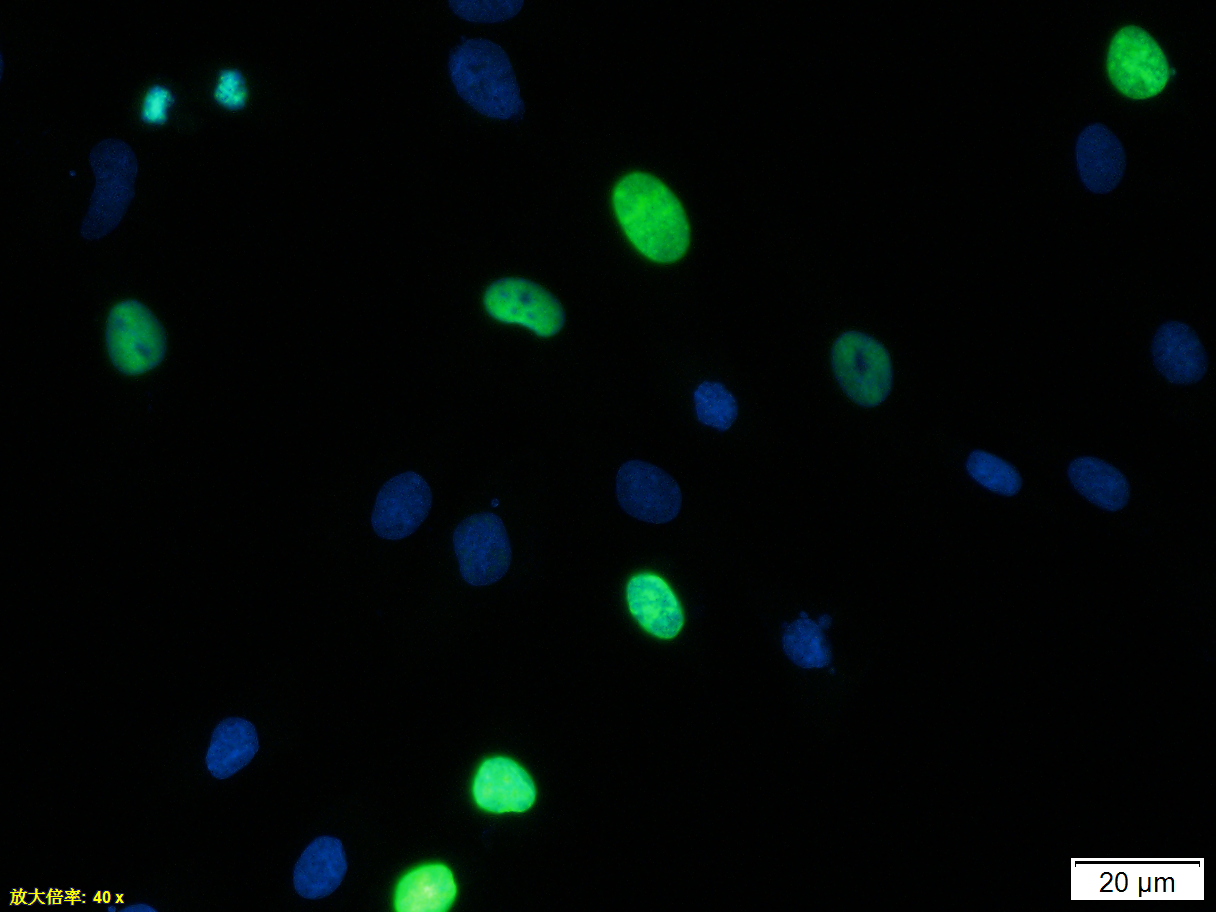

Supplement: S7 File — (ZIP) [file pone.0191616.s007.zip › Original data underlying the findings described in manuscript-TUNEL staining for detecting the apoptosis of CSCs-2/siRPTEN-INC group/fig_5.tif]

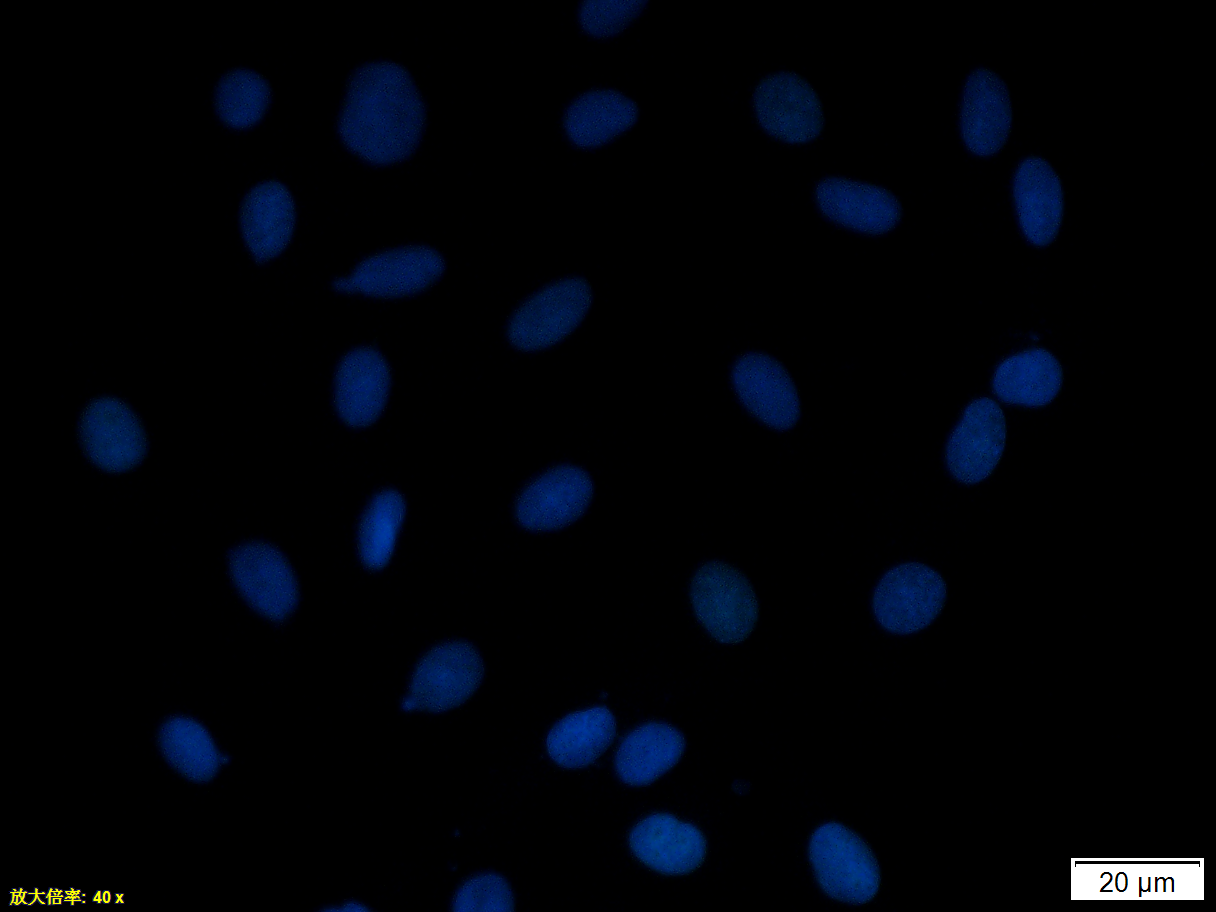

Supplement: S7 File — (ZIP) [file pone.0191616.s007.zip › Original data underlying the findings described in manuscript-TUNEL staining for detecting the apoptosis of CSCs-2/siRPTEN-INC group/fig_6-1.tif]

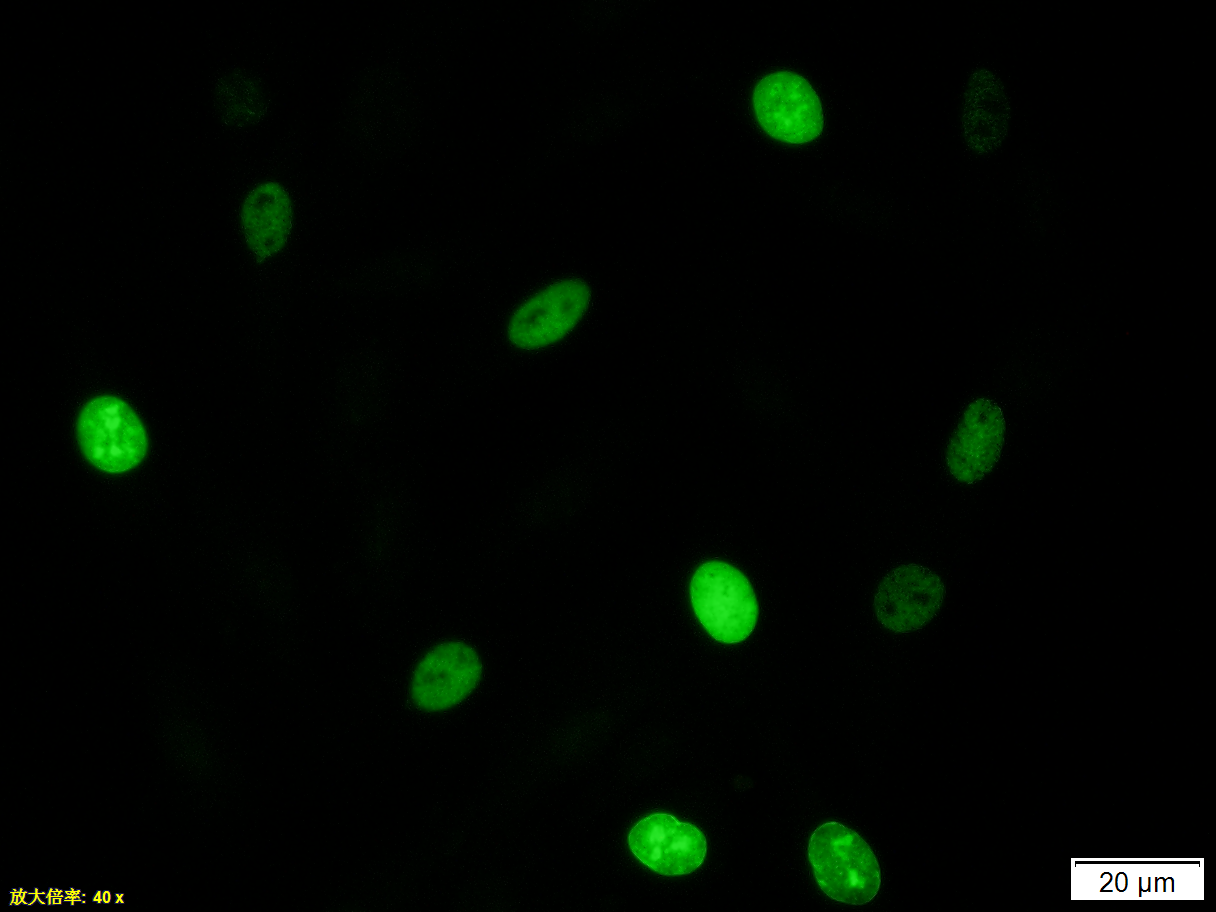

Supplement: S7 File — (ZIP) [file pone.0191616.s007.zip › Original data underlying the findings described in manuscript-TUNEL staining for detecting the apoptosis of CSCs-2/siRPTEN-INC group/fig_6-2.tif]

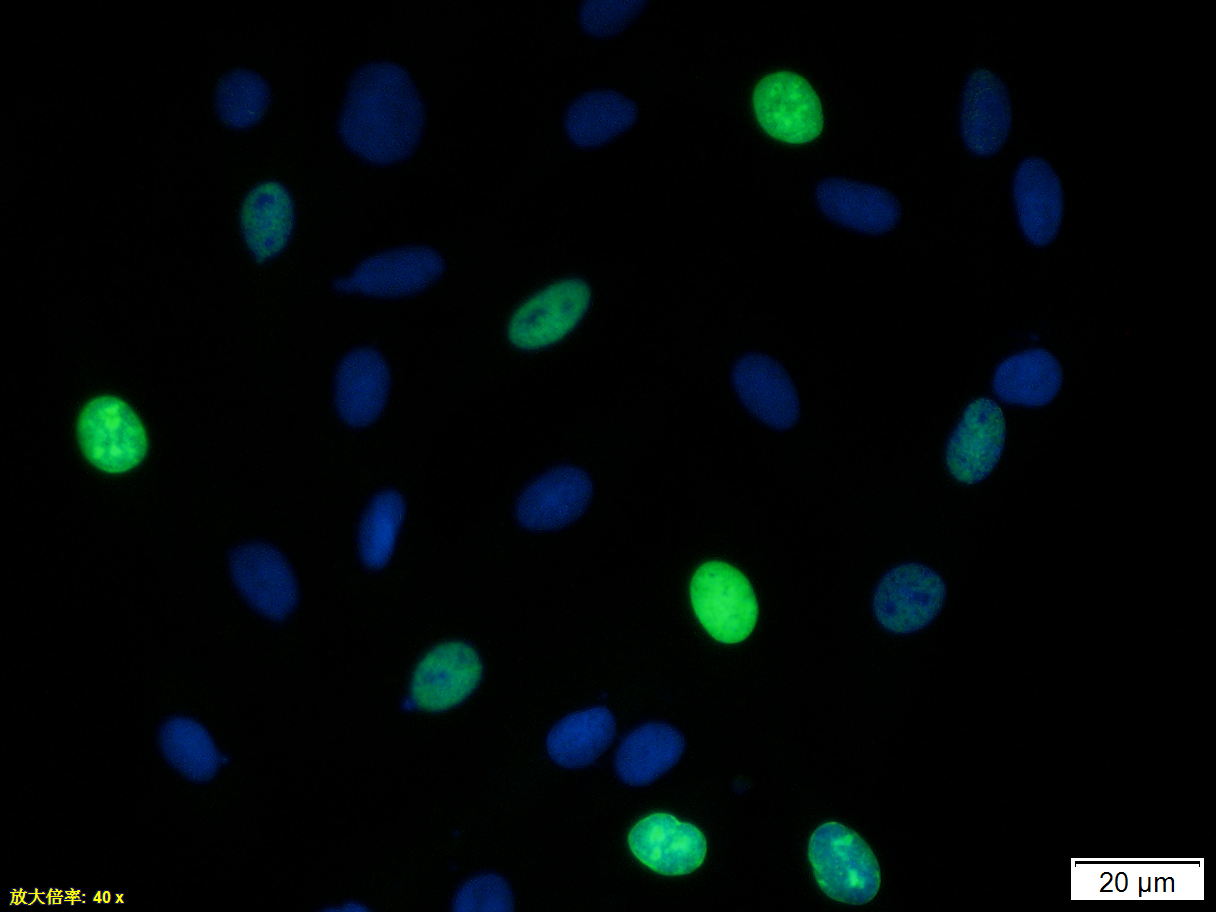

Supplement: S7 File — (ZIP) [file pone.0191616.s007.zip › Original data underlying the findings described in manuscript-TUNEL staining for detecting the apoptosis of CSCs-2/siRPTEN-INC group/fig_6.tif]
